# Mosaic Evolution of Molecular Pathways for Sex Pheromone Communication in a Butterfly

**DOI:** 10.3390/genes13081372

**Published:** 2022-07-31

**Authors:** Caroline M. Nieberding, Patrícia Beldade, Véronique Baumlé, Gilles San Martin, Alok Arun, Georges Lognay, Nicolas Montagné, Lucie Bastin-Héline, Emmanuelle Jacquin-Joly, Céline Noirot, Christophe Klopp, Bertanne Visser

**Affiliations:** 1 Evolutionary Ecology and Genetics Group, Earth and Life Institute, UC Louvain, 1348 Louvain-la-Neuve, Belgium; veroniquebaumle@hotmail.com (V.B.); gilles.sanmartin@gmail.com (G.S.M.); alok_arun@br.inter.edu (A.A.); georges.lognay@uliege.be (G.L.); 2 Center for Ecology, Evolution and Environmental Changes (cE3c) & Global Change and Sustainability Institute (CHANGE), Faculty of Sciences, University of Lisbon (FCUL), 1749-016 Lisboa, Portugal; pbeldade@fc.ul.pt; 3 INRAE, CNRS, IRD, UPEC, Sorbonne Université, Institute of Ecology and Environmental Sciences of Paris, Université de Paris, 78000 Versailles, France; nicolas.montagne@sorbonne-universite.fr (N.M.); lucie.bastin@laposte.net (L.B.-H.); emmanuelle.joly@inrae.fr (E.J.-J.); 4 Plateforme Bio-Informatique GenoToul, MIAT, INRAE, UR875 Mathématiques et Informatique Appliquées Toulouse, 31326 Castanet-Tolosan, France; celine.noirot@inrae.fr (C.N.); christophe.klopp@inrae.fr (C.K.); 5 Evolution and Ecophysiology Group, Department of Functional and Evolutionary Entomology, Gembloux Agro-Bio Tech, University of Liège, 5030 Gembloux, Belgium; bertanne.visser@uliege.be

**Keywords:** olfactory communication, desaturase, reductase, odorant receptor, odorant binding protein, chemosensory protein, PBAN, phylogeny

## Abstract

Unraveling the origin of molecular pathways underlying the evolution of adaptive traits is essential for understanding how new lineages emerge, including the relative contribution of conserved ancestral traits and newly evolved derived traits. Here, we investigated the evolutionary divergence of sex pheromone communication from moths (mostly nocturnal) to butterflies (mostly diurnal) that occurred ~119 million years ago. In moths, it is the females that typically emit pheromones to attract male mates, but in butterflies males emit pheromones that are used by females for mate choice. The molecular bases of sex pheromone communication are well understood in moths, but they have remained relatively unexplored in butterflies. We used a combination of transcriptomics, real time qPCR, and phylogenetics to identify genes involved in the different steps (i.e., production, regulation, and reception) of sex pheromone communication of the butterfly *Bicyclus anynana*. Our results show that the biosynthesis and reception of sex pheromones relies both on moth-specific gene families (reductases) and on more ancestral insect gene families (desaturases, olfactory receptors, odorant binding proteins). Interestingly, *B. anynana* appears to use what was believed to be the moth-specific neuropeptide Pheromone Biosynthesis Activating Neuropeptide (PBAN) for regulating sex pheromone production. Altogether, our results suggest that a mosaic pattern best explains how sex pheromone communication evolved in butterflies, with some molecular components derived from moths, and others conserved from more ancient insect ancestors. This is the first large-scale investigation of the genetic pathways underlying sex pheromone communication in a butterfly.

## 1. Introduction

The evolution of new life forms occurs through the transition from an ancestral to a descendant clade, where the new lineage generally shows a mosaic phenotype of conserved and newly evolved traits. Mosaic evolution is indeed a recurring pattern in paleontology [1,2,3]. For example, *Tiktaalik roseae,* believed to represent the transition from fishes to amphibians (the “fishapod”; ~375 Mya), shares some traits with more primitive sarcopterygian fishes (e.g., body scales, fin rays, lower jaw, and palate) and other traits more typical of tetrapods (e.g., a shortened skull roof, modified ear region, mobile neck, functional wrist joint, and other features) [4]. Investigating the genetic bases of ancestral and derived phenotypic traits is essential for obtaining a mechanistic explanation of how mosaic evolution takes place. Studies investigating the mechanistic basis of mosaic evolution have increased in the last decade, including recent genomic evolution analyses identifying patterns of gene loss, retention, and *de novo* evolution [5,6,7]. Other patterns in the genetic bases of trait evolution have suggested a role for hybridization between species [8,9,10] or co-option of molecular pathways that acquired new functions [11,12]. Derived phenotypic traits can thus be generated through different molecular mechanisms that need to be identified case-by-case.

Here, we focused on the genetic bases of divergence in sex pheromone communication during the evolutionary transition from moths to butterflies, which occurred ~119 Mya [13]. Sex pheromone communication is used by most insects, including butterfly species, for finding, identifying, and assessing the quality of potential mating partners [14,15,16,17,18]. Sex pheromone communication is under strong selection, because it determines mating success and consequently an individual’s contribution to the next generation [19,20,21,22,23]. Molecular pathways for sex pheromone biosynthesis, its regulation, and pheromone reception have been identified in several moth species [24,25,26,27,28,29,30]. Compared to other insects, moths appear to have evolved moth-specific genes or gene lineages involved in sex pheromone communication [25,27,30,31,32,33,34,35]. For biosynthesis, most moths use a limited number of enzymes for desaturation, chain-shortening, reduction, acetylation or oxidation of *de novo* synthesized saturated fatty acids [25,30,31,36], which generate tremendous chemical diversity of pheromone components and high species-specificity of pheromone blends [24,25]. Some of these enzyme families comprise Lepidoptera-specific subclades, such as the Δ9- and Δ11-desaturases [25,30,31]. Similarly, novel odorant receptor and odorant binding protein subclades have evolved in moths that bind specifically to sex pheromone chemicals [27,32,33,34,35]. We aimed to investigate whether butterflies use moth-specific molecular pathways, more ancestral insect pathways, and/or have evolved butterfly-specific molecular pathways for pheromone communication.

Butterfly sex pheromones have some ecological specificities unlike those of moths [17]. Female moths, for example, use sex pheromones to signal their location to mating partners in the dark or at dusk [22]. In contrast, butterfly sex pheromones are generally produced by males and the importance of sex pheromone communication in butterflies was less clear due to their diurnal lifestyle (see [17] for studies on male sex pheromones in moths). Studies on some butterfly species have, however, revealed that sex pheromone communication is important for determining mating success [17,37] and sex pheromones play an important role in speciation events [38]. Butterfly sex pheromones can indeed convey refined information as to the identity or quality of potential mating partners and can be critical for mate choice and species recognition [39,40]. We focused on the butterfly *B. anynana,* whose sex pheromone composition was previously identified and functionally validated [17,41]. Moreover, experimental manipulation, including the addition of synthetic sex pheromone perfumes [17,41], and artificial induction of “anosmia” [37,42] confirmed the importance of *B. anynana* sex pheromone for mating success. More than a hundred chemical components have been identified on *B. anynana* adult male and female bodies [43], but the composition of the male sex pheromone (“MSP” hereafter) consists of three main volatile components: (Z)-9-tetradecenol (MSP1), hexadecanal (MSP2) and 6,10,14-trimethylpentadecan-2-ol (MSP3) [17]. The identification and functional validation of these three MSPs and their role in *B. anynana* chemical communication set this species apart from other lepidopterans, where the role of specific chemical components often remains elusive in relation to fitness (but see [17,44] where specific components were identified). MSP1 and MSP2 are derived from fatty acids and are typically found in the pheromone blends of many moth species [25], which led us to hypothesize that the same genes as in moths are involved in sex pheromone communication of *B. anynana*.

**Figure 1:**
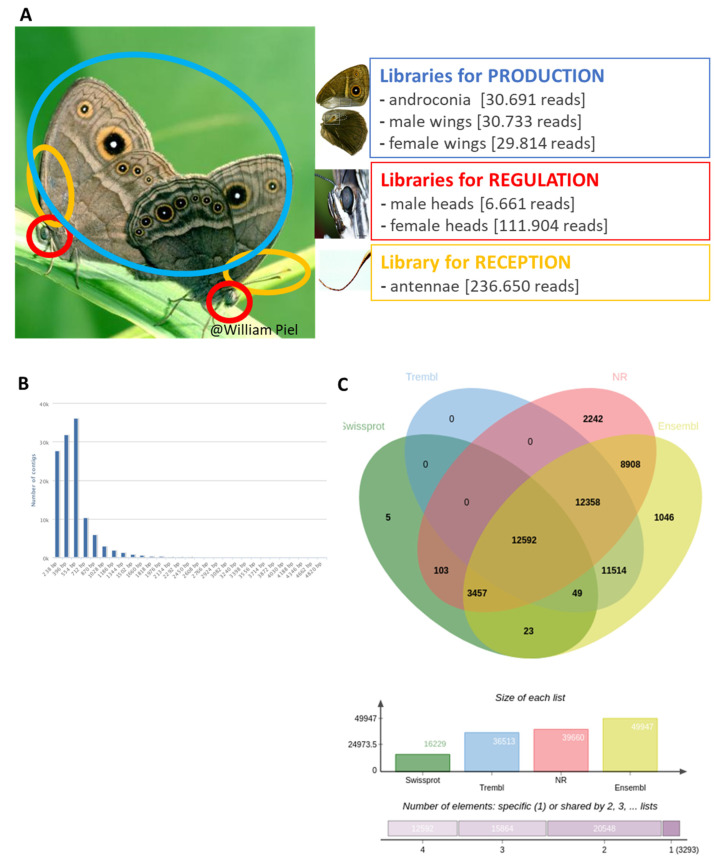
(**A**) Experimental design for the transcriptomics experiment showing the 3 steps involved in male sex pheromone communication and the corresponding tissues sampled to produce the RNA libraries (also including developmental libraries, not shown here). The number of sequenced reads per library is provided. (**B**) Information about the number of contigs in the transcriptome. (**C**) Venn diagram of annotated contig with regard to databases: Swissprot, Trembl, NR and 10 species of Ensembl Lepbase.

To investigate if butterflies use what were believed to be moth-specific gene families, we used RNA-seq to identify genes that could be involved in *B. anynana* pheromone production, regulation, and reception (Figure 1). We compared transcript abundance of sex pheromone-related adult tissues (male pheromone producing structures, heads and antennae) with control tissues (female wings and heads), and validated our findings with RT-qPCR. We identified specific candidate genes involved in the different olfactory communication functions and used phylogenetic analyses to identify the molecular origin of those genes. Our results reveal that sex pheromone communication in *B. anynana* evolved through a mosaic of ancestral insect genes, and more derived lepidoptera-specific genes.

## 2. Materials and Methods

### 2.1. Insects

*B. anynana* (Butler, 1879) (Lepidoptera: Nymphalidae) originated from an outbred wild type population that was collected in Malawi, Africa, in 1988 using 80 gravid females. Since 1988, several hundred individuals have been used each generation to maintain high levels of heterozygosity [45] in a climate-controlled room at a temperature of 27 °C, a relative humidity of 70%, and a photoperiod of L:D 12:12. Larvae were kept under these conditions on maize plants, *Zea mays*, and adults were fed mashed banana, *Musa acuminata,* for all experiments, except when stated otherwise.

### 2.2. Transcriptome

#### 2.2.1. Tissue Collection

For the transcriptome dataset, several hundreds of virgin males and females were separated at the pupal stage in different cages, and tissues collected in March 2010. Pupal tissues were collected from male and female pupae 1 to 7 days after pupation (1 or 2 pupae per day after pupation and per sex), after which the wing imaginal discs were dissected as described in [46]. Tissues for adult libraries (wings, heads and antennae) were collected from adult virgin males and females aged 1, 3, 5, 8, 10 and 14 days after emergence (Supplementary Figure S1): ~50 adults were used per age category and per library for wing libraries, ~10 adults were used per age category and per library for head tissues, and ~5 adult females and 5 males were used per age category for the antennae library. Brain tissue was obtained by cutting the head and cutting off the eyes, the proboscis and the antennae; antennal tissue was collected for a similar number of adult males and females (Figure 1). Dissected tissues were conserved immediately at −20°C in RNAlater (Sigma-Aldrich, Hoeilaart, Belgium).

#### 2.2.2. RNA Extraction

RNA of all dissections was extracted in April 2010, within a month after collection of tissues, in an RNA-free environment, on ice, and using the RNeasy Mini kit and the RNAase free DNAase kit (Qiagen, Venlo, The Netherlands). After RNA extraction, 1 µL of each RNA extract was used for testing RNA quality and quantity using a Bioanalyzer System (Agilent, Machelen, Belgium) at the LUMC hospital in Leiden (The Netherlands, courtesy of Dr Jeroen Pijpe), and the RiboGreen RNA quantification kit, respectively. The remaining RNA extract was stored at −80 °C for cDNA synthesis. For cDNA synthesis, we first pooled all RNA extracts dedicated to the same library in one tube per library, in such a way that: (*i*) the same amount of RNA was present for each sex (male and female), (*ii*) each life stage was represented by similar RNA amounts (days 1 to 7 after pupation for pupal tissue libraries; days 1 to 14 after emergence for adult tissue libraries; Supplementary Table S1). Total RNA yield was 27 to 40 µg per library as requested for sequencing.

#### 2.2.3. mRNA Isolation, cDNA Synthesis and Sequencing

mRNA capture, cDNA synthesis, and tagging for Titanium 454-sequencing was performed by Biogenomics, a KU Leuven Research & Development Division of the Laboratory of Animal Diversity and Systematics (Leuven, Belgium). Between 370 and 1340 ng (0.3 and 1.6%) mRNA yield was obtained for each library, providing enough mRNA (minimum 200 ng per library) for cDNA construction and tagging. Yet, cDNA synthesis failed when started from mRNA, which is why a SMART cDNA synthesis was performed from total RNA. A custom normalization step (based on the EVROGEN Trimmer kit) was optimized in collaboration with the Roche R&D department and applied to the cDNA libraries, as no validated normalization protocol was available from Roche in 2010 for Titanium cDNA sequencing. Each normalized library was quality checked for fragment length and integrity before sequencing. Each library was subjected to GS FLX Titanium Emulsion PCR and Sequencing, and each library was sequenced 5 times. After sequencing, data were processed through certified Roche software (GS Transcriptome Assembler/Mapper) and custom scripts for advanced analysis. Basic data analysis included read quality trimming and assembly into contigs, including potential alternative splicing products. The sequences were trimmed by removing low quality sequences, ambiguous nucleotides, adapter sequences, and sequences with lengths less than 20 nucleotides. The 454-sequencing generated 824,439 reads, with an average length of 293 base pairs and a total of 242,005,027 nucleotides (Supplementary Figure S2).

#### 2.2.4. Transcriptome Assembly, Quantification, and Annotation

Adaptors were removed with smartkitCleaner and adaptorCleaner. Raw sequences (reads) were cleaned with the software Pyrocleaner ([47], using the following criteria: *(i)* complexity/length ratio less than 40 (using a sliding window approach based on 100 bp sequence length, and a step of 5 bp); *(ii)* duplicate read removal (see bias associated with pyrosequencing, due to the random generation of duplicate reads); *(iii)* removal of too long/too short reads (maximum and minimum read length = mean read length ± 2 SD); *(iv)* removal of reads with too many undetermined bases (more than 4%). Contaminations were discarded by searching hits against *Escherichia coli*, phage and yeasts.

The reads were assembled de novo in 43,149 contigs of 488 base pairs on average with a total of 21,087,824 nucleotides (Supplementary Figure S2). The average GC content was 36.44%. The assembly was performed with tgic l.

(https://academic.oup.com/bioinformatics/article/19/5/651/239299) version 2.1 using standard parameters. The reads where realigned to the contigs and singletons with bwa aln version 0.7.2 using standard parameters and transformed in bam format, sorted and indexed with samtools version 0.1.19 with default parameters. The bam files were then processed with samtools idxstats to extract expression measures in the form of numbers of reads aligned on each contig for every condition. These measures were than merged to produce the quantification file using unix cut and paste commands. Diamond was used to search for sequence homology between contig and the following generalist databases: UniProtKB/Swiss-Prot, UniProtKB/TrEMBLRelease of April, NR release of end of March.

The following species from the ensemble database were queried: *B. anynana* (nBA.0.1), *Calycopis cecrops* (v1.1), *Danaus plexippus* (v3), *Heliconius melpomene melpomene* (Hmel2.5), *Junonia coenia* (JC v1.0), *Lerema accius* (v1.1), *Melitaea cinxia*, *Papilio machaon* (Pap_ma_1.0), *Phoebis sennae* (v1.1), and *Pieris napi* (v1.1).

#### 2.2.5. Candidate Gene Identification Using Transcriptome Sequencing

Numerous publications document gene expression studies focusing on chemical communication in Lepidoptera, but only three of these studies focused on butterflies [48,49,50], and butterfly sex pheromone communication has rarely been studied in this context [48]. Here, we produced six RNA libraries from different adult tissues that were specifically chosen to cover the different steps of male pheromone communication (Figure 1): pheromone biosynthesis (which occurs in dedicated structures on male wings, called androconia) [17], its neuro-regulation (in the brain), and pheromone reception (in antennae). Approximately 500 male and female *B. anynana* adults were dissected and relevant tissues assigned to different libraries (Figure 1A). For pheromone synthesis, we compared transcripts in male androconia (Library “androconia”) with those in remaining adult male wing parts (Library “male wings”) and adult female wings (Library “female wings”) as controls. For regulation of pheromone communication, we compared transcript abundance between adult male heads (where the regulation of pheromone synthesis takes place; Library “adult male heads”) and adult female heads (Library “adult female heads”, control). For pheromone reception, we compared transcripts between adult male and female antennae (the tissue where pheromone reception takes place) [17]; Library “antennae”) and adult heads (Libraries “male heads” and “female heads”) as controls. Two other libraries were also analyzed, corresponding to pupal wings in males (Library “pupal male wings”), and females (Library “pupal female wings”), but these data will not be discussed here. We focus solely on adults, the stage during which pheromone communication takes place. A total of 737,206 reads were obtained from the different tissues sampled in *B. anynana* and were assembled into 43,149 contigs, with 76,818 remaining non-assembled singletons (Figure 1B,C, Supplementary Table S2). Transcripts were annotated based on reference genomes for several butterfly species (including *B. anynana*; [51]), as well as other relevant insect species. Using the digital differential display (DDD) tool (of NCBI’s UniGene database; *p* < 0.05), a total of 422 contigs were found to be differentially expressed when tissue-specific libraries were compared (Supplementary Table S2). Expression differences were validated by real time quantitative PCR analyses on 10 selected candidate chemical communication genes, showing that relative differences in expression levels in our transcriptome dataset matched those quantified by RT-qPCR (Supplementary Figure S3).

#### 2.2.6. Identification of Specific Gene Families

We also mined the transcriptome for specific families of genes supposedly involved in sex pheromone communication based on the available evidence in moths: desaturases, reductases, odorant receptors (OR), odorant binding proteins (OBP), and chemosensory proteins (CSP). To do so:

*(i)* we downloaded the DNA sequence of every *B. anynana* contig named as a desaturase, reductase, OR, OBP or CSP in our transcriptome;

*(ii*) we checked the homology of the sequence of each candidate contig with gene members of the same family in other Lepidoptera by performing a blastx in Genbank;

*(iii*) every *B. anynana* contig that showed significant homology in step *ii* was blasted in the transcriptome, allowing us to find more *B. anynana* ESTs of the same gene family, even if some had not been annotated as such. All these contigs and ESTs were then “candidate members of each respective gene family”. If no significant homology was found using blastx in step *ii*, the sequence was removed from the list of candidate members of the gene family;

*(iv*) every *B. anynana* contig and EST candidate was then translated into an amino acid sequence using Expasy (https://web.expasy.org/translate/). When necessary, cdd analyses of domains were done. Using this procedure, 27 OR, 44 OBP and 70 CSP candidate members were found in the *B. anynana* transcriptome (Supplementary Tables S3–S5 for OR, OBP and CSP, respectively; for reductases and desaturases see Results). For example, 40 contigs were initially annotated as “odorant-binding protein” in our transcriptome, based on the characteristic hallmarks of the OBP protein families, including six highly conserved cysteines, i.e., “C” (in Lepidoptera C1-X25-30-C2-X3-C3-X36-42-C4-X8-14-C5-X8-C6, with “X” being any amino acid) [52]. As sequence conservation between OBPs is low, i.e., between 25 and 50% identity for amino acid sequences, manually mining the transcriptome allowed us to find another seven OBP candidate members (Supplementary Tables S3–S5 for OR, OBP and CSP, respectively).

*(v)* Candidate members were then manually aligned in Bioedit to group them into distinct expressed gene units, or unigenes: 17 Bany_OR unigenes (Supplementary Table S3), 9 Bany_OBP unigenes, including in some cases more “gene subunits” when contigs were similar enough to suggest that they represented different allelic variants of the same gene, such as Bany_OBP3, Bany_OBP4, Bany_OBP6 (Supplementary Table S4) and 8 Bany_CSP unigenes with some more gene subunits as well (Supplementary Table S5).

*(vi)* The expression level of each candidate unigene across libraries was then obtained by pooling the number of copies in the *B. anynana* transcriptome of each EST and contig forming the unigene.

### 2.3. Phylogenies

For the OR phylogeny, amino acid sequences found in the *B. anynana* transcriptome were aligned with OR sequences previously identified in the genomes of *Bombyx mori* and *H. melpomene* [53] and in antennal transcriptomes of *Cydia pomonella* [54] and *Spodoptera littoralis* [55]. Alignment was performed with MAFFT v7 (https://mafft.cbrc.jp/alignment/server/), and the maximum-likelihood phylogeny was built using PhyML 3.0 [56]. Branch support was assessed using a likelihood-ratio test [57]. Published datasets of Lepidoptera protein sequences from previous phylogenetic studies were used for testing the phylogenetic position of *B. anynana* ORs [58] (Supplementary File S1), OBPs [59] (Supplementary File S2), desaturases and reductases [36] (Supplementary Files S3 and S4).

### 2.4. Real Time Quantitative PCR

For biological replicates, mRNA was extracted either from a single individual or formed by pooling 3 to 5 individuals of various ages in experiments for the “reception” and the “production” communication steps, respectively. Each treatment is represented by 3 to 7 biological replicates. The protocol used for quantitative real time PCR is described in [60]. Briefly, total RNA was extracted using the RNeasy Mini kit following manufacturer’s instructions. Residual DNA was removed after treating extracted RNA using a DNase enzyme. A nanodrop ND-1000 spectrophotometer was then used to assess the integrity of the RNA before conversion into cDNA. qRT-PCR was carried out using the SYBR green dye in a 96-well thermocycler with parameters described in [60]. Primer sequences for all genes are available in Supplementary Table S6. Relative transcript abundance was calculated using the 2^−∆∆*Ct*^ method. Statistical significance of differences in expression levels expressed in Rq values after log-transformation was tested using nested ANOVA with technical replicates nested with biological replicates; the model was log (Rq) ~ treatment/biological replicate/technical replicate + Error (tissue/biological replicate/technical replicate). R version 3.6.1 [61] was used for statistical analyses.

### 2.5. Behavioral Experiments

#### 2.5.1. Mating Experiments for Quantifying Odorant Receptor Expression Levels

Naïve virgin females were reared in isolated conditions (devoid of the male secondary sexual traits putatively involved in sexual communication, i.e., olfaction, vision and audition) directly after egg collection. The virgin sensitized females were reared in a MSP-containing environment near cages containing males (and thus exposed to the sex pheromones of males). The sensitized mated females were reared in a MSP-containing environment and mated at an age of 3 days. All females were sacrificed at day 5 and the antennal tissues were used for RNA extraction and RT-qPCR analysis (described in Section 2.4).

#### 2.5.2. Daily Variation in Courtship Activity

We tested whether courtship activity in *B. anynana* males varies throughout the day. A large number of individuals were reared and age after emergence was recorded. The day before the experiment, 5 males and 4 females between 2 and 12 days old were randomly chosen and grouped in a cage (40 cm × 30 cm, cylindrical). The cages were placed in a room with a temperature of ~27 °C with natural light, and a 14:10 day-night regime. The butterflies were fed with banana slices and had access to water during the course of the experiment. We used 5 cages per trial and produced 3 trials with different individuals. A generalized mixed model with binomial error distribution was used to characterize the courting activity of males during the day. The presence/absence of courtship behavior for each male during 15 min of observations per hour was used as the dependent variable. As we expected courtship activity to peak at some time point during the day, we used “time of the day” (in the number of hours after natural sunrise) and its second order polynomial as a fixed explanatory variable. The age of males (in days) was also included as a fixed cofactor to control for the effect of age. The identity of each individual, cage and trial were used as random effects with individual nested within cage and cage nested within trial. We tested the model parameters with type III likelihood ratio tests, in which a model without the explanatory variable of interest is compared to the full model, both models being estimated by Maximum Likelihood.

#### 2.5.3. Daily Variation in Male Sex Pheromone Production

A number of butterfly couples were set up using adult virgin stock males and females. Three families were started from 3 couples that produced over 200 offspring. The 3 families were each partly reared into 2 different climate rooms that differed in the onset of artificial daylight (one at 9:00 a.m., the other at 12:00 p.m.). This allowed us to control for the potential effect climate cell-specific conditions on variation in MSP production. Forty to 80 males that emerged the same day were selected per family. MSP production of 8-day old males was sampled, an age at which each MSP component is produced in measurable quantities [17]. Four to 7 males of each family were killed and conserved at −80° for subsequent pheromone analysis at 7 sampling points during the day: 1, 4, 8, 11, 13, 18 and 23 h after the onset of daylight. MSP production was measured as described below in the Section 2.6. We used mixed models with normal error distribution to characterize the variation of MSP production during the day. The titre of each MSP component and the ratios between pairs of MSP components were used as dependent variables. MSP titres were square root transformed and MSP ratios were log-transformed to improve the normality and homoscedasticity of the residuals. As we suspected MSP production to peak at some time point during the day, we used a second order polynomial equation with time and time² as a fixed explanatory variables and family as a random effect. We tested model parameters with type III likelihood ratio tests, in which a model without the explanatory variable of interest is compared to the full model, both models being estimated by Maximum Likelihood. We estimated the percentage of variation explained by the models and each of their components with pseudo R² based on ratios of sums of squared residuals. We followed [62] for the variance decomposition procedure.

### 2.6. Quantification of Male Sex Pheromone Production

MSP concentrations were determined as previously described [17,63]. In short, one forewing and one hindwing of each male were soaked in 600 ul of hexane during 5 min. One ng/μL of the internal standard (palmitic acid) was then added. Extracts were analyzed on a Hewlett-Packard 6890 series II gas chromatograph (GC) equipped with a flame-ionization detector (FID) and interfaced with a HP-6890 series integrator, using nitrogen as carrier gas. The injector temperature was set at 240 °C and the detector temperature at 250 °C. A HP-1 column was used and temperature increased from the initial temperature of 50 °C by 15 °C/min up to a final temperature of 295 °C, which was maintained for 6 min.

## 3. Results and Discussion

### 3.1. B. anynana Sex Pheromone Biosynthesis

*B. anynana* was the first butterfly for which molecular pathways underlying sex pheromone biosynthesis were investigated and compared to those of moths [36]. In this study, one gene related to pheromone communication was highly expressed in *B. anynana* male androconial wing tissues compared to male and female control wing samples (i.e., ‘type 1′ contigs in Supplementary Table S2): an aldose reductase-like gene. This gene was also highly expressed in the male androconial wing tissue alone. Moreover, a Δ9-desaturase gene was also found to be highly expressed in this library. In contrast to earlier findings in *B. anynana* [36], no fatty-acyl reductase (FAR), nor Δ11-desaturase were found to be highly expressed in male androconial wing tissue (Supplementary Table S2).

Desaturases (that add a double bond to fatty acid substrates) were previously found to be involved in *B. anynana* MSP biosynthesis [36]. Therefore, we extended our search for desaturase genes for each of the libraries separately. We focused specifically on Δ9 and Δ11-desaturases, because these enzymes play an important role in moth pheromone biosynthesis [30,36]. Previous work with *B. anynana* suggested that a Δ11-desaturase is involved in the production of MSP1 [36]. Both Δ9- and Δ11-desaturase were present in the transcriptome, mainly in antennae. A phylogenetic tree containing our Δ9- and Δ11-desaturase contigs revealed a similar position within the larger desaturase phylogenetic tree, compared to earlier work [36] (Supplementary Figure S4).

To get more insight into the role played by the Δ9 and Δ11 desaturase gene, we used RT-qPCR (as in [60]) to compare transcript abundance between different adult wing tissues, the main tissue producing MSP1 (using RNA extracted from new samples). Δ9-desaturase transcript abundance was approximately four-fold higher than that of Δ11-desaturase (Supplementary Figure S3). When comparing the spatial distribution of MSP1 on *B. anynana* body parts with our RT-qPCR data for the two Δ-desaturase genes (Supplementary Figure S5A), the expression profile of the Δ9-desaturase gene, but not the Δ11-desaturase gene, matched MSP1 distribution (Supplementary Figure S5B,C, respectively). Indeed, the Δ9-desaturase gene showed overall significant variation in transcript abundance across tissues that correlated with the distribution pattern of MSP1 (Supplementary Figure S5). Specifically, Δ9-desaturase was found to be significantly expressed in male wing parts containing the androconia that produce MSP1, compared to remaining male wing tissues and female wings. Moreover, Δ9-desaturase gene expression was also found to be significantly expressed in male head tissue containing MSP1. No such match between gene expression and MSP1 abundance was found for the Δ11-desaturase gene, which showed no significant variation in transcript abundance across tissues known to contain MSP1 (Supplementary Figure S5). Altogether, these findings suggest that a Δ9 desaturase plays a role in *B. anynana* pheromone biosynthesis.

We then searched for genes from a second gene family known to be involved in sex pheromone production in *B. anynana*: fatty acyl reductases (*far*) that convert fatty-acyl pheromone precursors to alcohol [36]. While more than 20 FARs have been experimentally characterized from 23 moth and butterfly species, all FARs implicated in moth and butterfly sex pheromone biosynthesis are restricted to a single clade, suggesting that one FAR group was exclusively recruited for pheromone biosynthesis [64,65]. In our transcriptome, two reductase contigs were annotated and identified in male and female antennae: enoyl-CoA reductase and fatty-acyl reductase 1, *far 1*. As *far1* and another fatty-acyl reductase, *far2*, were previously found to be involved in MSP2 and MSP1 biosynthesis, respectively [36], we manually mined our transcriptome for *far1* and *far2* contigs by n-blasting *far1* and *far2* specific gene sequences. Contigs matching *far1* were largely expressed in androconia (171 copies), compared to wing controls (0 copies; Supplementary Table S7). While contigs matching *far2* showed an overall low expression level in wing tissues (Supplementary Table S8), a previous qRT-PCR study revealed that *far2* gene expression matched MSP1 biosynthesis [60], highlighting the potential importance of *far2* for *B. anynana* pheromone production.

The low expression level of *far2* is surprising given the amount of MSP1 present on male wings (2 µg/individual on average); hence we suggest that alternative candidates for MSP1 biosynthesis could be aldo-keto reductases, two of which are among the most expressed genes in androconial male wing tissues (Supplementary Table S2). Indeed, fatty-acyl reductases are usually associated with the reduction of aldehyde into alcohols producing various sex pheromone components in moths, but aldo-keto reductases are regularly found highly expressed in sex pheromone transcriptomes of moth species [66,67,68,69]. Guo et al., (2014) [70] and Yamamoto et al. (2016) [71] have proposed that aldo-keto reductases are involved in sex pheromone biosynthesis of the moths *Helicoverpa armigera* and *B. mori* by reducing 9-hexadecenal, 11-hexadecenal and 10E,12Z-hexadecadienal into alcohol. Our expression data suggest that an aldo-keto reductase, with or without *far2*, may be involved in MSP1 biosynthesis.

### 3.2. B. anynana Sex Pheromone Reception

The genomes of the butterflies *Danaus plexippus* and *H. melpomene* (i.e., species for which phylogenies of odorant receptor genes were available) have revealed a large number of genes belonging to families involved in olfactory reception in moths, including odorant receptors and odorant binding proteins [48,53,72]. Specifically, the odorant receptor and odorant binding protein gene families contain lineages specialized in the detection of sex pheromones in moths, the so-called pheromone receptors (PRs) and pheromone-binding proteins (PBPs) [35,59,73,74]. ORs are transmembrane receptors that bind volatile chemicals and are responsible for signal transduction in insect olfactory sensory neurons. They exhibit various response tuning breadths, and moth ORs involved in pheromone detection are often (but not always) highly specific to one or a few pheromone components [74]. Therefore, we expected to identify ORs binding to each of the three known chemical components of the *B. anynana* male sex pheromone: MSP1, 2, and 3 [17]. We identified the obligatory co-receptor “Orco” and 16 ORs in the transcriptome, some of which were highly expressed in antennae compared to other adult tissues (Supplementary Table S3).

**Figure 2:**
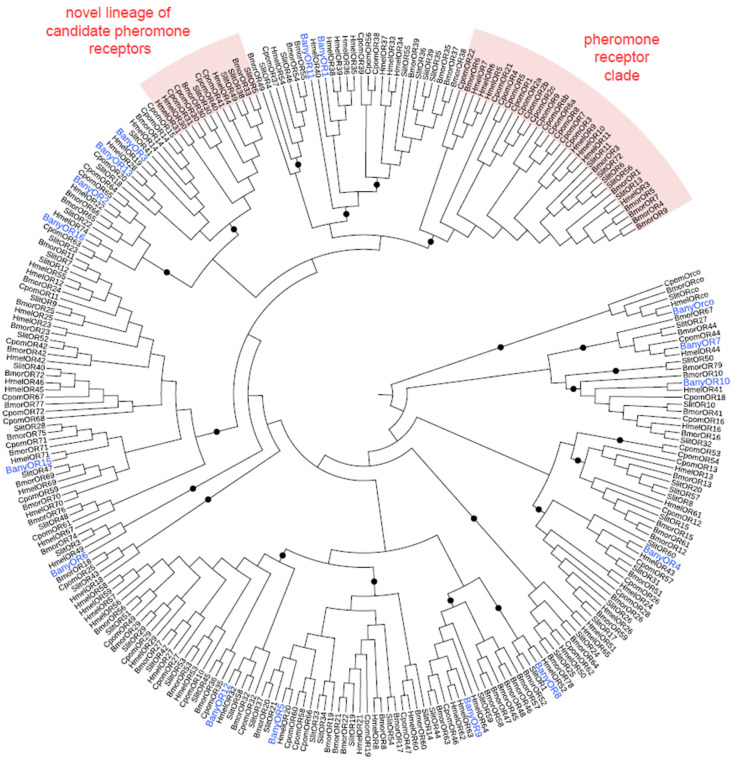
Maximum-likelihood phylogeny of Lepidopteran odorant receptors (OR), including the 16 ORs found in the *B. anynana* transcriptome (BanyOR, in blue). Bmor, *Bombyx mori*; Cpom, *Cydia pomonella*; Hmel, *Heliconius melpomene*; Slit, *Spodoptera littoralis*. Black circles indicate branchings highly supported by the approximate likelihood-ratio test (aLRT > 0.95).

Phylogenetic analysis revealed that ORs expressed in *B. anynana* were distributed among various lepidopteran OR lineages [53], but none were located in the classically defined sex pheromone receptor clade [35,75] (Figure 2). This suggests that *B. anynana* sex pheromone reception may have evolved from lepidopteran OR lineages other than the sex pheromone lineage.

**Figure 3:**
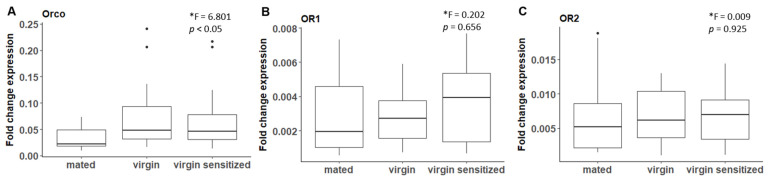
RT-qPCR mRNA expression level of olfactory receptors (ORs) in antennae of female *B. anynana* with different mating status. Orco (**A**), but not OR1 (**B**) or OR2 (**C**), mRNA level differed significantly in virgin naïve (middle) and virgin sensitized (right) compared to mated (left) females. Each treatment is the mean of 3 to 7 biological replicates. A nested ANOVA was used to test for differences between groups. F and *p* values are included for each graph. * log transformed data.

Recent studies have revealed that moth PRs do not constitute a monophyletic clade and, instead, evolved several times during OR evolution [35,76]. Functional PRs that have been found outside of the PR clade in some moth species were identified based on their sex-biased expression. We, therefore, searched for potential *B. anynana* PRs by quantifying the mRNA expression levels between sexes using RT-qPCR, expecting that PR in *B. anynana* should show higher expression in male compared to female antennae. We further expected that gene expression levels would correlate with temporally varying physiological and biological needs. In moth species, PRs are critical for detecting the female sex pheromone and the male’s behavioral and physiological responses to female sex pheromones were shown to be affected by moth age and mating status [77,78]. Therefore, we tried to identify *B. anynana* candidate PRs by comparing RNA expression levels in females with different mating status (using RT-qPCR). We expected that virgin females that had developed either in isolation (naive “virgin”) or in the presence of male scent (“virgin sensitized”) would exhibit higher expression levels for OR genes responsible for detecting the male sex pheromone, compared to mated females (“mated”) [79,80]. This difference would be due to virgin females taking information about the composition of the male sex pheromone for choosing mates regarding their inbreeding level or their age, and because recently mated females are much less receptive to courtship attempts in *B. anynana* [41,42]. The candidate genes Ban_OR1, Ban_OR2 and Ban_Orco were selected for RT-qPCR experiments because these genes displayed the highest expression among the 16 identified candidate ORs and were significantly expressed in antennae compared to control libraries (Supplementary Table S3). Orco expression was significantly decreased in mated compared to virgin (naïve or sensitized) females, but Bany_OR1 or Bany_OR2 were not (Figure 3), suggesting that regulation of the expression of Orco could be a mediator of sex pheromone receptivity. Orco, and not specific parts of the odorant receptor dimer, such as OR1, OR2 or other ORs that we did not test here, could be regulated by sex pheromone communication, similar to what was previously found in cockroaches [77,81].

In addition to the work described above, we aimed to functionally investigate if some specific OR candidate genes were responsible for the detection of male pheromone components using heterologous expression in *Drosophila melanogaster* olfactory sensory neurons coupled to electrophysiological recordings. These experiments did not lead to functional validation, but the procedures followed and results obtained are described in Supplementary File S5.

A second gene family specific to insects, the Odorant Binding Protein or OBP family, is involved in olfaction by solubilizing semiochemicals once they have entered the aqueous lymph within olfactory sensilla [27]. OBPs were proposed to play an important role in response sensitivity. In Lepidoptera, a dedicated lineage of OBPs (the so-called “pheromone-binding proteins” or PBPs) has evolved high affinity towards pheromone components [59]. We identified 46 contigs assembled into 13 OBP unigenes expressed in our *B. anynana* transcriptome (Supplementary Table S4), a number lower than what has been described in various transcriptomes from moth species (49 predicted OBPs in *S. littoralis* and *Manduca sexta;* [55]) and in the genomes of two butterfly species (32 in *D. plexippus*, 51 in *H. melpomene*; [82,83,84]). *B. anynana* expressed OBPs were found in most subclades of the phylogenetic tree of lepidopteran OBPs, including general odorant binding protein 1 and 2 lineages, as well as classic, minus-C, plus-C and duplex OBP lineages (with categories based on the level of sequence homology and conserved amino acid signatures; Supplementary Figure S6). In Lepidoptera, the OBP gene family also includes a lineage of the PBPs, thought to transport pheromone molecules [59]. In moths, such as *M. sexta* and *B. mori*, trichoid sensilla are associated with pheromone perception and express specifically PBP-A. No *B. anynana* expressed OBP clustered in the pheromone-binding protein-A or -B lineages (Supplementary Figure S6). This is similar to findings in other butterfly species: the PBP-A lineage is lacking in the genome of *D. plexippus* and the PBP-A and PBP-B lineages are also absent from the genomes of *H. melpomene* and *M. cinxia* [59].

In contrast, we did find two candidate PBPs (Supplementary Table S4) expressed in *B. anynana* antennae that belong to the PBP-C and -D lineages present in all butterfly genomes investigated to date [59]. These candidate PBPs indeed correspond to the two sole candidate PBP genes identified in the *B. anynana* genome, and are both most similar to two PBPs found in the antennae of *H. melpomene* [53] (Supplementary Figure S6). In most moths, PBP-C and PBP-D OBPs are expressed in basiconic sensilla and are associated with foraging [59]. Although we cannot exclude that we missed BanOBPs in our transcriptome, the lack of a PBP-A subgene family in *B. anynana,* as in four other butterflies studied (*H. melpomene, D. plexippus, M. cinxia, P. polytes*), suggests that butterflies lost this gene lineage (at least in Nymphalidae to which the sampled species belong), and possibly use other PBP lineages to functionally aid the OR-pheromone connection. The transcriptome was also mined for Chemosensory Proteins (CSPs), a third gene family potentially implicated in olfaction in insects [85,86] (Supplementary Table S5).

### 3.3. B. anynana Sex Pheromone Regulation

**Figure 4:**
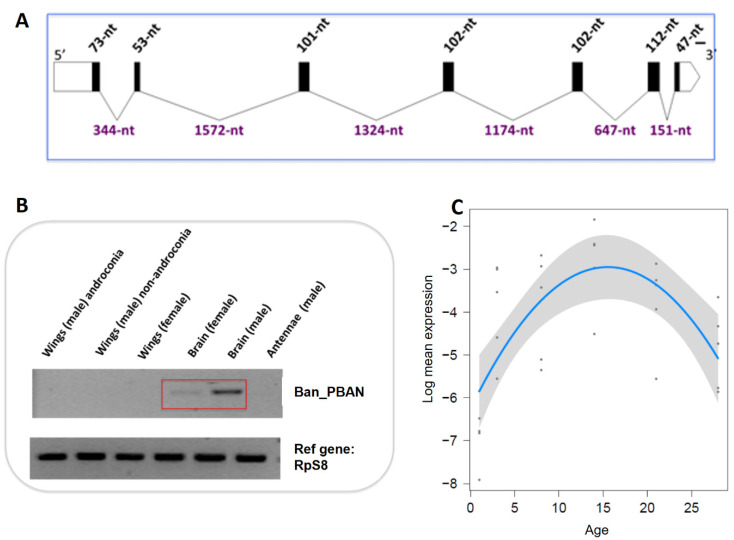
Pheromone biosynthesis activating neuropeptide (PBAN) expression in *B. anynana*. (**A**) Structure of Bany_PBAN full length gene sequence. The 7 exons are represented by black boxes and the 6 introns by lines. (**B**) PBAN expression level quantified by reverse transcriptase qPCR in adult tissues (brains, wings, antennae) of males and females ranging from 3 to 14 days of age. Higher levels of PBAN are observed in male brains compared to the other adult tissues. (**C**) PBAN expression level quantified by real time qPCR in adult male brains from 1 to 28 days of age.

Eleven contigs were found to be highly expressed in male compared to female brains, but their role in the regulation of sex pheromone processing remains open (Supplementary Table S2). Given its role as a key regulator of female sex pheromone biosynthesis in many moth species [87], we focused our attention on Pheromone Biosynthesis Activating Neuropeptide (PBAN). We hypothesized that PBAN could be involved in male sex pheromone regulation in *B. anynana*, and looked for it in our transcriptome database. We identified one unigene annotated as PBAN (BA_PBAN.1.1), which was expressed in adult heads. We used this sequence to obtain the complete cDNA sequence of PBAN in *B. anynana* (RACE), Ban_PBAN (Figure 4A). The phylogenetic reconstruction of PBAN across Lepidoptera shows monophyly of butterfly PBANs, with *B. anynana* full length PBAN encoding the typical five peptides (diapause hormone, α, β, PBAN, and γ), containing the signature FXPRL conserved amino acid sequence. We next investigated the PBAN cDNA tissue distribution using semi-quantitative and quantitative PCR. PBAN was found to be expressed in adult heads, but not in other tissues, and expression was higher in males than in females (Figure 4B). PBAN in male moths is suspected to be involved in male pheromone biosynthesis: the PBAN receptor of the moth *H. armigera* was found expressed in male hairpencils, and PBAN stimulation of the hairpencils was found to be responsible for the production and release of male pheromonal components [88]. Next, using RT-qPCR we found that PBAN expression level in male brains correlated with the amount of male sex pheromone found on male wings during the adult male’s lifetime, with maximum content around 15 days of age (Figure 4C) [41].

**Figure 5:**
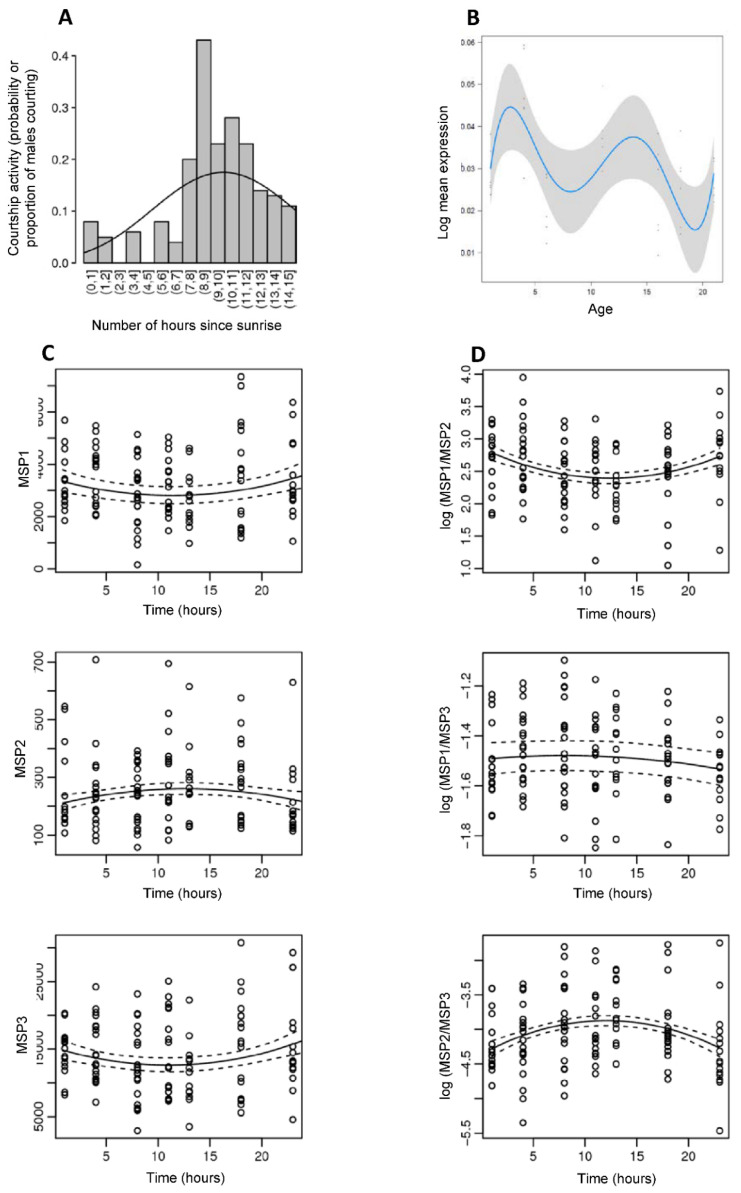
Daily variation in *B. anynana* male courtship activity (**A**), PBAN expression by RT-qPCR (**B**), MSP production (**C**) and log MSP ratio production (**D**). Statistics are provided in Supplementary Tables S9 and S10.

In moths, production of volatile sex pheromones usually shows a circadian pattern that is regulated by PBAN and correlates with the female “calling” behavior (extrusion of the sex pheromone gland) during specific hours of the scotophase [87,89]. A circadian rhythm of male sex pheromone production was also found in the moth *Aphomia sabella* [90]. We tested whether *B. anynana* displayed daily variation in courtship activity, MSP production, and PBAN expression in 8-day old adult males. We found that courtship activity peaked 7 to 12 h after sunrise, and courtship activity was significantly higher in the afternoon compared to the rest of the day (Figure 5A; Supplementary Table S9). Similarly, MSP production significantly varied during the course of the day, and peaked around maximum courtship activity, with MSP1/MSP2 and MSP2/MSP3 ratios displaying significant reversed changes during the day (Figure 5D; Supplementary Table S10). MSP amounts also displayed a slight, but non-significant, variation with time of the day (Figure 5C; Supplementary Table S10). MSP titers were estimated to be minimal around 11 h after sunrise for MSP1 and MSP3, while the MSP2 titer was estimated to be at a maximum 12.4 h after sunrise. We further found that PBAN expression significantly varied throughout the day (Figure 5B; Supplementary Table S9), with the highest expression 11 to 14 h after sunrise. Daily variation in PBAN expression thus correlates both to male courtship activity and to male sex pheromone quantities found on male wings: all three traits peak during the afternoon and PBAN expression is maximal just before the peak in MSP2/MSP1 and MSP2/MSP3 ratios and MSP2 amount. This suggests that the daily regulation of male sex pheromone may be associated to circadian variation in PBAN expression, a neuropeptide that is specific to sex pheromone regulation in moths [91].

In addition to the work described above, we aimed to functionally demonstrate the role of PBAN expression in regulating male sex pheromone biosynthesis in *B. anynana*. These experiments did not lead to functional validation of the role of PBAN, but all procedures followed and results obtained are described in Supplementary File S6.

## 4. Conclusions and Perspectives

Mosaic evolution appears to have taken place at the molecular level based on our investigation of the pathways involved in the production, reception, and regulation of the sex pheromone in *B. anynana*. Our data suggest that the biosynthesis of the three chemical components forming the male sex pheromone (MSP1, 2, and 3) could be partly due to moth-specific genes (*far1* and *far2* for the MSP2 and MSP1 components, respectively) and partly due to genes present in insects other than moths (Δ9-desaturase, aldo-keto reductase for the MSP1 component). This is also likely the case for the MSP3 component whose synthesis is not expected to rely on moth-specific gene families, as this pheromone component is not derived from fatty acids. None of the expressed ORs or OBPs in *B. anynana* belonged to Lepidoptera-specific gene lineages responsible for sex pheromone reception in moths, suggesting that sex pheromone reception in this butterfly may have evolved independently from their moth ancestors. In contrast, we found that sex pheromone biosynthesis could be regulated by the neuropeptide PBAN in both moths and butterflies, an evolutionarily shared derived trait for Lepidoptera. Recently, the genomes of 250 species of skippers (Hesperiidae) [92] and 845 North American butterfly species [93] have been sequenced. A systematic comparative analysis of major gene families involved in moth sex pheromone communication in these ~1100 butterfly genomes would provide important information on the level of conservation of molecular pathways when butterflies diverged from moths about 119 million years ago.

## Data Availability

Data are available at http://ngspipelines.toulouse.inra.fr:9011/.

## Supplementary Materials

The following supporting information can be downloaded at: https://www.mdpi.com/article/10.3390/genes13081372/s1, Figure S1: Tissues used for RNA adult wing libraries.; Figure S2: Contig and read length distribution; Figure S3: RT-qPCR validation of transcriptome data comparing the relative expression level of a subset of candidate genes between treatment and control libraries; Figure S4: Phylogenetic tree of desaturases.; Figure S5: Boxplots showing the spatial distribution of MSP1 (Z9-14:OH) and fold change expression of candidate genes Δ9- and Δ11-desaturase across the *B. anynana* body.; Figure S6: Phylogenetic tree of OBP gene family from a representative set of other moths and the butterflies.; Table S1: Yield of total RNA in µg per library and per life stage; Table S2: List of contigs significantly higher expressed in treatment compared to control library(ies) for olfactory communication in *B. anynana* butterflies; Table S3: List of Odorant Receptor unigenes expressed in *B. anynana*; Table S4: List of odorant binding protein (OBP) contigs and supposed unigenes expressed in the *B. anynana* transcriptome; Table S5: List of chemosensory protein (CSP) contigs and supposed unigenes expressed in the *B. anynana* transcriptome; Table S6: Primers used for the validation of our transcriptomic study using qRT-PCR; Table S7: Overexpression of FAR-1 (fatty acyl reductase 1) in androconial male wing tissues compared to control wing tissues of males and females; Table S8: Overexpression of FAR-2 (fatty acyl reductase 2) in androconial male wing tissues compared to control wing tissues of males and females; Table S9: Daily variation in courtship activity; Table S10: Daily variation in MSP production; File S1: Phylogenetic reconstruction of odorant receptors; File S2: Phylogenetic reconstruction of odorant binding proteins; File S3: Phylogenetic reconstructions for the delta desaturase gene family; File S4: Phylogenetic reconstructions for the reductase gene family; File S5: Functional expression of *B. anynana* odorant receptor candidate genes in transgenic flies; File S6: Manipulation of male sex pheromone production with Ban_PBAN synthetic peptide.

## Abbreviations

MSP, male sex pheromone; OR, odorant receptor; OBP, odorant binding protein; PBAN, pheromone biosynthesis activating neuropeptide.
