# Supplementary material for: Mosaic Evolution of Molecular Pathways for Sex Pheromone Communication in a Butterfly
Source: Genes (Basel). 2022 Jul 31;13(8):1372. doi: 10.3390/genes13081372 (PMC9407440; doi:10.3390/genes13081372)
Supplement: Supplementary file 1 [file Supplementary Figure S4.pdf › Supplementary Figure S4.pdf]

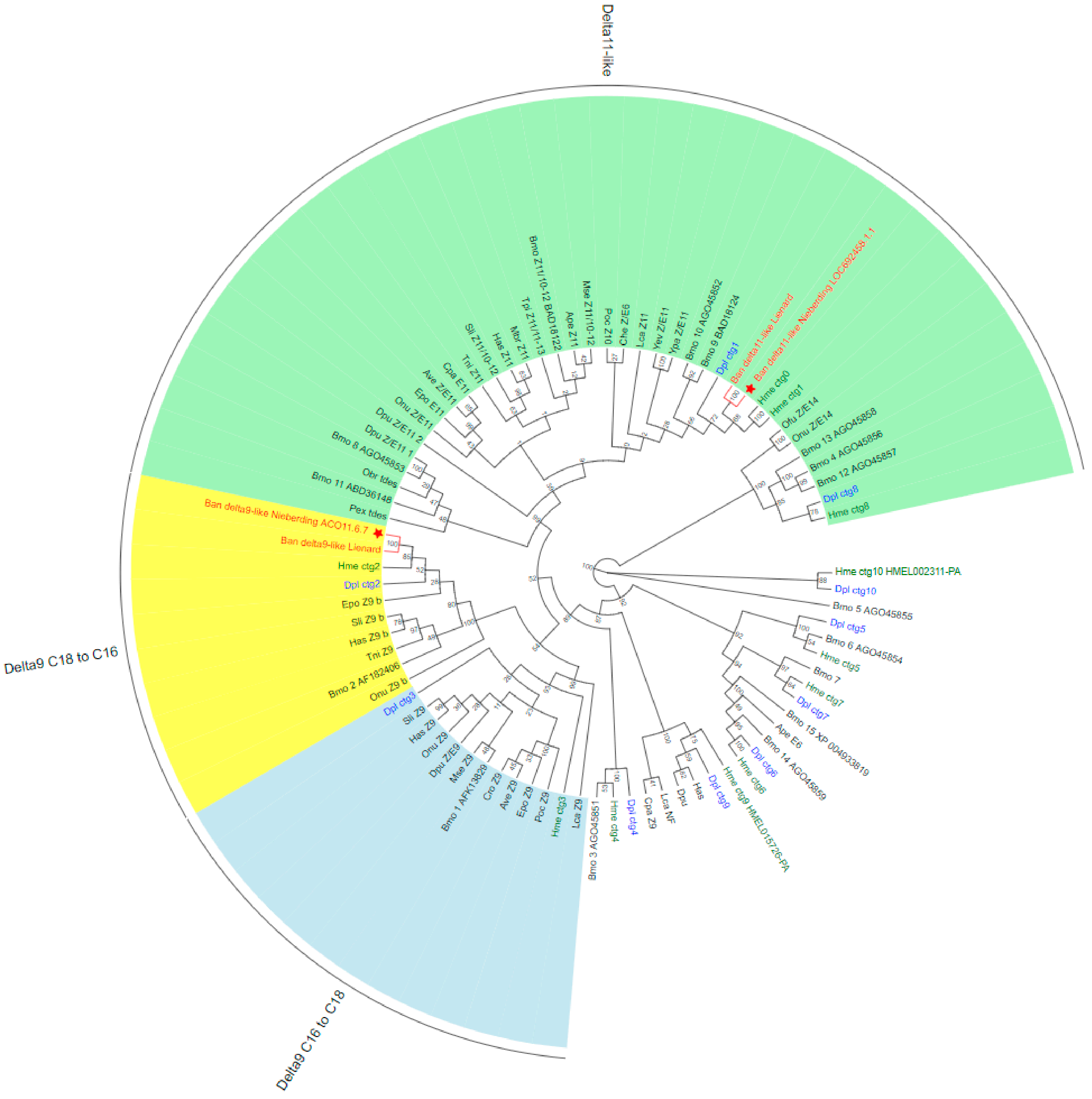

Supplementary Figure S4: Phylogenetic tree of desaturases after [36] (in red), and including our *B. anynana* candidate Δ9 and Δ11 desaturases (Ban delta9-like Nieberding and Ban delta11-like Nieberding, highlighted in red and preceded by a red star), comprising a representative set of moth and butterfly desaturases. Sequence abbreviations correspond to species names as described in [36] : Ape, *Antheraea pernyi*; Ase, *Agrotis segetum*; Ave, *Argyrotaenia velutinana*; Bmo, *Bombyx mori*; Che, *Choristoneura herana*; Cpa, *Choristoneura parrallela*; Dpu, *Dendrolimus punctatus*; Epo, *Epiphyas postvittana*; Has, *Helivocerpa assulta*; Har, *Helicoverpa armigera*, Hvi, *Heliothis virescens*; Hsub, *Helicoverpa subflexa*; Lcap, *Lampronia capitella*; Mbr, *Mamestra brassicae*; Obr, *Operophtera brumata*; Onu, *Ostrinia nubilalis* (Ofu, Ola, Onr.za, Opa, Osc, Oza and Oze correspond to other *Ostrinia* species); Pex, *Planotortrix excessana*; Poc, *Planotortrix octo*; Tni, *Trichoplusia ni*; Tpi, *Thaumetopoea pityocampa*; Yev, *Yponomeuta evonymella* (Yro and Ypa correspond to other *Yponomeuta* species). We used maximum likelihood inference to reconstruct the phylogeny using the standalone version of PhyML14 and the WAG+I+G model as determined by performing model selection in Topali v2.515 as described in [36]. The cladograms were visualized and prepared using the online tool EvolView v2 (https://evolgenius.info/evolview-v2/#login).
Numbers at the nodes indicate bootstrap values for 100 replicates.
