# Supplementary material for: Mosaic Evolution of Molecular Pathways for Sex Pheromone Communication in a Butterfly
Source: Genes (Basel). 2022 Jul 31;13(8):1372. doi: 10.3390/genes13081372 (PMC9407440; doi:10.3390/genes13081372)
Supplement: Supplementary file 1 [file genes-13-01372-s001.zip › Supplementary Figure S1.pdf]

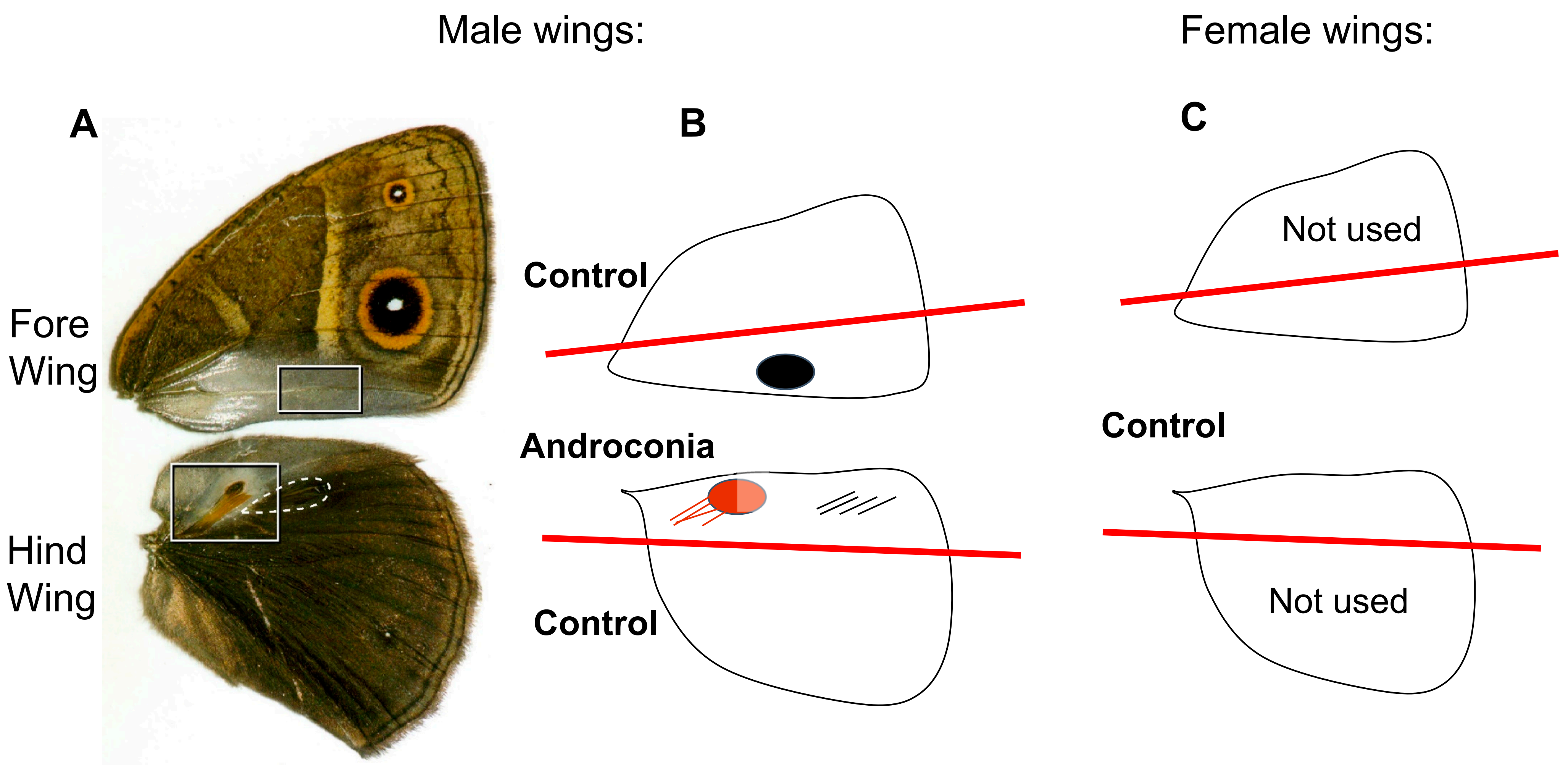

Supplementary Figure S1: Tissues used for RNA adult wing libraries. Adult male wing tissues are shown in A, with white squares highlighting the position of androconia, sex pheromone producing cells and hairs on the fore- (top) and hind- (bottom) wings (as described in [49]). Adult male wings were cut as in B and adult female wings were cut as in C.
