# Supplementary material for: Mosaic Evolution of Molecular Pathways for Sex Pheromone Communication in a Butterfly
Source: Genes (Basel). 2022 Jul 31;13(8):1372. doi: 10.3390/genes13081372 (PMC9407440; doi:10.3390/genes13081372)
Supplement: Supplementary file 1 [file genes-13-01372-s001.zip › Supplementary Figure S2.pdf]

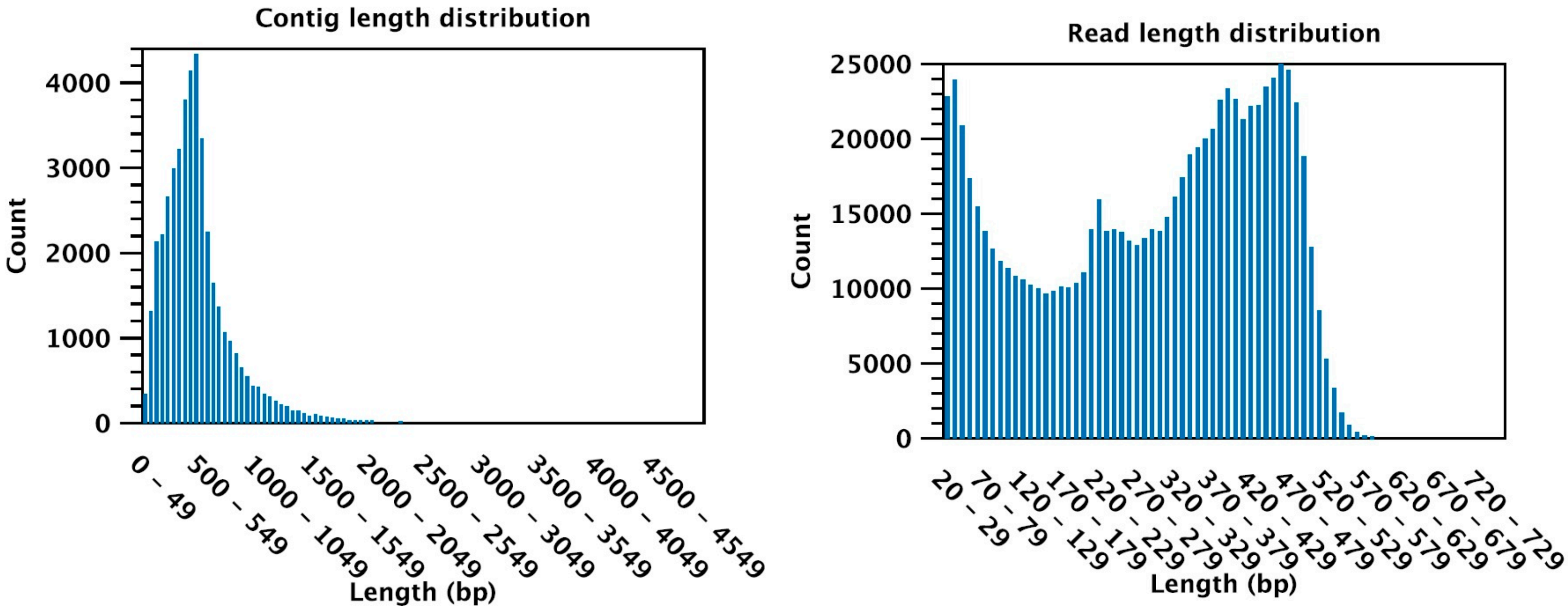

Supplementary Figure S2: Contig and read length distribution with counts on the y-axis and length (in base pairs) on the x-axis.
