# Supplementary material for: Mosaic Evolution of Molecular Pathways for Sex Pheromone Communication in a Butterfly
Source: Genes (Basel). 2022 Jul 31;13(8):1372. doi: 10.3390/genes13081372 (PMC9407440; doi:10.3390/genes13081372)
Supplement: Supplementary file 1 [file genes-13-01372-s001.zip › Supplementary Figure S3.pdf]

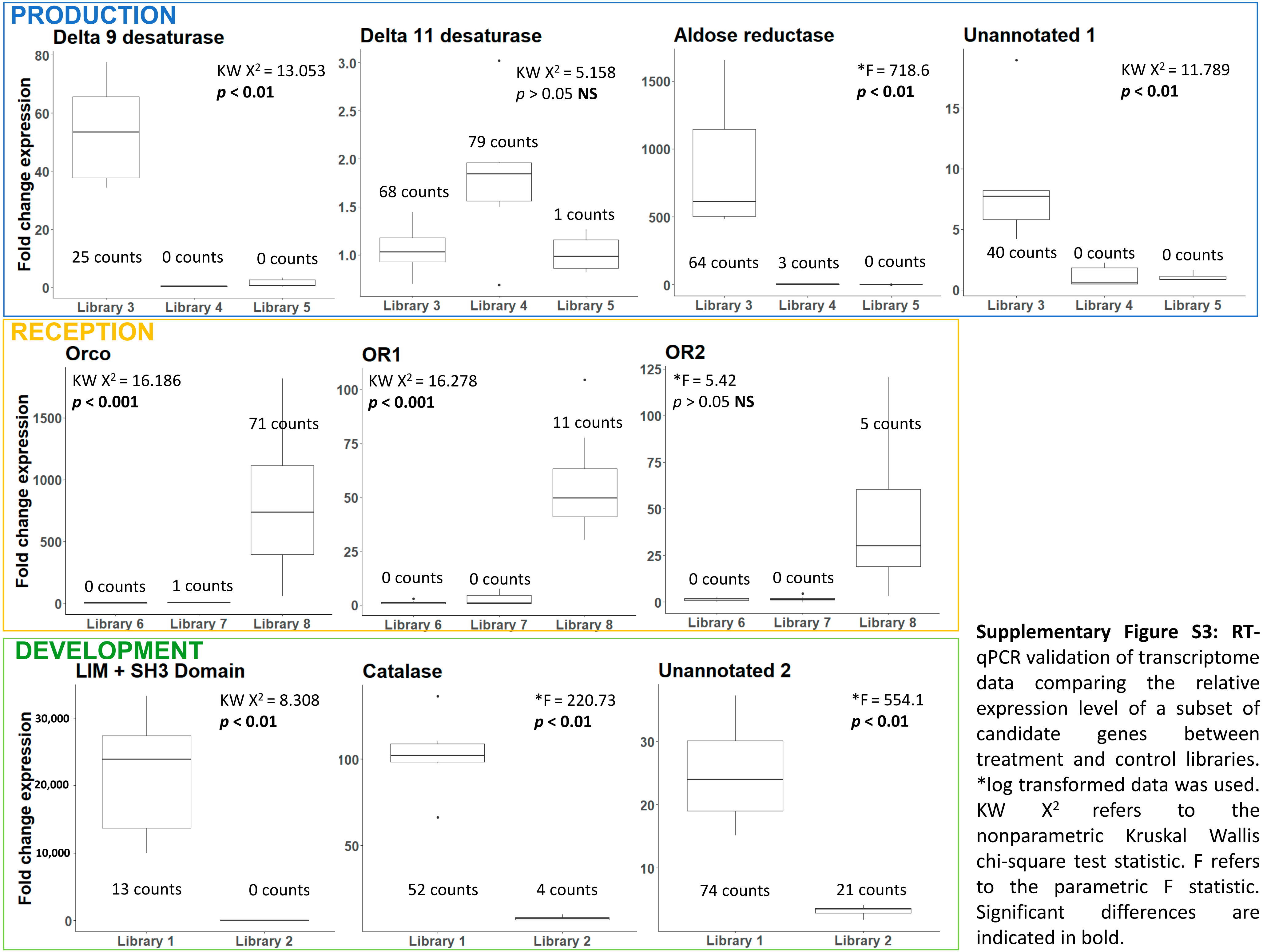

Supplementary Figure S3: RT-qPCR validation of transcriptome data comparing the relative expression level of a subset of candidate genes between treatment and control libraries. *log transformed data was used. KW X^2 refers to the nonparametric Kruskal Wallis chi-square test statistic. F refers to the parametric F statistic. Significant differences are indicated in bold.
