# Supplementary material for: Mosaic Evolution of Molecular Pathways for Sex Pheromone Communication in a Butterfly
Source: Genes (Basel). 2022 Jul 31;13(8):1372. doi: 10.3390/genes13081372 (PMC9407440; doi:10.3390/genes13081372)
Supplement: Supplementary file 1 [file genes-13-01372-s001.zip › Supplementary Figure S5.pdf]

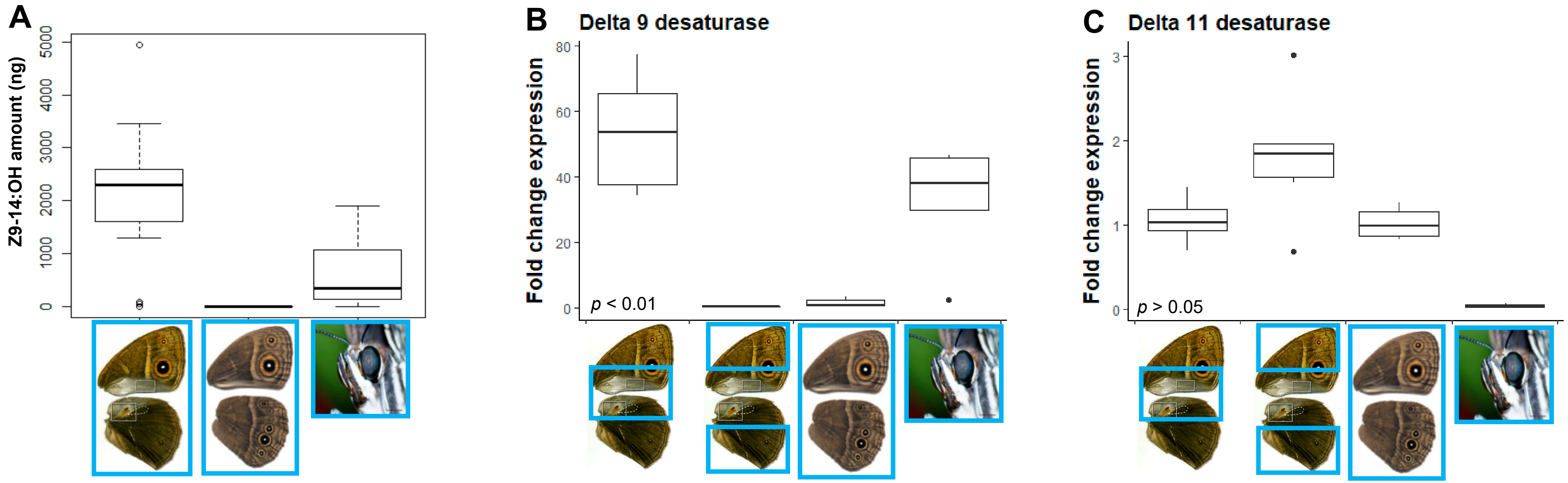

Supplementary Figure S5: Boxplots showing the spatial distribution of MSP1 (Z9-14:OH) and fold change expression of candidate genes Δ9- and Δ11-desaturase across the *B. anynana* body. (A) Amount of MSP1 (in ng/individual) with, from left to right, male wings, female wings and male heads. Data modified after Table S1 in [43]. (B, C) RT-qPCR of Δ9- and Δ11-desaturase genes with, from left to right, male androconial wing tissues, male control wing tissues, female wings and male heads.
