# Supplementary material for: Mosaic Evolution of Molecular Pathways for Sex Pheromone Communication in a Butterfly
Source: Genes (Basel). 2022 Jul 31;13(8):1372. doi: 10.3390/genes13081372 (PMC9407440; doi:10.3390/genes13081372)
Supplement: Supplementary file 1 [file genes-13-01372-s001.zip › Supplementary Figure S6.pdf]

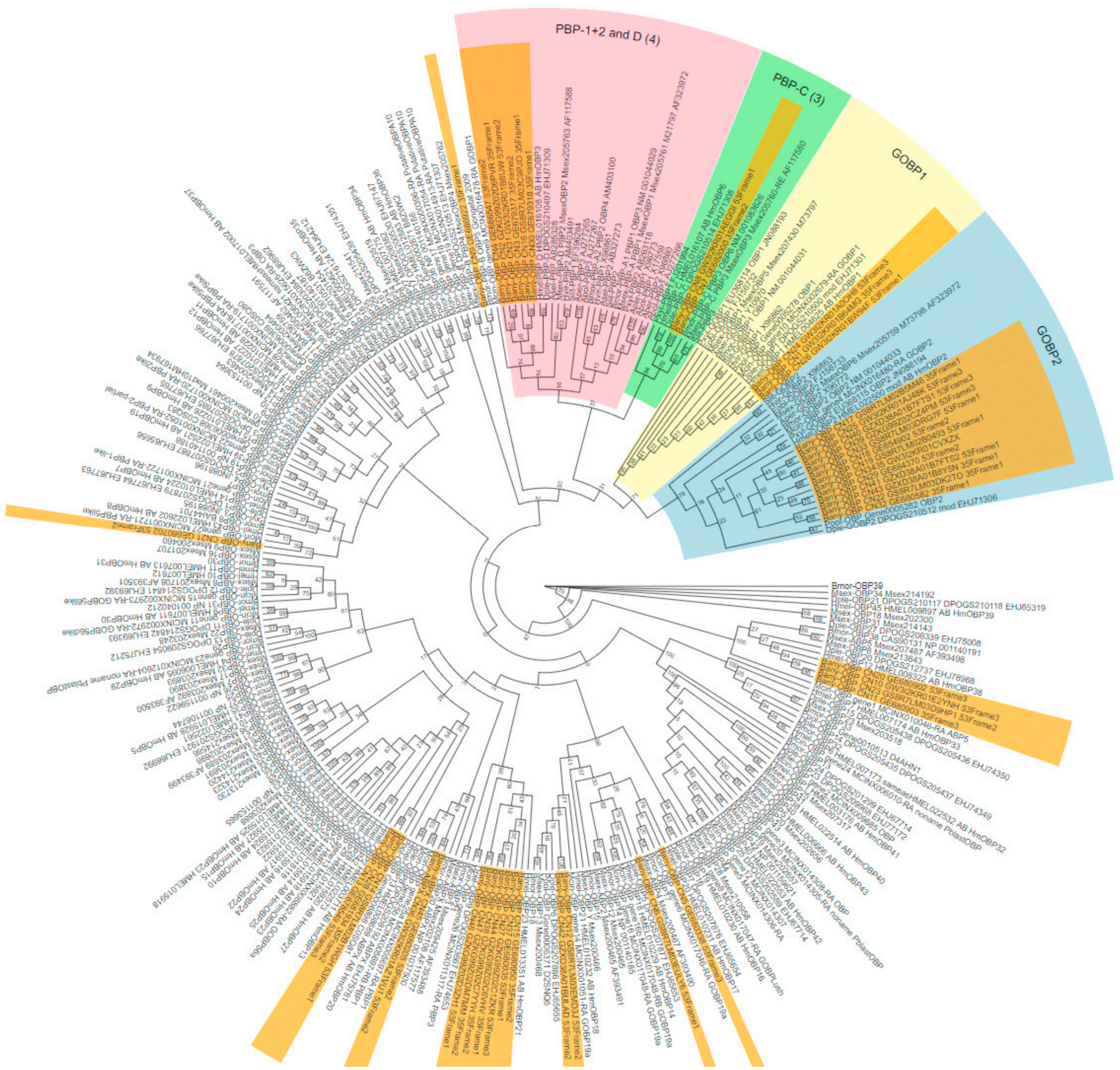

Supplementary Figure S6: Phylogenetic tree of OBP gene family from a representative set of other moths and the butterflies *Heliconius melpomene* (51 OBP), *Melitea cinxia* (32 OBP), *Danaus plexippus* (32 OBP), *Papilio polytes* (9 OBP) and *Bicyclus anynana* (47 contigs).
