# Supplementary material for: Mosaic Evolution of Molecular Pathways for Sex Pheromone Communication in a Butterfly
Source: Genes (Basel). 2022 Jul 31;13(8):1372. doi: 10.3390/genes13081372 (PMC9407440; doi:10.3390/genes13081372)
Supplement: Supplementary file 1 [file genes-13-01372-s001.zip › Supplementary File S1.pdf]

Supplementary File S1: Phylogenetic reconstruction of odorant receptors

We used the alignment of the OR gene family published by [58] (their Figure 3), which is based on the OR gene sequences found in a number of moth species and the butterfly species *Danaus plexippus* and *Heliconius melpomene*. We completed [58]'s OR amino acid fasta file with either all 16 Ban_OR unigenes identified in the transcriptome, or the 7 Ban_OR unigenes most expressed in our transcriptome. We aligned the additional OR sequences with Mafft V7 (website: http://mafft.cbrc.jp/alignment/server/) using G-INS-i reconstruction method, leave gappy regions, gap opening penalty of 3.0 and offset value of 1.0, which gave the same OR tree as in [58]. Phylogenetic reconstructions were carried using maximum likelihood using the "LG+I+G" substitution model and 100 BT. Rate heterogeneity was set at four categories, and the gamma distribution parameter and the proportion of invariable sites were estimated from the data set. Tree reconstruction was performed using PhyML 3.0 [58], with SPR (Subtree Pruning and Regrafting) method for tree topology improvement. Given that the phylogenetic reconstructions using all 16 Bany-OR unigenes positioned all *B. anynana* OR sequences out of the "pheromone receptor subclade" found for moths [58], and that most weakly expressed OR unigenes displayed long branches in the phylogenetic tree, we finalized the phylogenetic reconstructions using the 7 most expressed OR unigenes only. Images were created using Evolview (website: http://www.evolgenius.info/evolview/).

Fasta OR aligned amino acid sequences used for the final phylogenetic reconstruction:

>DpleOR1

-----------------MGLLFADAKDLVGIKKTSDINYMRVLKLGLKVIFCYPHIEGELQSK--
YVILGKIWVTLLSIATLSGQILYLY--HKTGK-IIFLKLGHTYITTAMNSVV----
SFRIMRTFLKSYNEIMNDFMIKIHIFNHKD---RSEYALKIQIIIHKASH-
FVTVFLNSGMIFAIITFNIAPLYNNYRSGAFNDYKNGNASFEHSVFF-YIPEEY-
ENNIIIYTAIFISNWYVSLMCAMLFIQFDLILCLMIINLWGRFKILIHSLETFGESF----
DFETFNNTTQEGFTVNQSTEVNKKLEDIITHHQVIIKFTSKLSSTFGPVLLHYYIFHIVTLCI
LLLECSKM--DPDALTRYGILTFLVVEQLIQVSVAFEILETMSSKLEKAVYYLPWE--
CMNVSQRRVVLIMLMQAQRGGTVKALDMVNVGVQTMLQILRTSFSYFIMLQAL-

>DpleOR2

-----------------MGLLFADAKDLVGIKKTSDINYMRVLKLGLKVIFCYPHIEGELQSK--
YVILGKIWVTLLSIATLSGQILYLY--HKTGK-IIFLKLGHTYITTAMNSVV----
SFRIMRTFLKSYNEIMNDFMIKIHIFNHKD---RSEYALKIQIIIHKASH-
FVTVFLNSGMIFAIITFNIAPLYNNYRSGAFNDYKNGNASFEHSVFF-YIPEEY-
ENNIIIYTAIFISNWYVSLMCAMLFIQFDLILCLMIINLWGRFKILIHSLETFGESF----
DFETFNNTTQEGFTVNQSTEVNKKLEDIITHHQVIIKFTSKLSSTFGPVLLHYYIFHIVTLCI
LLLECSKM--DPDALTRYGILTFLVVEQLIQVSVAFEILETMSSKLEKAVYYLPWE--
CMNVSQRRVVLIMLMQAQRGGTVKALDMVNVGVQTMLQILRTSFSYFIMLQALE

>HmelOR1

------------------------------GIEKTQDIKYMRILKRRLQILHAWPSKAIGEE----
YCLYGRIYVFLVFALSLPPGFLYLKNNLFVID---FMELAHTYVTVSMSTMA----
TFRVMLVFLKIYKNLIADFVNCIHLFNHKD---HSEYAMSTHLLVHKISH-
FFTVYIDILLVVGLLLFNFTPLWKNIYSDAFTNYKTGNSSLELSIYY-QLPFDY-
KHNIYWYLVMFIFDWYMSILCSFCFCYIDLLISLMVFHIWGHMRIIINDMVTFRRPN----
KVTIDANIQDEFFSDEEGRLVARDIGNIVQQHNKIAEFSARLISTFGPILLIYYLFHFVSGCV
LLLVCFQK--NAEAIMLYGPLTFVVFQQIIQISIVFELLATVSDQYSNAVYSLPWE--
CMKVGDRKKVFIMLINSQRPLPVKAMNIVNVGVQTMAAILKTSVSYFIMLNTFA

>HmelOR9

MDLQYMKRIKFYLNWLGSWPFEDLYEVYKEELPLRNSKILIIYFICILLGGIMYVK--
VHKNE-LNFFELGHNYITLLMNVVS--LQRVTMALLNS--YRVMMAHFILNFHLFHHKQ---
RSKYAMKIYKQIDTLCS-
WYTIYLYSQMYVGCILFNLGPLINNIMSNAYSAENGTTPEFEFSVYL-YLPFTV--
QNKKQYGLISLLNILLTSDGACIFCTFDVLMSIIIFHVWGHLKILKYHLESFPKPE----
REPGNRYTKVVMYTEDELPIIFEKLKENIEHHKLIIEFMNLASNGFGVMICVYFIFHQVIGC
ILLLEISAM--DMESFASYGVLTFVMFQQLIQISIIFELIGSKSETIKHAVYDMPWE--
CMDIKNKKIFLFFLNNVQKPIALKALGLVSIGVQTMASILKTSFSYFIMLRT--

>BmorOR1

------------------MLLSFKDDSRSPDIQKPQNFQYMKILRFNLKIICAWPEKQLNEI----
GHSIHRVILPIQSVVCLACGILYIH--FHFNE-IPFFILASTFITVMMNLAT--CSTALVMLFER-
-YLVLTGRFITVMHLFNFQK---NSDYAYKLCTFVNRMSH-
FYTLYVLFSMFMGLGLFNLLPLYNNYVSGAFSDPYGPNVTFFHSVYF-AFPFDY-
SHNFRGYIIMALFNSYVSVTCSIGLVMFDLLMCLMVMHVWGHLKILSHNLINFPRPV----
ITTPNGPTNVETYTEEESKEVFARLRECIKHYGTVDDFANDMSETFGVILLVYYGFHQVS
LCMLLLECSDL--STKAMLRYGPLTLIMIQQLIQISIIFELLGSVADRIPDAVYQLPWE--
CMDVKNRRVVYGFLRRTQNPVRFKAMGMLDVGVQTMASILKTSISYFVMLRTVA

>SlitOR13

------------MDIKLSSVKIFSDGSDLEGIEKVEDILYLRILKKIMWVIDGWPKEAHKS-------
QFFRYYICILDMVSLIPGTLYLK--INTGK-IPSFELGHTYITVFMNAIA----
ALRTVLVLTRNYNEIIFYFLKEVHLFNFRR---KSKYAYETHILVHKISH-
FFTMYVFMLMCCGILLFNLTPIYNSYAAGMFRDEPPPNATFDHAVYF-ALPFDT-
STSFKGYAIVSLYNWYICITCSTYFCIIDLTIFIMVFHLWGHMRVLSYNLENFPKPA----
NDAHAYTACENKYNEEELVEVFIRLRDCIQIHSLVINFSSMMADSFGWTLLVYLFFHQVS
GCLLLLECSQL--DTAALARYGPLTIIIFQQLIQLSVIFELIGSSNDRLIDSVYSVPWE--
YMDTANRKNVFVMLRQSQRSMNLKACSMITVGVQTMIAILKTSFSYFVMLRTVA

>HvirOR13

-------------------MKILSDGSDLEGVEKVEDIFYINLARKSMWILDSWP-KAFNAS------
SKYRYFVLALNVATLIGGAIYLR--NNTGV-LSSFELGHTYITVFMNCIT--CSRCLMILSKD-
-YNHVMTLFVQKIHLFHHKH---KSDYAYLTHIFIHKISH-

FYTVYLLGLALNGLFLFNMIPFYNCYSRGMFRDVIPANATYDHAVFY-SVPFDY-
TTKFKGYLAMTSFNVFISYTCTSYFCVVDLTISLVIFHLWGHMRLLTYHLANFKKPS----
NDNNKDEIKDHSYTEEELKEVFSKLREYIQHHNLILEFSSEMSNAFGPALLAYMVFHQVS
GCILLLECSQL--DTKTLVRYGPLTIVIFQQLIQISVIFELLGSSNDKLIDGVYLVPWE--
YMDTKNRKLVFTMLRQSHRSINLTMMSMVTVGVQTMTAILKTSFSYFVMLKTVA

>BmorOR4

FKIIKNIIVENDALKQVEKPQEFQYMKWVQYHLKYIDGWPNMDMNKKNVSKIRFHKRH
LLVVEQTITFLSQMFYIV--KNYGK-LSFFEIGHSYITALMTIVI--FSRSVVTALGR--
YRKIARYFVSSLHLYHYKD---ISEYALQTHLLVHRLSH-
YYTVYLISLVVTGMLLFNITPLYNNISSGVFNSPRPENMTFQHAVYL-GLPFDY-
TTDIKGYFVVFILNWHLSHIAASYFCTFDLFLSLLILHLWGHLRIILNNLKTFPKP----------
YTNNSMYTEEENQVVLLKLQECIRYHNFIISFTVMMSNVYDVVIIVYYLFHQVTGCLLLL
QCSTL--DWESLSRYGPLTLIIFQQLIQVSMIFEILGFLSDKLPNAVYSIPWE--
AMNVTNRKLVQVLLQKSQKPIQFKAMNMMSVGVQTMASIIKTSISYFIMLRTIA

>BmorOR9

----------------MVARRPLQFHQGRNVDNVEDFKYVKWLRNHLKTVDAWPVHSKSKR----
K-IQKRYVLPIFSAACFISQTVYLK--NGIGT-LSFVVLVHSYICFLINGSC----
LCRGILIATERYKRLATCYLKTVHLFHHKN---RSEHAMKIHVIVHRLSH-
YYTIYLISLVFVGMVLFNFMPIYNNINSGAFKSPRPENVTFQHAMYL-ALPFDY-
TTNIKGYFVVFILNWYISLVTTSHFCTFDLFISLMIIHLWGHIKILMCSLEDIEGF----------
VPGSSFKFTIEQNRKIYLILQECIRHHQFTIDFTNEMSSTFGLVILFYYFFYQVSGCLLLLAC
SQM--DIESLSRFGPMTFILFQQLIQLSIVFELISSLSENLPNAVYNVPWE--
FMDKNNRKMIQVLLLQSQKLIQFKATSMMNVGVQAMATILKTSVSYFIMLRTMY

>SlitOR6

MGLKKFLFEDESVQGITAPTDYLYVKLLRMTLRIIATWPQTELGEKEPVYLNVFLKYFYL
VATIGCQIGSYCYLRTYSDELT---MMEAGHNYIMIMMTFID--ISRITSLTFSTE-
YRKLCKEFFTKMHLFYFKD---ISEHAMETHKRVHLISH-
LFTLWLLFQMLFGVPLFNLVPMYYNYAAGRYKPGGTQNSTFEHSMYY-QYPFDT-
LTEIKGYIVANIINWILSSLCVIWFCMCDLILLLMVFNIWGHLRMLTYTLNNFPRPI----
TIDGGLTVTSAIYSDEENVQIFRKLKECVDYHRRIVEFNSKVSDVFGPMLIIYYLYHQTSG
CLLLLECSQM--TAPVLMRYLPLTIVCTQQLIQLSVIFELIGTQSEKLPSAVYSVPWE--
CMSISNKKFVIFFLMNVKEPIAVKPLGIANVSVTTMASILRTSLSYFTFLRSI-

>SlitOR16

MYIKIVRFMLVIVGSWPRREIGEPEPRYQTIMLKLFFFSVVNAALYGSISYVYMHTSELS--
-FLEIGHMYIVILMTAND--MPRVFTLTLSQK-YRDLAKEFLTKIHLFYFKD---
HSPYAMLTHKKVHLVCH-
LVSLCLLSQMLTGLSLFNLIPMYTNYSSGRYASGGTQNSTFEHSMYF-SYPFNT-

STDFNGYVVACIFHWLLSYFCSTQFVMFDLFLSIMVFHLWGHFKILINSLNNFPKPF----
ELEGGININAEKYSKEELVKVSEKLKECIDYHREIIQFKKAMSDVFGPMLFCYYVFHQTS
GCLLLLECSQM--TAQALIRFLPLTIILTQQLIQLSIIFELVGSESEKLKDAVYGVPWE--
CMDVSNRKVVAFFLMNVQEPVHIKALGVANVGVTSMAAILKTSLSYFTFLRSM-

>HvirOR16

MGLRQFLFENEAVEGINTASDYLYIKILRFTLVIVNSWPRKEIGEPESPRLSAFAKYFYLFL
TVLAAIGSIAYVAVHNRELT---FLETGHMYIVVLMSLVD--VSRVATLTMSTT-
YREVARDFLTKIHLFYYKD---RSKHAMETHRAVHKISH-
LFTLWLVGQMLSGLSLFNLIPMYSNYAAGRYSGDVSKNSTFEHSLYY-SYPFDT-
STDIRGYSIACVIHWVLSYLCSTWFCMFDLFLSLMVFHLWGHFKILINTLNDFPRP---------
SSKVEGAQFSDEELVDVAARLKDCIVYHREITLFTDRMSNVFGPMLFVYYSFHQASGCL
LLLECSQM--TAQALMRYVPLTIILTQQLIQLSVIFELVGSESDKLKHAVYGLPWE--
CMDVKNRRVVLIFLANTQEPVHVKAMGVANVGVTSMAAILKTSMSYFTFLRSM-

>HvirOR6

MNLRKFLFENEAVEGINSPADYLYTRILRFNLDFIRTWPRKELGEPENLAFTVFMQYFYLI
LNIVTVMGSTSYIVVRGSELS---FIEAGLMYLIFLIGIVD--TLTVVCLTFSEK-
FRVLAKDFLTKTHLFYYKD---RSKHAMEIHKKIHLISH-
LFSLWILFQMLSGLSLFNIIPMYSNLAAGKYRKGGLQNSTFEHSLYY-LYPFNT-
STDITGYIIACILHWIISYLCSCWFCIINLFLSLLVFNLWGHFKILISTLNEFPRPS----
SKSVDTQESPYKYTEEELIEVAEKLKDCINYHREIKIFTNRMSDVFGPMLFIYYAFHQASG
CLLLLECSQM--TARALMRYLPLTIIMLQQLIQLSVIFELVGTESEKLKDAVYGVPWD--
CMDTKNRKVVMFFLMNVQEPVHVKAMGLANVGVTTMASILKTSLSYFTFLLSQT

>HvirOR14

MTGIRDFFFNYEAKDGVTNPTEYPYMIMSRHLLTVITCWPKKPKEGLNARAWVIVQKIF
HMSLCLLTTLGMAMYIG--LHKKS-MSFLELGHLYISLLMTVVI--FSRITTLCLNPK-
YRAVSTEFLTKIHLFYYKD---DSEFSMQIHKQVHKISH-
LFTLYLTGQMIAGLSLFNLTPMYNNFSAGKYKKGGLKNSTFEHSLYY-SYPFNA-
SSDVGGYIVSNICDWIISYLCSTWFCTLDLFLSIMVFHVWGHFKILLHDLDHFPRPK----
LDNSNITLTSEKFSSIELGQVSEKLKKCIEYHRKIVSFTDEMSEVFGPMLFVYYGFHQTSG
CLLLLECSQM--TVAALVCYLPLTIMLFQQLIQLSIIFELVGSVSDKLKDAVYSLPWE--
AMDIKNKKTVAIFLMNVQEPVHVKALGLAEVGVTSMTAILKTSMSYFTFLRSK-

>HvirOR15

MTGFRDFVFNYQPKDGITNPVDYPYLIIARYLLTFISMWPKKSVVYHSARAWLWVQKFY
HLLLCAVAFFGGVLYITLHKKSMT---FYELGHLYISLLMMACT--FSRITTLCFNDE-
YRVVAKDFVTKIHLFFYKN---RSDYSMQIHKKVHMISH-
VFTLYLSGQMMLGLFLFNVTPMYNNYSAGKYKSGGLKNSTYEHSLYF-SWPFNA-

STDMRGYIVSNILNWMLSYTCSSWFCVIDFFLSLMVFHIWGHFKILLHDLDHFPRPS----
VIEDSITITNEMYSQTELDQVFDRLGKCIDYHREIVSFTDKMSEVFGPMLFAYYGFHQAS
GCLLLLECSQM--TVAALVRYLPLTIILFQQLIQMSIIFELVGSVTDKLRDAVYGLPWE--
AMDTKNRKTVAFFLMNVQEPVHVKALGLAEVGVTSMTAILKTSMSYFAFLRSM-

>MsexOR1

MIFMDDPLSKSIKDPRDYRYMKLFRSTLRLIGSWPGRDLKEEGATKYEIAPLYWVLVIKI
TCFLLTIIYLI--ENTNK-LGFFEIGHVYITVFMTMIT--LSRSITLSLNPK-
YRRVMTKYITKMHLFYYKD---MSDIALKTHIRVHKLSH-
FFTMYLSTQVVLGTVTFNIVPMYNNYKVGRFENNILVNDSYELSIYF-KTPTKF-
LSTLNGYIAITTFNWYSSYICSNFFCMFDLALSLLIFTVSGHFKILIHNLNNFPLPS----
KVLKTDEIQAPLYNKTEKKDITLRLKQCIDYHREVLEFTQDISEAFGPMLFVYYLFHQVS
GCLLLLECSQM--
DAAALMRYGLLTAVLFQQLIQLSVVVESVGTVTGYLKDAVYNVPWE--
YMDTQDRKTVCIFLMNVQEPVHINALGLAKVGVQAMAGILKTSFSYFAFLRT--

>BmorOR3

MIFVDDAVIGIKDPREYRHLRVLRTSLRLLGAWPGHYLGEETGSKYECAPMFLLMFIKIA
CLYLTIVYLRNNADVLG---FFELGHVYLTIFMTFVT--LSRGFSLTWNPN-
YHKVVKKFITEMHLLYFKD---NSEYAMKTHRRVHKISH-
FYTVFLKVQMIAGLTLFNVIPMYNNYRQGNYASDRPANITYDLSIY--YETFDI-
LNTPNGYIFICVFNWFASYICCSFFCSFDLILSLMISTVSGHFRILIHNLLTFPLPG----
NRSEFVLEEAKLYSPAEMWQVTDRLRQCIDYHRKLVEFTGDISEAFGPMLFVYYLFHQV
SGCLLLLECSQL--NTAALVRYGVLTVVLYQQLIQLSVIVESVGTVTGRLKDAVYEVPWE-
-YMDTSNRKTVAIFLMNVQEPLHVNALGLAKVGVQSMAAILKTSFSYFTFLRTVS

>SlitOR11

MFNLDTYAGDAVVGISGPMDYKYMKVLRFVLRIISGWPGKALGEKTLKIEGMGHAYYN
TILSLIYLALGIAYLKKNAHRFG---FLELGQLYIVLLMNMLS--TSRAFTLCLSEK-
YRTVAKIFIQKVHLFYFKD---KSDYAMSIHVTVHRISY-
FAAIYVSIMLSIAACLFNLIPMYNNYAANFASFDNLQNKTYEQAISC-LYPWNF-
ETNFKGYLVATLSGWYGTVLCGSSVSMFDLFLCLMIFNLWGHFKILIYNLDHFPRPG----
EERSGRVIGSEMYSESELEEVAIKLKDCIQYHMLIVNFTNNMSDAFGMALFIYYSFHQITG
CLLLLECSQM--TAAALSRYLPLTIIMFGELVLLSIIFETIGTMSEKLKDAVYKMPWE--
YMDTKNRRTVLIFLIKVQEPIHVKAGGLVDVGVTTMASILKTSFSYFAFLRTF-

>HvirOR11

MHLAGNAVTGITGPMDYKYMKVLRFVLRIISGWPGKALGEKTLRIEGMGHAYYNTILSL
VYLALGIAYLKKNFHRFD---FLELGQLYIVLLMNMLS--TSRAFTLCLSQK-
YREVAKIFIQKIHLFYFKE---KSDYAMKIHVIVHKISF-

ISAVYLSVLLFIAAVMFNLIPMYNNYSARYSSFDNLENTTYEQAISC-LYPWNF-
ETNFNGYLVATLSGWYGTMLCGSSVSMFDLFLCLMIFNLWGHFKILIHNLEHFPRPG----
AERSGRIIGSEMYSQAELEQVAVLLRECIQYHMLIFDFTNNMSDAFGMALFIYYSFHQITG
CLLLLECSQM--TAAALTRYLPLTIIMFGELVLLSIIFETIGTMSEKLKDAVYKVPWE--
YMDTKNRRTLLIFL--

>CpomOR4

MSLKSRVWEKLKKKFDDGEVDSPLKYTYVKQVSCLMSSVGAWPHRQFGRQKLHTLLSI
YNLGLHGVCAGMFILGLLYWR--QRRHN-MSFFDSGHIFLCMLFDLLV----
LLRFMVTQTTKYQETIKAFLLEFHLFYFKD---RSPYAAKVHTQVHTISG-
MFSFYVICQMAHGMALFVLMPCYSNLRKGMFGKNRPENSTFENSAYY-YLP-
DACYTTLRGYWILLAFNAFSSYIITIGLFEFDLMISMMVFQIWGHLKILKNSLLTMPLP-----
-----
VNSKDGLYSPEENIKIKALLKEIIEHHTLIIKFVDGCSNTFSEYLFTFYLFMQFITCILLLEVS
SF--TADALGKYGPLTIGMHQQLIQVSILFEVLNTKSNELIDAMTQIPWE--
HMNTSNRRTVLFLICRIQIPVSLKAGGMVPVGVNTMQAVLKGSVTYYMMLKAFA

>BmorOR7

------------------MLLYHPNTQVEEKVNNVEEFTYMKFLKSFCKIMDFWPEREEKNS----
KTRIFRLRYILVLQFCFTLVAGVLYLKNNFGK-KTFYDLGHTIITVVMNVVS--
VSRLILRCFKK--YDVVGQQFINKIHLYHFRN---DSEYSMKTYKAVHKISN-
NMTYIFSFSIFVCVVTFNLNPVFNNIGSGAYKNPRPDNVTLQQCVYY-ALPFDY-
TGDFKWYMLVAIFNVQKTFFCTSLFILFDLLLSMMIIHLWGHIRIFIHNLNHIPAP-----------
RNSLEYTREERQEVDNTLKKCIQHHTLIIGFVRIMSETYGLAVLIYYAFQQVVGCLLLLQ
CSRL--DLKTITRFGFLTTMVNQQLIQISVIFELLGYMNDKLQEAVYCVPWE--
YMDTSHRKMVYMMFRQSQIPLQLKAMNMLSIGVKTMASILKTSVTYYLMLKTIT

>BmorOR5

------------------MLLYYPNTQVKEKVNNVEEFTYMKFLKSFCKIMDFWPEREEKNS----
KTRIFRLRYILVLQFCFTLVAGVLYLKNSVGK-QTFYDLGHTIITVLMNVVS----
SRLILRCFKK-YDVVGQQFINKIHLYHYRN---DSEYAMKIHTVVHKISH-
NMTYIFSFCIIFGTVTFNLTPIFNNIGSGAYKNPRPDNVTLQQCVYY-ALPFDY-
TGNFKWYLLVAIFNVQKTFFCTSLFILFELSLSLMIICLWGHLRIFIHNLNHIPAP-----------
RNSFEYTKEERQEVDDTLKKCIQHHTLIIGFVRIMSETYGLAVLIYYAFQQVVGCLLLLQ
CSQM--ELKTVTRFGFLTLVLNQQLIQISVIFELLGYMSDKLQDAVYCVPWE--
YMDTCHRKMVYMMFRQSQIPLQLKAMNMLSIGVKTMVSILKTSVTYYLILKT--

>MsexOR4

-------------------MKFFVDGSEIAHITKPQDIQYMQMLKFFTNSLAGWPIEAVEGI----
KNFYWRNGLVVIAYAYFFGQVFYIY--RYIND-YTFLVMGHSYITVLMTIVT--
IARHTLPYFKC--YDDTTADFVHNIHLFNYRN---KPGYYKEFHLKIHKISH-
AFSVYLCTLLVTGPSMFNGIPLYNNYASGAFSFNRSPNVTYEQAVSL-LLPFDD-
TNNFKGYFVVFLANCCVSYISSCCLCIYDLLLSLMVFHLWGHLKILTKTLDNFPKPP----

QAIEADPNKSLKFSDEELKVIHKKLGECVAHHQLISNFSTRMSNTFGLSLFIYYGFHQLSG
CLLLLECAQL--EAAAIICYGPLTLVVFQQLIQLSFIFELIGTVNEGLTDSVYCLPWE--
AMDQGNKKIVFTFLRQSQKSMNLKALNMLSIGVQTMAKILKTTMTYFLMLQTIA

>HmelOR10

-----------------------------KEISSPMDLRYMKRIKFHLHFMGGWPLNELYDAYREN--
RHFKFLLVF-WSTIFINSLFYLK--FNLNK-LDFFKLGQVYIVIFMTAVA--LS-----------
LLPVFITILN-----------------SQVHKEVHKVLE-
YLTTWILIELYVGTSSFHLIPLYNNIRSTVNDRNVNNGTIYEYAMYF-YIPYEQ---
NNIIFIFIFVISLILIFDIGSAFCTYDLLMSTIIIHVWGHLKILIHHLENFPRPE----
ADKYGNKENDFRYNKKESRVIFDMLKENIEHHKLIMGFMNSVSIAFGVMMFVYYIFHQI
TGCILLLEISAL--NAEAFVTYGLLTAFMYQQLIQTSFIFELIGSKSEVIKEAVYGLPWE--
CMDVKNKKIVLFFLYNVQAPIRLNAMGLIAVGVQTATAV---SIK---------

>HmelOR11

---------------MFNSNSIIHFLFRNEEISSPMDIKYMKIIKRHLHIMGGWPFNELYEEPKY-
IPLRNYKFLVVFFTAILLNCLYYLKLHIKRLD---FFKLGQIYIVLFMNVVG----
LVSFFFSINNEVNKILK----------------------------------
YFTTWILLELFTGIAAFHVIPLYNNIQSSASDHSLENGTLYEHAIYH-YMPFEE---
NNIIYAIIFILNLMLTFDGASSFCIYDLLMSIILIHVWGHFKILKHHLENLPRPK----
AERSEGKANGIMYSKKESQKIFNMLKENIEHHKLIMEFMDSVLSAFGIMMCVYYLFHQV
SGCILLLEMSAL--NTEAFVSYSLMTLVMFQQLIQTSVVFELIGSKSEVLKDAVYGVPWE--
CMDEKNRKIVLFFLYNVQTPISLRAMGLVTVGVQTAAT----------------

>MsexOR15

---MLYIY--TRWNN-
IAFSTHGTIFTIVPITFLI----LTKVLSAQKESYRRLMKTFLQEIHLCNFG----
SNSYMKQRAVEVEKYSR-YLATFFFVFLVVYRALWTIVPIVFNLKN--
SKAIENKEIPLKTCFYM-WLPFDY-
EHEYKYWVITHIANSTLIALGCIVVTSYDTINYSIVFHLIGHIKSLKHLIKTNISQ--------------
-----NLSDDETKRGLVEVIRYHCFVLKIFGEIERAFGINVTGNYLYNLIADSLLLYHLMLG--
DKENKLMYGVMLLVFMGGLIVMTLILEEVRRQTFDIPQTVYDMPWE--
KMSVSNQKIVVIMLARTQPTLEYKSAGGLKAGVNPTIQIIKSTFSYYVMLKSSL

>HmelOR5

---------------------------QPPLNQMFYRIIANMMSISTVGNRTNWGYKTYPK-----
MYVIMNIVAIIFAPTVIVSQVAYLCVNFTKLD---FEILIIAFSILPSTVLA----
NVDIFSSKTKTNISLSRNLLSKIHLYNIYKNDTNDEFKLIFVEKSIRISS----
YYLICFCGTNWLSWVTMPVYNNYHD--KEAVLNHTVQLQTCIFL-WLPYDY-
RYDFNNWIIVHTINSYIIFAGASAIMTYQVIFYTLTYNLIGHIEILRNKIKTEFKE--------------
-----DLTDDQAHSKLVEIIKYHDFICQVFEDLQTAFGVNVAANYLENLVSNSILLFQIMYG--
GKEKIFQYLAMGMAYTGGPIIISIVIDELRRRTYNLSELLYSIPWE--
DMSVPNQKIVLLVLQKMQIVMDFKAFGGISAGVAPMISVSTIKYNESLCLFGMC

>HmelOR7

-------------------MDLFFLRQPPLNQMFYRVIANMMSISTVGNRTNWGYKTYPK-----
IYVLINIVAIIFAPTVIVSQVAYITINFTKLD---FEILIIAFSVLPSTIVA----
NVDIFCSKTKTYLNLSRDLLSKIHLYNIYKYNKNDEFIKKKLIFTEKSTR-
ISSYYFIGFCLTNWLSWITMPIFNNYRN--KEAILNHTVQLQTCVYL-WLPCDY-
RYDFNNWIIVHTMNSYVIFAGASAIMIYQAIFYTFTYNLIAHIEILKEKINTEFKE------------
------
DLTDHQVHAKLVEIIKYHNFIWQVFEDLQTAFGINVAANYLQSLVSNSILLFQVMYG--
GKENLVQYITMVMAYAGGPIILSIVIEELRRRTYDLSDLLYAIPWQ--
NMSVPNQKIVLLVLQKMQIVMDFKAFGGISAGVAPMISVSISIKKYYESLCFSG

>HmelOR6

--------------------------QPPFNQKFYRFIANMMSITTVGNRTNWGYKTYPK-----
IYVFINIVAIIFAPAVMVSEIAYLCVNFTKLD---FEILIIAFSIFPYIFSS----------
KTETYIRLSRSLLSKIHIYNIYKYN-KNEFMKKKLIFVEKSIR-
ISSYYLICFYTSNWVSWILMPIYNNYKN--KEAVLNHTVQLETCVYL-WLPYDY-
RYDFNVWIFVHTINTYIIFAGAYAIMIYQIIFYILTYNLIGHIEIFKNKLKTEFNE--------------
----DLTDEQVHFRLVEIIKYHNFTV--FEDLQTTFGLNVAANYLENLVGNGLLLFQLKYG--
GKDKLLQYIAMGMTYTGGPIIMSIVIEDLRRRTLNLSELLYAIPWE--
NMSIPNQKIVLLVLQKMQIVMDFKAFGGISAGVAPMISV-SI-----------

>BmorOR6

-------------------MKEEYYLQHPRTQLFYKVLAHVSTIESTIDLTWWGYTFPKYVGW--
FYHLQCNVVRLFGKCVVVSQILFIILNYQTID---KSVFIIAITITPLGALV----
GIKAESAKAECYVNLMKNFMDKVHIHSIYRKNENNEFVKKKVIQIERVSR-
FTAYFLVILIAINCLSWMLKPTLHNIKH---FEEIMNSMEFQYYIYF-WTPLDY-
KYNLRDYIIIHTLCIYLGATAVTVIVTFDIFNFIAVFHVVAHIQILKNNVKSNWSD-----------
-------DFNESEKKGYLVSILEYHAYIIRIFGEVQSAFGLNVASNYLQNLIEDGLFLYQIMNG-
-EKENVLMYGLMIILYLGGLIFLSIVLEEIRRQNYDLCEYVVYALPWE--
GMSLENQKIFVVFLQRTQPDLEFETVCGMKAGVKPAFSIVKSMFSYYVMINSRF

>DpleOR22

---------------------------MPWNVEVLLRTLTIRSYRRLNKPISWI----------
LTTLSGLFYIYSYVISPVWFLMVKS--RETGD---FTAAIVELSLSMCSLTC--
VPKIFYMKYYSSTVMDLIDEYLR------CNELIVKGTRFEKNFLKNMKTVK-
KQATVTWAVSMLNSATYLTVPLLRPGRH-----------FTQDMYV-IYGLEP-
MLESPNFEIATFMMVLSIIFALKVIVYITLFVIVIIGFLESQMLALSVELTNIWEDY----
NYYILQHNESGDEYEVRNVFVTNRLRDILKFHIMNINLRNKVEKELRFIFVIDFIFMMLGL
VTELLGLDNT-------LFQFSFT---LGIVFIDCCIGQRLIDASIAFETAVYDCKWE--
NFNLSNQKTVLFMLQCSQKTMTLTAAGIAVLNFIFLMSVLKSTYSAYTTLASFT

>DpleOR20

----------------MMWNVEVLLRTLTIRSNRRFDKPIPWIYYVIN--------------------
ISTGLCYFYTYMLSTAWFVLVRC--RETGD---FISAMVALSLTMCSITC--
IPKIFYMKYYQNTVMDLVDNYLH------CNELIVSGTRFEKNFFANMRTVK-
KQATIAWVILTLNGVVFVTLPFLRPGRH------------FSQDLFA-LYGLDP-
MVESPNYEKASILLTLAVYFSLTVMVHITLFIIVIIGFLESQMLALSVELSNIWEDY----
NYYVLHHNESITKYELRNLFIKNRLRDIVKFQIMIVSLRNKVEKELRFIFVNDFIFMIFALV
SELVGLENT-------MVQMPFT---FGMVFIDCFIGQRLIDASKAFERAAYGSKWE--
NFNLSNQKTVLFMLQCSQKTMTLTAGGIAVLDFIYLMSVLKSTYSAYTTLKSSA

>DpleOR19

--MFVCEMVARSGLLG--
LFDED-LVYFDAPHYSPATLFPSQL--EAGMTLKVYRLEAVMDLVDEYLN------
CNELIVKGTRFEKNFFTNMRIIK-KQATVSWFVLTLNSAIFASLPFMRPGRH-------------
LTQDL-YVMY---
MMESPNYEIAAILTVSSVYFAVTIIIHLTLFIIVIIGFLESQMLALSVELTNIWEDY----
NYYILQHNESGDEYEVRNVFVTNRLRDILKFHIMNINLRNKVEKELRFIFVIDFIFMMLGL
VTELLGLDNT-------LFQFSFT---LGIVFIDCCIGQRLIDASIAFETAVYDCKWE--
NFNLSNQKTVLFMLQCSQKTMTLTAAGIAVLNFIFLMSVLKSTYSAYTTLAS--

>DpleOR35giEHJ75373.1|

----------------MMWNVEVLLRTLTIRSNRRFDKPIPWIYYVIN--------------------
ISTGLCYFYTYMLSTAWFVLVRC--RETGD---FISAMVALSLTMCSITC--
IPKIFYMKYYQNTVMDLVDNYLH------CNELIVSGTRFEKNFFANMRTVK-
KQATIAWVILTLNGVVFVTLPFLRPGRH------------FSQDLFA-LYGLDP-
MVESPNYEKASILLTLAVYFSLTVMVHITLFIIVIIGFLESQMLALSVELSNIWEDSKKFYN
YYVLHHNESITKYELRNLFIKNRLRDIVKFQIMIVSLRNKVEKELRFIFVNDFIFMIFALVS
ELVGGLEN------TMVQMPFT---FGMVFIDCFIGQRLIDASKAFERAAYGSKWE--
NFNLSNQKTVLFMLQCSQKTMTLTAGGIAVLDFIYLMSVLKSTYSAYTTLKSSA

>MsexOR50giAFL70813.1|

WTGIRKFGLEYCDLPTMLWNVAFLLRPLTLNIDSRHKKPIPLLFYVLT--------------------
VVITSSYFYVYLISMSWFVFVRS--LETKE---LIPAMVVLSLGISSEIG--
TLKFFYTFVYIDKVRKIVDEYLE------CDALVVPFSRFSKNLLTTLRFVK-
KRAIIYWLVIIGNGFAYWSKPLFMKGRH-----------HLEDNLV-IYGLEP-
MLESPKYEIAYFLMTAGVCFICYPPANVTILLIVVVGYTEAQMLALSEEILNIWDDANDH
YNNLPSTNQNTN---
ELKSKIINKYIKDRLKDITKSHARNINLLRQVEFVFRGAIAIGYVFLILGLIAELLG--
GLENTFLQIPFA---FIQVAIDCFTGQRVMDANIVFETAVYDCKWE--
NFDKTNMKTVLLLLQNSQKTLTLSAGGXTMLNFKCLMSVIKSIYSAYTTLRT--

>Bany_OR2_GZKG99Z02DT2MR

---AMAAA--
VSLTSCNVTC--ITKFLFMIIHVTHIRDITEKYVK------FDSQFEPESRFAVDLRTHLRVVK-
SRALYIWTVLVVNCIVFVLIPLLRPGRH-----------VTEEMYI-LYGLEP-

MLESPNYEVVHTVSAISVGFCVYIMVNVAVYVIVIIGYNESQMYTLSLEVRSLWDDSQN
FYNNIKHKINNKVYGSYIKDTMVNEYIRIRLKQIVGYHVANIHLFHALNNEFLIILAIEYTL
LSIAIIGELLG--GLENTYLQVPYT---VVQIYMDCLAGQRLIDACNEFEFAVYDCQWE--
NFNASNQKTVLLMLVLSQKTLMLSAGGMANLNYECLMKILRSAYSAYTALKSSL

>DpleOR21

----------------------------MTVSKSPVSGY--------------------------------------LNKMYQYTENIF----
PETGD---IRRAAVELSLATASGTC--VAQFLYMKLCSNRLMDIVDEYLS------
CDKLVSRGTRFEKNLSWKLREIK-KRALVVWIVLIGNAALYVTVPIFIPGRH-----------
FTEDLFV-LYGLDP-
MVESPNYEKASILLTLAVYFSLTVMVHITLFIIVIIGFLESQMLALSVELSNIWEDY----
NYYVLHHNESITKYELRNLFIKNRLRDIVKFQIMIVSLRNKVEKELRFIFVNDFIFMIFALV
SELVGLENT-------MIQMPFT---FGMVFIDCFIGQRLIDASKAFERAAYGSKWE--
NFNLSNQKTKYEVLGNNTEDLHLVCYRNVQNSRKNSNEDDGE-----------

>DpleOR28

-------------------------------------MNVLRKLRKFGL--------------------DYYDMQMML----------------
WN------IEVLFRAFTINIRKETG-----------QRNSIRDLVEKCLQ------
CNKLIVKGTRFETNFLASLKDVK-RKAIMLWSGFAINGVVYILAPFLKTGRH-----------
FPQDLIV-IYGLEP-
MYESSNYEIAMMLFMASSYFSLFIITYVSLLMIVIVGFVESQMLALSEELENLWDDY----
NDYKFDEESERKENVIRNLFIKMRLSDIVKFHIMNINLRKEIKDKMLFIFFNDFIFMAFAM
VAELVGLQNT-------AVQIPFT---FGMVCFDCYVGQRLLDAAATFEEAVYSCQWG--
NFNVSNRKTVFLMLLCSGRAMTLSAGGMSVLDYSFLMAVLRSTYSAYTTLKSTE

>DpleOR16

--ISWIFYFLI--------------------
FSSCACYTSAYIFNAIWFVFVRC--VETND---FITAMVALSLTTCSASC--
VIKFFYMKLFKNQLMGLVDEYLY------SDTLVERSTRFASNRLQNLRLVK-
RRAIIVWTILISNAIIYVIEPLLRPGRH------------FTADLYV-IYGLEP-
MFESPNYEIIYVIEAASIYFSVWTTVHLTLLIFVVLGCIEAQLLALSEELNNIWED-----
SYKYYKQNENRNEYFIRNTFLRRRLRDIVRFHIVNIHLRRIVDKVTRFIFGVDCAFMILAIV
TELVGLENT-------FVQFPFT---IGIVFIECFIGQKLIDASIVFEKSVYSCKWQ--
NFDVSNQKTVFCMLQTSQNTLSLSAGGVAVLNLAFFMFVLRTTYSAYTTLKSS-

>DpleOR17

MGASNSAIERRTHGYCCLHAARGAPNLAKSYRALTTKPRYFILKSTFTSSCACYTSAYIF
NAIWFVFVRCVETN--DFITAMVALSLTTCSASC--VIKFFYMKLFKNQLMGLVDEYLY----
--SDTLVERSTRFANNRLQNLRLVK-RRAIIVWTILISNAIIYVIEPLLRPGRH-----------
FTADLYV-IYM-----
FESPNYEIIYVIEAASIYFSVWTTVHLTLLIFVVLGCIEAQLLALSEELNNIWED--------
SYKYYKQNENRNERNTFLRRRLRDIVRFHIVNIHLRRIVDKVTRFIFGVDCAFMILAIVTE

LVGLENT-------FVQFPFT---IGIVFIECFIGQKLIDASIVFEKSVYSCKWQ--
NFDVSNQKTVFCMLQTSQNTLSLSAGGVAVLNLAFFMFVLRTTYSAYTTLKSS-

>DpleOR18

------------------------------NDTQPVSWALYLII-----------------------
TICSSCYFFAYVVNLVWFVLVRC--VETGD---FITAMVALSLTFCSGTC--
LAKFLYMKMYTNTVMNLVDEYRL------CDKLLVSYTRFAMNRMNNLRIIK-
KRAITVWLVLVTNAVIYLLTPLLSPGRH------------FAEDLYV-LYGLEP-
SFETPNYQIIFAIEAASIFFSISTTVHITVFIIVIVGCIEAQLLALSEELENMWED-----------------
----SAKFYDRK---------TVMKLID------------DYLHCNLVVRGTRFE------------------
TNTIAIVFIDCFIGQKLIDASDVFESSIYGCRWE--
NFDVHNQKTVLFMLKNSQKTLYLSAGGIAVLNMSCLMCIFRTTYSAYTTLQ---

>HmelOR40

------------------MGIFGNLGLDHSDLRTLIENIGILFRGLTINIDQNNIRPIPWIIYI--
FNVVAGTFYIYVYILSMIWFVLFRC--PECGD---ITICFLEVSLGMCSASA----
IIKFFYIKLTTVRKLIYEYIK------YDRLVLPETRFADNIKKHLRIVK-
RRALIIWITFMVNGTIYIIMPFLMPGRH------------LTEDLYV-IYGLEP-
MLESPNYEIATMLMIPAIIIGVFSLAVIVAFMVVIVGYIEAQMLALSEELLNLRQDF----
CEKVTRQNNSLSTIAIQNIFIKNRLRDIIRFHIMNIKLRNNVENKFRIIYIIEFIFLFFAIVAELL
GLENT-------YVQLPYT---FDQIFLDCLIGQRLLDASIVFEKALYSCEWE--
NFNVANQKTVLLMLRNSQKTLTLSAGGIAVLSYPFLMHVIKSTYSAYTTLQSTV

>HmelOR35

------MDRPALSSRPSVWTVVRKFELDHDDFTTIMENIHFMLRILTINIFNNSNSIPVISY------
IFVIIGSFAYFYVYILSIIWMAY--FYYGN---WTAMAIVLSLFACSITS--
FTKLIFMKLNSKNVKNLIERFLQ------CHAQVVPGTRFCKNLQKRLKIVQ-
QRAIIIWVFGMLNGAIYVLLPFIIPGRH------------FTTDLYV-IYGLVP-
MFESPNFEIATTLNITSIAFGIYALVSIAAYILIVVGYIESQLHALSEELSTIWEDY----
NVSKNNCDGIGEREDIINQFIEVRLRDILQLHIANINLLHELDVELRMSLILEFSLITFAITAE
LLGFKNT-------YLQLSYT---ILQVYMNCLAGQRLIDAGKCFEHSLYSCKWE--
NFNVKNQKTVLIMLRMAQKTLVLSAGGIAKLDFACLMEILKSTYSAYTTLNS--

>HmelOR36

------MIKFSFCSH--IWKRLRKFGLDYDDFPVILENVNSLLRNITIDIFKNSKPISFIFY----
LTVIGSSLYFYVYVFSMVWLVCFNY--GRAGN---WTAVAVVLSLFSCSITS--
FTKLVFMKINSNDIKEIIAKFLQ------CHAQVIPGTRFANNIRKRLRIVK-
RRATNIWLFGVINGGIYVIIPFLTTGRH------------FTTDIYF-IYGLVP-
MFESPNFEIATVVNITGIGFGIYTQVTAAAYVLVTVGYIESQMHALSEELQVVWDDK----
HNLRDKVNAVDNKRRLMNIFIRVRLRDITKLHIANINLLHELDKELRATLALEFTFIAFSIT
AELLGLENT-------HLQLPYT---VLQIFMDCLAGQRLIDASNNFEISLYNCKWE--
NFSIKNRKTVLLMLRVSQRTLVLSAGGVAKLGFPCMMMILRTAYSAYTTLKS--

>HmelOR38

----------MRNKERGIWGTLKTFGLRHDDFPTIMNNVAILLRALTINIYASAKPISLILSI---
LTLIVSFAYIYECVISMVWLVFFYY--GRTGQ---WDGICVALTLFSCTVTC--
VTKFVYMKIFSKDVQKTVEKFHK------CDAQVIKNTRFARNLRKRLRTVK-
KRATLIWITLVVNGGIFMMIPYVTPGRH------------FSLDLYY-MYGLMP-
VLESPNYEIATFINTLSSIFCSYTMVCVAIYVIVIVGYTEAQMYALGEELINVWNDK----
HKVQDKIHIIDIKDKISNDFIKIRLRDIIKFHIINIKLVQDLEEELRSTFALEFSIVAFSITVELA
GLEHT-------YLQLPFS---LVQVFMDCLAGQCLIDACNYFEDAVYSCKWE--
NFNIDNQKTILLMLGTSQRTLMISAGGVSKINFESLMVILKSTYSAYTTLKS--

>HmelOR33

---------------------------------------IPTLYYI--------------------
FTAVVITSYIYVYNISVVWYSFFYC--RTTPT-CYRLSSIIVFSLGVASSIG----
VCKLFFMYLDKIRDIAAECLL------CDAKTIPGSRFEINILKTMRFIK-
KRAMIFWIVIISNGVVYFLKPLLIPGRH-------FSEDDLIVLGKC----LDP-
VLESPNYEIAFALTTAGIFVTVYLPSNITGFLIIVTGYIEAQILALSEEIIQLWVDP----
VHNEELPRQEKNKEKAINEFINNRLKDIVKIHIININIFHQVEDVFRGAIAIEFTLLMISLTA
VLLGLENT-------YMQIPFG---LMQVAVDCFTGQRVIDACVTFERAIYSCQWE--
NFDVKNMKIVLLLLQNSMKTLKLSAGGLSTLSFPCFMSVLNSIYSAFTTLHSTL

>HmelOR34

ILFCITIIIYVYMYIFSTVWYTFVYCRRTD--NRLSAIINFSVGVSSTIA--
FCKFMFMYLFEDKVREIIAGYLV------CDSLIVPGSQFEKNILKTMRFVK-
KRAMIFWIVIISNGAIYMLKPLFTPGRH------------FPEDDFI-LYRLDP-
MFESPNYEIAIFLFSASIGLTVFLSSNITAFLIIITGYVEAQLLALSQEILQIWDDN----
VETFEDLKQLEDQNEDINNLIRDRLKNIVKIHVINMNLIQQVEAVFRGAIAVEFILLFISITA
VLLGLENT-------YLELPYS---LMQVAVDCFTGQRLIDACVNFETAVYFCKWE--
NFDTQNRQTVLLILHISQKTLTLSAGGLSTLSFPCLMSVLNSIYSTFTTLHSAL

>HmelOR37

------------------MHVIRKFGLGFTDLPTLIWNVSVLLRVITLNIDGRNRTPIPKAFHA--
STITLISCYFYVYFVSMIWYVFFYC--RRTSD---WISAIIVFSLGVASQIG--
VCQFDCIFFFRSSVRDVVAEYLT------CDELLIPASRFESNILKTMRHVK-
KRALIFWIVIISNGFVYVTKPLVIPGRH------------LPEDEFI-LLGLNP-
VHESPNFEIAYLVTIAGVMITVYLPSNITAFLIIITGYTEAQMLALAQEMQLIWDDL----
RGNYPIEDHIKMKKLIINNFIKYRLKDVIKIHTINLKLISKVESVFRGAMAIEFTILIISLTAV
LLGLEKT-------YLELPFA---LMQVAIDCFIGQKVIDACEIFEKSVYASKWE--
RFDKSNMTTVLVILQTAQKKMTLSAGGMITLSYPSLMAILKSIYSTFTTLRS--

>SlitOR39

-----R---

EIEDVMRGPNAXGFLFLIVGLVAEXLGLNNT-------ILQLPFT---

LSQMGTDCFLGQKIMDANIKFEHAVYDCKWE--
NFNQVNKKIVLVMLQNSQKTMTLTAGGMATLSFSYFILL--------------

>BmorOR38

--------------------MNLSQSVNEQANEYVKMRLERISKIHSPMLPFEDIQDFRELCCIP--
GSITASYVYAFLISLLWFLFARC--TDPED---FQVAMVVFSLGISSEIG--
STKFFNSIIYIKELRKLFKDYLL------YDATCPAQGRLRLHLLTTLRYVK-
RRAIIYWLVIIGNGFIFAIKPLLVEGRH------------LAQDDLV-LIGLEP-
MRQSPNYEIAYAIMTMGVCFICYPPAHVTMFLIIIVGYTEAQMLALSEELKHLWNDK----
HSRTEREADAAMKSKILNSFVNFRLVQIIKSHSTNVNLIGRVENVFRGSLAVGYVFLIVGL
IAELLGLENT-------YLQVPFA---LIQVAIDCFIGQRVNDANIDFEKAVYDCKWE--
NFDKRNMKIVLLLLQNAQKTVSLSAGGIAKLNFSCFMSVIKSIYSAYTTLRT--

>MsexOR28

---MAWFGFIHT----------------PHTGD---
LLAAMVVLSLGISVQIG--TLKFFYTFVYINKETNIVKYYLE------
CDSLIVPGGRFSGNLLRALRNVK-KRAIVYWLVIIINGITYVTKPMFMRGRH------------
HMEDRYV-IYGLEP-
MFESPNYEFAYFLMTAGLCFICYPPANVTVFLIVVVGYTEAQMLALSKELLHLWTDN----
QHETTLINAQASKNKIINDYVRYRLKEIIKMHAFNIHLVRQVEFVFRGAIAIGYVFLTLGLI
AELLGLENT-------YLQIPFA---LIQVAVDCYTGQKVTDASLIFERAVYDCKWE--
NFDKMNMKTVLLLLQNSQKTMTISAGGITMLNFSCLMSVIKSIYSAYTTLRT--

>BmorOR35

---------------MKLWQSIREFGLEYCDLPTTLQNVASLLRAITLNIDSRHT-ARIPFI----
MTVVITLSYFYVFLVSMAWFVFVRS--AETRD---YLAAMVVLSLGISSEIG--
TLKFFYTFIYIKKVQRIVREYLE------CDHMVVPESRFADNVLKTMRNVK-
KRAILYWVVVIGNGVVYYVTKPLFMSGRH------------HMEDRYI-VYGLEP-
MFESPNYEVAYFLMMFGLCFICYPPANVTVFLIVVVGYTEAQMIALGEEMLRIWEDK----
YHTVGALTNSSEKNKIINQYVKFRLTEIIKMHTTNIQLLRQVEFVFRSAIAMGYVFLVLGL
IAELLGLENT-------YLQIPFA---LIQVLVDCYTGQKVMDASSLFEQAVYDCKWE--
NFDKSNMKTVLLILQNSQKSMRLSVGGITVLGFSCMMSVMKSIYSAYATLRT--

>BmorOR39

---MLWSVFSYF----------------TRADD---
VLAGIVIFSLGVSSEIG--LVKLCFMYANIDKIQKITEGYLK------
SDAASARNSRFSKNILHTMQSVK-KRGVIFWLVIISNGVVYLVKPIVTPGRH------------
FMEDQFI-ILGLEP-
KYETPNYEIGFFMMAVGVCVTCYLPANITAYLITVAGYSEAQFLALGHELANLWPDA----
QLHCRAMNLSQSVNEQANEYVKMRLRELVKIHSTNVNLLRDIEGAFRGAIAVEFLLLIGL
IAELLGGLENT-------YMQVPFA---LIQVSVDCLTGQRVMDANLALERAVYDCRWE--
EFDASNRRVVLLLLQNAQKVATLSAGGIATLNFSCLMAVIKSIYSAYTTLRT--

>SlitOR36

-----------MLTFHEIIHEIRKFGLEYCDLPTMLENVSILLRVLTVNIDSKYKKGITILS----
YIVTAVTAACFYYVFLFSMTWFVFWRARITGE---LVGAMVVLSLGISSEIG--
PFKLFYMCYCMDKTHKIADGFLE------CDANTIKGTRFHSNLMKCLRNVK-
KRAMLYWVVVAGNGVLYVMKPVAMRGRN------------LPENYFL-IYGLEP-
MLETPNYQIAYFMMIASVFFVCYVPASVTAFLIVITGYAEAQMLALSEEMLELWSDE----
DTSEELDVYNPKVKVIINQFVERRLREIIGRHANVINLLNQVEIVFRQAIAIGFVLLVGLLS
ELLGKLENT-------FLQLPFA---LMQVSMDCFAGQRVMDASTMFEASVYDCKWE--
NFDKSNMKLVLVMLQNAQKTMTLSAGGVRTLSFSALMSVFRGIYSAYTALRS--

>SlitOR35

-----------------MWNKIRKFGLEYCDLPTMIWNVAVFLKVLTLNVYGKKKTGIPFI-----
FYIVVVVAALSYLYVYLICMVWFVF--VRSRE---
TEDLGAAMVILSLGISSEIGTKLLYMFLYIKDIRNLTDAFLD------
FDSHMVPGTRMYTNLLRYLRDVK-KRAIFYWAVIMGNGTLYVTMPMLRPGRH------------
LTEDMLV-IYGLEP-
MFESPNYEIAWFMMAASVATICYISANVTAYFIVIIGYAESQMRALSDEVTHVWKDD----
LNYGTDLQDFDRKKMIINEHVFKNLKYIIKRHATIKTLINQVEDVCSGPTAVGLTFLLGLI
SELLGGLENT-------FLQMPFV---FMQVGTDCFIGQRIMDAGEVFEWAVYDCQWE--
NFDHKNMKMILLMLQISQKTSSISAGGMTKMSFRCLMGAMRVTYSAYTAFRSIM

>BmorOR37

---------------------MELGCSRHLKLPCSLHPIGISKHGNTLSELLIYFPAIPKIT----
YAILAVLLTVYYYIYLCSITWFVFVRCPQTGD---LAAASIVFSLGVSSEIG----AIKLFIIAK--
LRDITGEYLQ------CEADM-APGRLRARVGRSLRTVR-
RRAFVYWLVLVVNAFAYDLMPAFLPGRH------------LSEDVFV-IYGFEP-
MFESPNFEIASTLMGVSVVFICYTAGSISAFLIVIVGYSEATMLALSDEISCVWDD-----------
--
ACASECQQPNDFIRARLGKIVAIHTKQIRLIREVEVVFRGALAGGFACVAGLIAALLGGLE
NT-------FLQLPFC---VIQISVDCFVGQRLRDANVAFETAVYNCKWE--
YFDKSNMKTVLLILQNSQKTMGLTAGGVAALDFTSLMTIFKSVYSGVSQTDD--

>CpomOR38

------------------METLDKFGLAHCDLPTMMWNVAVMLRVVAVKIEGGATSIPIFF-----
YLLATVAIVLYFFNYYASMLVFVLVGCRETGD---ILAGIMVLSISMNSLIG--
INKLFYIYRHQDKVQSLVADYVA------YDRIA-PWPGTSALMAELMRSVK-
KRLILFWTVTMGNAFIFNLQPLVMPSRL------------NLYDKHV-VYGLKF-
ILGITNYPIAIVNIVACTFFICYITSSIGGLLIVTTGYSEGRLLALSQEMRDLWSDY----
SENFEGDSEDKKAAKELNNYVKYRLQVIVKSHAINIDVIKKLEGIFRNAIAVEFILLTAGL
SIDLLGLEDT-------YIILPFS---LMQVGMDCYLGQKLMDACKVFEDAIYDCKWE--
NFDVKNRKTVLLMLKISQRTLSLSAGGVATLSFECLMAMYKAVYSAYTALRS--

>DpleOR56

-----------------------MPESGKTKGRRFFVVHYILLRFLGLGWWHSPEENNTNN-----
FPGIYLYYSILTEVVWVAGFVGLESMDPFIGH-KDLDRLMFSLAFVVTHDLT--

IVKLYIFFFKNQQIQDIIRTLEI----------------------KTVRITR-
IFTGLFIFFGWMTIGNGNVYGIIQDLRWVAQLNDTSLRPFRTLPQPI-YIPWEH--
QNDLSFIITFLLETVGLLWTGHIVMTIDTFIGSVILHMSTQFSIMQEAISTAYDR---------
DMIVRTYCTKQEIEIALENTLKSCFRQHQMLISCVDKFASTYSYGFMTQLLSSMAAICSV
MVQVSRDASSLKSIVTSLAFFGAMIIQLALQCFTSSELTHQAERVSEAVMCCKWE--
RMPVRLRRYLVLMIARSQRPLQLTAGGFATMNNDCFLAILKAAYSYYAVLSQRQ

>HmelOR60

---------------------MSNAQSRDGPTRYFAPHYYLLRFLGLGWWHHPDENDTRN-----
FPGWYLYYAILTQLVWVAGFVGLESIDPFIGE-KDLDRFMFSLSFVITHDLT--
LTKLYIFYFKNDNIQDIVRTLNI------RIQDFYQNYEKNRKTTRITKILT-
ASFVFFGWITIGNTNIYGIIQDLRWKAKVAKLNESVSKPPRTLPQPI-FIPWKY--
ESEESYIFTFLLETVGLLWTGHIVMTIDTFIGSVILHMSSQFVILQEALITAYDRP----
TRRNERLARAYYTDKDIETALENTLKSCIQQHQMLINCVKKFASTYSYGFMTQLLSSMA
AICVVMVQVSQDASSFKSIATSLAFFVAMIIQLAIQCFTGSELTYQASLVSDAVMQCKWE
--RMPPRLRRMLLLTMMRAQKPLHLRAAGYAYMDNSCFLSILKAAYSYYAVLSQKQ

>SlitOR14

-------------------------MTETRPKHYFGFHYRILRFLGLGWWHHPEEGNTSN-----
FPGWYLYYSIATQLVWVAGFVGLETIDPFVGE-KEMDRFMFSLSFVITHNLT--
LIKLYIFFFKNADIQEIVHTLEI------GIYDYYQNDDKNRKTVRVSKIIT-
AAFIFFGWVTIGNGNVYGTIQDLKWKSLVATLNDSDVRPVRTLPQPI-YIPWDY--
QKDASYIPTFVLETVGLLWTGHIVMTIDTFIASVILHMGSQFEILHEAITTAYDRE----
DENNERIVKSFVPKQDIDAALKNTFRNCFRQHQVLINCVEKFSRTYSYGFMTQLLSSMA
AICVVMVQVSQDSFKSVRLITSVAFFVAMILQLGMQCLTGNELTLQAERISDAIMQSKW
E--RIPPSQRRLLLIMMMRAQRPLRLTAAGFTHMDNACFLAIMKAAYSYYAVLSQ--

>BmorOR60

----------------------------MVRPCRYFAIHFILLRFLGLGWWHHPHENETRN-----
YPGLYLYYSILTQLVWVVGLVGLETIDPFVGE-KDMDRFMFSLSFVITHDLT--
LIKLYIFYFRNVEIQDIVRTIEI------DLY---RYYQNDDKIRATIRISR-
IFTAAFLFFGWVTIGNANIYGIVQDLRWVKNLNETTSKPLRTLPQPI-FIPWPY--
QEDKHYILTFILETMGLLWTGHIVMTIDTFIASVILHMSTQFAILREAIVTAYDRN----
EENNQIFLESFYSKEHIESVLESTLLSCIRQHQLLIGCVEKFSKTYSYGFMTQLLSSMAGIC
VVMVQVSQGASSFKSVVTSLAFFFAMVIQLAIQCFTGNELTIQAERIADAVMESKWE--
KMPVRLRRLLLVTMMRAQRPLHLTAAGFAYIDNTCFLSILKAAYSYYAVLSQKQ

>HmelOR8

--------------------------MSPSTKRLLTFFCKYVYYAGAGDFLCNGVKES---------
KIYRIYTLISFSVYFIMIFLENLA-ALIGD---
FPEVEGKSAVMFSAIHDIILIKMFLIFYYKSSIIKLNNEMASVM----------
SDIEEESVIKRQQNKVL-WGIGLYVLTVYLSLISYGVESLRKVIVE-------------GTPFYT-
VVTYFPSYYDNSFMASTFRVFYMTWLYMMLPMIAADCMPIVHLIIMAYKFVTLCNYYD
RIRRN---------

FYNNLKVMNNRSAARILKLQCLRGILLHRKLMFLAEEIHRIFGIIMSLQVCESSAVAVLLL
LRLALS-PHMNLTFMTYTFVGSLFFLLALNLWNAGEITYQASLLSNAMFYCGWH--
VCHNDIRCIILIGCAQAQKPLILKAFGIQDLSYSTFVSV---SICQSLKVAP--

>BmorOR8

---------------------------MSLSTRCLLKDFCKYVYYAGA--GNFWYEDIYKET------
VPYKMYVVISFFTYTVMIFLENLA-ALFGK---
LPEVEKNSAVMFAAIHNIVLTKMFLLLYHKRSISKLNCEMAAVG----------
ENLEEASIMRRQFRKMR-LGTALYFISVYLSLVAYGVESARRTIVE---------
GAPFYTVVTY----
LPDYDNTTVLASFLRIFFYITWLYMMLPMMSADCMPIAHLITMTYKFVTLCRHFDQIREK

FQINVKIMAKTEATEILKLGFIEGIKMHQKLMYLADEIHRVFGIIMALQVCESSAVAVLLL
LRLALS-PHLDLTFMTYTFVCSLFLLLALNLWNAGELTYQASLLSNAMFYSGWY--
FCDFEKIRRVLIGCAQAQKPLILKAFGVLDLSYETFVSVM--TYSVFAVFYKRG

>DpleOR58

---MAALFAD----------
FPEDEKRDSVTFSVAHTIVMIKMFSIIVNKKNIKKLISDIIAVC----------
ESHEEESLMNEKYKVMK-INVIAYFFTCYSSAACFVFEGLRKMYEV---------------------------
----------------------------DGFTMVILIMFKYKLITLRNYFDKLRKD---------
TEILHQSSDPQLAVNHLVNGLIKGIIMHKELLRILKEIDKAFGTVVALQLCQSSGAVSLL---
-----------LGIAASL-----------------------LSESIFYCGWH--
NSGRNIRRIVLHACIQAQRPLVMKAFKMIELTYGTFLWSVKDQGTFKELLNT--

>HmelOR21

-----------------------------MKDYLILKKICKKIFLVGS--GNFWYEESEIGNDD--V-
ILYKIFNYILFLVYALMTLLEIMA-ALIGD---
FPEDEKSDSVTFAVSHTIVMIKIFSVIWNKELVKKLIQDLISVC----------
EIHEEEYLMKEKYNIMK-INVIAYFITVYGSAACFVFEGLRKMLTG----------SHFVTAVTY-
YPSYDD--
NSLLATITRIFMTMALFMMMLTMIVSVDCFAVVILIMYKYKFITLKNYFQNLRLE----------
-
FEAFDIQENCSGEKLLKGLIDGIIMHEKLLRISKDIDKAFGTVMALQLCQSSGSAVSLLLQI
AVT-------MKILFFVIALFFLLGLFLCNAGEITYQASLLSDSIFYCGWH--
FPRRNLGRLVLQACAQAQRPLVMKAFKMLELTYGTFLLVIRGTYSVFALFYA--

>SlitOR19

-----------------------------MKNHYILKTYCKRIFLIGS--GNFWYKESEIGND---
QSLLYRTYNAVLYFIYGFMTILEIMA-ATMGD---
FPPDEKRDSVSFAVSHTIAMIKIFSVIANKSLIKKLNHKMVTVC----------
ERYEENTLMAEKYKIMK-INVLAYFVTVYASAFFFVFEGLRKMLTG-----------
SHFVTVVT-YYP---

-------KFEDSIGATSARVLATVILYIL------MIT-----------------MIMSVDSF----------------------
----TM-------IYLIMYKYK----------------FITL-KQ---YY-------

>MsexOR29

------------------------------MKNYYILKTYCSKIFLLGSGDFWYENKVIGDD---
KRILYRMYSCALFFIYGFMTVLEIMA-ALMGE---
FPTDEKRDSVTFAVSHAIVMFKIISVVFQKELVKTLNRKMVTIC----------
EHYEEQALMSEKYRIMK-INVIVYFLIVYGSAACFVFEGLRKLFDG-----------SHFVTVVT-
YYP-
DYEDNSMFANSVRILATVILFLMLMNMIVCVDSFTMVYLIMYKYKFITLRHYFENLSVT-

IDKLNTPGNEEVVAKMLTDGLVEGVEMHSKLLRLSKDIDKAFGTVMALQLCQSSGSAV
SLLLQIALS-DQLTFVLKIVFFVMALFFLLGLFLCNAGEITYQAAQLSDAIFYSGWH--
VCSRHVRKIVLLAIMQAQQPLVMKAFKMLELTYGTFILVVRATYSVFALFYAQD

>BmorOR22

---------------------------MNKNHYILKTYCDKIFLVGS--GNFWHQKTESRND---
KTLLYKIYSCVLFFTYGFMTVLEIMA-ATMGD---
FPEDEKRDSVTFATSHTVVMIKFISIIKNKELLKTLNRKMMMIC----------
EAHEEQTLMDEMYRTVK-INVVAYCVAVYGSATFYVFEGLRKFYNG-----------
SHFVTIVT-YYPSND-
DDTMLASIVRIATTLVLLMMLLSMIISVDTYTMAYLIMYKYKFITLRHYFKRLREN--------
-
VDELVAAGKARLAAEKLAQGLVEGIKMHNELLSLSKDIHKAFGTVMALQLCQSSGSAV
SLLLQIALSQLTFTMGMKIFFFLAAMYLLLALFLCNAGEITYQASLLSDEIFYCGWH----
NSPVIRDVLIAILRAQSPLVMKAFKMVELTYATFILVVRSTYSVFALFYA--

>BmorOR21

---------------------------MNKNHYILKTYCDKIFLVGS--GNFWYQKTESRND---
KTLLYKIYSCVLFFTYGFMTVLEIMA-AMMGD---
FPEDEKRDSVTFATSHTVVMIKFISIIKNKELLKTLNRKMMMIC----------
EAHEEQTLMDEMYRTVK-INVVAYCVAVYGSATFYVFEGLRKFYNG-----------
SHFVTIVT-YYPSND--
DDTLAATIVRIATLVLLMMLLTMIISVDTYTMAYLIMYKYKFITLRHYFKRLREN---------
VDELVAAGKARLAAEKLAQGLVEGIKMHNELLSLSKDIDKAFGTVMALQLCQSS------
GSA------------------VSLLLQIA---------------LSDQLFTMGMK-----------
IFFFLAAMYLLLALFLCNAGEITYQVCTSIV-------------

>CpomOR19

----------------------------MKNYFILKNLCRKIYLVGA--GDFWFEEGEISKG---
KSLRYQFLCFVLFSIYIFMTVLEIIG-VFFGD---
MPKDERSDCTTFAVSHTIVLGKMFSVILNRKRVKELNRKLVEIC----------
ANHEDEHRVAENYRIMK-INIWAFAVSVYGSFIFFLFEGIRKMMSG----------SHFITIVTY-
WPFYED----NSIIAVSFRFFTLVLAVMMATMICIDSFAMIILIMYKYKFITLRYYFEGLRER-

----
FDRNNYTGNEEYATELLHAGFIEGIVMHSNLTRLSKDIDRSVGTVLALQVCLSSGSAVSL
LLQLALSDLTVAAQLKIIMFVIAVFFLLALFLCNAGEITYQASLLSDSIFYCGWD----
RRDLRRLVLFSCAAAQRPIVMKAFNMLELTYGTFIQVVRGTYSVFALISAQN

>HvirOR19

----------------------------MKDRDILFKYCKVMFYIGS--GNCWYKEDEIGND---
RSILYRVCSASLMLLYSYMAIFELIA-FAFGN---
FPEEEKRQALIGGAGHTVMLLKALFLTTKKLPIRSLNRKIVSIC----------
EDYEDSALMARKYKIMK-INAISYLGLVNGGVLLYIIEGLRNMLNG-----------SHFVTVVT-
YYP--SFEDDSMLATIVRVFNIIFIMIMLTIVISVDTYIVTYFIMYRYKFITLRKYFENLRND--
----
FFTLIERKEVELATEKLANGLVEGIKMHSSLIRLKPEIDKAFATVSAIRVFESSLAVCL-
LFEISPSDQRIPIEVKTMIFIFALFFAMGLFLCNAGDITYQASQLTDAIFYCGWQ--
SCPRPIRKMVLLAIMQAQRPPVMKAFKVLELNYATYIQLVRSTYSVFTLLCAQK

>HmelOR20

-----------------------MIYETNETIKFISIANKIWCFCGI--HNLWIENIFDN-------
KFTKYFNKIITFSLMLFVSMEMGALFTRTD---
LTEKQCSDLYLFVPTHCILGSYYIGSQYYKKNIKRLLIMLSIDMK---------
SVYNDSEIEKQMIKRAT-YYSIAYVMSASSALLSY--------------------EGSFTTVVTA-
WPDSED-TSVFSSFARLFFYLWWAFMARISIGIVLTIIISVGISHQFKILQKYFYSLNDIFNN-
----
ETIDSHEKQLKYEQSLKVGIKVHSQALWCTSQCQLSCSFVFGGQMMVCVYALSFLMLQ
MMNSERTLTTAMVIATTSICVLICTGLIMWNAGDI--TAALIPSAIFSSGWH--
HCGDTLRRVTSIILGIAQ--------GQVNCKRSIMVER---HWVFK-------

>SlitOR37

------------------METTTTSYTRSKTTNFFYKINFIVYIFGL--PNFWIQDLKLSKRF--
SNKFYDKFSMFNNTLIFLLIIFELCSYFTQTQ---
LTETHQSNRLIYAISHPMLFMRVMMTSIKDR-VKLVMYSLNVGLK---------
RVHNDPEVEKQMIACTI-MYLSALLLSCLMSMLMYAAEGFGEVIRH---------
GKTFTTIITA-YPRVED-
ASDMANVVRAICFIWWIFLTRIFAVYILVISLTTCLSYQYKKPAELFHSLNDIFER------------
--
NDLSQIEKEEQYEAGFKVGIKLHADTLRCTQLTQSVCRGVFSGQIVFNILLLVVLMAQM
ANSERTLVNLGSAGFTACAVLISTGFYMWNAGDVTVEASHLGTAIYFSGWY--
HCSVRIRKLVVITMSEAQRPVVLKGLGYIDLSYQSYIRIVKSSYSVFSVLF---

>MsexOR26

--------------------------------------ITYLYLFGL--PNFWIEDFKLPK-W--
FMRSFDIFTKIINNVLYFFILMEMIAFFTQEN---LSERQKSDLLVYGISHP--
ILSYRVFISYKEDLRAVLLDLVVTLK---------RVYNDVKVERQMIKKSL-
LYSSALVFSCILAMFFYTFDSILHVIRT---------GATFNVVITT-WPKVED-

RSTLANAGRIVFYIWWFFMSRVSGAYTTVICLTTCLSHQYTNLRSYFENLNNIFET---------
------
NFDQAVKEQKYEDGFKVGIALHLDTLRCTRECHSICQGVFSGQIILNILLLVVLMSQMVN
SERTLVTAFATASSASAVLISTGYFMWNAGDVTVEASRLSSAMYLSGWH--
RSSITIRKLVVITMFNAQKPVILKGLGIVDLSYQSYLSIVKSSYSVLSLLY---

>BmorOR20

--------------------MIQASKYPNSKTKELFRKIAHIAYICGL--PNFWIEELNLPKS---
FIRVYDKIVRIFNVATYFFLGIEIAAHFTQHH---LTNKQKFDLLLYSISHP--
ILNGYGVIVSRQVKKVLLDLIVNL-----------
KVKYNDPVIEEAMIKISMTYSVSFITNCVLSMLTYTFDALLMVYKK---------
GVTFNVIITA-
WPDVEDTTTEASIGRIGFHIFWWLFVTRPFAVYVLVINLTTCLSHQYMNLQSYFFHLEDIF
KE---------------
NLSQNEKEAKYEAEYKIGVMLHANTLRCTRRCHMVWNGVMSGQIIFNISLIVIIMAQMM
NSDRTLVNTFGTVLTASAILISTGFFMWNAGDVTVQASRLATAMYCSGWQ--
CRGKSIRNMVMNTIAVAQRPLVLRGLGVIDLSYQSYLSIVKASYTVFSVIY---

>SlitOR21

-------------------MDNFSGSYRQTKTTEFLINLNKFIFVFGL--PNCWVDNLDFPDN---
FMKVLNVLSIYGNWSVIIMNISEYGAIYTQNN---LSERQKSDLMLFLISHT--
IIGFRIRICHQRDIRNVMYKLGIALK---------AVHNDCEAEQKMITRSK-
FFSWALIFNCVMSVIMYTIEGVLRVIRT---------GDTFNTVITA-WPDVHD-
RSVLSNIGRTVIYIWWIYLTRIFSVYSLVICLTIAVSHQFKNLKSYFYSLSKIFEN-------------
DNLSQTEKEQEYEKAFKVGISMHSETLKCTDEIQTICREVFSGQIIFNLTLLIVLMYQMMN
SPRTFSNVLTLGLTALTILFSTGFFMWNAGDITVEAEGVPVAMFSSGWE--
NCSVRVRKLIVISMSQAQKAVALTGLGIIALSYQSYVSIVKSSYSVFSVLY---

>HvirOR21

-------------------MDHFSGYYKSSKTTEFLINLNQFVFIFGL--PNFWVQELDISDS---
FRKIVGYLNKYGNWSIFGLILAEYGAFFTQKN---LNQRQSSDLVLFMISHS--
IIGFRVRICHQEVIRNVMYKLGIALK---------EVHNDSEAEEQMIKRSK-
FFSWALILNCVISFLMYTIEAVLRVIRA---------GVTFTTVITV-
YPDVEDRSGLSNGVRVMFYIIWCIYLTRVFAVYTLVICLTIAMSHQFKNLTSYFYSLSSIF
DD-------------
DQMTQAEKEQEYERAFRVGIKIHSDTLNCTGDIQKICRDVFSGQIIFNITLLIVLMYQMVN
SARSLTNALTLVMVALSILLSTGFFMWNAGDITVEAKSLPTAMFSSGWE--
HCRDSVRKLIVIAMMQAQEPVVLTGLGIIALSYQSYVSIVKSSYSVFSVLY---

>CpomOR34

--------------------MMPIKPFQNNRTSDLFHTICKIIYLSCA--TNFWFEDIDYPA----
FMKIYNSTSRVLEVTVAVLIISDWG--AFWTQ-
PNLTEKQSNDRMLFAFSHVVLYSVYCSVIYYKREIRELVMTLTVRLK---------
EVCYDGSIEKMMLRTTF-RYTTAFVFVCSSTLFSFGIGSGFQALTT---------NATFTTIIPI-

WPDVED-RRLVAGAARIILYIVWWIFLV--RFISVYIILLISTIGIAHQFKNLCKYFEDLTDI---
----
FEGSGSQEEKERRYENAFKVGIKMHSITLWCMRQIQLVGGVAFSGQVIINVSVLGLLMIQ
MMFTERTLIAVMPIIFMVSSVLVGTGVFLWNAGDVTIEASRLPAAMFHSGWH--
RQSVRVRKLVTIAIAQAQKRVRIKGLGFIELSYESYVTIVKSSYSLFSVIY---

>MsexOR9

-----------------------MTSPDSKIKEFFRKFTLITYLCGL--ADFWIEDLDLPTQ---
FIKCYDTFCKIFNNFLYLYVIAQILSLFTQHN---
LTEKQRNYQLMFCVAHPFVVTFPTLIAKHRKKLQSVLFRLVVSLK---------
LVYNDLDIEKEMIKRIK-FYLFAVSAPFWISMISYCVDSYVQVVTS---------GTTFNIMILA-
WPLVDD--HSVIASLARFIYHIIWILFETRVTAAY-VMVISLTTCISYQYRNLRSYFESIHRV-
----
FEEDLTQKEKEKKYEKLLKIGIQAHSDTLRVTKDGTEACSAVLSAQVLFNTVFLVILMYQ
AVVVNENRSLVFSTLCTVITLLFSTWFFTCNAGDVTYEASLIGTAMYTSGWQ--
NCSLSIRKLLVIALARAQEPIILKGFWILTLSHQSFLSIVKFSYSIFSLLY---

>MsexOR5

------------------MKVPLKKFRPTETTKLLDELNKLFYIFAF--RGFWVEEVKLPKT---
FVKIYDAMYPALNVSLVIFCALQIGAHFTQKH----
LNIQQKININIMGIAQPLTKCCLNCMYYKEECKQVLYHLFVAVK---------
EIYNDEETEKMLVKKLK-FSLWVYMFSSVSTTLLYGMYALIEMVRS---------
GSTFVGTVTA-WPDTTD-
TSALAACARVYLYFWIVYTSTGSMVILMVLTFFMGLTYQYKNLQKYFENLNSIFED-------
----
KQVTHEELEMNFEKALQKGIKAHSDTLWCVNQCQTICKRINMAIVLINTILIVLMGFLILK
V-------GTCLIVLGMCLMILAFFMWNAGDVTVEAQKLSDAIYSSGWY--
NCSARIRSLVVIAMIQAQEPVVFTAFGVIELSYETYVAIIKSAYSVFSVLY---

>MsexOR6

-------------------MEETKKYAPTDTTKLLDKFNKILYVFGF--RGFWMGDVKLPKT---
VMKIIDILYPVLIITQGTFGTLQLGAYFTQKH---
LNSVQKIDALVMGFGQPMMLLYCLNCRYYKKACREVFYHLFVVLK---------
TVYNDKETEENMVKRLK-LYFGAYLIFSLLVVLLYGSYASVETVRK---------
GATFVTVVTA-WPDVTD-
TSKLASYARVGIFMWSVYSFTTSVVIIIVLIIFLGLTYQYINLQRYFENLNSIFEN-------------
DRLSHEEMEKNFEKALQNGIKAHSETLWCVKQCRMICSAINAGVILLTTGTLVIL---
MPAILGSKDDLLEAMMYLIVLMIMAFFMCNAGDTTV-EAQKLPNAIYSCGWY--
NCSARIRSLVVVAMIQAQEPVVFTGFGVIELSYENFVTIIKSAYSLISVFH---

>BmorOR19

-------------------MHEFVINVQNETTKLYDQLNIILYILGL--QGIWVDEIKLSRR---
FHVFFKVVTFILHIMCGMFAGLQFFAIFTQNS---
LNSQQKSDVIVIGISNPMAYIFCINFIRNRNEIKDLFYHLAVVLK---------

IYYNDVEIEKSMVNKIK-SYLSTYVFASITILVSNGIIAFFQTINS---------DEPFLGIITA-
WPDKTDTSKTASYARIGFYLFWCIHFFRISTVFAVIVCILISIKYQYKFLCSYFESLNKIFDD

ETSSHEVKEAEFENAFCNGIKIHTQIIWCVRRCQIMCRTVFSANIMLDTFVLVILMLAMV
NS--ENDFYCSQMSSVLVTVVLMAFFMWTAGDINVQASQLPDAIYGSGWY--
NCGKSIRSLVTISMNKAQQPILMWALGFVELSHKNFVAIIKSAYSVFSVFY---

>SlitOR33

-------------------MSNQIKMYTPNEMTKLLGKLNVICFIFGL--KNIWVED-VKLTNR--T-
RSCNRLLLFAEIFYIIFASTILGSLFTQKN---LSEKQKTDQMMFSITLP-
GNIFHYILLYRRHEIRNLLYHLAVVLK---------EHYNDEDLEREMIKKIK-V-----
FSISLCGLVGMGLSAFYRVVTA---------GETFVTITSA-WPDIHD--
RSTAAGLVRVFVYFWWYPFAARIM-VTFLILVTMLVSICYQFKNLQSYFYSLDEI---------
FSDGTQSQEQKE--
KKYENAFKLGIKMHSLTLWCKNQHQHVSKELFATEIFLFFGMLLSQLTA--
LLDRNMTQLGTMFITSVTTCLALGFFMWNGGDITLEASKLSEAMYCSGWQ--S-
SVRVRKLVANALRQAQNPVVYKTLGIVEFSYESYVRLVKMPYSAVSVFY---

>SlitOR34

-------------------MNFFRNPEVPTQLYKETDTTKYNKFWFLCCCPDFWVKKVDYPDP---
FTRVYRPSMLVNHIVMVIFCSSCFLSLWTQHD---
LSQSQKSDRLAYAASTPIITVYHFVILYYTEDVKQVLYKLAVVLK---------
VDHNDKNAEDEMIKQSK-LHNGIFFSSCVCNMVLVGLYNFYRAVTT---------
DATFITCISA-WPDIED-
RSTLAGLTRVVVVYFWFSHVTRNMGVFLITHTALLLLSQQYKNLQSYFEDLNRIFLE--------
------
KELSQEEQELKFEVRFKKGIEQHALTLWCVDETQRIFQITFSSHVLLWCLLISILPDVLNN
DTHSPTMLVSNAPRVCAALVGLGYFMWPAGDITVEASNVPHAMYGSGWQ--
CCSPRIRKLVVLAMMQAQRSIELKAFGHLTFSYETYVAIVKMSYSLFSVLY---

>DpleORco

---------------------MITKSENRGLVSDLMPNIKLMQMAGHFLFNYHSDNSGMSSL---
LRKCYSSIHAFLIVMHYLCMAANMA--KYSEE---VNELTANTITVLFFSHS--
IIKLTFFAITSKNFYRTLAVWNQSNSHPL------FTESDARYHQLALTKMR-
RLLYFICGMSFFSVICWVTITFFGESVVDKETNETITEPAPRLPLKA-WYPFDA--
MSGTMYIAAFAFQVYWLLFTIFIANLMDVMFCSWLIFACEQLQHLKAIMKPLMELL----
QNFGQNNNNPNGLTQKQEMLARSAIKYWVERHKHIVRLVSSIGDTYGTALLFHMLVSTI
TLTLLAYQATKINGLNVYAFSTVGYLSYTLGQVFHFCIFGNRLIEESSSVMEAAYSCQWY
--DGSEEAKTFVQIVCQQCQKAMSISGAKFFTVSLDLFASVLGAVVTYFMVLVQLK

>HmelORco

---------------------MMTKIKTQGLVSDLMPNIKLMQAAGHFLFNYHSDNSGMSTL---
LRKVYSSVHAFLIMIHYLCMAANMA--KYSEE---VNELTANTITVLFFTHS--
IIKLAFFAITSKNFYRTLAVWNQSNSHPL------FTESDARYHQLSLNKMR-

RLLYFISGMTIFSVVCWVTITFFGESVRDKETNDTLTEPVPRLPLKA-WYPFDA--
MGGTMYIIAFAFQIYWLLFSMAMANLLDVMFCSWLIFACEQLQHLKAIMKPLMELL----
QTFGQQNNNPNGLTQKQEMLARSAIKYWVERHKHIVRLVSSIGDTYGTALLFHMLVSTI
TLTLLAYQATKINGLNVYAFSTVGYLSYTLAQVFHFCIFGNKLIEESSSVMEAAYSCQWY
--DGSEEAKTFVQIVCQQCQKAMSISGAKFFTVSLDLFASVLGAVVTYFMVLVQLK

>Bany_Orco

FFAHT--IIKLTFFAITSKNFYRTLAVWNQSNSHPL------FTESDSRYHQLALTKMR-
RLLYFICGTTIFAVTCWVTITFFGDSVRMLVDKETN-ETLTEPVPRLPL---DA--
MSGTMYVAAFAFQIYWLLFAMSIANLLDVMFCSWLIYACEQLQHLKAIMKPLM-------
QN---PNN-
PNGLTQKQEILARSAIKYWVERHKHIVRLVSSIGDTYGTALLFHMLVSTITLTLLAYQAT
KINGLNVYAFSTVGYLGYTLGQVFHFCIFGNKLIEESSSVMEAAYSCQWY--
DGSEEAKTFVQIVCQQCQKAMSISGAKFFTVSLDLFASVLGAVVTYFMVLVQLK

>SlitORco

---------------------MMTKVKAQGLVSDLMPNIKLMQAAGHFLFNYHAENGGMSGL---
LRKIYASTHAILITIHFACMGINMA--QYSDE---VNELTANTITVLFFTHT--
IIKLGFFALNSKSFYRTLAVWNQSNSHPL------FTESDARYHQIALTKMR-
RLLYFICGMTVLSVVSWVTLTFFGESVRSKETNETLTEVAPRLPLKA-WYPFNA--
MSGTTYIIAFAFQVYWLLFSMAIANLMDVMFCSWLIFACEQLQHLKAIMKPLMELL----
QTFGQQNNNPNGLTPKQEMLARSAIKYWVERHKHVVRLVASIGDTYGTALLFHMLVST
ITLTLLAYQATKINGINVYAFSTIGYLSYTLGQVFHFCIFGNRLIEESSSVMEAAYSCQWY-
-DGSEEAKTFVQIVCQQCQKAMSISGAKFFTVSLDLFASVLGAVVTYFMVLVQLK

>HvirORco

---------------------MMTKVKAQGLVSDLMPNIKLMQMAGHFLFNYHSENAGMSNL---
LRKIYASTHAILIFIHYACMGINMA--KYSDE---VNELTANTITVLFFAHT--
IIKLAFFALNSKSFYRTLAVWNQSNSHPL------FTESDARYHQIALTKMR-
RLLYFICGMTVLSVISWVTLTFFGESVRNKETNETLTEVVPRLPLKA-WYPFNA--
MSGTMYIVAFAFQVYWLLFSMAIANLMDVMFCSWLIFACEQLQHLKAIMKPLMELL----
QNFGQQNPNPNGLTPKQEMLARSAIKYWVERHKHVVRLVASIGDTYGTALLFHMLVSTI
TLTLLAYQATKINGINVYAFSTIGYLSYTLGQVFHFCIFGNRLIEESSSVMEAAYSCQWY--
DGSEEAKTFVQIVCQQCQKAMSISGAKFFTVSLDLFASVLGAVVTYFMVLVQLK

>BmorORco

---------------------MMTKVKTQGLVTDLMPCIRLLQAAGHFLFNYHADTSGMNML---
LRKIYSSAHAVLIVVHYICMGINMA--QYKDE---VNELTANTITVLFFAHS--
IIKLAFFAFNSKSFYRTLAVWNQSNSHPL------FTESDARYHQISLSKMR-
RLLYFICGMTVFSVISWVTLTFFGESVRSKETNETLTEPAPRLPLKA-WYPFKT--
MSGGGYVFAFIYQIYFLLFSMALANLLDVIFCSWLIFACEQLQHLKAIMKPLMELL----
QNFNAEN-
NPNGLTAKQEMLARSAIKYWVERHKHVVRLVASIGDTYGTALLFHMLVSTITLTLLAYQ

ATKINGINVYAFSTIGYLVYTLGQVFHFCIFGNRLIEESSSVMEAAYSCQWY--
DGSEEAKTFVQIVCQQCQKAMTISGAKFFNVSLDLFASVLGAVVTYFMVLIQLK

>CpomORco

----------------------MMGKVKSQGLVSDLMPNIKLMQMSGHFLFNYTEETGGMSLL---
LRKIYAAMHAFLILLNFVCMGINMA--QYSEE---VNELTANTITVLFFAHT--
IIKLAFFAINSKSFYRTLAVWNQSNSHPL------FTESDARYHQLSLDKSR-
RLLYFICGTTCLSVVSWVTLTFFGESVRDKESNDTLTEPAPRLPLKA-WYPFDT--
MSGSMYIMAFVYQIYWLLFSMLIANLLDVMFCSWLIFACEQLQHLKAIMKPLMELL----
QNFNSTNPNPNGLTQKQEMLARSAIKYWVERHKHVVRLVASIGDTYGTALLFHMLVSTI
TLTLLAYQATKIDGLNVYAFSTVGYLRYTLGQVFHFCIFGNRLIEESSSVMEAAYSCQWY
--DGSEEAKTFVQIVCQQCQKAMSISGAKFFTVSLDLFASVLGAVVTYFMVLVQ--

>MsexORco

------------------LSGHFLFNYYADNSGMTMLLR------------------------------
KMYSTVHAILIFVQFVCMGVNMA--MYADE---VNELTANTITVLFFAHS--
IIKLGFLAFTSKSFYRTMAVWNQSNSHPL------FTESDARYHQIALTKMR-
RLTYFICFMTVMSVVSWVTITFFGESVRNKETNETLTEPAPRLPLKA-WYPFDT--
MSGSMHVFVFVFQIYWLLFSMSMANLLDVLFCAWLIFACEQLQHLKAIMKPLMELL----
QNFVQNTVNPNGLTQKQEMLARSAIKYWVERHKHVVRLVASIGDTYGTALLFHMLVST
ITLTLLAYQATKINSINVYAFSTIGYLCYTLGQVFHFCIFGNRLIEESSSVMEAAYSCQWY-
-DGSEEAKTFVQIVCQQCQKALTISGAKFFTVSLDLFASVLGA-----VV-----

>HmelOR65

--EMTLRCVGCWDWYREPKNEYQIF----
FNNIYLGFVLFILLNLPISLIVHLY--TVWTD---MMSHLENMADGLPLFAS--
VFIVAYLSAHKNDLYNLVKYMNENFRWHS------ARGLTNMTMLKSYQTAK-
SFAYWYTGCTLFSVTMYVAMPIFAHLWT-------------KNPLQP-WIYKDL--
TSSPAIVLVFMRQCLAQAFVGLAVGQLGVFFACNSILLCGQLDLLCCSLRNARHAD----
SSYHYDDKVVNIYSLHYDEATSEALRECGRVCQIINEFKVKFEEFVSPLLVLRVVQVTLY
LCTLLYA-----ATLKFDMITVEYLAAVALDIFVYCYFGNQIILQADRVTTAAYQSSWP--
SMGVRPRRLLLNLLLANLRPTVVRAGRFLPMNLHTFVLIIKTSFSYYTLLVNVN

>SlitOR1

-----------------MASNVDKTTLNSEKPEHYIHYIEMPLKLVGCWDLFPNSVTENRKI----
FNDIYLGIVLFVLTHIPMVLTVHLY--TEWQD---IMSSLGTIADALPLLVS--
LVIVAYYAIYRRNLYELLNYLDKNFKYRS------ARGLTNMTMEQSCMTAR-
RFGRIYTACTMFSVTMYATLPVIVHLWT-------------KEPIQS-WIYMDV--
TQPPFFEFVFMLSCLAQMYVGLAMGQFGVFFASNSILICGQLDLLCCSLRNARYTT----
HNYVDAKTNFDIYSPEYDDATMEALRDCASLCQVVNKYKEMFESFVSPLLALRVVQVT
LYLCTLLYA-----ATLQFDMITVEYLAAVALDIFVYCYYGNQIILQADRVSTAAYQSMWH-
-TMGIRPRKVLLNILLANRRPVVVRAGKFLPMDLHTFVVII--KTSIF-LLHSSS

>DpleOR44

---DCIFVFLFIFLYNCLLTINLY--
FLPSD---LNVIIEDLVLYFTCLSI--MSKVLTYFIRKESIRDILRFLDG-------
DKFQPKNEKELQIINAAKKFNE-KYWKVVAILSYSSNITHVSTPIILHLID---------
SVPVVFPVSG-YFFLSE-
DRRRENLYYIYLFQAVGIHFNMLYNINIDSFFIGLLILAIAQIDILDEKLKNIATN----------
IWDADGMVNRDIDEICIRKLNDCILHYNNISKFCLLVEEVFSVTLFVQFSMSSFVICVCLY
RLTQP-APLPYYLFLGSYLFIMIIQIFVPCFFGARIMDKSSQLSHAVYSCDWT--
PRSRRFKSSMRLFVERSLRPHSITGGKIFPLSLVTFTSIMNAAYSFFTLLTN--

>MsexOR41

DLIIEELVFYFTELAA--ISKVLTFVFFRDKLAKILDALED-------
PMFQAANGREQKIIDGAKRFNK-RYWKIVATVSLTSHATHILSPIVEHLFL---------
SVPLQLPTCR-YSFLSE-
NTIQQFIYPLYLYQTLGMHSHLWFNANIDSFFLGLMILIIAQLQILDLRLRTVTDV-------
KKNDDIGQANTSEARANYSLTQLNKCIVHFDEVGKFCGLVEDTFSMTLFMQFSMSSCIL
CVVLFRFTLP-APFEYYIFLGTYMCVMISFIFVPCWFGTRVMELSVLLCSSVYECDWT--
AMPKKFKSNLQLFVERAKRPLTITGGKMFMLSLTTFTSIMNSSYSFFTLLRN--

>BmorOR13

--------------------------MAPKQIDCFEINWKFWKFLGI-----WSENKPHRY----
YKYYSKIFITFFVILYDVLYTINFY--FVPRQ---LDLIIGEMLFYLTELSV--
LSKVFTFIIMRHKLKIIFEILES-------DAFQTDTEEELKILHRAKVFIK-
RYWKIVALVSITANLTHISSPLLKNLIF---------KVELVLPVCS-YSFLSE-
SFLKTFEYPLYFYQIVGIHFHMLYNLNIDTYFLGLMILIIAQLDILNVKFRNLKSG-----------
-----KDHTQLNESIMGLNKNLDHYNEIERFCSLVQNIFSFTLFVQFSMASCIICVCLFSFTLS-
VPVEYYIFLATYMFIMIIQIMVPCWFGSRIMDKSILLSSAIYNCDWT--
SNSKDFKINMRLFVERANKPLSITGGKMFSLSLATFTSIMNSAYSFFTLLRY--

>SlitOR8

--------------------------MESHQIDSLNINLMYFKFLGV-----WPGNNPWRF----
YNYYSKAFIMVFVIIFILLFSINFY--FLPRQ---LNIFIEETIIYFTDLAV--
TSKVLTFVFMHEEIIEILDILEC-------DLFQPEDDEEMIILENAKKSSK-
TYCKIIMIVSYLSSTLLILSPLISHLIL---------GVELLLPICR-YAFISD-
EYRLMFIYPIYLYQIVSIHFHVLYNVNVDIFFFGLMVLAIAQLDILDRKLRKVTDG-----
NKVEDTDGEASRSPIDKNKEDVMKINQCIIHFIKVCRFCELIEDAFSATLFVQYSAASCIIC
VCLCRFTMP-AEMGYIVFLASYMSLMISQMMAPCWLGTRLMDKSQLLAFSVYNCDWT--
SRSRQFKSNMRFFVDRANKPLSITGGKMFNLSLGTFTSIMNSAYSFFTLLQHFQ

>HvirOR8

---------------DSYCKIHNSFAIKIFFIMVFRQIDCFKINMKFLKFLAIWPGKDFTRR----
YKYYTVAFLTAYFIIFMILFTINLF--FLPKQ---LDIFIENMVFYFTDSAT--
LSKVMTIGFMRKKILQLFEMLES-------DIFQPDNAEGLAVIEKAKKFNK-
LYWNIILAVSFASCASNLFPPIIAHFIL---------GTELVLPICN-YGFLSE-

DFRQMFGVPLYLYQGSAMMFDMLYSVNIDTLFAGLMVLAIAQLDILGIKLRRVTDK-----
EVLEETDSETSRQHRDNHKEAIKKINHCIIHYEKIHKYCSLVEDVFSITLFVQFGMASCIICI
CLMRFTMP-APLVYYLFLATYMFVMILQILVPCWFGQRIIDKSNLLAFSAYDCEWT--
SETRQFKSSMRIFIERAHKPLSITGGKMFCLSLVTFTSIMNTAYSFFTLLQN--

>SlitOR20

--------------------------MGYNQIDCFKIHLTILRVLGV-----WPEENPSVY----
YVYFSRIFVFIFTVLYVIIYTMNFY--FLPQQ---LEIFAEELIFYFTNVGA--
LSKALAFIFLRDKVKKMLYMLES-------EMFQTDNPEEVKLIEKTKEKSL-
FYWKITFGLSVSANTVNVFLPFVLHLIF---------SIKLEFPVCR-YSFIPE-
QYEAIFLYPAYLYQSIGITSHMLYNVNIDTFLLGVMFLAMAQLDILDRKLRKVTDV------
CVNVDAPRGSIDKMIDDQNAVLEINKCIKHYDAVCEYCKLIQDAFSEILFVLFSSGSCKIC
MCLFRFTMP-AETQYFVFLTLYIVVMTLQVMVPCWFGSRLMEKSSQITFAVYDCDWT--
PRCRRFKSNLRLLVERANRPIIIKGGKMFLLSLATFTAIMNSSYSFFALLRN--

>HmelOR13

--------------------------MPLKQSDCFSINKKYWTLIGIYSTIDSKM----------
YIWYSRIVLFIFIFIYDTLSTLNFY--YVPRD---LDHFIEELIFYFTELSV--
ISKVLTYILMRESITKILCVLDS-------EKFEPDTEKGIQITTAAMKFNV-
KYWRIVAFVSYFSHLIHILSPLVAHLSL---------SVPLVLPLCS-YSFLTE-
SFKERFIYPLYTYQCLGMHAHMLCNLNIDTFFLGLMIFTIAQLEILEDKLVTL----------------
--------TNISRLPNSKTGDRNISNKNEFCDLVEDVFSITLFVQFGIASCIICVCLFRFTLP-
APIQYYMFLASYMFVMTIQIAVPCWFGTRIIVKSSLLSQAIYNCDWT--
PRPRRFKSTMRLFVERANRPLSVTGWKMFPLSLNTFTSVI--VFLYYVFFVTAC

>DpleOR45

--MAIFIFVNFIIYNSLLILNLV--
FTPRK---IEYMIREVIFLFTEVAV--TGKVLMIMFKRKKILSVFDILNC-------
EVFKGEDDISRRIVESHVSKYK-TYWSLYAILSNFAYSSQVFVPIIVYFIF---------
NTNIKLPICK-YYFLDE-
EYVRRYFVFLYLYQSLGIYGHMQYNVNIDALISGLLILAIAQVKVLKYELKNIKPD---------
----
SPLKDKRETERSQIFKLHKHLKHYEELLNYCSGIQDIVGLSMFVQFGIASIIICVVLSGLRF
T-ASVEALMFMVTYLFTMILQIYVPAWLGTQLNYESQELVFAAYSSQWI--
RRSEKFKRSLKLFMERANTNIVLTGWRIFPLSLDTFTSVI--TRNIS-------

>HmelOR61

-------------------------MVENKLSDCFKVNFIFLKIFGL-----WPGSRDSGY----
YKYYSTVFLLIILLVYNILLVFNFL--STPRK---IDLIMNEVMFLFTEVAV--
FSKVFLMLFMRQKIFDAFDILNC-------EAFKGQDDFSRQIIKNHTRKYQ-
IYWKSNAILSNFVHFSQIYIPIFQHVVF---------NKDIELPVCK-YYFLDD-
QIIRNFFVFWFVYQTFGIYAHMNYNVNVDALIAGLLVHIIAQLKVIRHEIENIKVL-----------
-
KSNKKSVQETDYLQISTLNTYLKHYEALLKYCSIVQDIIGITMFVQFGVSSVIICVVLYCLL

MP-FTIQGLMFKLAYLFAMMLQIFVPAWLGTQVEYESQKLTFAAYSCEWI--
PRSEIFKRSLKLFMERGNTSIVLTGWRIFPLSLDTFTSVS--EHSYVTYI----

>SlitOR15

----------------------------MPSDQSKMFDNSFRIAKIFGI-----WPGLKPSRY----
YKFYSFIYLFATFVCYNLLLTLNLL--YTPRK---IELLLREVIFYFTEITV--
ATKILTILFMRDKIIQALNFIDC-------DEFVGDYENKKGILYKTNMGFR-
LGWRSYLVLSNIAYSSQVIVPIFLDILR---------ETKSELPICK-YYFLSD-
EDRESHFLFWFIYQSFGMYGHMMYNVNIDSIIAGLLLIAIAQLKLLGNNLTNLKLS---------
--
DEESKLPKDSQDKIQIKRFHKLLRHYEVILRYCDTVQDTLNVTLFFQFGVASIIICVVMCG
LLLP-SSTETKVFLVMYLFTMTLQIFVPGFLGTQLTYGSEGLVTAAYNSEWI--
PRSESFKSSLKLFRERAARPVVISGLKMFPLSLITFTSIMKTAYSFFTLIRNVQ

>MsexOR23

---------------------------MSSFCQTDIFKPNFFFWKCFGI-----WGGRTENKN----
YKYYSFSYLFVTLFVFNILLTINLI--YTPLK---IESLIREVIFYFTEIAI--
TVKVLMILVMRSKILDVFNLLDC-------KEFQGDDEESKQIIETNHSFYR-
TCWKLNAVLSNISFASNVFAPLFINLIW---------TAKIEFPVCK-YYFLSD-
EMRDKYFIFWFIYQSIGIYGHMMYNVNVDSFIAGLLLMAITQLKVLNAKFTKFKLE--------
------
KRHEKHHQNKIQILRLNRYLKHYDCVLRYCEIIQDLLSVTMFVQFGMASAIICAIMCGLL
LP-STTETFMFMVTYLFAMTIQIFVPAWLGTQLSHESCGVFAA-YNCEWI--
PRSMSFKRSIMIFVERANNPIQLTGLKMFPLSLATFTSIMKTAYSFFTLFRNLQ

>BmorOR15

--------------------------MMTLVYQTDIFKPNVFFWKMFGI-----WADRKSSKT----
YKYYSFVFLFITLIMYNSLLAINLL--YTPLK---IELLIREVIFCFTEITV--
TTKVLMILFKRNKILDAFDLLNK-------NEFRGNSEESSAIIQKNNSAYK-
TYWKLYAILSNFAYSSQVLGPLIVKLIW---------KTKLELPICN-YYFLNE-
ELRHDFFSGWYIYQSFGMYGHMMYNVNIDTFISGLLMMAVTQLKIIQTKLLSLKLN-------
----
PRERKMDRGLMNITEVLKLNEILKHYELVLKYCSTVQSILDVAMFVQFGVASAIICVAM
CGLIMVRSSTETLLFMVTYLFAMTLQIFVPAWMGTQLHFQSQELVFAAYNSEWI--
PRCQSFKRSIIIFVERAKIPITITGLKMFPLSLATFTSIMKTAYSFFTLIRNMQ

>BmorOR12

-----------------------------MTRITDVFSLNFIFWKFLGL-----WGKSAPSK-----
YNMAYTVFYLFSLFVYDIFLTLNLI--HTPRK---LETLVRETMFYFNHLVA--
VTKILMMFIMRKKILVIFDLLDC-------EEFKPNDENSQEIMKRKTDFYY-
IYWRIVAVTSNLSCFMLVIGPLIKMLIW---------KIELGLPVCK-FYFMSD-
ELRNKYFVIWYIYQSFGIYNQMVNNLNLDTFNCGMLWMAVGQLQILKTKFVNLKLN----

DFENGLDLKSRDDMQIERLRKYLTHYEIILKYCAIVQDILNITIFVQLGMSSIVICVGLCGF

VAMPSNTETAIFMFSYLTTMTMQIFVPSWMGTQISFECGELMSAAYSCEWI--
PRSKLFKRSLILFVERAKTPVRITGLKIFTLSLDTFTSIMKTTYSFFTLIRQ--

>BmorOR61

----------------------------MARITDVFRLNFIFWKFLGI-----WGKSAPSKY----
NMAYTALYLSASLFVYDIFLTLNLI--HTPRK---LETLLRETMFYFNHLVA--
MTKILKMFIRRKKILVIFDLLDC-------EEFKPSDEDSQEIMKRKNEFYY-
IYWRIVAVTSNLSCFMQVVGPLIKMLIW---------KSELGLPVCK-YYFMSD-
EFRNKYFVIWYIYQSFGIYNQMVNNLNLDTFNCGMLWMAVGQLQILKTKFVNFKLN----

DIENSLDLKTRDDMQTERLRKYLTHYEIILKYCATVQDILNITIFVQLGMSSIVICVGLCGF
VAMPSNTETAIFMSSYLITMTMQIFVPSWMGTQISFECGELMSAAYCCEWI--
PRSKLFKRSLILFVERAKTPVRITGLKIFTLSLDTFTSIMKTTYSFFTLIRQLQ

>HmelOR45

------------------------MVEFSDLPQEYIKLYSICFSLIRFNDFNFFDDHVPFFK------
KYWRFFVKMFLTSIQFVSMTIYLKRRIQGD-FKIPELSYVISCYI---------
VIFQVLYNKTNIQKLI------------------------------------LSLGVMWRTDNLTDIQI-------------------
----
ENKNRFLKTFSFFCISVSGLGYMYFGIEFLTITLCAYLSTAFSLLRDDLKRLTPR------------
NNRGNLNENLRNESPYTIEMIIKQHQKLIELTKLMNNSFSVIFFTNLSFATFEICFYAFACI
VA-HTPMDFSKNILAVLIILPSIFILCYYSELLREESGGIADAAYENVWY--
RGDDGYQKSIRFIIMRAQRPCSLMSLNYTPITLNTFTKVVSTTWSYLSVAIT--

>HmelOR46

---------------------MVEFSDLPQEYIKPYSICFSLIRFNNFNFFDDHV-PI--------
FKKYWRVFVRMFLTSIQFVSMIIYLIRRIQED-FETTELSYAMSCLMVITQG--
FLKFYLVLYHKANIQKLILSLGV------------MWKKDNLTDIQIENKNR-----
LLKTLNMLAGLAY---
IYYGVELLPITICGLLSTAFSFLREDLKRLTPR------------
NNQVNLNENFQNEPTNTIEIIIKQHQKLIELTKLMMNIFTVIFFTNLAFAAIEICFYIFASIVA
-PTPMDFAKNILAVIIMLPSIFILCYYSELLREESGGIADAAYENIWY--
RGDVGYQKSICFIIMRAQRPCTLMCLNYIPITLNTFTKIVSTTWSYLSLAMT--

>SlitOR40

IIFVNLSSASISMCFFGF-
CAKVAHRALDMANNFVAVLALTLPLFNLCDYAERLREASAGMADSVYNNLWY--
ISDKHYQKSCGFILRRSQKPCCLTSLKYSEIGLNTFSAVLSTTWSYFSLASS--

>MsexOR40

-------------MTYKNRQHQELEMELSHLPGDYLKPLIACFDLLARCNIGFFHGNSSYFK------
KYWRYSYIISCVVAYYSSLTVYALKIFLGQ-MELFELAYVVPVFVVCTQA--
ILKAIIVIIHKGEIRALVLQLG-------------ETWRTDNLTTRQLNKKN----------LLLKKLNF-----
----
CYGVFRIVYSYLGTEFLLISLCSHLSTEFCLLREDLLNVKPV-------------
GNRRFRILQDSSNIELHDIVVKHQKLIKFSEQLNEIFNKMIFVNLSSVTITVCFFAFATKVA-
RGPVDMANNFMAVMALILPIFNLCYYAEMLINASAGKQESAYHSLWY--
VANKQYQMSIWFIIRRSQKPCCLTSLKFSPVALHTFTAVLSTTWSYFSLASSLF

>DpleOR39

----------------------------MDYDRTLYLTSEAFKFSGIEMGYKRIPG---------
YLKYLYHFNALWLYADVFGEFFWLCEGIAMGK--SIDLLSRVAPCSALCILS--
TAKTLPMVTNNDILKDAVLKLRRLHQGVDL-----RNSFECDELMKTSTILK-
NIITVLVVGFVCAVVLFTSMPGFLMAYD----FFTTGTYQLQLPFFT-KYFVDV-
FADARIYTFVYLHQFVSTVLVGLNIFAADTLFFAFCSYVKMHFRILGHYYENIVGQ--------
----
SESETRNNLRRCVRTHQVLIKLVNQINILYSKSTLVNIMTSSFIICLCGFNITITSKDMSTYL
LFIPFLMNSLTEIFLMCFFGDQMIDSSYNITQAVYSSRWY--
DAGEDLKRSVLLILFRSQQSCKITAANFSDLHLRSFTTVC--VYIIL-------

>HmelOR23

------------------------MSSIKGFVNSLKLTKKTFFISGIEIDKEDDTKTSIFK------
WLFHFNMLWLHTDVFGEFSWLIEGINMRKS---LADLSMVAPCTTICLLA--
TAKSLNIYLFRDVLDSAVAKLRE-----LYPSDVKEGSVESQILERSMKFLN-
IVVLFLSACCVVVTVIFSSMPLILMGYE----YYQLGYTELKLPFLT-EYFFDS-
YANKMIWSFVYLHQVWSTVIVCLNLFATDTLFYAFCAHLQMHFNFLCHDFENIVRT------
----
TDDETRRELRKCARRHQDLIELVDKMELLYSKSNLFNILSSSLLICLSGFNITLLENSKAAS
LTFIPFLVVSFTQISLMCFFGDMLMASSTKISDAIYNSRWY--
NAAPDVKRNLLFVLFRARKPCKVTAANFAVINLTAFTTILSRSWSYFALLRT--

>HmelOR12

---------------------------MSYFDKSLEIVKFVFTLSGITYKSKKLNTKEKLI------
VRSIYYFNFVSVNSDVVGAVYCFIDGVQTG-KTFVELTYIAPCITFAILC--
SLKAFCMFHYKDHVSDLIDQLRDLDN---------KRKDESDIKKRETNFLH-
AILKASNIINLMLLLTFCVSPFILIAYK----YVKTSEVELFLPLLI-KYPFDS--
YDIKYWPYVYVHQFWSVVLVIVNLSVSDYLLFICCTYLRIQFRFLQKDIREIVTS------------
--
NRKLTEPELVLLQEKIVNLVKWHQELIRLTGCLELIYAKSTLYNFVSSSVLICFTGFNVVTI
-DNVAFTTIFVVFLSTSLLQIFSLCFFGDLLMESSTEVRNAVYESKWY--
YTNTKAAKSLLIIQTRTDNPCKLTAFGFSEVNLKAFMSILSSAWSYFALLKT--

>HmelOR55

----------------------------MSNFDKSLEKIKPIFVLSGITYQSKKLNIKEKIQ----
IRCVYLFNFICLNSDLLGSIYCFIDGVRTGKT---FVELTYIAPCVTFSFLC--
NLKGIFLIHYEDRVCELIDNLRNLDNK--------RRKDDTYIKKGEINFLH-
AILKTSNILNVILVLTFIMSPFILIAIK----YLKTNKVELILPFLI-KYPFDP--
YDIRYWPFVYIHQFWS-----------DYLLYICCTYLRIQFRFLQSDIKNIIRS--------------
NKMLTEPDLAGFQEKIVNLVKWHQEVIRLTSCLELIYSKSTLYNFVSSSILICLTGFNVIAI-
QNMAFTIVFLVFLTASLLQIYLLCFFGDLLMKSSIEVRDAVYESKWY--YIKARAAKSLLII-
--------------------------QT------------

>SlitOR7

-------------------------MPKPLLFDGSLDKLGVLFRYSGMNLEKDIITPMDTIK----
HRWLYTLNYFS-VLSAEIAGIYYIITGIVQGK--SFIEVTSVAPCLTFSLLA--
LIKCSYHHMYEKNIKELINLLRHLERKENDRT---ECADKQEIIDEEAGFLN-
KVINVLYVLNCCMIVVFDMTPMVLIAVK----YYKTKQFEMLLPYLD-VFSFIP--
YELVYWPFAYVHQIWSECMVLLSMAAADYLFFTCCTYIRIQFRLLQYDFERIIPD-----------
-
RSVSKEERFDEAEFRDKFRELVQWHQDLIRSAIILDIIYSKSTLFNFLSSSLVICLTGFNVTI
V-DDIVIIVTFLTFLSMALMQVFFLCFFADLMMTASLEVSNAVYNCKWY--
LGNTQVGKQLLLVQTRAQEPCKLTAAGFADVNLNAFMRVLSSAWSYFALLQT--

>HvirOR7

-----------------------------------IEKLGVLFRFSGMNIKNKIVTPLDTIK----YRWLYTLNFLV-
VFSAIIGSVYYVILGIKQGK--NFIEVTSVAPCLTFSILS--
MIKSLYHLMYEEHIQELIELLRELELRENNRE---KCIEKEEIIASETGFLN-
KVINVLYVLNCSMIVVFDMTPMIMIAVK----YYKTNEFEMLLPYLD-VFSFIP--
YELKYWPFAYIHQIWSECVVLLDMAAADYLFFTCCTYIRVQFKLLQYDFERMIPD---------
------
RGISKGEENELRNKFTELLKWHQDIIYSSTILEIIYSKSTLFNFLSSSLVICLTGFNVTIV-
DDIVIIITFLTFLSMALMQVFFLCFFADLMMTASLEISNAVYNCRWY--
SANIKVGKQILFVQTRAQEPCKLTAAGFADVNLNAFMRVLSSAWSYFALLQTVY

>SlitOR12

------------------------MEEEPLLINKTLKKIEIWLRTTGTNVKSTPKTRMDTIK----
SRAIYIINFFWLNMDLSGAVVWFISGIANKKS---FTELTYVSPCITLSFLA--
NFKCFFLFLNEDTVDKLLETLRELEMNERSRP---RHKDKDAIMVHEHKFLT-
NLISFLNVFFCGLIVAFAIGPVTLTIFI----YVTTNEVDLQLPFLI-IYPFDA--
FQLKIWPWVYLHQIWSEVVVIVGLGAADYLFYIFCSNISVQFQLLKYNIENLIPD------------
-
DETCGRLVNVEEVRAEFVDLIKWHQDLISSVKLLETIYTRSTLLNFVSSSALICLTGFNVL
AG-SDFVYVMTFVSFLFLSALQIFFLCFFGDLLLTSGVKVSDAVYNCRWY--
LAGTSLGKDLLLVQTRSQTPCKLTAWDFSEVNLKSFMTILSTAWSYFALLQT--

>HvirOR12

-----------------------MMEEEPLLIDKTVKKVEFLFRWTGINIKSGTKTRMDTIK----
SRAVYIINFIWLNSDLAGAVVWFFAGIANSIG---FTELTYVAPCITLSFLG--
DLKSLYLIIREKNVDNLIQMLRDLEINERARP---KSEEKDAIIKYEHNFVT-
TVISVLNVFYFVLLVAFALSPVTLVALK----YYTTNELELLLPFLI-VYPFNP--
YDIRYWPWVYLRQIWSEVVVVIDICTADYLFYTFCTYIRMQFRLLKHCIERVIPE-----------
--
DDGSGRLLNIEQVRAEFVQLIKWHQDLISSANMLETVYTRSTLFNFVSSSVLICLTGFNVV
AI-SDVAFVVTFLSFLFMSLLQIFFLCFFGDLLMTASTEVSDAVYNCRWY--
LADTSFGKDLLLVQTRAQTPCKLTASDFSDVNLKAFMKILSTAWSYFALLQTLY

>MsexOR10

---------------------------MALHFDDTIKKNDFVFRYAGINIKSGERKKNDAIK----
SRRMYVFNFFWLNIDVYGSIFWFIDGLRMGKD---FIGLTYIAPCISLSTLS--
LIKSLFLISSEKHMFKLIDNLRELETRENARP---RSVQKEEIINTEIKFLN-
YVLKTINILYVVLVMTFALSPLIIIAIK----YMQTGEVELILPFLI-LYPFDP--
YNIKLWPFVYLHQFWSECVVTLNIC---------CSYIRIQFRLLQHDFERIISA--------------
PSGNRRVRENDFKAKFIELVKWHQDAIESVSLLETIYSKSILFNFMSSSLIICLTGFNITVV-
NDFAFVVTFLSFLFMGLVQVFFLCFFADLLSESSVEVSNAVYNSQWY--
MADSNIGKQLLLVQTRAQKPCKLTAWGFADVNLNSFMRVLSTAWSYFALLQT--

>BmorOR11

-------------------------MDEHSHFETSLNKIKVLFKYSGMNLENTVTNT---------
YEFLNHRWVYILNHAWTLAAVTFICIGISNGQ--NFIEMTCIAPCVAMTVLA--
VSKSFFHYINENAVKSLLENLIELERTDFERT---KSVQRTEIVATEKQLLN-
MVINVLYVLNCSMILVFDMTPLIIIAIK----YWTTNKFVRLLPYLD-IFVFVP--
YKFEYWVMAYILQIWAECIVLLFIGAADCLFFTCCTYIRIHFRLLQYDFERLTSS-------------
RRESDGLRDDEDFRETYTNLVKRHQGLIESSSILEMIYSKSTLSNFVLSSLVICLSAFNVTV
V-NDVTIVMTYLIFLAMSLMQVYFLCFF-DMLMSASEEVGNAVYNCSWY--
TEKASTGKDLLFTITRAQKPCELTAAHFAYVNLKAFMRV-SFSASITTLPTI--

>MsexOR36

--------------------------MAETLLDKSLSKINILFRCSGTNIAIGKAAPTHTKR----
NRCIYSFNFIWQNTDVLGAIYWFISGLKSGKS---FTELTFIAPCIILSFLS--
VMKSISIIIYEKKVYQLIKNLRMMEAHERNRE---NTTERGKITEKGVNFLN-
LVINVLYVFNMLVLVCFALNPLILMILN----YMKTNEIDFILPFPI-AYPFDP--
YNIKVWPMVYLRQIWTEIVVVLNTIAPDFVFCIFCSHITIQFRLLQYDIEHVITG-----------
TRKSIYNDEMRGGRIRNKLIEIIKWHQELITCVNLLENIYSVSALYNFISSSVIICLSGFNVT
EN-KDIVLVISFIAFLFMGLLQIFFFCFFGDMLIHASIEVSDAVYNSKWY--LVPPKMRKD---
---------------FTSNSNSCPTSL---------------

>HmelOR25

-------------------------------FNKALKKVQAFLRPIGLQIDENNSNMTIRKW----
LRFRWAFIVNMWMNMDITGQSAWLIQSAFTGK--SFVELTNLAPCLALCLLG--
DIEAFFFMRHSNKARELVRSIRFLQSLTAEKD---MDIEDIAFLKGQIKIFY-

IAVNTITVIITVGIMGFGVSPFINMALH----YKKTSEFVLNQPFLI-LYPFDI--
NDIRFYFIAYFHVMWTAVIAVLTVLAPCFLCYACSTFIIYQFHRLQKDLEQIIPY------------
TDNLILYEINDDVKKEFMQIIDRHRKLIECVNLMEFIYTESTLCNVITSSLLICLTGFNVMA
I-DDIALTLPFLQFLLLGLVQIYILCYYGDMIMIYSAKVGDAAYNCQWY--
MIIATRAQKPCRMTALRFSDINFKTFTRILSTSWSYFALLSS-----------

>BmorOR25

-----------------------------MFEKALRSANFYMRVIGIPTDIRDGNRTLMERL---
RNRWFYCINFLWLNTDVAGEITWFVKGLLSGS-STLIENTYLIPCLTLCILG--
NVKTFFTIKYANHIIDLVAILKDLEIKNNA-----ARKNETEIVKERLKFLT-
TSNKFLLFVIGTGIIAFGIGPLMLTASI----YFSSGDMKLKLPFLI-WYPFDS--
SDIRYWPFVYVHQVWSACIACCAVYGPDCFYFTSCTFIHIHFIHLQNDITNVIVE-----------
-
SSRARRNGLYRGCHQAFLELTNRHKDLIRCVNLLEIIYSKSTLVNVVSSSLLICVTGFNVM
AI-DFLPLIAPFTSFLALGLVQTYLLCYYGDTIMCSSTEVSDAVYNSTWY--
GTNISQMRDYLFVMKRAQKPCKLTAYGFSDVNLRTFSRILSTAWSYFALLIT--

>SlitOR9

--------------------------MVEPFQKCLSQVNFFCKLLGMNIDYRGDSETLIQFL---
RSHKYFIAQFILFNSQNVSQISWVCEAVITGK--SFLDVTYMIPCLIFCILS--
IFKGVSILYYAKYNYEFIVTMKNLLAGLTPSNEE-HNIFMKTVIDKHVLMLT-
IIFKRMVNIVAVCLIMFALGPIFIMLSN----YYKTKEVKLELPFIG-YYPFNE--
YDLRIYPFMFLHQWWTSLFATLMVCGTDYFFFTCCTFIQIQFTILNYDMEKLVND----------
-----------SSSCDKDRLKELVLRHIELMRCVDIMEKIFSTSILFNALTSSVIICITGFNVSVV-
ENVFMVGSFLAFLIFSVVEIFLYCYYGDIIMRSSMNVGNAIYNSCWY--
MTGVADRKLFLIIITRSQTPCELTAHGFAKINLYTFSSILSTAW----------

>HvirOR9

--------------------------MVDQFQKCLKSVNLYLKFIGLHLESKDTTKTFIERSR--
SHRLYFAHFFSLNLEVVAQILWVLEAVITRKS---FVEITRLIPCLILTLIS--
DFKTLSLLYYARHFIVTMKSLLLNQKQLEEK----ETRFREDLIDKHVLMLT-
SITKKISYLIGMGLLMFALAPAFIIIPH----YFKTNEVKLEMPFIA-YYPFNE--
FDSRIYPWVYLHQVWTACVAMIMVYGPDCFFFTCCTFIHIQFSLLNNDMERIVNE----------
----------DTPRYDKTKFKELAVRHIELMRCVNLLEKIFSKSILFNALTSSVIICVTGFNVLVV-
DNIVMMASFTAFLLFGLMQIFLYCYYGDTIMRSSMQVSTSIYNSPWY--
NIRAADRKGFFIVIIRAQKPCELTANGFFKINLSAFTSILSTSWSYFALLKTMY

>HmelOR48

------------------MDEATRKAAKMEIDESLMLSTFSMRRIGLSFDKPRNATDYFLQ------
KLIFSLSICGICCHVSTEFINIVLTFASSP--RVEDVVPLFHTFGYGALS--
IAKVFVLWYKNKVFGELIEELTG------IWPMPPLDGDALGIKEKSLTALR-
ISHRWYFSVNVLGVWFYNLTPIIVYFYR----VWQGQDAQVGFVWVS-WYPFDK--
HQPFAHVAVYLFEIFAGVTCVWIMVSSDLLFFGMASHIALLMRILQRRLESLAST-----------
--------EQTDEENYEEIRSNIKLHQRLIRQGESVEECLNNGFAIIKPILQLRLLEPFMA----------

-ISNKLFLGSALIQVGAMCWYADDIFHANGNIAAAVYSSDWY--
KMSPRCRRALLFLILRCQKPIAFTAMKFTNISLVTYSSILTRSYSYFALLYT--

>MsexOR12

-----------------------MEQAKREIDESLKLSAFCMRRIGLSFEKHKNASAHLR-------
QQLMFALSVCSICYHVFSEIMYIGLTLANS-PRVEDVVPLFHTFGYGALS--
IAKVFALWYKKDVFSQLLRELVG------IWPTPPLEDEAQAIKDKSLDALR-
ITHKWYFAVNVLGVWFYNVTPIAVYFYR----LWQDGDAQVGYVWVS-WYPFDK--
HQTIAHVAVYIFEIFAGQTCVWIMVSTDLLLSGMASHISMLLRMLKRRLESLAST---------
---------EKTDDEYYHEILENIKLHQRLITYCYDLEDAFSLSNLVNIVLSSLNICCVVFVIVLL-
-EPFVAVSNKLFLGSALIQIGMLCWYADDIFHANADVAAAAYNSGWY--
STNARCRRALLFLMQRAQKPIAFTAMKFTNISLVTYSAILTRSYSYFALLYT--

>CpomOR12

--------------------MNTNAEARREIGATLTLCTFSMQCIGLSFERPDG-TARLLR------
QKLMFVVSVCTIVYHVFSEIVYIGLTLSNS-PRVEDVVPLFHTFGYGALS--
IAKVFALWSKKNVFTEHLDELSG------IWPMEPLDEDARNIKEKSLTALR-
LVHQWYFSINVGGVLFYNVTPICVYMYQ----LWQGQDAVVGFVWMS-WYPFDK--
YKPINHVFVYIFEVFAGQTCVWIMICTDLLFSGLASHIAMLLRLLHKRLETLAET-----------
-------EKSQEEYYQEIVANIKLHQRLIRYCNDLEEAFTIVNLINVVFSSLNICCVVFVIVLL--
EPFMAVSNKLFLGSALIQIGMLCWYADDIFHSNADVALAVYNSGWY--
RTDPRCRRALIFLIRRAQKPVAFTAMKFTNLSLVTYSSILTRSYSYFALLYT--

>HmelOR49

------------------MVTQDKIIAKKEINKTLKLCNFCTRIVGFSFIDDLPKSL--------
YLKIWKYICFLVIIVLKEAGQMNYAITELLTS-TSVADFVAGLHIFGYDCIS--
LSKLLTIWYKRNIFRNLLSELSD------LWFLTSSDRNAIEFKKKKLSKLE-
IVQFWYAFWNVLGVWLYNLTPVVIYLYQ----KIRGEPASLGYIWHL-KYFFDK--
TQPGYHELAFVFETFSGLFSVWAMLGSDILFMTMASHISMLLRMLQDKIRRLDMR--------
------------KNQNCYADITSLIKIHQRLIRYGEDLEDAFCLVNLINVLMSSVNICCL--------
LDPVMVMSNKFYLGAVLTQVGILCWYADDISASSSGVATAVYESGWY--
HCDIRSRKLLLAVIQRSQKPLYFTALKFGPITMSTYSSILATSYSYFALLYT--

>BmorOR51_BAH66346

--------------------MDCTIVAFYSQAKTQIKMLRYDLEQL-GK----------
FERSSHIWKALKDEKIHSKLVFCVEHYRQIVWFVKEVESIFGEAMTVQFFVMAWVICMT
VYKIVGLSIYSAEFVSMGVYLGCMLAQLFIYCYYGTQLKVESESVNTSLYCSNWL--
SSLPKVRRQMLIMMQYCSKPLTPRTAYVIPMSLETYISVLKSSYSLFTLLNQKH

>DpOR46_gi|357617181|gb|EHJ70629.1|

MSVNQILRDFNNNTDTSSEILAKSSINVISKRVYPRQPADVGYGQIMNGMDLRLQKTTST
FCSTHSNGDDVLKMFDSAAAEYQIHNVHLDILSAEFLTMGTYLCCMMTQLYIYCYYGT
QLKVESELVNDSVYQSSWL--
SLSPAFRRGLLVMMERCKRSIEPRTAYIIPLSMETYISATSRLFSAPNVVSAI-

>Bany_OR3_GZXD38A02GNYNJ

DHYDRVVWFAKEVESIFGEAMIVQFFVMVWVICMTTYKIVGLSLMSAEFVSIVLYLGCM
LAQLFIYCYYGTHLKVESELVNDSIYQSDWQ--
SLSPAFRRQLFVLMERCKRTVEPRTAYIIPMSLDTYISVLRSSYALFTILDRK-

>DpleOR62

--------------------MTFKFLSNLENPDHLLLGP----TLWGLQTSGIWQPCKKRVK------
IIYNTIHIM-AILFVLSQYVELY--FIRND---LAMALKNLSVTMLSTVC--
VVKAGTFIIWQKYWKDIFNYVSK----LEKNQLYKPDYLTKNTIRKYTEYSR-
FVTYFYWTLVTATVFSLILAPLASYLSSQNKELGDSGASSYPEIMSS-WVPFDK--
TQGCGYLVLTLIHVLICFYGGGIVANYDSNAVVLMCFFAGQLKILKINCERLFGN----------
------
GEEIVSYKEAVERIRICHHHHILLIKYSKVFNSLLSPVMFLYIIICSLMICASAVQLTTAGTT
SMQQIWIAEYLIALVAQLFLYCWHSHKVLHMSLQVDSGVYTSAWW--
SQDVRIRRSVLLLGGQLKKKVMFTAGPFTDLNIATFVAILKGSYSYYTLLSK--

>SlitOR42

-------------------KWYFKFLTVSEDPDNPIIGN------YYLIASMWWSLGERSFX----
XKLFSHNLVHFVAFIFVLSQYVELW--VIRND---LEMAMRNLSLTMLSTVC--
VFKACNLVFWQNTWKELFDYVSE-----------LKDDTINKIIFQYVKYAR-
RVTYLYWSLVTATVLIVSLAPLLIYWSSPTYRHNIRNGTVPYPEIMSSWTPFDR--
TRGIGFCVATVY---------------------IMTFFAGQLKVLSANCSRLFGD----------------
GNELINCDETVKRIKECHLHHLYLIKFSAVLNSLLSPVMFLYVIICSLMICASAAQLTTEGT
TTVHQVWIAEYLMALIAQLFLYCWHSNDVFYLSNQVDDGVYSSAWW--
SQNVRIRRSLLLLGGQLRKPIVFTAGPFTKLNMATFVTILKGSYSYYTLVAKKE

>HmelOR27

---------------------MVILFPSLEDPECPLLGP----TLWGLQTWGMWQPNKGKAK------
IVYNTIHIF-AIIFVMSQYIELW--YIRSN---LEMALRNLSVTMLSTVC--
VVKATTFISWQKSWREIFEFVSL----VEKQQLNKQSKKTKRIVNEYTKYSR-
AVTYFYWCLVTATVFTVILAPLVLYFSSQRREQIRNGTLAYPEIMSS-WTPFDK--
TRGLGYWTIVVFHSLICFYGGGIVATYDSNAVVLMSFFAGQFEFLREKCADLFGN---------
----

GEEFISYNTAVKRIKECHHHHVLLIKYSKILNSLLSPVMFLYVIICSLMICASAVQLTSEGT
TTMQQVWIAEYLVALVAQLFLYCWHSNKVFLK---VDEGVYTSAWW----
SSRGPHTQYSAAWRST--------------------E--------------

>MsexOR43

--------------------MLERLSKYLENPNHPLLGP----TLCGLKCWGMWQPL-GVNR------
IIYNAIHFF-AILFVISQYVELW--FIRSN---MELAIRNLSVTMLSTVC--
VIKAGTFVFWQKSWNDVIDYVSG----LENIQLSKRDRITNSVISEYTKYSR-
SITYSYWVLVTATVFTVILAPLVGFLSSSDKDLMLNGTLAYPEIMSS-RLPFNR--
SRGFGYWVAAIEHSLICFYGGGVVANYDSNAIVLMSFFAGQLKLLSINCARLFND---------
----
NEVLKYSDTMKRIKDCHHHHVEIVKFSMVLNSLLSPVMFLYVIICSLMICASAIQLTAEG
TSNMQRIWISEYLMALIAQLFIYCWHSNEVLHMSSKVDEGVYPSHWS--
AQNVRVRRSVAPPRRATAQAHRLHSRTLYQAHYIYFCSYSEGIVQLLYAAKQ--

>BmorOR27

-------------------MPSSFFLPNLENPDYPSLGP----TLKGLKYWGMWQSGGIKR-------
ILYNSIHAF-ATFFVITQYVELW--IIRNN---VELALRNLSVTMLSTVC--
VVKAGTFVCWQKYWSGIIGFVSN----LEKEQLSKNDAATQAAIVKYIKYSR-
RVTYFYWSLVTATVFTVILAPLVGFLSSPERELIANGTLPYPEIMSS-WVPFDR--
SRGFGYWVTALVHTLICFYGGGVVANYDSNAVVLMSFFAGQMKLLSINCSRLFDD-------
---------GNEVISNNEAMKRIKECHYHHVFST----
IFNSLMSPVLFLYVIICSLMLCASAVQLTTDGTSNMQRIWISEYLMALIAQLFLYCWHSN
QVLYMALERLGGLFEACLE--
RFPSKWKTGRLVLLRKDGRPADSPAGYRPIVLLDEAGKMLERIVAARIVRHL--

>CpomOR28

---------------MVSVQKIISLAKRLEDPKHPLLGP----NLKGLYVYGLWQSGSKFRN------
TCYNVIHFC-AFLFVISQLIELW--IIRHD---YLEALHNLSLTALGMVC--
IFKAVSYVMWQSDWKKLVEGISA----EEISQSDSLNDACIELKQKYTNYVR-
IVTYLYWNVVVSTNITMVSAPFLKYATSEYREQISNGTEPLPQIFSS-WFPFDK--
TTMPGYSLAIFIHILINIHGGGVIALYDSNAVAVMVFIRGQLGMLREKCKHIFDE------------
-----
YELVNQEIILGRIKECHRHHNFIMRHSSLFNSLLSPVMFLYVLVCSGMICCSVIQFTSEEAT
AAQKVWVLQYTTALVSQLFLYCWHSNEVVVECQHVDGGVYDSEWW--
KGDTHVRKQLAMLGGKLTHNIVFSAGPFTTLCVPTFIDVIKGSYSFFTLLTQ--

>DpleOR63

-----------------MASKIIRIFERLESPKYPLLGP----NLQGLYWFGLWQCGNRIRD------
GLFNILHLA-SVLFVLSEFVELF--AMEID---LMKILFNVSVTALSLVT--
VCKTVLFIYYLPHWKNLVNSISK----LEQEQLKSNNFKLVAIIKRYTLYSR-
VITYSFWSVICITSLLTVTAPFLKYITSSYRQSIQNGTELYPQILSS-WFPFDK--
TKMPGYLIAVSIHIIMTTQGAAIVAVYDSTAVAIMSFLKGQQILLRYKCERIFGL-------------
----

NEVIPTEKVLANIEECHRLHCFLLEQHHRFNSITSPVMILYVLVCSVMMCCSVVQLSLGH
LSTSEKLWVIEFTTALITQLFLYCWHSNEITYESNLVDRGVYASNWW--
RGDVKVKRQILILAGKLAPSLILKAGPVTTLSMATFISILKGSYSFYTLVTQ--

>HmelOR53

----------------MLLQKIIRTLSSLENPEYPLLGP----NITGLYWFGLWQCGRKYRD------
AFFNFVHFC-SLLFVISQFLELY--FMEKD---LLKVLFNVTITALGLVS--
ISKTLFFILFLKDWKLLIKNISA----EEIHALSVGNKNVVNYMNQYKSYSR-
NLTYLYWCIMLMTSFVIMLSPYLKLLSNNYLELVKNGTEPYPQMLSS-WFPFDD--
TKMPGYFISVTVHILIAIQGIGVIAVYDSNAVAIMSFLKGQMQILKYKCENIFGD------------
---
DYEVISQAEVISNIKECHRLHNFFIEQHKLFNTILSPVMFIYVLVCSIMICCSVVQLSVEGV
PISQKFWVIEYTTALIIQLLLYCWHSNEIAYESASVDLGVYTSNWW--
RGDVRLRRHALLLAGKLSPMFILDAGPFNQFSMTTFINVSHR-----------

>BmorOR53

----------------MALKKMLALTKGLEDPTHPLLGP----TLKALSVFGLWQTGSQKST------
VIYNTFHFL-TFLFVITEYIDLY--TVRKE---LSKMLNNLSVTVLSTIC--
MIKTLSYVCRQSHLKVLVREISE----LELELMKTTDKNIVKRLRQYTVYTR-
AVTYVYWFLVVGINVVLLTSPLLKYASSIYRSEIKNGTEPPPLILCS-WFPFDS--
ARMPGYFWATMVHIIMSIQGCGVVATYDMNAVAVMSYLKGQTSILKDKCKAIFDE------
----
TASSRDVLNRIRDCHRHHNILLRHYYMFNSLLSPIMFVYMLICSFTICCSIIQLDSSETTISQ
RIWIIQYSIGQISQLFLYCWHSNEFAA-----------------KVKKKHFLF----------------------------
-PINLF-------

>DpleOR61

-------------------MLKALLSSIEDPTRRLLGPNYWILKNMGL--------VLPQNP----
IAKVAYIVLHEIVSFFVLTQYMELY--VIRSN---LDLVLTNLKISMLSIVC--
IVKANTFVIWQVQWKEVIEYVTR----ADQYERDNQSVERRKIIDSYTKYCR-
GIVYFYCVLVFTTFITVTNTPLMRYLSSQYREALRNGTELFPHIFSS-WMPFDK--
YNSPGSWITVIWHVCLCAYGAGIMAAYDTVIMVIMVFFGGKLELLRERCTHIFEN---------
----
TEIASDDNQTNEKIRKLHNIHVLLMKHSRLFNSLLSPVMFLYVVMCSLMLCASAFQLTSA
-TNTTQKLLMAEYLVFGIAQLFMFCWHSNDVLIKSQKVMFGPYESEWW--
TVNSKQARSILILT--

>HmelOR28

--------------------MIQSFITSIEDPQRPLLGPYWILKKMGLVLSKNL------------
LNKAFYIIVHEIVTVFVITQYIEAY--VIKSD---LDQVLTNLKISMLSIVC--
VVKANTFVFWQHSWGEVIEYVTQ----ADKYEREDTNVEKTQILDSYTEYCR-
RVIRFYWTLVFITFISVISTPLIKYLSS--QEAFNNGTELFPHIFSS-WVPFDK--
YSFPGNWITVICHVLFCAYGGGIMAAYDTIIIVIMVFFGGKLQLLRERCKAIFSC------------
---

NGLTVSDEQAYRTIRELHKTHVLLMKYSRLFNSLLSPVMFLYVVMCSLMLCASAFQLTT
E-TNTTQKVLMAEYLIFGVAQLFMFCWHSNDVLVKNQQVTLGPYESEWW--
KMDFRQRKNILILAGQLKIVHTYTAGPFTELTLSTFVTVY-------------

>SlitOR29

---------------------MNSFLQSLEDPARPFLGPYWILKKMGL--------LLPKSI----
LGKVLYIFLHETVTFFVATQYMELY--VIRSD---LDLVLTNMKISMLSVVC--
IVKVNTFVLWQKHWREVLDYITE----ADNFERQNEDPVKSQIVEAYTKYCR-
RLTYFYWALVSTTFLTTTCSPLMRYLSSMFRENMRNGTEPFPHIFSS-WMPFDK--
YHSPGCWITVLWHTVLCAYGAAIMAAYDTCIVVTMVFFGGKLDLLRERCKHMFGS------
----
YGTVTTDKQCEEVVRQLHGIHVMLIKYSRLFNSLLSPVMFFYMVMCSLMLCASAYQLTS
A-QNAAQKLLMAEYLIFGIAQLFVFCWHSNDVLIKNENMTSGPFESNWY--
TANYRQKKDVLLLSGQLRIKNIFTAGPFANLTLPTFINILKGAYSYYTLLRK--

>MsexOR18

---------------------MLSFLKNLEDSERPLLGPYWILKKMGLLLPKNK------------
ISKIFYILVHEIVTLFVVSQYMELY--VIRSD---LDLVLTNLKISMLSVVC--
VVKVNTFVFWQSDWKQVLEYVTK----ADMFERQNQDVPKSNIINSYTKYCR-
RLTHFYWGLVFTTFLTTTNTPFMRYLSSNFRNNFANGTEMFPHIFSS-WMPFNK--
NHSPGCWITILWHVLLCAYGAAIMAAYDTCVVVIMVFFGGKLYLLRERCSNMFKN-------
----
SDENASEATVKQLHGIHIQLIKYSRLFNSLLSPVMFFYMVMCSLMLCASAYQLTSA-
TNAAQKLLMAEYLVFGVAQLFLFCWHSNDVLIKNENVTLGPYESNWW--
SASLRQKKNVLILSGQLRISNKFSAGPFADLTLATFITIIKGAYSYYTLLRK--

>BmorOR29

---------------------MFDFLQNLEDSERPLLGPFWLINKTGL--------LLPKTN----
FGKLAYILVHEIVTFFVVTQYVELY--VIRSD---LDLVLTNLKISMLSIVC--
IVKVNTFVFWQTSWREVLEYVNE----ADKFERNQTDETRGKMIETYTKYCR-
RLTYFYWSLVFTTFLTTTNTPLMRYWSSIFRENLRNGTEDFPHIFSS-WMPFDK--
NHSPGSYCTIVWHVLLCAYGAAIMAAYDTCIVVIMVFFGEKLNLLRERCKKMLAN-------
----
DLYNHAFVIGQLHDIHVQLIKQSRLFNSLLSPVMFLYILMCSLMLCASAYQLTSA-
TSTAQKLLMAEYLIFGIAQLFVFCWHGNDVLFKNANVSLGPYESNWW--
SSSPRVRADVLLLCGQLRVRHVFTAGPFADLTLSTFIKILKGAYSYYTLLRK--

>BmorOR56

----------------------MKLLEKLEDPDRPLLGP-------NVKALKFWGLLLPESR----
SKKYFYLFMHFAVTVFTATEYIDVW--FVKSD---LALLLNNLKITMLATVS--
VLKVTTFLLWQNAWRDLIGYVSR----ADLEQRATSDSRKLALINGFTGYCR-
KITYYYWFLMYTTVAIVTVQPIFKFFSSAYRLDVQSGNGTYLQVVSS-WIPWDK--
NTLPGYLLASIYQTYAAIYGGGWITSFDTNAIVIMVFFRAELELLRIDCAALFDD------------
-----

EKSFGDMAFMRRLKECHRRHTELVKHSRLFDSCLSPIMLLYMFVCSVMLCVTAYQITIE-
TNPMERFLMTEYLVFGVAQLFMYCWHSNDVLYASQDLSRGPYESAWW--
SRDVKYRKNLYILVAQFNKVIVFSAGPFTKLTVATFIRILKGAYSYYTLLSQSQ

>MsexOR30

----------------------------LENAERPLLGP-------NVKALQFWGLLLPKYR----
LKRYFYLTMHILVTLFTFTECVDVY--FVKSD---MNLLLNNLKITMLATVS--
VCKVNTFLYWQKRWTDIIAYVTR----ADLNQRNTKDEKKLALIKKFTLYCR-
KITYLYWFLMYTTVVIVVAQPIFKYAFSNYRDNVRMGTETYLQVVSS-WVPFDK--
NTIIGYIAASIYQTYAAKPNS-------------------------ADIHHTGGR-------------------------
SSMHLEGPIRPIVSR---
ITIHWP--SFNVVTGKT---------------------LALPNLIALI--------------

>DpleOR60

-----------------MTSTIQRLLQKLEDPKRPLLGPIKWLKLYGLILPRTRKG------------
KMIYLSILTVMGLFIISEYVDMI--IMRND---VNHLLLNFKYSMLSSVN--
VIKVITMYAWHKHWMGIFKFTTR----VDIERRNSPDPIYRETVRKYTKYCR-
IITYIYWFLGYCTVIMIVVQPAAKYLLSTYRQNVKDGTEKFLEVVNS-WTPFDK-----------
TTMSGVASLYSGGWITSYDTNAFVLMIFFRGELELLRRDCRSLFGT----------------
EENPVTKEEAQKRIDHCHQRHNDILKYFRLFDSCLSPIMLLYVIVCSVMLCATTVQLTTE-
SSAMQKLITFQYLMFGVSQVFIYCWHSNDVFYVSQDLMLGPYESYWW--
TRTLSEQKNLHLLAGQLNKQIVLTAGPFSYINLATFINILKGAYSYYTLLN---

>HmelOR57

-----------------MKLFIQKLFNVLEDPNQPFVGRIKLLKIFGLLLPENNKK----------
LKLIYFIFHAY-VLMFIVTQWMELF--TSRSE---IIMILRNVKYAIFASVN--
VTKVSTCILWQKDWKFILDFITK----VDEERRTTKDKVSKDILQKYTFQSK-
IVTYLYFYLTYCTIAMMIIQPTVQFSLSSSYREKLKSGKEKPLEVVNSWLPVDK--
RTSSGYIIMIICQMLPLIYSGGWITSYDTNAIAVMTFVCCELELLRRDCQILFGT--------------
--
IKEPASNEEAKAKLAKCHKRHNDLVICSQLFDSCLSSVMLVYVVICSCMLCATAYQFMIE
-NEPLQKIITAVYLIFAVSQLFLYCWHSNEAYYISKDLMLGPYESIWW--
MRSLSEQRNIHILTNQLDKIIVFSAGPFSSITVTTFINILKGAYSYYTLLSQSQ

>HmelOR58

-----------------MASLLTKFHSALEDSNYPLIGR--NVKLLKMSYLLSERPGYRAAV------
RVKAGILLSLMVFFALTQATDTF--INISN---IVKFLRNLKYVLMSTMI--
IAKVSSYMLWQKRWETIIQYVNE----VNVDRLITDDPISIKIYKKLVKYCK-
IVTYIYIYLTSATVFLVLIQPILIYCLR--MYSSLEDDLDKYIPITNTWVPFDK--
DNLIVYLLSLLLQIYATIYSLIYIVAFDANAVSTMVFFRGELELLRRDSQILFGT--------------
---
NSPVNEETARVNLSKCQKRHKDLVTYVKLFDSCLSPIMLLYVIVCSVMLCATAYQLTVE-
TNAMQKFITAEYLIFGVSQLFMFCWHSNEVLY-SKDLTLGPYESMWW--
TRSVSEQKDISILTDQFNKEIVFSAGPFTSITVATFISVS-------------

>SlitOR45

QFQYFRVYRSFQKCRTNVRLGNLLKFLEDPEYPSVGPHLKLRQTLIGRFKQILFYVTITFF
FSQYVKCFIKFN-ADSLKLILQYAPFHMGIV-----KSCFFQKDYKTWELVIDYMSS----
VEKKQLDKKDKKHDDIIHEYIKRNR-KVSYFFWALAFFSNFSIFTEPYQKNQVN------
VNGTSIYLKIFDG-YTPFDN---
EPPGYYFSMLIQTVLGHIVSAYVVGWDTLVVSIMIFFTGQLKITCMYCRLAIDA------------
----
NSTKSHENIAKCHRFYTTLVEYTHLFNALVSPVMFMYLVVIAINLGVCIVQIVEIHDDITT
LVSSILFVIACLIQLLLFYWFANEVTVESTFVSYSTFESDWP--EANKKLQKE------------------
------------V-------ALLG---

>MsexOR35

-----------------MVVIEKILSFLEDPRYPSVGPIRLLSLTGL-----WH-PDLKSP----K-
SRFKIIIFLVTVAFFLSQYVKCIIKFNTDD---LKLILQYAPSHMGII------
KTCLFQKDYKIWEELIDFISS----VELKQISRKDENLDKVMKAYIRRNR-
RVSYFFWALAFFSNFSIFTEPYQKNNFN------VNGTSTYLYIFDG-YTPFSR---
EPNGYYFSMCIQTMLGHIVSAYVIAWDTLVVSIMIFFAGQLKITRLYCTRMITA------------
----
NNEESHRNIAECHRFHTSLVKYQKTFNCLISSVMFVYLVVISVNLGVCIKQIAEIEDDLPT
LVSSCVFLMACLIQLLLFYWHSNEVTIESDLVSYSTFQSNWA--
QSNKNIQKEVALLALTTRKKLVFRAGPFNVMSLSTFVSILRASYSFYTLLKG--

>BmorOR36

------------MVFNSKKNIISLFSLLEDSRHPSVGPHLRLLSLTGIWYPNSKT-----------
NITLLKRACFYVIVLFFVSQYLKCIIKFKIDS---LQLILEYAPFHMGIV------
KTCFFQKDYNVWQDLVSFISK----TERDQIAKKDPKSIKTIQSYISRNR-
KITYSFWALAFIANIGVFSKPYQNNQSD------VNGTVTYNHLFDG-YTPFSE---
EPPGYYFSMGIETILGHVVSFYVLGWDTLVVSIMIFFAGQMQMSRLQCSRMING-----------
----
SPERTHKNIIKCHKFHTDLIKYQKQFNSLISPVMFVYLFVSSINLSVCIVQIAEIEDDFATVL
SSFIFLLACLIQLLLFYWHSNEVTVQSELVSYSTFESNWT--
STQNKLQKEVALLGLTTSKTLVFTAGSFNHMTLATFISIIRASYSFYALLNSTK

>DpleOR48

------------------------MSLLFTHIKSSLKFTNVIFTVFGVWPPPGLNDLARRVY------
SLYSFLLLFMMSVYIIIQTGDLV--QVWGD---LTLMTASVFMLFTNIAY--
AIKLAYVIQRRELIQKIIFDG--------------DNELDGQKTKLAIQTVE-------------------------------
-----------------RYPFDA--
TKRPAYQWTYAHQFIALSINAAVNLCCDVLVATLIAQCRCRLRLIAQSLRTLCDD---------
VDVDKKGRITADSEKVVSSRVRGIVMRHQLVLAQVEQLQQCFSVPILGQFAVASFIICVT
AYQMAFV-TNLYRLISMASFEIAVTIQVYIYCVAGTNLTIDSDEVSRAAYECPWY--
EFPASIRRLLLVIMVRCKRATVITVAGIVEISLDTFMSLTSTCVKTLTDLHSQI

>DpleOR49

-------------------ISVLMGNVLEEATAKDIKNC---
TFIILQMIDLY--LIWGN---LSLMTGTALVLFTNISQ--
VTKIINLLARRKHIEFLIKDTNDGLEVM-------TSSRARELIKSCDHETR-
NQQVIFLLVTFVTTVGW---ATITDKNG--------------LPLPA-WYPYDT--
SVSPAYELTYTHQTLSIMIVAVLNISKDSLVTTLLAQTRCRLGLVGLALTTVCSDE----
DNTYSGGYSNKLFTAQQEMIAKNRLRICVQQHQRALRAAKILQECFSVPTFAQFSVSLIII
CVTAFQLSVICIDGQQDAWPTPVTFFIIINFSAYSDIGYYHKKTSEELARRAYEMPWY--
MCSVSVRRSIQIMMIRCHRIVKFTAGGFTTLSLATFTAVIKLSYSMLTLLQQ--

>HmelOR62

------------------------MGILVENSIFSLRLSLTALRFTGFWPPEGWSKGKLILY------
HCYGFFCFMLLGIFLITEVADLF--LIWGN---LPLMTGTAFLLFTNLAQ--
AAKFLNIIIRRKSILRIITECEV---
LKEHKVAKFKGSRNILCIVQSCNRETLQQQVFFFILTILTVAGW-----------------
LGSAEMGKLPLRA-WYPYNT--
NISPLYEITYIQQGFTILLAALLNVSKDTLATTLIAQCKCRLRLIGLSLRNIGDC---------
YYGEVNEPLTSSQELKAYQHLRRCVIEHQRTLNTIMLLEKTFSEPTFAQFSVSLVIICATAF
QLVSQSGNYVRLISMGTYLFNMMFQVFIYCYQGHSLSTESEDLSACVYDMPWY--
NYSVRVRRSLLVIMVRCRRIAKLTAGGFTTLSITSFMAV--------RLML---

>HmelOR63

------------------------MDILVKNSNFSLSLSLTILRFTGF-----WPPETLRKDKMI-
LYNCYGFIFFVLMVLFLISELGDLV--LIWGN---LPLMTGTAFVLFTNLAH--
ATKFINIVVRRESVLKIITDSDDILKAE-------TSVEGRKIVHSCNRETR-
WQQLFYFLLTFFTCMGW-----------------ASSAEKNKLPLRG-WYPYNT--
NKPIAYEITYFYQVFSMFGAAFLNVSKDTLVTTLIAQCRCRLRLVSLSLRNLTSL---------
TVSEKTLIFTPTQEAIVYKRLRSCAIKHQKVLETTLLLQNTFSEPIFAQFNVSLVIICVTAFQ
LVSQTGNYIRLISMSTYLMNMMFQVFLYCYQGDQLSTESEELAKAAYEVPWY--
ASSVRLRSSLLIMMVRCRRVVKLTAAGFTTLSLNTFTAV---RFFELVGLFS--

>BmorOR63

------------------------MKLWIRNANFTISLSLTLLRCLGFWSPDGLAGNKRLL-----
YNCYSFVFFMFLLGIYILIQVVDMI--KIWGD---LPLMTGTAFLLFTNFAH--
ATKVINIVIRKNRIQRVIQQANAVLMGV-------QSEEARRIVKSCDFETS-
IQLCLYFLLTFVTTVGW-----------------ATSAEKHQLPLRA-WYPYDT--
SKSPAYELTYIHQVAALLIAAYINVAKDSLVSSLIAQCRCRLRLVGLALASLGQD---------
LKIDYQSQLSPAQENILNLRLKTCVLEHQTVLAAVTELQACFSKPTFAQFTVSLIIICVTAF
QLVST-GNLVRLLSMGTYLMNMIFQVFIYCYQGNKLSVESSEIAGSVYFSPWY--
LGSVKLRRALLIVMVRSRRVAKLTAGGFTTLSLASFMAIIKASYSLFTLLQQVK

>CpomOR16

-----------------------MGIIEKNVNFSVSVSITALKLFGFWAPEGLTREQNILY------
NCYGFLSFMLLGTYLIIQVVDLF--LIWGD---VALMTGTAFVLFTNLAQ--
TTKIVAVVARAGTLRPLVSSADT--------LLAAETGPGKEIVDSCNRETW-
QQQLVYFCLTTVTVAGWAGSAEKNQ-----------------LPLRA-WYPYDT--
SVSPAYELTYLHQVGALFAAAYLNVGKDTLVTCLIAQTRCQLQLAALRLASLTDD--------
-
LTPTAQGTLTAEQEAEAWSRLEVAARRHQEALHAAAQLQACFSAPVFAQFGVSMLIICV
TAFQLTAQNGNLVRLASMGTYLLNMMFQVFIYCYQGNQLSGESMDIATAAYSCGWT--
ACGVRLRRGLLLVMVRARRAARLSAGGFATLSLASFMAIVKTSYSLFTVLQQAD

>MsexOR16

-----------------------MGIFVQNVNRSLRFCLTMLKLTGFLVPNGVIEFTVFHN------
IYWFFWMMFVVGINVITQTGDLI--QVWGN---LSLMTSAAFLLLSDVAM--
MMKIINVVMRGRVIQTVIDGMDL-------ELRSEARAKGRKIIKECDDQTT-
RHLYLFLCLSGVTVLGW-----AGSAEH------------NKLPLRA-WYPYDT--
STSPAYELTYIQQ--------------------------------NQLITTGQN-----------------------
TIDLRLRSCVLKHQAALRAASQIQECFSEPILTQFTASTVIICVTAYQLRIEQSNLVRVISM
MAYLLCMMLQVFLYCLQGNQLAEESSNIAEAVYECPWY--
RLPLPLRRSLLLIMVRSRRVAQLTAGGIATLSLACFTSIIKVSYTFFTVLQS--

>MsexOR34

-----------------------MKIFIDNANGTLWLSLNLLRWVGFLVPDNFEGRKKKLL------
PFYSFFWFMIVGIYVIVQTGDLI--QVWGD---ITLMTGTSFLLFTNMAF--
ITKIINVMVRRDAVLAIIDEGDEVLRSE-------RRIEGKAIVKSSNQETS-
RLLYLYGLLTVVTVFGWAASAEKGS-----------------LPLRA-WYPYDT--
SKSPAYELTYLHQSVAVILLAFLNVSLDVLVTSLAAVCRCRFQLLALSLRTLCHD---------
IPIDEKHLVSPEHKQIVHERLRLCVLQHQSILESAAKIKTCFSGHILAQFTVSIVIICVTAYT
LALERDNPIRLIAMFSYLLGMMMQVFLYCYQGDYLSEESSDIADAAYECPWY--
ACPIPLRRSLLVIMARSCRVAILTAGGFSTLSLACFVSIIKASYSFFTVLQTSR

>MsexOR37

-----------------------MKIFIHNANGTLWLSLNLLMWVGFLVPYNFECQKNNL-----
LPLYSFFWFMFIVGIYGSFRTRNLI--QVWGD---ITLMTGTSFLLVTNMAF--
ITKIINEMVPRDAGLSIFYEGDEVLRSQ-------RRIEGKAIVKSSNRETS-
RLLYLYGLLTVVTVFGW--------AAS---------AEKGSLPLRA-WYPYDT--
SKSPAYELTYLHQSVAVILLAFLNVSLDVLVTSLAAVCRCRFQLLALSLRTLCHD---------
IPIDEKHLVSPEHKQIVHERLRLCVLQHQSILESAAKIKTCFSGHILAQFTVSIVIICVTAYT
LALEHDNPIRLIAMFSYLLGMMMQVFLYCYQGDYLSEESSDIADAAYECPWY--
ACPIPLRRSLLVIMARSRRVAILTAGGFSTLSLACFVSIIKASYSFFTVLQQVE

>HmelOR64

------------------------MKLVQNVNFSLIVTQVVFFTFGIWTPKCDG------------
WKKRLFSFYSFVSFNFILGSYII--IQIGD---LIQVWGNLSLMTGTIFL----
LLTNFSLSTKCNRETI---------------------------------MQLSVFYTLSYMTVFGW----------------

AASAEKNELPLRA-WYPYDT--
SKTPAYQLTYAHQSIAVSMEATINVCLDTLVTSLICQYRCQLRLLALSLRTLCDG---------
LEFDNMGRLTEYSNEIVSRRLERCVVRHQTVLHQVNLLQKYFSIPILAQFTVSMGIICVSA
YQLAFETTSLVRIISMVAYLMVMMLQVFLYCYQGNQLVMDSTGVSSAGFDFPWY--
ACSIPIQKSLLILMMRTKRVARLTAGGFTELSLATFMAV-STYYILN-------

>BmorOR45

-------------------------MKVLDNVNHAVKVTMNCCRLYGLFVSDDLTKRQLIIM------
RAFSLMLYLFFVGFFITTQSALII-TMWGD---LNLMTNVGLVLGTHLTL--
SAKVFTLHYKEKEITNVIYKNEVRLRAE-------TREQGKYIISEMNRETT-
LFMRLFIPFGMGTVTAWLLCTPKGELYT-----------------PA-WYPCNT--
TKSPAHEIILAHQGIAVILTATLEIAIVLLMTSIVAVCRCRLKLVGLSFETICDD---------
LPSNIMNKLTADEQVIVAKRVRENVIEHQAVLECINDIQDCFSSAMLVHIAISTMIICATA
YQLAVESLDLTQRMTMASFLGGMSTEIFLFCYQGGHLSIDSMEVATAVYSCPWY--
TFPTSLKRSLLVIMIRAQQPALLTAGGFAPLLLDTFVSIMKASYSFFTVLQN--

>BmorOR47

------------------------MKLVFDNFIFALKVTLNWCRYFGIFIPDELTGRRQKL-----
LVQAYSVFMFMLFIGFFIITQIILFI-LVWGD---LSLMTDVGLVLGTNLAL--
SAKIAVFFFKREELASILKKNDDTLRFE-------TREEGKKIISEIDRETN-
AFMKVFFCFGVGTVIAW----FLSTPKG-------------ELHIAT-WYPCDT--KRSPAYEIIMIHQ---
------LAITADLLMLSMIAVCRCRVKLVGLYLQTICDD---------
LPCNVKNKLTSDEEVIVAKRIREYVIEHQAILDCISELQNHFSPALLVQLLTSVVIICVTAY
QLAVESSDLLRKFTMASFLFAMSTEMFTFGYQGGHLSHDSMEVATAAYSCPWY--
TFPTSLKRSLLVIMIRAQQPALLTAGGFTTLSLETFVTIMKASYSFFTVLQE--

>BmorOR58

------------------------MKLVFDNFIFALKVTLNWCRYFGIFIPDELT-GRRQKL----
LVQAYSVFMFMLFIGFFIITQIILF-ILVWGD---LSLMTDVGLVLGTNLAL--
SAKIAVFFFKREELASILKKNDD--------------TLRFETREEGKKIIS---------------------------------
----------------EYPCDT--
KRSPAYEIIMIHQTIAVAVIASLAITADLLMLSMIAVCRCRVKLVGLYLQTICDD---------
LPCNVKNKLTSDEEVIVAKRIREYVIEHQAVLDCISELQNHFSPALLVQLLTSVVIICVTA
YQLAVESSDMLRKFTMASFLFGMSTEMFMFGYQGGHLSHDSMEVATAAYSCPWY--
TFPTSLKRSLLVIMIRAQQPALLTAGGFTTLSLETFVTVS--------------

>BmorOR46

------------------------MAFFIRNKMLGLTITLNTLSWAGLIMRDQYTKTQRIIV------
RVYGWLVFLLFVAATYVQIADLI--DIWGD---LDLMAETSLLLFMELAV--
ISKILTLIFKYDKIMEIINGTEDILCSE-------NRLEGQKIIASIDKETT-
RFFQYYTSSVIFTTFFW----FLGEHSS-------------TFFIRA-KYPFNE--
LKSPGYEFALIHQCMMMVFTGYFEFNINIFFASVVAGCRCRLKLVALSLRNICIN---------
IPVNKKNLITPEEEKLITERLHCAISQHKYALDAAEDVKHCLSKVLLVQLTVSIVIICTTAY

QMAVS-TDTIQKLSMAGYLLGASFEVFLFCFQGQSLSNASEDIADAVYECPWY--
TLTQPLKRTLLIIMMRAQSPAILTAGGFVTLDITEYMAVLTGHGGFGDFLHR--

>BmorOR57

------------------------MPSLIKNRIFGLTLTLNTLSWAGLILRDDYT-KTQRII----
MKVYGGLVFLYLFVFTAYVQIADLV--VIWGN---IDFMTETSLILFMQLAV--
SAKVLTLMLKSKKIMEVTNEADA-------ILISEKKVEGQRIIASIDKNTT-
LFLKYYGFFVAFTIICW----FMGENTS-------------TFFIRS-KYPFNE--
LKSPGREFAFVHQCIVVIFTGSFDFNVDIIIISLVAVCRCRLKLVALSLRNLCLD---------
IPMNKRNLITSDEEKVITERLRNIISQHKRALDAAEAIKHYLSGALLVQLMVSIVVICTTA
YQLAVK-STTMQSLTMAGYLFGTSLEVFLFCYQGEFLRESSEEIADAAYECPWY--
TLTRPLKKTLLIIMTRAQRPATLTAGGFVTLDITEYMAV--------SLISNT-

>SlitOR44

--------------------------------YCYQGNQLSEESFEIAGAAYECLWY--
KFSLRLRRALLIVMVXHX-PSSTTHCWRIHYTFTCFFHG-NHSFVFISATTS--

>HmelOR47

----------------------VYTGDVKDYDATYTIPTVILKVVGIRIFLKDTKLKSICW------
NFYFWFNFVSILISCMLESINMFSMASVGT---FVDLFTMMPCVGYLIIG--
TCKAFKIKRYYYVYKNLVEELRD------MWPNGPITEEEHKVMNDALRRVR-
FIIKGNRNSTMALGSIFNGPPLIAWVKN-----RLGFDTELLMPFFF-
WLPFKTGRFEAIYFTQCFLCKLLTTMRIPGYMMSADLLYCIFLCHITTQFSLLSIRIKKFIY
VY----
SKKYLESQSNPIHDEETQEKLAAELFSIIQRHRALIRLSSDVESMYTFSLLVNFVNSSVIICF
CLFCCAFV--EKWNEFIYKMFLITSTLQTWMLCWYGQNLISTSTGVAESLYNCGWY--
TGSSKIKKSIFIMINRSQKQVHITTCGFSIISLASYATILKTSWSYFTLLIN--

>SlitOR28

MTSLYRKFLFKKKLKQRIDERVYNKRDYDASYAPTKKVLGWVAIRMTHNISEKFYWFE
MMNLFLVGPSELVSMLTTAYEAKTFRD---SIKVFRTMPCFGCVVLS--
MFKSIKMVVHRPVYENLANELRD------MWPEGEVSEEEHQIISAALKQLN-
FIVKGYYWCNNALLISFLSPPYFITLAR-----YFGYDSPMGLHFLY-WLPFDP--
YQSVYYEITLVLQTWHALVVIWFNVAWDMLFCLFLCHITTQFDLLARRVQRLFYVE----
FIQTEGERVNSYGAQYWEARHQKEITEIVLRHHSLIRLTGDVENMFSLALLINFMNSSIIIC
FCGFCCVLI--EKWNEVAYKSFLVTALSQTWLLCWYGQKLIDSSQRLADALYGCGWY--
NSSKRARSAVLIMLHRAQKGIYVTTHGFSVISLASYSTIIKTAWSYFTLLLN--

>MsexOR42

-------MASLWRKYFTKEIQVLKRVYEKSDYEDTYETPRKYLKWSGIRMKHNISKSLSICW-
-----LVYYWFCFVNIVFASVAEIIAMCMTASAGT---
FDDAIAIFQMMPCNGFCGLSLKSFKMVKHRPVFENLITEIGN------
MWPQRLVDEEEHKIISSALREIK-IFVKGYHWCNNYLMLSFLYPPFWELIKR-----
LSGEKWEPKLHFIY-WLPFDP--
LQPVYYECMLALQTWQAMTVIWTNMSGDFMFCLFLSHITTQFNLLSVRIRKLIYVE----
YLRKNKEPVDSYTPQQWEEKHFKEITEIVLQHQALIRLSRDIENMYSLTLLVNVVNSPLVI
CFCGF-CSVVKWNE---TAYKSFLVTALSQTWFVCWYGQKLLDSSEGVAEAVYNSGWY--
RASKKIRRSLMIMLHGSQKGVGVTTYGFSIISLASYSTIIKTSWSYFTLLLN--

>HmelOR42

--------------------------MDIPNFEEMFKQININFWLVGLNFHQSRV-------------
TLAYFIILFNIVIVLVEETGFFVSKFSVEN---FLELTQLAPCTCIGSLS--
LLKIIFIAKKKQNIFDLTQYLRE------LYNKALIDSSKKELIKQDFIFLK-
YLVKYFFILNAILICVYNFSSLVLIAYY----YYTKRTIFYILPYAM-LIPFST--
DHWYSWLIVYLHSIACGFVCVIYFTTVDALYYIMTSHICAQFSLLSEEIKGLDAD-----------
--------------TSCQLSDIVKRHQYILKLSQNLEEIFRAPNLFNVLVGSLEICALGFNLTMG--
PWSQVPGVVLFLLSVLVQILMISVFGENLIRESRKVGESAFLCKWY--
DMDQKSKKIILLLMLRSNKPQKLTAYKFSVISYESFTKIISTSWSYFTILKT--

>HmelOR43

--------------------------MDIPTFEEIFQQIKINFWLVGL-------PFGKSNV------
TKTYYILLFNIILAFVEEMGFFVSQFSVEN---FLELTQLAPCTCIGLLS--
FLKIIFIAKKKENIYGLTQHLRE------LYNKALSDSTKKELIKQDFIFLK-
YLVKYFFILNAILICVYNFSSLVLIAYY----YYTKRTIFYILPYAM-LIPFST--
NHWYSWLIVYLHSLASGFVCVIYFTTVDALYYIMTSHICAQFSLLSEEIKGLDAD-----------
--------------TSYKLSDIVKRHQYILKLSQNLEEIFRAPNLFNVLVGSIEICALGFNLTMG--
PWSQVPGVVLFLLSVLVQILMISVFGEHLIRESRKVGESAFLCKWY--
DMDRKSKKIILLLMSN--KPQKLTAYKFSVISYESFTKIISTSWSYFTILKT--

>BmorOR42

--------------------------MDIPKFEELLKQIKMNFWLMGI------P-FDNPKI------
QIRYYVLLLPLSLMLIEEIAFFGSRMSSEN---FLELTQLAPCICIGVLS--
VLKILALTAKRQKIYELTQNLEC------LHKIILNDTRKTELVRKNLVLIK-
FITKYFFVLNAVLIFVYNFSSPVIIAYN----YIVSNEVQFVLPYAV-LLPFKT--
DSWIPWLIVYVYSIFCGFTCVLYYATVDVLYCVMTSLVCNNFSLISFKLQKVNRN----------
---------------TAHLLKEVVKEQQYVLKLAEDLENIFTAPNLFNVLIGSVEICALGFNLMIG--
DLTQIPGCILFLSSVLLQILIMSVFGENLISESSRIAEAAFLCKWY--
EMDQKSKKTILTIMIRSHKPKKLTAYKFSVISYGSFSKIISTSWSYFTILRT--

>DpleOR53

-------------------------MRRTNGCARTDGFH-----------------------------
SLYRALVLVLTIAYLLQEFVYAY--QERND---MTKLSRVMFLLLCHITS--
IAKQIVFHYDADRIQEMIEGFDD-------PIYNGESASALSLLRSTTLVAR-

RLERAYAGCAILTCTLWLVFPVLHRLSG------------NPVHFAF-WTGIDT--
DPPAMFVAVLLYSYYVTTLVGIANTTMDAFIATILFQCKTQLTILRLDFQTLPQR----------
ALIECQKNDLTYESALIKLFVKCFLHYRRVTETAALLQDIFGTAILVQFGIGGWILCMAA
YKLVSLNVLSIEFASMTLFILCILTELFLYCYYGNELTVESELIVRSVYSMRWV--
SSPLRFQRLLLLLMLRVRRTLRPAAGRVIPLSLDTFVKILKSSYTFYAVLRQTK

>HmelOR26

--------------------------------------MRAPGGAGK--------------------
GGYRALVLAVTAAYLLQECVYAY--QERSD---MSKLSQVMFLLLCHITS--
IVKQIVFHIDADRIDQLVQSLND-------PIYNSTCPRARELLSHTTARAR-
RLQRAYAGCAALTCTLWGMFAAWRRACG------------GAVEFPF-WTGPRT--
DSPAVFIVILLYSFYVTTLVGLANTTMDAFIATILYQCKTQFTILRNEFETLPER----------
AKSLSIKTNEDYERVIMTLFVKCYHHYQKIIETSTVLGHIFGVAILVQFGIGGWILCMAAY
KLVSLNILSIEFASTALFILCILTELFLYCYYGNELTVESDKLVCSVYCMRWQ--
RAPLRLRRLLVLVMERARRPLCPIAARLVPLSLETFLKILKSSYTFYAVLRQ--

>SlitOR17

---------------------------MSVCAGSVAPHVRCLRRVGFCRWGAAAPANPLLAT---
AARFYHVFALAATSTYVLQQLIYAY--QERSD---MDKLSQVMFVLLCHVTC--
VAKQVAFHVDADRIDELIARLDE-------PLLNQCAGPRGALLHGTARRAA-
RLLRVYSGCAVATCVLWIVFPVLYRIRG------------ISFEFPF-WAGVSY--
DHNVVFGAVLLYSFYTTNLVAIGNTTMDAFMATILDQCKTQLRILRLNFETLPER----------
ARALQQSGGGVYEASLQRLFVDCLLHYNIITETCTVLHDVFAVPLLIQFGVGGWILCMA
AYKIVSLNVFSIEFASTTLFIICILIELFIFCYYGNEVTVESSRVSESLYSMEWA--
RAGLSFRRSLVLVMERAKRPLRPAAGRVIPLSLDTYVTILKSSYSFYAVLRQTK

>HvirOR17

------------------------MSLRSECARSVAPHVRVLRRVGFLRGAALP-ARPRAV----L-
RGYHALALAATSTYVLQQAVYAY--QERGD---MEKLSQVMFLMLCHVTC--
VVKQIAFHVDADRIDRLIAGLDE-------PLLNQCEGERGALLRGTARGAA-
RLLRTYVGCAVATCVLWIVFPILNRIQG------------ISFEFPF-WTGFSY--
DHNVVFSLVLLQSFYCTNLVAIGNTSMDAFMATILDQCKTQLRILRINFESLPER----------
ASALHMETGENYDTILDELFVDCLVHYNKITEMCAELHDVFTAPLLVQFAVGGWILCM
AAYKIVSLEVLSIEFASITLFITCILIELFIFCYYGNEVTVESERVSQSLYSMEWR--
RARLAFRRSLVLVMERAKRPLRPAAGRVIPLSLDTFVKILKSSYSFYAVLRQ--

>SlitOR26

---------------------------MSLSSGSVATHLRILRRCGYCRLAGGARVARLGS----
LHALYRGFTLALTTLYLLQECIYAY--QVRQD---MDKLARVMFLLLCHITS--
IAKQLVFHLKADRIDEMLGGLNE-------PLYNQSAAVHQRLLRVTAARAA-
RFVRAYAGCAVVTCTLWITFPVMYHLQG------------QLVEFPF-WINVDY--
NQSKMFILVLAYSYYVTTLVGIANTTMDAFMATVLNQCKTQLRILRMNFECLPLQ--------
-
AVEVLKTSPGQSYDAVLMKLFKECLLHYEKITKTAKMLQDIFGTAILIHFGIGGWILCRA

AYKIVTLNVMSVEFASMTLFISCILTELFLYCYYGNEVTDESSRVSESLYSMEWA--
RAGLSFRRSLVLVMERAKRPLRPAAGRVIPLSLDTFVKIIKSSYTFYAVLRQTK

>BmorOR26

----------------------------SLSGSSVFTHLFLLRCCGFCRLSRSSTARRGLSV---
AHEVYRALTLTLTVVYLLQECVYAY--QERTD---MDKLSRVMFLLLCHITS--
VAKQLVFYLDADRIDYLIATLDD-------PSY--NEMSHQRLLVDASRWAS-
RFVWAYSGCAVVTCTLWIVFPIIYHVQG------------QTVEFPF-WIQIDY--
TKSSMFVVVLLYSYYVTTLVGIANTTMDAFMATILGQCKTQFTILRIKFETLPTR---------
AKQALRCDSEQNYDEVLMRLFHDCLKHYQKIV-----------
SAILIQFGIGAWILCMAAYKIVNLSVLSIEFASMILFISCILTELFLYCYYGNEVSTESERLV
TSIYSMEWV--
GARLGFQRGLLVLLERARRPVRPAAGLVIPLSLQTFLKIIKSSYTFYAVLRQTK

>MsexOR3

MTSAGSSVAPHLRCLRMVGFCRLSAGTAGATGRGRWRRAHGAYHAFALSATSTYVLQ
QLIYAY--QERGD---MEKLSRVMFIMLCFVTC--VVKQIVFHVDATRIDMLVAGLAE-------
PLFNPAAEWARALLRETARAAR-RQALAYAGCAVATCVLWIVFPVLDRARG-----------
RHVHFSF-WAPIDC--
THTAKFLTLLLYSFYATSLVGVGNTTMDSFVATVLHQCKTQLTILRINLETLVER---------
ARAACGDARDQYDRVVYKLLVESLVHYQKITQTCALLQDIFGAALLVQFGVGGWILCM
AAYQIVSLDVMSIEFASTTLFIVCILTELFLYCYYGNEVAVESDLVLQSMYALEWL--
AVPRRVRRALLIAMERAQRPMRPAAGAVLPLTLDTYVKILKSSYSFYAVLRQTK

>BmorOR59

-----------------------MDTNPSAAGDSVAPHLRRLRQVGFCQLDPTSQSRRPILAL--
MHRVYHRLVLAATVLYIFEQLTYAY--QARND---MERLSRVLFLMLCHLTC--
IAKQFVFHSDADKINQLVVGLDD-------ALCNQPVETHRLLLLETSRRAA-
RLLMLYSGCAVSTCILWAVFPLLDQLRG------------RTVEFAF-WIPIDY-
RHNAFQFAVVLAYAFYSTSLVAVANTTMDAFIATVLYQCTTQLRILRMNFESLPER-------
---
AYALSRKTRQDYHTVTHELLVDCLLHYKKITETCNLLEQIFGKAILVQFGVGGWILCMA
AYQIVDMEILSIEFASTALFMGCILTELFLYCYYGNEVTVQSGLVSESVYAMSWL--
SLCPRERRALVVVLERARRPLRPAAGRVVPLTLNTYLKILKSSYSFYAVLRQTK

>SlitOR31

----------------------MEDNVAYSTFEGFRPHFDALARVGYFKIVLKPISSLKRF----
FHNVYRIISWSLILTYNLQHVIRVV--KVRHS---FNLIVDTLFILLTTLNT--
LGKQTAFNLRSRRIDDIISIING-------PTFAASKPYHVEVLKQNALVMS-
RLLTLYHGAIFTCGTLWTVFPVVNRALG------------KEVQFTG-YFPFET--
TSTLAFSLALAYMTVLITFQAYGNVTMDCTIVAFYAQAKIQLQMLRYNLEQLVEFN----
IKKFNSIKTQYRDEGEEKTELQERLKKCVRHYDQIVRYAKEVESIFGEAMVVQFFVMAW

VICMTVYKIVGLSICSAEFVSMAVYLGCMLAQLFIYCYFGTQLKVESELVNQSIYCCDWL
--CLSPQFRRQLLVMMQCCGRPLAPRTAYVIPMSLDTYIHVLRSSYTLFTFLNR--

>CpomOR31

------------------------MTTTYSTFEAFRPHFNALAYVAYFKIIPKPSSGVKHT----
LHTVYRAVVWFLVIIYNLQHVIRVI--QARHS---TEQAVNTLFVLLTTINT--
LGKQVAFNSRVERVDRLVATIEG-------PLFMARNTYDEKVLRSNAWIMS-
RLLMMYHGSIYLCGAMWGISPLVSKLSG-------------EVELTG-YFPFDT--
SGWLGFGIAVAFNTIVITLQGYAHVTMDCTIVSLHAQTKVQLQMLRNSLEHLTDSR----
EICVQSTVYKDIEDTAFGVVLKKRLTRCVEHYKLIVWFHSEVEAVFSEAMIVQFFVIAWV
ICMTVYKIAGLSLVSAEFFSMFVYLGCMLGQLFIYCYYGTQVKAESEFINYSIYRCDWV--
SLSPRFRALLLILMSRGMRPVAPRIAYIIPMSIETYISVLRSSYTLLTFLERK-

>HmelOR24

--------------------------MEVKLSYRCVEQHLLRLRVGGYYQIDPHSPTLHKR-----
LHDIYMRITLVWIIVYTVQQAIKLY--EVRDD---MDMVMGTLFLFLTHTDS--
IYKQLVLWIKADKVEELLDIMKGGIYNLLCQLFNQGEDDHMTYLLQAAYTST-
CLLNTDNCLALATCFLWTLLPFVLHLQG------------KSVEFAI-WFPFDV--
NVDPAFYFLLLYVWFTTSWLAFCNTTMDVMSFLFGQCKTQLSIMFVILSRKYLP---------
--------------------------ATERIW----
MIEDIFSTAIAYQFLISGWIICTSVYRMVDMSPASVQFLSMVLYISCILIQIFMYCYYGNEV
TIESENLTGSAYSMNWL--
EINLKLRRHVILFMERIKRPIQPLAGRIIPLSNNTFVSIVKNSYTFYALLKNTN

>DpleOR51

--------------------------MADIKSEDLFLNR-------------------------------------
AKFVMKLLGVWIIYIVQVWGD---LEAVSQASYLLFTQASL--
CFKVTVFQVNVKSLRELLKRMND-------KIFLPQSSKQNRILSLQAKRIK-
RLLLAFMISSQTTCGLWALKPLFDDPGS------------RKFPFDM-WMPVTP--
QSSPHYEFGYAFQLLTICMSAYMYFGVDSVALSMVIFACGQIDIIKDKILKINSI---------
RKESGDEMSRRIVASDNNKKLIECIRQHQAVVLFKELLENTYHTYLLFQLVGSVGLICMS
ALRILVVEWRSIQFFSIVTYLSVMISQLFVCCWCGHELTATSEDLHTVFYRSTWY--
EQDVRFQRCLCFAMMRLGQPLVLRAGHYIPLSRQTFVSILRMSYSYFAVLNQTK

>HmelOR16

------------------------MKVLKSEDLYLNRAKFVMKYLGV-----WV-PAKNET----
ASKCYRLFMMSLQYLFLLFQIIYII--LVWGD---LEAVSQASYLLFTQACL--
CFKITVLQINMDMLKDLLRRMND-------KIFFPNSKEHERILKLQAKRIK-
RLLLAFMISSQTTCGLWALKPLFDDAGN------------RKFPFDM-WMPVSP--
EFSPQYELGYVFQLLTICMSAYMYFGVDSVVLSMVIFGCAQLDIIKDKILNIGFV---------
DIKGQDSMEKKTIIAENNTKLINCIRQHQAVVLFTELIENAYHTFLLFQLVGSVGLICMSA
LRILVVDWRSMQFFSILTYLSVMISQLFVCCWCGHELTATSEELHTVLYKCAWY--
DQDLKFKRTLLFAMLRMDRPLVLRAGHYISLSRQTFVSVS-------SVL----

>SlitOR32

---------------------------MVSSEDLFLNRAKFVMKYLGV-----WVLADNASY----
FLKAYRGFMITLQYLFLIFQTIYII--QVWGD---LDAVSQASYLLFTQACL--
CLKVTVFQINMPMLKDLLRLMDS-------KIFRPENDVHEKLLELQAARIK-
RLLLAFMVSSQITCGMWALKPLFDDADR-------------KFPFDM-WMPVSP--
EDAVQYYIGYAFQLGTICISAYMYFGVDSVTFSSVIFGCAQINIIKEKLMSITSI----------
KEKRGTKAVDEALAENYNKLVDCIKHHQAIVTFTELVENAYHPYLLFQLIGSVGIICMSA
LRILVVDWRSMQFFSILTYLSVMISQLFVCCWCGHELTATSEDLHRVLYKCYWY--
EQDVKFKRELCFAMMRISRPLVLRAGHYIILSRQTFVAILRMSYSYFAVLNQTH

>BmorOR16

--------------------------MSFNSEDLYLNRAKFVMKYLGV-----WVPPENENF----
ARKFYKIFMMSLQHLFLFFQIIYIV--EVWGD---LEAVSQASYLLFTQACL--
CFKITVFQINMNKLKELLKQMNG-------YVFQPKNINQQNIIKVQATRIK-
RLLFAFMISSQLTCGMWALKPLFDDVGS------------RKFPFDM-WMPVSP--
ERSPHYHLGYSFQLVTICMSAYMYFGVDSVAFSSVIFGCAQIGVIKDKIMSIKPL----------
GIYRNHKTYTKISRYNRKTLIECVKHHQAVISFTELVEDTYNSYLLFQLVGSVGIICMSAL
RILVVDWRSVQFFSILCYLSVMISQLFVCCWCGHELSATSEELHTILYNCAWY--
DQDVKFKRDLNFMMARARRPILLRAGYYISLSRQSFVSILRMSYSYFAVLDQ--

>MsexOR32

------------MSCIMYLAVSEQLTMTLNSEDLYLNRAKFVMKFLGV-----WVPPNDENI----
IRKLYRYFMISLQYLFLIFQIIYII--QVWGD---LEVVSQASYLLFTQACL--
CFKVTVFHVNVDLLKELLLQMNG-------ETFRAQSFDHEKILESQAARIK-
RLLLAFMISSQLTCGMWAVKPLFDDVGS------------RKFPFDM-WMPVTP--
ESSPHYHIGYTFQLVTICMSAYMYFGVDSVALSSVIFGCAQIEIIKDKLMSIQPV-----------
DKNANDAINAFSDNYQQLVNCIKHHQAIVRFTDTVENAYHTFLLFQLVGSVGIICMSALR
ILVVDPRSMQFFSIVSYLSVMISQLFVSCWCGHELTATSEDLHTTLYMCAWK--
DQNTKFQKELCFAMLRMSRPLVLRARHYIALSRQTFIMILRMSYSYFAVLNQ--

>MsexOR45

-----------MDVCIMYLAVSEQLTMTLNSEDLYLNRAKFVMKFLGV-----WV-PPNDEN----
IRKLYRYFMISLQYLFLIFQIIYII--QVWGD---LEAVSQASYLLFTQACL--
CFKVTVFHVNVDLLKELLLQMNG-------ETFRAQSFDHEKILESQAARIK-
RLLLAFMISSQLTCGMWALKPLFDDVGS------------RKFPFDM-WMPVTP--
ESSPHYHIGYTFQLVTICMSAYMYFGVDSVALSSVIFGCAQIEIIKDKLMSIQPV-----------
DKNANDAINAFSDNYQQLVNCIKHHQAIVRFTDTVENAYHTFLLFQLVGSVGIICTSALR
------------------------IL-----------VSEDLHTTLYMCAWK--
DQNTKFQKELCFAMLRMSRPLVLRAGHYIALSRQTFIMILRMSYSYFAVLNQ--

>DpleOR52

--MGIWLSSNNYVI----
LRDLYKSLIMSTQYSFLLFEIFYVF--VVWGD---IDEMSEASYLLFTQTSV--

CYKASVFIINKKNLIVLLVSMKA-------KIFEPQSSIHYGILYRNARVVK-
NLCAVFLMSAITTCSLWTIMPLFDAAET------------RFFPFKI-WMPVDP--
RDSPQYELGYIYQVMSIYISALLFVAVDNITLGMIMFGCAQLEIVIDKIKQLKNA----------
SSKHNKERRKLICENNKLLDECIKQHQSVIRFIETLENTYHANIFFQLTATVGIICIIGLRISI
VEPRSVQFFSMLNYMVTMLSQLFLYCWCGSHLTTKSEELREWLYQCPWY--
NQDPRFRKSVFIAMERMKRPIIFKAGHYIPLSRPTFVSVLRLSYSYFAVLNQAK

>SlitOR10

------------------------MEAEKNTTLFLGRPKKILTAHGV-----WPHPNNYII----
LRKLYMLFVMWTQYSFLLFEIIYIV--NVWGE---IDEVSEASYLLFTQASL--
CYKSTTFMINKKSLLELLDIMDS-------DIFKPKSAEHEKILAAQALKIK-
RLCLFFLTSATTTCTLWAMIPLFDDASK--RSFPFRICKQETLTLLF-RMPVTP--
LKSPDYELGYLYQMISIYISAFLFISVDS-VAVLIMFGCAQLEIIMDKIKKIQSI---------
FNTDQSEEGRKKLLKMNNELLDECVKQHQTVERLIQLCEDTYHANIFFQLSGTVAIICNI
GLRISIVEKNSVQFFSMLNYMVTMLSQLFLYCWCGHELTIRSENLREWLYQCPWY--
EQDTKFKRSLFIAMGRMKKPIIFKAGHYISLSRPTFVAILRCSYSYFAVLNR--

>HvirOR10

IDAVSEASYLLFTQASL--CYKSTAFMVNKQSLLELLEIMDC-------
EIFEPKSAEHEKILAAQARKIK-RLCLFFLTSATTTCTLWAMIPLFDAASK------------
RSFPFRI-WMPVTP--
LKSPDYELGYLYQMVSIYISAFLFISVDSVAVSMIMFGCAQLEIIMDKIQKIKYV---------
FESADSEEKRREIIKINNEFLVECIKQHQTVERFIQLCEDTYHANIFFQLTGTVAIICNIGLRI
SIVEPNSVQFFSMLNYMVTMLSQLFLYCWCGHELTIRSENLREWLYQCPWY--
EQDTKFKRALFIAMERMKKPIIFKAGHYISLSRPTFVAILRCSYSYFAVLNR--

>MsexOR31

--------------------------MAQNTELFLGRPKKILTFFGI-----WLPSKQYQD----
LVKIYMILVMITQYSFVLFEIVYII--NVWGD---IEVVSEASYLLFTQASV--
CYKTTAFLIKKRKLVLLLEHMEQ-------EIFASQSKDHEKILLELSLRIK-
RLCTFFLTSAITTCTLWAMIPLFDDAGH------------KTFPFRI-WMPVTA--
DESPGYELGYLYQMVSIYISAFLFIAVDSVALSMIMFGCAQLDIIKDKMQKVREV----------
PICLNVDEKKEKLKSNNDLFTECVRHYQNVISFVELVEETYHANIFFQLSGTVAIICNIGLR
ISIVDHHSVQFFSMLNYMVTMLSQLFLYCWCGNELTIRSEVLRDWMYQCPWY--
DQDTKFKRTLWITMERMKKPIIFKAGHYISLSRPTFVAILRCSYSYFAVLNR--

>CpomOR10

----------------------MTVSTVDNVTLFLNRPRNILLYLGI-----WLKPANYVS----
LYVAYAIIVMLTQYSFVFFEFIYIA--LAWGD---MDAVTEASFLLFTQASV--
CYKVTRFMINKDNLVFLLSFMEE-------EVFQAQNERHVRCLLNQSIMIR-
RLCLFFLGSALTTCTLWGLMPVVDSTGG-----------ERIFPFLI-WMPVGP--
EKSPQYELGYFYQMVAIYISAFLFIAVDSVALSMIMFGCAQLEIIMDKVQQIKRV--------
PMSGKVKKQDREQLIQENKVLFVECLKHHQAVIRFIESAEDTYHANIFFQLSGSVAIICIIG

LRITATTPGSVQFISMLNYMVTMLSQLFLYCWCGNELTIRSEILREVMYLCPWH--
EQSNSFRRLLWVAMERMKRPIIFKAGHYIPLSRPTFVAILRSSYSYFAVLNQ--

>BmorOR41

--------------------------MMGNSTDLFLDRTKSILNFFAMWRSFEKPIPL----------
KVYMAFIMTTQYLFLIFEIIYIV--NVWGD---MAEVSEASILLFTQASV--
CYKITSFISKTNNFVILLGLIES-------EIFSAQTELHEKILILKARKIK-
RLCMFFLVNAVTTCSLWAVIPLLDISSK-------------MLPFKI-WMPAST--
GESPHYELGYLYQMITIYISAFLFIGVDSVPLSMIMFGCAQLEIIMDKIGKVKSR----------
PLDQQPMQRQAVLNSNYELLVECVRRYQSVVRFIELTEKTYHANIFFQLSGSVLIICNIGF
RIAIVDSNSLQFYSMLTYLVTMLSQLFQYCWCGHELTIRGEELRETLYQSPWH--
EQDIRFRKVLIITMERMKRPIIFKAGHYIPLSRPTFVAILRCSYSYFAVLNR--

>HmelOR41

---------------------------MNSNIEIFLSTPKIILIWLSI-----WLRIGDHVI----
LHQLKQAIVLFTQYSFLLFELIYII--VVWGD---LDEVSEASYLFFTQASV--
CYKVAVFVINKNKLKKLLEFMMV-------DIFASQSTVHDGYARTHVSPLI-TVCIQNLAE---
----NEERSILTLGS-------------LTHSHCFFRMPVSQ--
HIFSQYMIGYLYQIISVYISALLFFAVDSTTLSMIIFGCAQIEIIIDKIKK---------------------------
----
VKHISKCFRFIELLENTYHANIFFQLSGTVAIICIIGLRISITEPDSVQFYSMVNYMVTMMS
QLLLYCWCGNELTTKSEDLREWIYQCPWY--
EQDSKFRSSLVIAMDCMKRPIIFRAGHYIPLSRPTFVSV---------------

>BmorOR10

---------------------------MRTNAKSFLFVPSKVLTLCGV-----WPVEKTSI-----
FSLIYRSIMLSSQFCFLVFNGIYIG--LMWGD---LKAVSDALYMFFTQTTC--
CSKAIGFYFNFMKIKRIVASMDD-------VLFTAMSIEDQATIFSHSRTVN-
KLYKGVLGFTGFTLVQWTVLSLIGSGRT--------------LPFNEMWVPTDI--
SKSPNYEITFVVELWMMVISAALFMSVDTITVATMMFSCAQLDIIMKKTQQIQEI--------
PLSPDLSSRNRSELHEKNNGILIDCIKQHQAIVRFSELCEGTFQVHSFFHLGGIVFMICVIGF
RMAGESPVSAQFWAALSYLVIILGQLYLYCWCANELTTKSEQLRDKLYLTPWY--
DQDVKFKRNLCIAMECMAKALTFRAGSYIPLSRAMFVSILRSSYSYFAFLNQAN

>BmorOR40

-------------------------------MTGAGAGT--------------------------------------
FRTGAGPGRGDGVARRGESGE---
TTTLGRDAFAALGCFGAADGSTARARFFPRVTVLNPSEVPGSGLAADSNSISDSESEPELD
AAQDAIDAGA------------------GVGGDIGESRA--------------RTVFG-
IQGHDASDSALRMHNNVAIYA--KTTMSGNSQLTFATAATIFLKNASGP-----------------------
------------NGVAIGTDYAICVVSLSLFFRFTELVEDTYNSYLLFQLVGS-
VIICMSALRILVVDWRSVQFFSILCYLSVMISQLFVCCWCGHELSATSEELHTILYNCAWY
--DKDVKFKRDLIFMMARARRPILLRAGYYIGLSRQSFVSV-SIRIRFNAILVI--

>SlitOR27

MIILNENMKTKLAFLSPVVPYGVFESWEDLNPQLYHAVHIYWLKFYGM-----WF-
NDFSPQ----Q-MMYTLIVLWVCFFPGIGEVVYLL--RRREN---IGDVAEGLYLFLSEMYT--
YFKVVVFWLNRNKVIDLLKYLHC-------EEFKPKEPEHRDIIIKSIKSAR-
FVMTYYSTMCVGAVSVGIIMPLTENFDI--------------LPTNV-EYPFFN-
VYQTPAYEAVYIHHIYYKPATCIIDGVMDTILAAFVASAIGQIEILXFNLRNFELL------------
------
EYKPPYYVRSVLKDCIVHHNCIIRYVSMIESAFSLASALQFMLSVMVLCLIGIQFLSIPTSH
PMQAWMGIYLTCMLIEVFILCWFGDELIWKSMDLAKAAFEGPWM--
NSDRKSNMFIIIFLERCKRPMRLTAGKIFTLSLDTYTVLINWSYKAFAVMRN--

>DpleOR34

-------------------MSFFKRFTDRVCESDLFDFNLKYLYYVGLWPREDWPKYKQILY------
KIYEFKLHLLSITFIVITGIGTY--QQRNN---VIMLMTNLDKTLVGYNF--
VLKIFFFVIKREKLNVLINEIKN---------------SGDKVSDDRKTLMA-IHVIFITFISTALVSAF--
-SLLSQYKA-------------EMTVEA-
WMPFDPFKDRMSLLMAAQILAICFVVPCLYRAFAMQGIVCSIIMYFCDQLLHLQGNLKK
LGNT-------------------
KHNEMQARKEFKGIVKKHVRLMRYTKTFTEIFKEFFLIQNLAVTIELCLNALMVTVVLD
EKTLLASFLTFLGLALFNAYIFCYLGDELIILSTGISQAAYESEWT--
SWPIDMQKDLLIIIKVAQRPLKLSAGGMAIMCIQTYSQALYNAYSIFAVLNDVV

>HmelOR18

-------------------MAVLKAISEQILHRDLFDFNLKYLTFVGL-WPENWNRDKLFLY------
KIYEINLHFLSIMFIVLTGIGTY--QYRDD-ITIAMTNLDKCLAAYNFVF----
KIFFFVLKREKLRALIVEIRS---------------SSDKVNDDRKMLMG-
IYVIVITLVATILVGAFTLVAQLK----------------MEMTIEA-
WMPFDQYENRSSLLMATQILAVGFVVPCLFRAYAIQGIVCSIIMYFCDQLEELQDGLSNL
DYS-------------------
EETDRFVREELKNIIKKHVRIMGYSKTFTSIFKEFFLIQNLAVTIELCLNALMVTVVLQEKT
LLASFLVYLGLALVNAYIFCYLGDELISQSTGIANAAYEIGWT--
SWPKDLQKDLLILIIAAQRPLTLSAGGMSIMCIQTYSQVLYILATIF-------

>BmorOR18

------------------------MGDRMVTRGHFFDFNIKYLFYVGLWPSNEAKRIEKIAY------
KIYEYQLHVLSLIFLVTTGIGTY--KNHKD---IIALLTNLDKTLVAYNF--
VFKVIVFVYKREELRKLIEQIVQ---------------SGDQITEDRKALMA-
KLVIVLTGISTVIITAFSCLALFEGEMT----------------IDA-
WMPFDPMKSKMNLFAASQILAATFVVPCGYRAFAMLGIVCSLILYLRDQLVDLQNKIRD
LRFA-------------------
TGNVEKLRDDFKLIVKKHVRLLGYSKVIEMIFKEYFFIQNMAVTAELCLNAMMVSVVLE
QKTLAASFLAFLSVALLNAYIYCYLGNELIVQSEGIAMAAYESSWI--
LWPVDMQKDLLIVITAAQKPMKLSAGGMAVLSVQTYSQTLYNGYSIFAVLNDIV

>CpomOR21

-----------------------MFGSLLELSDDFFAYNLKYLFLVGLWPDDAWAKTHPSLY------
KIYENITHVLSIIFLITSGIGTY--QIKDD---VVLLMTNLDKCLVAYNF--
VAKVGIFVWKRRQVEILISEIVN---------------SGDQLTEERKKMML-
MIIIVVTGLSTSIVGAFSALALYHNELS----------------VEA-
WMPFDPMESKMNLLTASQLLAITFVVPVIWRAIAMQGIVCSLIMYLCDQLVELQDRIRSL
EFT-------------------
SMTERVVREEFKNIVNKHVRLMGYTQDMNKIFEEYFLIQNLAVTLELCLNLMATMIGFE
QKTLLATFFAFLCVALMNAYIYCYLGNEMIIQSGNLALAAYESSWI--
SWPLDLQKDLVILLRVAQKPLYLSAGGMVAMSIQTYSQTLYNGYSIFAVLNDVV

>SlitOR43

-------------------------NVFILIDDGFFSFNIKYLFFVGL-----WPEKTLTRN----
QKFLYKIYELFIHAITIIFLIMAGVGPIKNKDDILIVLSNLDKSLVVYNFFF----
KTIIFLIKRDQLSDLIDEIEA---------------SGDEVTKERKKLMA-
NYVMAVTGMTAAVVSTFSLLALLEGIMS----------------IEA-
WMPFDPVKNSMNLVLSLQII-
AFCVFPGLCRAFAMQGLVCSMIMYSCDQLIHLQKELRSLDYV-------------------
KDTEMITRMKFKMIIKKHIRLMGYSMKMESIFNEYFLVQNLAVTVELCLNAVMVSIVVQ
QITLLFTFLAYLVLALINAYVYCYLGNELIIQSEGIALAAYESTWT--
SWPVDLQKDLLILINASQRPLKLSAGGIALLSIQTFSQALYNGYSIFAVLNDVV

>HvirOR3

-----------------------------LIDDGFFSFNLKYLYFVGLWPEKTLTANQKILY------
KLYEYFITFLTTTFIVLASIGTY--QHKHD---LVVVFCNVDKCLVVYNF--
FLKTIIFFIKRDKLRDLIDEIEM---------------SGDKVTEERKKLMA-
NYVMFITGMTAAVIGAFSLLALLEGTMS----------------VEA-WLPFDP-
MESLMNQILALEVLAFCVFPGLCRAFAMQGLVCSMIMYLCDQLTHLQTELRDLTYV-----
----
KESEMAMRLKFKNAIRKHIRLMGYSGRMESIFKEYFLVQNLAVTVELCLNAVMVTVVV
HQITLLVSFVAYLLLALVNAYVYCYLGNELIIQSQGIALAAYESTWT--
SWPVDLQKDLLIVILAAQRPLKLSAGGMALLCIQTFSQALYNGYSIFAVLNDAV

>MsexOR20

-------------------MAVSVLKRLLTYGDNIFEFNIKYLMLVGLWHNENWTRNQKLLY------
KIYDNTLDVLGLIYLTLTAIGIH--GNMDD-ITAALAGVDKSLVAYNFML----
KIIVFHFRKHQLRKLVKEIIA----------------
SGDVVPEEHKILVAKLSLATTTITTIIVTIFTGISVLA----------------GELPAKV-WLPFDT-
SKNFMNLLAGVQICVTFGVPICYRGLALKCFVSTMIFYLRDQLIDLQVKFKELDNF--------
-----------KDAEEEVRTNFKKIVKKHIRLIRYSKTIDNLLREYFLIQNLAITIEVCMNSVTLT---
--------------------------------LSEGISSAAYNTSWT--
SWPVDMQKDLLTVIIVAQSHFKLTAGGMAVMSLETYAQTLYNGYSIFAVLID--

>MsexOR21

--MLVGFWHNENWTRNQKLLY------
KIYDLTMHVFGITDIALSAVGIYQTKHDLT---VALAGVDKALVAYNFMF----
KIIGFHFTKDQFKKLVKEMIA----------------
SGDVVPENHEILMAKLSLATTITSTIIVVIFTGSSMMVG----------------DLPARV-WLPFDI-
SKSFMHLLAGVQFCVIFAIPVCYRGLALQCFVSTLIFYLRDQLIDLQDKFKELENF----------
----
QNVETELRRNFKKIVKKHIRLIRYSKEMDYLLREYFLIQNVAFTTEVCMNAVILTLIGFSQ
KTLINFIAFLITALIHAFVYCYLGDEIIEQSKA-----YRLLLT--
IQPSTMQKNLLTVIIAAQSHLKLTAGGMAVMSLETYAQTLYNGYSIFAVLADAV

>HmelOR22

-----------MALFDNLRKKILGSDFDYDGFVGDLYEFHPQLKILLVTNGIFFNNRDSYWR------
FLWPVISVLLSIITCAFELMFIHHGVDIED---YSFATECFCYFLIIFTI--
PIVYTSVMLNRNNIIQILDTMNR-----DFLFICNLGSKYRKTFLQGQMLIK-
KLCVCWLIFCVSIVLMYIFSTTAILLYQ-SLFATQHENMVRPLIFPF-WLP-
ADDPYRTPNYEIFFFLQCVEGLIVPQTFMYIYVLFHILLHHYYL-MSMMTFDIEVLFHGL---
-
DESVVSLSYTDSRLMEVKVILRNRMRRVVKWHNLVLGSVDAVSSVYGPPLVYQVTITSL
VTCLVAYQVADSLDHGKLHVIFTMLFIATCVQLWIPCYLGTLIRNKAFAVGEAMWNSG
WH--TTLGRLLRSIIIFIKRCQKPVSIKFTGLPNLELETFSSV-GVSFVYLNIINLFL

>SlitOR22

------------MRLQIIKDFLMKASFDFDRPDIDLYNFRIFLAVKGVFFTNRGSR----------
FRLIWPSICIQLSIIGMTFEEMFIWRGVSLKD---YSFATECFCYWLLLGCI--
PTVYVSILVHTNKIYDIVVKMNE-----EFIYVCSLGPRYRKPFLEGQLLIW-
QLCYAWFGFVSFVGGLYVVFPLVGLIYQ-SLFATLNENTTRPLQFPM-WLPHDD-
PYRTPNYELFLFIQSTLCFCFVQTFCVYIYTLFHILLHYYMIMDMIIIDFSVIFDGL----
EESVALLPRHDSRRMETQRILNARIEKIVKWHLSVFGAVKTVSSIYGPPLVYQVSFTSIAIC
LIAYQIAEKLDHGSLDILFSLLSICACLQLWIPCHLGTMIRNKAFEVGDAGWTCGWH--
PLGLMIRTDIIIIILRAQQPVTIKYTGLPQIQLETFSSCMSSSYSYFNMLRQYS

>MsexOR22

-----------MGWIERIKGFILKKSFDFDRPDICLYNFRILFALKGIFFNKQNSK----------
LKIILPTYFNLLTILGMVFEGMFAHRGLTIKD---YSFAIESFLYFIILTST--
PLVYLCLFYHKDKIIQLLDDMNE-----EFKFVCSLGPRHRTPFLKGQLLIW-
KLCYAWYTLSISTGTAFMMFPVMALVYQ-TLFVTHTEKTIRPLAFTM-WLPNDD-
PYRTPNYEIFLFFEMNYCVIIVQTFGVYIYTLFHLLLHHYFILDMMILDFEAIFDGL----
DESVAALPSKHPRRREVQLILNARIKRIVTWHNSVIKTINTLSIVYKPALVFQILLSSIMVC
LIGYQIAESLDNGVIDFLFIMLGVCTCMQVWIPCYLGTLLRNKVFAVREACWNCGWH--
SLGTLIRLDILIIIQRTQVPLLIKLSDMSTVELETFSSIMSAAYSYFNMLRNSN

>CpomOR24

-----------MKAFVSTAKTFLYKNDFEWNKEITLQNFQIFLAINGVFFNNRESK----------
IRFILPVLSTLITLVAVAFEIFFIWHGISMND---YGFATECFCYFFILGSV--

GIVYSSVLLNRVKVFKLLHNMNN-----DFLFICNLKAEYRDTFLTGQLLIW-
RLCWSWIVFISFVSLLYISNTLLYLLYQ-STLATQDEHMIRPLIFPM-WLPEDD-
PYRTPNYEIFLALEVILIFVVLVTFGLYVYILFHLLLHYYNLMDVILIALDDLFDGL----
DESVVALNRGDPRRQAVQDELNIRMGQIVRWHLSVFDSVDDISSVYGPTLVYQVMFSSI
VICLMAYQVAEQLSEGKVDYLFGILGIGACLQLWIPCYIGTLLRNKGFFVGDRCFYCGW
H--PLSRLLRPDLIIFIQRTQRPVAIKFTGLPHLQLETFSSIMSNAYSLFNMLRQYK

>CpomOR22

PGEYSLCSQSNMTLLTIFRDIKSFVNKDGYDLERPDVTLQNFHPQLEVFFAIKGIKRFIWP
ALSSFMASVATGFELLFIWRALTIKN---YAMATESFAYLIILGSV--
ILTYLGVLTNRTTILTLLSEMSK-----DFRYICNLASNYRKSFLDGQLLIW-
KLTMTWGIFVICVAILYVLNTLLLLLYQ-SLFATLDEHYVRPLIFPV-WLPHDD-
PYRTPNYEMLLVFDIAIILVAMASFGLYVPLSLHLFMHYYKLLDMILIAIDELFEEL----
DESVVTLPVTDQRRLDVKAELSRRMGRIVRWHQSVFDSVGAITSIYGPMLVYQVMFSSV
IICLMAHQVAIQMADGKFNYLFALLTFGAILQLWIPCCMGTLLQTKALSLGERCFYSGW
Y--PLTQLVRQDLLIFITRTQVPVEIKFTGLPEMELHTFSSIMSTAYSYFNMLRQYN

>BmorOR66BAH66361.1

----------MRFGLKVYIYTLFHILLH----FYYILHMIKFDLEAIFDDI----
DESVALLPHRDTRRIEVQKILNGRMKRIVTWHISVFKAVEAVSSIYGPPLAYQVMFTSIAI
CLIAIQITQKLENGILDIRFTMLGVAACLQMWIPCYLGTLLRNKAFGVGEACWNSGWHQ
TPLGRMIRQDIIIVLLRAQQPVTIKFPGLQSIQLETFSSVIFNLYGYYYYYCLDG

>BmorOR65NP_001166622

---MRLGFEVSISEYLYRNIFYIYTLFHILLHFYYILHMIKLDLEAIFDDI----
DESVALLPHRDTRRIEVQKILNGRMKRVVTWHISVFKAVEAVSSIYGPPLAYQVMFTSIA
ICLIAIQITQKLENGILDIRFTMLGVAACLQMWIPCYLGTLLRNKAFGVGEACWNSGWHQ
TPLGRMIRQDIIIVLLRAQQPVTIKFPGLQSIQLETFSSVIFNLYGYYYFLLLRW

>DpleOR65EHJ63907

------------------------MIFHVLLHCFYMLSIIILDCEALFGGL----DESVVKLDRY--------------------
---------IQSVSSVFGPVLVYQVSLSSVAICLAAYQVAH-------------------------------
AFTVADAIWESGWNETPLSLLIRTDMMIIILRSHNPITIKFTALPNLELETFSSVSIYMSVK--
------

>Bany_OR4_GZXD38A02FY60I

----------------------------LLHHYYLMSMINFDCEVLFVGL----
DESVVRLPKHDPRRMGVQTILNSRMRRIVKWHNLVFQSIEAVSSIFGPPIVYQVMFSSLLI
SLTAYQVAESLDHGKLNVMFAMLGIAMCLQLWIPCYLGTLFRNKAFSVGDAMWSSGW
HETPLGRLLRADITLVIMRSQLPVSIKFTGLPNLELETFSSIMSTSYSYFTLLRQYT

>DpleOR29

------------------------MMWPINFEEMYKISTYSLRLNGAHPNIKNKNKFWYLK------
IYLLRFIELLCCGLLVNSIIFYD--VVSKK---YTEAIKNSLMVIVGITV--
IFKHSILINCNDSIKQLLKILDE-----DYKAAELYGEREKDIILKSAMSGV-
KICRFWLVSATLTCFMFPAKAIIEMGNL-----YMRGEFKLVPMFDF-
TYPIEVHKESTVAYTVLFLMCLSFDLFSLSIYIGFDPLVPIFMLHTCGQLELISNKLVNVFS
K-------------------
DASRQDIVDDLKRINVKLQNIYKFVKMVQNIFIILYEFNMKTTTFLLPFSVFQIMQSLRNR
EISLEFIFFFSGACLHFYMPCYYSNLLMEKSESLRNAIYFSGWE--
SRDIGIRKTILLMLTRTYVPLGIKTV-FYPICLDTFAEMVRQAYGIYNIMNAAW

>HmelOR29

---------------------------MHKFEDVFKISTLALHFNGSHPQATRK-----------
FRIIYEHLISFFCFVSLLYSIFFYD--MKNEV---YTEVIKNGVMVIVCVTV--
TFKFKILLQNRDDIQRLIDSINH-----DYDLAKNLSEEEQLIVSKYANMGV-
IVCKCWVTAALATGFMFPVKAFILMGYK-----
YSKDEWELIQMFDLHWPLINGIKDNLFIFTFIFVSCFSFDAFSVSMYIGFDPLGPIFLLHAC
GQLDLICNRLKNLFSE--------------------
TTADAKENLKLINLKLIEIYRYVKTIQDIFVVLYEFNMKTTTFLIPFSTFQFVHSLREKDISL
EFAFFTICAIFHFYMPCYYGSKLVDMGEKLRQSIYACGWE--
ERNIEIRKTILFMMTRTTLPLVIKTI-FNSISLDAFGEMARQAYGIYNIMNA--

>HmelOR30

-----------------------------KFEDVFKLSTLALHFNGSHPQATRNRFKM-------
FRIVYEHIITFLCFVSLLYSIILYD--VNNKE---YTEVIKNGVMAIVCVTV--
TFKFTILLRNRDDIQSLIDTINQ-----DYELSEDLSEEEQHIVMKHANMGV-
DVCKIWLAASIVTGLLFPVKAFVLMAYK-----
YINGEWELVPMFDLNWPFINGIKKNPIIFTCIFAQCLSFDFFSASMYLGFDPLGPIFLVHAC
GQLDLLSNRFMKLFSQ--------------------
TTADAKENLKLIHLKLLEIYMYVKKIQDIFVVLYEFNMKTTTFLIPFSTFQFVESLRKKDIS
LEFASFAISAVFHFYMPCFYGNKLMDMSEKLRQSIYACGWE--
MEDIEIRKTILFMLTRTTVPLGIKSI-FYSICLDTFGEM-ASAYGIYNLMNAAW

>BmorOR30

-----------------------MSVSNLKFEVLFKPT-----TMSLHMNRSHPSIKRNKI----
WLLQFISLMTLTAFCATGLITSLLFHDLKFGK---YMEASKNGTIAMLSFTT--
TFKYSLLLYLQKSLNRLIAKIDM-----DYEIAKGLTPQEKVTVLNYAKKGV-

IVSKFWLFTAFAITFCFPLKAFIIMGYRFIIKDEFRLEPMFDMTYPE---
PIESYKTSFPVYFILFVVCFLFGCYASSLYVAFDPLVPIFVLHACGQLDLLSVRITKLFSD---
----
TKNPRIIAKELKVIIIKLQELYSFVNFIKVNFSILYEYNMKITTISMPLSAFQVVESLRRGEF
NIEFTYFFFGCILHFFMPCYYSNLLMERSENFRFAIYSCGWE--
NHNDKNIRQLLFMLTRAAEPLGIATV-FTNISLDTFAEVN--TFD--TVLLA--

>CpomOR33

---LILYSMFFHD----------------LKNND---
FAAACTNGILSVLYIAV--TFKYVVLLVKVEDITFAMNKVKG-----
DFAAAKHLCSDEQAITTEYAYKAC-WVTKVWLLTASSVFCVFPIQVIVLSIYN-----
YAIGDFQFVHMYQM-
TFPIETRKYETNMYLFLLILQTYFGVYVLLMFAGFTPLGLIFMLHVCGRIEIVKYRISKLFE
G------------------
EHYDPREIHQRLKNIVTPLQDALDFVDLIKKTFRLVYEVYMKFTTIVIPIASYEVLESLKE
GRLSIEFMTFIVAGAVLCFAPCYYSDLLMEKGLSLRMSVYTSGWE--
AYDSAMRRTLCIIMCRLERDVAIRTL-FQTVNLDAFSELCHQSYALFNVINT--

>DpleOR30

----------------------MTFALKFNRSHPSIKRDLKWMFHFGL--------------------MHGTYFAVF---
VTLVYSSISID--LKNDD---FTQATANGVLCLIFTVS--TYKYCIIWYYQDLLLDVIKKINM---
--DYEEAQCFTKEEKQIVHKFINKGR-EVNKYWFILIVTTAFIFLAKSLGLMLYY-
TIIDEFKLVHLYEMHYPK-FLE-DK-
KDTLNVFILLYIIFLFYDLYGALTYIGFEPFGPVLMLHSCGQLEIVRGRILKIFQS--------------
-----
NYTDAEIIEQLNDIAKYLQKILSFIESIKTSFKILYELTLKATAITIPITIYQIIQSLKKGELRIE
FLTVIIAGVGLSTTPCYYSEMLMNAGKDLNQAIYECGWE--
NFEPKSRKLIFLLLLLRTSKSMAVRTI-FVEVCLEALTDVM--AYAISNLLNAK-

>DpleOR31

----------------------MTFALKFNRSHPSIKRDLKWMFHFGL--------------------MHGTYFAVF---
VTLVYSSISID--LKNDD---FTQATANGVLCLIFTVS--TYKYCIIWYYQDLLLDVIKKINM---
--DYEEAQCFTKEEKQIVHKFINKGR-EVNKYWFILIVTTAFIFLAKSLGLMLYY-
TIIDEFKLVHLYEMHYPK-FLE-DK-
KDTLSVFILLYIIFLFYDLYGALTYIGFEPFGPVLMLHSCGQLEIVRGRILKIFQS--------------
-----NYTDAEIIEQLNDIAKYLQKILRS---
IKTSFKILYELTLKATAITIPITIYQIIQSLKKGELRIEFLTVIIAGVGLSTTPCYYSEMLMN---
---AV---------------

>HmelOR4

---------------------------LNIKFENSFQFM---LRAMYISQFHPYTKKDIKWA----
SKLLSMYLTCLAVICIIVYSIIFID--LKVKD---ITQACSNGVICLIFSVI--
TLHYCIMFYYQKLIVNMIEIMNN-----HYEKAEEYTVEEKHVILEYVEKGK-
FIVIIWRRISLFGGGLFITRPIFVMVYY-AIFSEFKPVHLYEMVYPS---

PIEELKDTFSVYCFLYITFIIFTCCAMFTLHGFQPLGPIFMVHACGQLEVARTRIMKIFHD---
----
DNSVEEIIVKLKHVAVSLKNIYSFIDKIQTCFKIFYELILKTTILVLPIVIFQILGSFSKGELKI
EFLSIVVGAITLSSVPCYYSGMLMESGENLRLAIYSCSWE--
RWDRTSRTLILLLLLRTTPPVTIRTM-FCRLSLDALADVFRQAYTIFNLLNA--

>SlitOR5

-----------------------MTNQKDLDFENIFKIT-----TTALHISGSHPSVPKDLK----
WALKFTILHGTFALGFVTVIYSIIYHDLKEQN---FVQICKDCVVFVLFCVT--
SLQYCVLLIHQDNLVLLIKNINS-----DYEQMQNLSDKEKQLMDKYMNLGA-
KVCRHWFVLVLVTCLIFLIKSVGFMCYY------
YLINDIKYIPYYDIKYPEERKNENFSVFLITYCLMFYFALYSVLIYVAYVPLGPVFMLHAS
GLLEVVKNRIDDLFSD--------------------
SNPEKIREKLKDVVMKLQYIYSIVDDMKTIFRFGYEVSLKGTAVLLPVTFYAVLEAAKN
GEISMEFISFIAGGIVISAVPCYYSDLLMEKGEAVRLSLYMCGWE--
QHDRRTRTTLQLMLLRALRPIAIQTL-FRTLCLDALTDLFQQSYGIFNLMNAMW

>SlitOR30

------------------------MDATCLNFEELFKIRTVFMRLNRS-----YPSIARDRP----
WKFQFSIIMMIIFSHFFFLSYSVFYHDIRSGN---FAVACLNASVAIICITI--
TLKYIILLYHQESIKQLKIAMER-----DYAAAQEFCNEDKEIVVQYAKRGV-
TVCKFWLVFGFGTSTIFPIKALVLTGYN-----YWKGEFVYVHVFDL-
IYPIEDYKNVTICFWLLFIFNNSFGLLASSMFVGFDPMVPIFMLHTCGQLDLLS---------------
-----------------------------------RHIS--TLF---------------
VDANI---

>BmorOR33

-----------------------MIYYRKCKMELNFDKI-FKIAIISQKFSGTYPYTKRDKK----
WATHFILMHGELTIICMLFIYNIIEFDLKAAD---YSQMCRNMCLSFVYMVI--
TLLYINMLYYQSKLKMLIETMKA-----EYELAKTMSEEEQNVILEYAKKGR-
WLCRAWAILTTCGMAQFFLKSIVCTIYS------
AIQGNFRIVQYYEVICPIERHRNNPVIFITLYFCTFFYSLYTSALYTSVLPLGPIFLLHGCAK
LEIVRLNIKNLFDN---------------------
DDYVVQERLKKTVLQMQDIYCYSHEINECFQILYEFLLKATSLVLPITIFAVIQALGRGQFI
PEFFAFIFGAFMVGTTPCYYSNMLMEKSEDVRMTLYSCGWE--
RFDLNTRKCIILMLCRALRPVSIRTI-FRSVSLTTLTDVFQQAYALFNLLNAVW

>BmorOR34

-----------------------MIYYRKSKMELNFDKI-FRIAIISQKFSGTYPYTKRDKK----
WATHFILMHGELTIICMLFIYNIIEFDLKAAD---YSQMCRNMCLSFVYLVI--
TLLYINMLYYQSKLKMLIETMKA-----EYEIAKTMSEEEQNVILEYAKKGR-
WLCRAWAILTTCGMAQFFLKSIIVCTIY-----
SAIQGNFRIVQYYEVIYPIERHRNNPVIFITMYFCTFFYSLYTSALYTSVLPLGPIFLLHGCA
KLEIVRLNIKNLFDN---------------------

DDYVVQERLKKTVLQMQEIYCYSNEINECFQVIYEFLLKSSSLVLPITIFAVIQALGRGQFI
PEFFAFIFGAFVVGTTPCYYSNMLMEKSEDVCMTLYSCGWE--
TRDLNTRKCIILMLCRALRPVSIRTI-FRSVSLTTLTGVFQQAYALFNLLNAVW

>MsexOR38

--------------------LSLPLQSSHVTMDLNFDKM-YILTVFAMRINRSHPSIAKDTK----
WFLQLLPMYGIFSIMFALLINCIIHYDLKAKD---FSSTCRNGCLCVLYFIS--
TLSYYVMLVHQTTLKTIINTMNE-----DYAQALKFKADEQKVVLDYAKKGL-
YVCRQWLAMSIMGVGLFFVKNVLLYAYN------
YYVDDMKLVPLHDMTYPIEEKRDDIIVYASLYALTVYYGVFAAIMYMSFVPLGPVFML
HCCGQLELIVMRLENLFIK---------------------
THEEANEKLKDIIRHLQNIYGFVKNIEKCFTGFYELTLKATTITLPIAVYEIIESCHRRELRM
EFLVFIFGAMIISNSPCYYSHLLMENGEQVRLAVYCSGWE----VVPIPRRLALL---------------
--------------SW----------

>SlitOR38

---------------------------MADQFEKTLKLFRLTLTSPEIPITFKW--------------
LIRYLLGFLTLTALAIVITYNTY--ISITN-RVFFEACRNITLSLTYYGC--
CMNNILSFWHRSSLRALLETIRH-----DYKMAAQLPREEQLIFEDYDRKKC-
LMCKIWFHLFTVSLFLFVAKAVVLMIYY-----YFIGEFRLVHLYEL-
TYPIEKRKGELVIYLFIFCIVFLYGLLSGLGFASFVPYGTICMLHACGHLEIARKRIDSLFTG

DRRYVNEKLKNIAQLLQYTYKFMENTNGCFRLFYDATLKLSAIAIPFTLYALLEG-------
LRHGEFSMFVLNAIXQYTLLPKXXIIKGEEVRLALYSCGWE--
CEYNRRVRIILLLLLTCCSRPIAMQTM-FTTLCLYAVTDMFQQAYTILNLMNA--

>DpleOR13

-----------------------------MRNPETYRSL---------------------------
IAPFWILHFSLLVYVYGAGSVMYQL--RVASS---TRDFLKSYVNITLIILI--
VNNSYWFIKERPLLRTVLNQVIR------NDILSMETELLQDKHEKLLSVIK-
KIILIFYGFNLFDAFF-IYIPHRSDVNN----------DHYSMTSCVGLEP----
LTATPNRQICRLILGLQEVTIMTAVLNYQTLLLFLIAHTEAMYRLLSEEMLNFNQY---------
-------ASTPESNIFVKNRLPLLVSRHSLTLDIIKNLKALYSMPMGVNFGSNAVCICLFFYL----
--PLREWITFSPVLIYCFLVFFLYCYLCQRLINASEVFENAVYSCGWE--
KFNLNEQKTIYVMLLQAQKPVTLLAADIIPVNISTFATTIQAMFKFVTVVKL--

>HmelOR54

--------------------MKFIDNIKDYFIETRFQLRENIIWLVNLVPSKAGFSILKDKI----
SAPFWILHLSLLFYVYGVGSVVYQI--KFAQG---ASDFIKSYVNISILILT--
ANNSYWFLMKRPLLKVVIEQVTR------NDSLSKETVFLKDKHGKLLSVIK-
KILFIFYGFNLLDALF-IYIPHRVDVSN----------DYYSMTPCVGLEP----
LTMSPNREICHVILFSQEISIMTVVLNYQALLLFLIAHTAAMYQMLAEMLAFEEY---------
----
ENSDINIVVKEHLPSLIERHALTLSIIDNLKALYSMPMGVNFGSNAVCICLFYLSLHEW-----

--ITFAPVLIYCFLVFFLYCFLCQRLINASEVFENAVYSCGWE--
KFNMKEKKTIYVMLLQAQKPITLLAADIIPVNIYTFASTLRAMYQFITVVKL--

>BmorOR54

-------------------------MGLNTIKEFFVNVKRRFQDVSIDSLLWIVNIVPSLAGFSI--
PFWIVHWSLLVYVYAVGNAVYQW--KFANE---AIDYITSFINVSLLILI--
GNNSWWFLANRRLLKSVLHKIEV------NDELSRRSEQSRLKHKKLLKIIK-
RIVLVFYMSNYVNASFIYLPNRVDVLNN------------YAMTPCVGMEP----
LTVSPNRELCLTILCMQEFSIMTVVLNFQALLLCFIAHTAVMFQILADEIMALNNY---------
-------ENLEEHQAYVKEMLPIFVKRHSLTLSAVDNYKSLYSVPLGVNFGSNALTILLILYL--
----PVLEWFKFIPIFVFCFMLFFLYCFLCQKLVNASEAFETAIYCCGWE--
NFALREMKMIYVMLHQAQKPVELLAADIVPVNMNTFATTLQAMYKFVTVVKF--

>DpleOR12

------------------MSLIDRFKTKLVDYKETFMDYSIILLLNFIPSMAGFSILNKKN-----
SVIFWIVHFFLVLYVYVLGSIMYQV--NVADG---PIDLIKSYVNLTLVILI--
INNSYWFIVKRATLTKLMENVIE------NDTQSHQLDFLKPKHTKLLRTIK-
IIVYIFYAFNILDVIF-VYIPRRIDVTN----------EYYSNVACVGMKP----
LTHFPKKQLCQLVLFATEISICTVVLNYQALIFFLISHTAAMYRLLCEEIFALDDD-----------
--------NMDTATVKKRLVVLIGRHTLTLTITKTIKDLYSMPIGINFGSNA--ICIISFA-----
LPWEEWVNLSPVYIYCFLVFFLYCHLCQMLINASEQFEYAVYSCGWE--
KFDLKEQKTAYVMLLQAQKPVTLLAADIVPVNIYTFATTMQAMFKFITVFKF--

>CpomOR36

------------------MECFSRAQDGFHRLKKRLRENLVCLVMVMPSLVGFEITRKKI------
FVPFWIIHLSLLTYVYGVGSLVYQAKHARVAS-----DFIKSFVNVSILVLT--
VNNSYWWLTHRDLLRNVLRKANA------SDKMTIQAGLFVDKHQHSLSIIK-
RILIIFYFINLTNEFT-SYLPKRAELNE----------KTFSMTPCVGIKP----
LTSSPQREVCIVLTSLQELTIVIVVLNFQTMMLLLIAHTSTMYQLLSDEIMTFNTI------------
----LTNPSNYDLLKERLGVIIKRHILTLDIIKDIRVLYSIPMGINFGSNAVCMCFFFFL------
EPEEYFNFMPISMYCFIVFFLYCFLGQRLTNAAEVFSRAVYSCGWE--
LMDIKEQRAISIMLLQSQKEVDLLAADLIPVNMLTFAST-SQIYKFVTVFKL--

>BmorOR55

-------------------MCFLKIKQQIIDIQKHFKDYSLWIVNLLPRLMGFNLRADKVGV------
FFWTIYILLLVYVFGIGIFVYLW--KHVDT---MSGLMKSYLNLSLILVI--
VNNSCWFLSKRSLLNKVLKKIHL------
IEDLSCESEHALAKYRRVFKIVTHLLLASYVLFYFTEIYFMFLFRNYDLLED------------
YSLAPCVGLEP----
LSSSPNSEICLIIVLIHEFISTTVMMSFAALFLVLIAHTAVMFLVLAEDMTKLTDL------------
----INLADHRKMIRESLRSLIHRHSLLLQIVYELRLLYSVPLGINFISNASILVLLCLPIHEW--
-----PSFLHIIGYCFFAFFLYCFLGQNVINASEKFIDAIYCCGWE--
HFGVAEKKLVHVMLRQAQKPVEIIALGMISVNMNTYVEALQLIYKFVTVLKI--

>SlitOR4

--------------------MVNTFRGRLLNIRDRLKQNNLVWLINFLPNLAGFPLLSQKF-----
KFFFWIVHTVLLFYVYLLGTAMYQT--YFAED---FVDSINSFFNISVFILI--
GNDSWWLISKRNDLNDLLKMVKK------
NDDLIIESGRFQDVHQKLMKSIKIIVIMCYMFHFVNDVMIF--IPSRTIGMD-----------
DFSTVSCVGMEP----
LTSSPNRQACMVVLALQELTAIVAVCSYDVALLFLFSHTTAVFQILYQDMTEFANI--------
----
SKSHETYYVVKDRLKNIVFRHVLALHTVRKLEDIYSVAIGIGFGLDAISMCLFVLPLDVC-
------LNFAPLIYHSLFIFFLYCFQGQRLTTASEKFEMAVYCCGWE--
NLRVKERRQVLLMLKQAQKPVIVYAARVIPIRIHTFASTMQSIYKFVTIFKV--

>DpleOR33

------------------------------MYPNPVYDE---------------------------
KRKKLIVMAILSVLPAFTLAGNDIRLRILNND---MANVVRQSIILVSITFV--
IIKFIISIARKDEFRSLLEEIDA-----DYERFNGMSEQYQDLVEDTIRKTR-
KVEKSWCFVLLVTTASYPVLAGACTIYS--QLFSDYPRRYMVHELKA-
ILISEEQKYQSPYFEIVSLYTMYIVIFLFIGFTGFDGMFSVCLLHVSLKLKIYQENLRNLFNE

KDIQKIKYNIGLFVKNHCGVLRLIAKIQTCFEVWLVGIFINAVVQIGMAFTQITNQ-
TESDINQMYYLYALATVVHIYLPCYFASDVTYNAAEIANVAYSSSWE--
RVDSKIRSSICFIIAKCQTPVRLTALDMLTFNMELFVSV---GHQM--------

>HmelOR14

-------------------------------YTTFVETYKIISFIMGVGMIYPNPKTEKRK-------
IVLLMLISIGPISMMILNDMFNC--LAMRD---YVNVIRHSTVVGPFLGG--
FFKILIQFYKRKPAKEIVDEINR-----
DYASYNDMTVTYKKMINASIENNLFYSEKCWAITVVTCVSAFPIMAVLSNLHSTLFAAE
PKRYMVHDIILPY-AEPEKR--
FESPIFEIMFAYMLYASVLYIINFIGYDGFFGLCINHACLKMEIYCRSFEDALRE-------------
------DNESCVHLKIVNIIVEQNKLKRFVDLIQDTFNIWLGLILVATMIQIGTCMYLISEG---
YALDLRYLIFIIGTVVHIYLPCRYAAKLKHMSMETATLIYSGGWE--
RLSIRIRKMVLFMIARAQIPIEITAFNLMIFDMDLFVTV---SFMFIPLL----

>SlitOR41

--EMWR-------------
LASIPFLIATITPLATMIFIDMYKCWMAKD---IVNIIRHSTVVGPFLGG--
FFKMILMYHKRIQAKQILDEIDR-----
DYENINNYSEIYKDIARASVKNCQIYSERGWAITVVTCVMTFPVMAISLNVLQSCFKSEP
VKYMIHDLEKPFSEDPEDR--
FESPYFEIIFVYMFYCSILYVVNFTGYDGFFGLAINXACLKMDLYCKALEEASKL-----------
----------DAHEVCGRVIGVIKEQCRMFQFVDLIQDTFNIWLGIIFLATMIQICTCLYHINEG---
YGFDLRYMIFVTGAVIHIYLPCRYAAKLKAMSLETANRFXSSGWE--
QVDQRVRKMILFMVARAQVPNEIVALNMLAFNMELFVSILQTSYSMFTLLRS--

>HvirOR20

--------------------------EFKPFHETYKVI-----TFTLCIAMIFPNPRTEK-----
WRLISIPLLIATVAPVASMIFLDMYKCWTNND---IVNIIRHSTVVGPFLGG--
FFKMILMYHKRKEAKQILDEFDR-----
DHFMFNDFSETYKDIARASIRNCQIYSERLWALLVTTCVMTFPVMAIVLNIYNFLFKSEPT
KYMIHDLEKPFSESPEER--
FESPYFELLFAYMFYAAILYVVNFTGYDGFFGLCINHACLKMELYCKALEEAMVA--------
----
DREEVYGRVVAVIREQCRMFRYVDLVQETFNIWLGIIFIATMIQICTCLYHITEG---
YGFDIRYMIFVYGAVVHIYLPCRYAAKLKAMSMETSNRFYCCGWE--
KVDERVRKMVLFMIARAQVPNEITAFNMLAFDMELFLSILQTSYSMFTLLRS--

>DpOR20_zhan2011

MMWNVEVLLRTLTIRSNRRFDKPIPWIYYVINISTGLCYFYTYMLSTAWFVLVRCRETG
DFISAMVALSLTMCSITCIPKIFYMKYYQNTVMDLVDNYLHCNELIVSGTRFEKNFFANM
RTVKKQATIAWVILTLNGVVFVTLPFLRPGRHFSQDLFALYGLDPMVESPNYEKASILLT
LAVYFSLTVMVHITLFIIVIIGFLESQMLALSVELSNIWEDSKKFYNYYVLHHNESITKYEL
RNLFIKNRLRDIVKFQIMIVSLRNKVEKELRFIFVNDFIFMIFALVSELVGGLENTMVQMP
FTFGMVFIDCFIGQRLIDASKAFERAAYGSKWENFNLSNQKTVLFMLQCSQKTMTLTAG
GIAVLDFIYLMSVLKSTYSAYTTLKSSA--

>MsexOR7

-------------------------MDEPEFKPFHKTYQIITFALSVGMIYPNPATDKMR-----
LASIPISILTILPLACMIFLEMYQC--WTQGD---IVNIIRHTTVLGPFLGG--
LFKMFLMYHKRKQAKQILDEFER-----
DYHLYNSFTGDYLNIARDGIRNSLIYSERGWAITVTTCVMTFPVMAILLNMYNYTFKFQ
ATKYMIHDLNKPG-ASPEAR--
FDSPYYEIMFFYMMYCSLLYVINFIGYDGFFGLSINHACMKTNLYCKMLEDAWKA--------
-------------EPNERYRRVVAVIDEQCRMFEFVNLIQDTFNIWLGIIFLATMIQICTCLYHITEG-
--YGFDLRYMIFVSGATIHIYLPCRYAAKLKAMAAETATHFYSSGWE--
RVNDRRLRNLLFMVARAQTPLQITAFNMITFDMELFVSIMQSSYSMFTLLRS--

>BmorOR14

--------------------------MSNYIFKPFHETYRIITFTMIAAMIYPNPATEKRR------
LIYIGLMLLSVIPLAFMIVTEMYEFFMASD---LNNTIRHSTVIGPFIGG--
FVKVALMYYKRRQANELVSEINR-----
DHLAYNGLKGEDREIAASSIRNCQIYCELGWTLIVMSCGLSFPVIAILLKIHSFTFKLDSTK
HMIHDINNPFTDDPEDR--
FESPFFEIMFVYTFFSSFIYIINYVGYDGFFGLCINHACLKMKLYCRALEDAMRS-------------
---------DSRRHEKIVAVIEEQRRTYEYIALIQDTFNIWLGLIYVATMIQMCTCMYHIVQS---
FNIDVRYIIFV-ISIIHIYLPCRYAANLKCMAAETPTLIYCCGWE--
SVDLRIKRMMPFMVARSQVIVEITAFNMFAFDMELFVWIMKTSYSMFTLMRS--

>CpomOR20

--------------MKSFRRKESSTLPLVPVTEELEFKTYKIITFTMIVGMLYPTPNTEV-----
CRVVGILTILVTMSPVCIVALLDMWNSWFRGD---IINIIRHTTVIGPFLGA--
IFKMMLFFYSRKEAWSIIKKMDS-----
DHARYNTLPEQHKEIARRHIQNTQYYSEKCWSITVATCVLTFPLTAVVLTFYN--
YTFKENPVKYMIHDIDKPFSPRED-
RFTSPYFEIMFFYMGYCSLFYIISFTGFDAFFGITINHACMKMELACKTMEDAMLE---------
----
RDRDSRHRRMLDVISEQNDLFRMVELIQETFAIWLGIIVIATMLQICNCMYQIIEG---
YGIDPRYLVFIVGTIAHIYLPCRYAAKLQVSALEVATHLYCCGWE--
RVDERARKMIVFMIARAQIPMKITAFNMFDFDMELFVSILQTSYSMFTLLRS--

>Bany_OR6_GZXD38A01B02O1

EVWLVGIFFNA--VVQIGMALCQITSN--
AEADINAMYFAVATAVHIYVPCHLASDVTYHAAEVATVAYSSSWE-
SVQDAGVKRSIAIIIARAQIPINFQALGMLTFNMEMFVSILQTSYSMYTLLRTNI

>BmorOR14_ABK27845

----------------------------------MKLYCRALEDAMRS--------------------
ESRRHEKIVAVIEEQRRTYE--YIALIQDTFNIWLGLIYVATMIQMCTCMYHIVQSF---
NIDVRYIIFVI-SIIHIYLPCRYAANLKCMAAETPTLIYCCGWE-
SVSDLRIKRMMPFMVARSQVIVEITAFNMFAFDMELFVWIMKTSYSMFTLMRS--

>CpomOR14

---------MDATKAFRTSDSSAVLAPVFEDMEFKPFRETYKIITFNMIVGMLYPTPETAVC-----
-RLLGIVLVLSISPAALIALLDVWHSWQRGD---IINIVRHITVLGPCLAA--
IFKMMLFYYTRDEAWRIIRKIDA-----
DHARYNILSESHKEIARRHIQNTQYYSEKCWAITVAVTVLTFPLTAVVLNFYNFVFKEEP
VKYMIHDLEKPF-SPPEDR--
FASPYFEIMFGYMAYCSLWYIISFIGFDAFFGVTINHACMKLELACKIMEDAMLE----------
---------EDRDSRQSRMKEVISEQNDFFSMVELIQETFNFWLGLIVIATMCQICNCMYQIIEG-
--YGIDPKYIIFILGTIAHIYLPCRYAAKLQVTALDVATHLYCCGWE--
HVDERARKMVAFMIARAQVPLKITAFNMFYFDMDLFVSILQTSYSLFTLLRS--

>DpleOR32_EHJ72221

----------------------MIYPNRENIQTRFKVIFSVIAINGV--------------------------------
MLYWFLLYLWKCICNHD---MYNVSRNLTIGILVTLF--FFKSFYVTNKTHRFAQLLEKISN-
----DLLKGNDMDDSYQEIYEKYIKLGK-LGQTCWIFIPILLTSQFPIYAGVSTIYE--

SLKTDTGQKYMIHEMDL-KY-
LEDKQYDSPIYELTFAYHLITCLTLSPNFAGFDGSFCIATNHLRLKLKLLAHKVERAFKD--
----
AKNKWELHTMIKETIRDHQEALSFYEDLQEVYGSWLFTVFAVTSLLISLNIYQIYLS---
ERVDPKYAIFAISGIIHMFAPCYFASNLMKTSEELSLDLYSVEWE--
VWYPAVTKQLIFMIAKSQQTLILTGNGIVYFNMQLFISLLRSAWGVPGFIYK--

>Bany_OR7_GZXD38A02GO9NU

-IAMNLYQL-YLSDHIDMKYTIFAVSATSHVRAVLFTCNLIKVSEDLSWDIYDVPWE-
TWADPSVTKLLIFMIAKSQQTLILSGNGIVYFNMQLFISVLQTAYSFFTLISR--

>HmelOR66

---------------------MDKSEKKFKCFNETFPRCTFALAFSLTYPNPTNIK----------
KRLIIFSIAIFVNSGMLIWFILYLFKCIQTRD---IYNLARNITIGVLVLLF--
FSKMYYTTRNAKKFARLLEKITI-----DLLLGNDMAEEYQMIYEYYIKIGK-
HGETIWIIIPILLSSQFPIYAGMCNMYEKSDVGKKYMI----HEMEL-KYIEDI-
QYETPYYELLFAYNIINVILLSPNFAGFDGSFCIATNHLRLKLKVLAHKVCEAFRQ----------
--------AKS-
KAELKEKLKGAIKDHQEALEFYDDLQEVYGGWLLVVFLLTSFLISLNIYQIYLS---
QQIDTKYSMFAISGIIHMFTPCYFSSNLIKTSEELSWDLYNAAWE--
DWDPAVRKLLIFMITKSQQTLVLTGQGIVYFNMQLFISVLQTSYSVFTLITS--

>SlitOR18

----------------MEMKSDILTQKKYKTFNDTFKLCAFSLAFAFL-----YPNRNTALK------
RCITITLIVFCGGQLFWFITYTFKCLYTLD---LYNFARNMTLAVVLILF--
FIKTYYAIYATQKFATLLDKISE-----DLLEANNLGERQQKLYDEHITIAK-
FGEISWLLVPVLMSALFPIYAGTLMSIESIQTDDYQRRMVHDMELLY----
VEDIQSETPFFQCMFAYNCVQCVVLVPNYCAFDGSFCIATTHIQLKLKLMVLKVHDAFK
Y------------------
SKTKYELRMKMNEAIRDHQEALDFYVQSQNVFGPWLFAVFMLTSFMISFNLYQIYLL---
QRIDPKYTSFGLVGVLHIYLPCQYASSLTKVSEEIPNDLYLVDWE--
AWDPKVTKQLIFMITKAQKEMILTGMGIVVFNMELFKSIMQTSYSFFTLITA--

>HvirOR18

-----------------------MEMKVDVLPEKKYKGFNETFKLCAFSLAFAFLYPNRTTA----
LRRCITITLIVFCGGQLFWFITYTFKCLYTLD---IYNFARNMTLAVVLVLF--
FIKTYYVIYATSKFAPLLDKISE-----DLLEANNLEEEFQVLYDDHIKIAK-
VGEISWLLIPTIMSALFPIYAGALMTIE----
SIQTDDYERRMVHDMELLFVEDIQSETPFFQCMFAYNCVQCVVLVPNYCGFDGSFCIAT
THLRLKLKLMTLKVYKAFKY-------------------
SKSRQELRVRLYESIKDHQDALDFYVQLQNVYGPWLFAVFLLTSFMISFNLYQIYLL---

QRIDPKYTSFGVVGVLHIYLPCRYASDLTRVSEEIPDDLYLAEWE--
AWDPSITKMLMFMITRAQKEMIVTGMGLVVFNMEMFKSILQTSYSFFTLITA--

>CpomOR18

--------------------MNQSNCLKYKSFNETFKFC-----SFALALGLIYPNRKNVC-----
LRTTIFFFVLLFNFGTLFWFIWYTVKCLWELD---IYNSTRNITVGVIILLF--
VFKTIYVNLKTDMFASLLEQITK-----DLLKGNNMDEDYQEIYDYYIKQGL-
FGQKCYVWIPLILTSIFPTYAGISMTYGSLRSDDFKKVMLHEMDLKY----
IEDKQYDFPYFELVFAYYFLGIYILIPNFAGFDGSFCIATSHLRMKIKLMTHGVQRAFTD---
----
SKDILELKARLKTCVKDHQEALEFYTLIQRLYGGWLFAVFLLTSFLISCNLYQIYLT----
GIDPRYTMFAATGVFHMYTPCYFASCLIELGEQTCTDIYCAKWE--
SWDPTVTKFLIFIMARAQKRLLLNGLGIVFFNMESFVSLMQTSYSFFTLITSK-

>SlitOR25

------------MGLIKNLCLKLSYTKAIDRSSGRLETIFFENYYRIAYMAGMSTADDNIGY------
LVYSNTVKLMIVLLVFGVVWYGF--METTS---FDEFAGNLNVSLLQFIT--
FYRYRNMLAHEKFYKELASSMES-----PYFDI--STEQRKKLVEFWSRTNV-
KYLKLLMGLGNCTLVAWFIFPLLDDIEY-------------NLIVGL-YLPFFY--
KSPSIYPLAYMLSVIFFFYISHFVMVTDLKMQTHLIHLLCQFSVLADCFENMIPDV----
TGFEGVPRNHLMYNNHFAAKYTERLGNLVKQHKALLGHAMNLRDTLSGPLLGQLAAS
GVLICFIGYQATAT-
IGQSVVMTSFLFLAYNLFDFYMICRWCQEITNQSANVGEAIYCSGWE--
CGKLPGVRSIMFVIARANKPLVLTAGGMYDLSLTSYTSLVKTSYSALTVLLRFR

>MsexOR25

-----------MELCKTIWRIITPTKALQQSSGHLETLFYRVSYLMGLSTSDHDM-----------
FYLLYSTTVKFMITLLVCGELWYIF--TETSS---LDGIASSINVTLIQFIT--
IYRYKNMMDHKDIYKKLATSMES-------PYFDTSNEKRKQLVVFWAKRNE-
KYLKLLLFLGNCTLAAWYLYPLVDDIEY-------------NLIIGF-HTPFSF--
KTPLRYPVVYLVVVIAFTYISHFVMVTDLIMQAHLIHLLCQFTVLADCFENLLDDQ----
HGFEDVPRNMLVNNKQFAAKYIRRLGHLVEQHKKILKHTVNLRNTLSRPMLGQLAASG
TLICCIGYQATTT-MTESIVLMSLFYLGYNSFELYIICLWCEEITTQSMNIGDAIYCSGWE--
GVKLPGVRSIMLVLARANKPLVLTAGGMYNLSLTAYTTLVKTSYSALTVLLRFR

>BmorOR64

----------------------MGVSNGRGTVKPFLYPLDYNLIVGVHLPFEYKTPSRYP----------------
LAYITVVIAFIYVSYFVMVTD---LIMQAH-------------------------LLHLLCQFNV------------
LADCFENMLNDCVKGFE---------GPLVSLHEY-IHPLIDEFEY------------NLMVGL-
RLPFSF--
DTPLRYLFTYVIVLIAFNYTAHYVMVTDLIMQSYLIPLICQYAVLADCFENILID------------
------------CSNDYGDHARRNDIVYSRSMELRAILSRPMLGQLASSGLLICFVGYQATTS--
ISVIVLMSLFYLGYNMFTLFVVCRWCEEITNKSLNIGNAVYCSGWE--
GMTVVVRSTILLVILRANKPIVFTAGGMYNLSLTSYTSLVKGSYSALTFLLRIQ

>DpleOR54_64

MSSSLDDIIEGVNVILIQIGTYVKYKTMFFYPLFDDAEY------------
NLIIVISLPFDYRTPALYPVTYMSTFIVFMYIAYFVMLHDLVIQVYLVHLI---
CQFAILNDCFENILID---
CKKGFSKEQLDSIYFNEEFIQKYIKRLGRLVDQHTFIINNTLKLRDILSTPMLLQVAVSTTL
ICSIGFQIATTSVNVSKMLMSLLYLGYNMLSFFIMCKRCEDLKIQSTKVGEAVYFSSWEV
VVTIPRVRTTVLLILLRAHKPIGLTGGGMYELSLEAYGNMIKTSYSALTVLLRLR

>Bany_nBa.0.1-g11159genome

MDVVYNNLPFQSEGIGEAVYCSGWEGIVMVPGVRTSILLILARANKPVRLSAGGMYDLS
LEAYATCDLRGPESKLVSDLAP

>SlitOR24

--------MRVLSHVWRRISQSKALELAGPLETAFFASVYRLSFVVGLSTSDDY------------
LLYTMYSSFIRIISALVVWFEIWSVLGNTD-VSLDQIISSVNVIFIHLVT--
FWKLVTVVKNKRIFKKLAKALES-------SSFDMSTERRKGIVNDWILINN-
KYLKVVLTLGCLTLIVWESYPMIDELKF-------------NLMVDV-KLPFEY--
QSLLTYMVTYTAVAIMFAYASLMVIISELMMQAHLIRLICQFDVLADCFENIFEEA----
EEFPDLKKHELVKDKTFVDKYVKRLGDLVAQHREILDQTNDLRTILSAPLLGQLACSGL
LICFVGYQATATAENLGKFVMSLLYLGYNMFTWYLMCRWCEEITIKSQRIGQSAYFSGW
E--SLAPGARATIVLVIARANKPLVFVAGGMYTLSLSSYTTLVKASYNALNILLTMS

>BmorOR50

-----------------------MEGTSEGDEPFIYR--------LNDNGTGPNLVVKIL----
YCPCWYTAHLMPFKLHYCFTAEIVR--LMIVP---
LVDSQDVLPPVITQIVISIFIRCYSFIVEVNREHLLMQETLL-----------
KPARRDPKIANYNMVRNDRLSARGGGTVIYYRRALHCVPLDPPALA---------
NIEASVCRPI----FAAKTLKDKQHLIQNKKE-------VTRFARLLLTYVTVGGFIWP----
MSFCFRRIKDP---------------------NTVVPFYVPFTPDNWTKLI---
NEVTDIFNPCLTFQFFTSSVAICMVIYKLSDT-
YIVLEFVFLLNFIFVLLTQMFIYCYYGNVVSYESKYINTSLYLSDWS--
SASPGVRKMFLIVMPRWTRPLVVRIARVVPLSLDSFVSVYKCSIEHFIYSHVK-

>DpleOR59

------------MGSLLKWLFNPRTAYIKKSKASVKDIIDNLFQDSGVLLGKEQT-----------
TVPWKVKISIFCFLVCNLLQLIALI--LALGD---LDELLACSGRLTFLTMS--
VPKLFSIFLSKKLKRDLAYEYESEDE---------EDDYLTEHIDSYTKQFQ-

GFAKILSSIYFYAANVYVLSPFIEYGYH----VYKGNDRMYPHILPG-WSPQDA---
NVISYSVLIVAEVIAAEYCVKLHTVYDVTAVGLMIFIRGQFLLLHQFSEHIGGR-----------
GNDLSLSRRRDDRAHCRIKKCHYIHVSLLKSISDLDDLIRNVLGIYFCIAAMTLCSAAVQL
LKW-NSLDAMVSLLQFPIATLIQLYLFCLYGDGMFNESAVMSGEGFRAARW--
CLSKHIIQEIAILCVSMSQSRRINAGPFVTVDLPTFVMIVRGTYSVYTVLTQE-

>BmorOR32

QERGRPPGLEGEYVANRPFRKSLRGSQEEPRANGKSENVNSHILHCGLRFNETNC----------
-HTHYIAKVAIFCFIVTYMLQVMELY--WSKGD---QEKLFECFSILSFCGMG--
VMKLVILRVYHQRWRFLLNQVSILENRHLDPGPLSDDNEIVTFITKYTDKFK-
RTSSILIKMYASTLVIYVLSPFVEYIFR------QFLNIAYPHILPA-WTPLDE--
FSVTGYLIMVSFETVACIYCVFVHVAFDLTCVGLMIFACGQFYLLRYRSERIGGK-----------
-
GRICRLLKSTEVRAHYRIVFCHGIHVLLVQLIEELDRLIKHILGVYFFLATLTLCSVAVRLK
TEDMSITQLVNLLQYMCGTLTQLFLYCKYGDSVYNEAYGLPDACLESGRF--
QWKTGYRPI--VLLDEAGKLLERVVAARIVQHLTGVGPDLSAQFGFRSTIDA--

>SlitOR23

-----------MWQKLKEFYNKTHTDFSKGYIDPYEFHKTFYQIMVNFKVADLSNENPPSY------
FNQNAIIFMTGFVAKLLCISSFYHGLMTFN---LRLATEAGTYIIVMAYA--
LLISSCTRRNVPQYHNFLRAMKEDFQFICTSG---
EKYRVPYFSNQLLTWKICIFACIFTASIAVGMVSFAFLSLFYFLAT----
YKEEIGGSRPLLFPF-WLPNVDF-
GETPVYEIAFMFSNICALLYAYNYIFMIQTQIVWIRQITSKVDIVIWSISDLLVD---------
IHPAGSEEESVYFSYLIKARMRDILLHHQSMYSLMEDYAVVYKKMLMFEQKCCGSVVC
LTAYCIAEAFDAGEFQAILLLLCIGTTVLHFVPCYFCTFLAVKVSSVCDACWNIPFW----
AGPLIRPMVLIMQRSLRHLPLQAAGFEDISIETFSKKMTNAYSLFNMLRQAN

>DpleOR37

------------------------------MDETFQVFDRVLSFAGISLFAKRNWNSKKW-------
LFFQCFNVIIGFITFIFTTAFVF--TNISN---LLLCIQGGCVWTTGVIM--
FISLCVCLIFRTKFRTFLEEMVFEDRVFDIPLVKSELEKLVVNSQEKLFKLTRVLLKTYVG
SVWLCVTLYLCGPIYQMCVT-------KDKSLRLLAFDM-WFPYGL--EDFRVYVATFIFH------
AYAAYPGLQSTIILFVGQIIRQLQILTFILNNLDEI----------
AKELVGKRGELWQETCIQIFSQCVDHYIQTKRFSNKLNVICQPFYLALIVVATMLVCVCS
VKIAISDKLSPDTMKYYVHELCFVLVVLMFCYLGQQVDNECEKLEAAV-TEKWY--
LFNSSHKTNIRIFMMAMCQKMPIYIYGSITLSLQTFTWFIRTGMSFFTFLMS--

>HmelOR3

--------------------------MMYEYDTFVTFD-RILSFAGIPIFAKHKCNSKKW-------
LLFQIFNFFLAFLTFVFTSGFVL--SNLSN---LELCIQGACVWTTGVIM--
FISLGVCLVFRLQFRSFLEEIIFNDSLLEMPFINGELRKSVINSRETLFRLTRVLLKTYIASLG
VCATLYLCGPIYLMCIR-------NDKSLRLLAFDM-WFPWGL--
ENFAVYVASFVFHAYVGYLCS--FAGLQSTLVLLVGQIIRQLKILTFILSNMDDL----------

IKELVGERGDKWQRECTTLLTQCVDHFVKTKRFANRLNAICQPFYLSVILVAMMLMAV
CSVKIAISDKLSPDTVKYYIHEICNILIVLMLCSLGQQVENECEQLEMAV-TDKWY--
IYNSKHRVCVRIFKMALSQRMPIYIFGSITLSLPTFTWFIRTGMSFFTLVMS--

>SlitOR3

-------------------------------MDEAFLAFHRVLSFAGISIFAKQNWNSHRW-------
LAHQTFNFIIGVLCFIFTTGFVV--TNVSD---FLLCIQGACIWTTGVIM--
TITLGVCLIFRKEFRSFLEEMVFRDHMLEMPLIDHELRNLVTGSQEKLFKYTTLLLKSYVA
NVFLCATLYLCSPVYGMLVR-------EDKSLRLLAFDM-WFPWSL--
EDYTVYIISFIFHAYAGYLCCIAYPGLQSTIILLLGQLIRQTRILTFILLHMNEL----------
ALEVTGVQDERWQDICTLILSQCVDHYVKIKRFSNRLNLICRPFYLTLILVAIMLVCMCS
VKIAVSNKLSLDTFKYYIHEFCFILVVLMFCLIGQQVENECEALERAV-TEKWY--
IFNHIHKSNVRIFKWALGQRMPIYIFGTITLSLPTFTWFIKTGMSFFTLVMSVL

>BmorOR17

---------------------MREDKMEINNSQKFYTKM-IFRYLYSVGLGDWWYQHEDRSDS---
HRKLYCLWAVI-SNAYIFLNICNELLANFRKD---
LTDVEKNDAIQFSFAHPLIFAKIASFFFNRKKIREVFGRLLEENR---------
SVYSCGELEKESMKQIK-RYSLAFIGVSYMTLVMSTIDGLRAHFKE-----------
GIPIRTEVTYYP-SPSNSGVIVNILRFLVE-
FHWWYIVSVMVAIDSLAVASFVFVTFKFKLLQRYFKDMGLT---------
VRRDQSNMTDEALADKFRRDFIVGVKLHENALWCAENVQKAFGWVYSVQVFETVALL
VMCLVKLVTTNHNMIFLLANFAFMLCVIILNGSYMMPAGDVTSE---VPTSIFLCGWE--
LVQTDLRFLVVVAIQRSQVPVIMKAFGIMTLSYSNFIAVS--LFKFYNLF----

>HmelOR68

--------------------------LNLPNFSAYLKKSDVNMSILQV------------------
TSIFKRLLVPYKRLLTYYNKSLDEIGFTISKE-GFFYLQINATDYVILGLYS----
RAKILALSSHTFTKNFQWLGP-------------------
LLLCCSSKCLTYLFFFLLIHFILHLVCRVTKILLLNFVY--------------IKLFN-YFIIDY-
VYLLPFYTISINFNLISKIFCFFLAINMILRSESEPIHVHGASWVSSKKFSTENFD---------------
----
TKNPVSSTKTITMLKSIMKYANYILKIYIQKEFFSYIIGCKFYCIVFFLWNKDNEQIQKNFL
TTVLL-KILRKMVWLNFVVENLKQ----IYKIISISKYM----
KSKSKANLYLLYINYFLSYTFCTFRYSFKNKYLLEISIIQNHIYFTLINP--

>HmelOR69

-------------------------------IFFYFYTIHLWLNKKKISLKKQKNVLARKIT----
KFSIYRYFLINACTLRMINIFLII---GMQDN---FIQVGPYYNIYLLNPQL---------------
AFTLVTHMCS------------CATTGLRFAGSNLATFT--
IIYTFSKLSTTNYLLMALNKLYRIIMY----------------------------
NRLSTFKTYLLYLCKIICFLCSMRGLWENYIGIIIYSILPARGFVCLNLLLCYKQ--------------
--NRYINIITVTFIQIHSVVG-TYLRHTNKKI-

KIVYLCFTRSTLTSKGNQIANSTFPC-----
KCNTNGSNCLFFIFLNTRFPLPFLLCAGYSVKLNLRMKLK--KWNYFLYFCENN

>SlitOR47

---KLLNTSATY-------------------------------------

QVHQRFGGCFSVVNLINVLLSSVNICCVMFTIVFL--
DSWMEMSNKFYLGAALTQIFIVCWYADDIYRASVGVADAVYESGWY--
KSNTEVQARARRNFKRSQRPLYFTALKFRAITMITYSSILTTAYSYFTLLYTRD

>SlitOR46

----------------------------GKQWYHAEDIESIVPKISGFSYRKDKV-----------
SAWFWYIHMFLLFYVYAVGNFWYQW--KFAHG---AGDFIKSYVNISIIVII--
GNNSVWFVKQRPLLRTVLKKIEQSDELSR------RSSFLMRKHEKLMKIVK-
RIVLIFYGFNYIDAFF-IYFPHRVDLRN-----------NYSMTPCVGLQP----
LTASPNREICMTILTLQEFTINIVALNYQALLLFLIAHTAAMYQMMAYEMMALNDY-------
----
KKENLRQVKKKLSSLIERHCLTLDVVDNLRSLYSVPLGVNFGSNAVCISLFYLPLREC-----
--LQFMPVFVYCSLVFFLYCFLCQRLINSAEVFARAVYCCGWE--
NFGLKEKRLVFVMLRQSQKPVELLAADIXPVNIYTFATTLQAMFKFVTVVKF--

>Dp0R36_EHJ75140

MKSLVCSLAFHSITNNSGDRKRPLLFPFWIFEIDFGLTPTYEIAFILTVVKLLQLEHGLARP
RKYDSLTEDSFKGCVSADDLLEHYAQVYKKLLLFEQKISAPVVCLSAYAATEKWDAGE
FNAILILLCVGAITLIFIPCYLCTFLCVKINSICYACWNIPFWN--
AGPVIRPYLLLIMQRSLQPLPIKSPGFEEVSLQTFSNKMASAYSFFNMLRQANI

>Bmor_XP004924066

----------MK-----EDFKYIFTNG--KKYS---------------
LMEDYSQVYKKLLLFEQKCCGPVVCLTAYCATEKLAEGELNVVLILLCVGTIVMHYIPS
HLCTFLTIKVRSICDACWDTPFWN--
ADKAIRPYIVLIMQRSLRPLPLRAAGFEDICIQTFSRKMTSAYSYFNMLRQANR

>Bany_OR5_GZXD38A01A8I9X

MHSNFPVVCILELHITCLQTVKQKHREETCIPGNFLNSLRVSLKIRIGSAWWYFWPNPSHS
MRRLELSIVGFYYLIKIMIFFVVTSIAFACWNIPFWD--
ASTVIRPYLVLIMQKSLQPLPLKVPGLEEFSMLTFSNVSFL-------------
