# Supplementary material for: Mosaic Evolution of Molecular Pathways for Sex Pheromone Communication in a Butterfly
Source: Genes (Basel). 2022 Jul 31;13(8):1372. doi: 10.3390/genes13081372 (PMC9407440; doi:10.3390/genes13081372)
Supplement: Supplementary file 1 [file genes-13-01372-s001.zip › Supplementary File S2.pdf]

Supplementary File 2: Phylogenetic reconstruction of odorant binding proteins

We used the alignment of the OBP gene family published by [59] (their Supplementary File 5), which is based on the OBP gene sequences found in the fly *Drosophila melanogaster*, a number of moth species, and the butterfly species *Danaus plexippus* and *Heliconius melpomene*. We completed [59] OBP amino acid fasta file with:

i) our 47 Bany_OBP contigs, and with the OBP sequences we found in the butterflies
ii) *Papilio polytes* and
iii) *Melitaea cinxia.*

To do so, we mined the genome of *P. polytes* (website http://papilio.bio.titech.ac.jp/, accessed on May 23 2015) and *M. cinxia* (website: http://metazoa.ensembl.org/Melitaea_cinxia/Info/Index, accessed on May 23 2015) using the keywords "odorant binding protein" and found 9 annotated Ppol_OBP and Mcin_OBP genes.

We aligned the additional butterfly OBP sequences using Mafft V7 (website: http://mafft.cbrc.jp/alignment/server/) using G-INS-i reconstruction method (which gave the same OBP tree as in Vogt et al 2015 when only their OBP sequences were used), leave gappy regions, gap opening penalty of 3.0 and offset value of 1.0.

We failed to improve the alignments, on which the quality of the phylogenetic reconstructions depend, using the tools *Prank* (website: http://www.ebi.ac.uk/goldman-srv/cgi-bin/), *Gblocks* (website: http://molevol.cmima.csic.es/) or *Guidance* (website: http://guidance.tau.ac.il/). Hence, the alignments were manually curated to remove highly divergent regions and we excluded the OBP sequences (DpleOBP22, Bany-CN33, Bany-cn15 and MsexOBP26) because they produced huge gaps in the alignments. Given that large portions of the OBP amino acid matrix still showed gaps in alignments between OBP sequences from different species, we next used *Phyutility* to cutoff amino acid sites without enough information [94]. After removing sites where either 30% or 50% or more of the sequences provided no information, 165 and 147 amino acid characters, respectively, remained in our dataset. Having 147 or 165 amino acid positions to sort the relationships between 383 OBP sequences is not statistically sound. We, therefore, removed all OBP sequences from *Drosophila melanogaster* and several moth species present in [59]'s original OBP dataset. The final dataset used for our phylogenetic reconstructions contained 281 OBP sequences listed in the table below:

Species	Number of OBP ORF	Origin of OBP amino acid sequences
Manduca sexta	49	[59] Suppl file 5
Bombyx mori	44	[59] Suppl file 5

Other moths	27	[59] Suppl file 5
Bicyclus anynana	47	This ms
Heliconius melpomene	51	[59] Suppl file 5
Danaus plexippus	32	[59] Suppl file 5
Melitea cinxia	32	http://metazoa.ensembl.org/Melitaea_cinxia/Search/
Papilio polytes	9	http://papilio.bio.titech.ac.jp/)

We ran Phyutility again on this final aligned amino acid dataset of 281 OBP sequences, for a cutoff of 10%, leaving 229 amino acid sites in the dataset. The fasta file is available below.

Phylogenetic reconstructions were carried using maximum likelihood using the "LG+I+G" substitution model and 100 BT. Rate heterogeneity was set at four categories, and the gamma distribution parameter and the proportion of invariable sites were estimated from the data set. Tree reconstruction was performed using PhyML 3.0 [56], with both SPR (Subtree Pruning and Regrafting) and NNI (Nearest Neighbour Interchange) methods for tree topology improvement. Images were created using Evolview (website: http://www.evolgenius.info/evolview/).

94. Smith, S.A.; Dunn, C.W. Phyutility: A Phyloinformatics Tool for Trees, Alignments and Molecular Data. *Bioinformatics* **2008**, *24*, 715–716, doi:10.1093/bioinformatics/btm619.

Fasta OBP aligned amino acid sequences used for phylogenetic reconstructions:

>Msex-GOBP1_MsexOBP5_Msex207430_M73797_

---------------------------MGQN-----------------------------

--------------------------------------TR--SLV--LV---------VL

VGLVGA-V------------------------S---ADVQ--------VMKD-VTLGFGQ

ALEQCREES-----------------QLTE-E-------KM----E-EF-FHFWRED--F

--KFE------HR-ELGCALQCMSRH--FNLL----------------------------

-TD---S--S-R---------MHHENTDKFIKSFPNG----A-VLSKT--MVE-LIHNC-

-----ELQ------HDA----EE-D--------------H---CWRILRVA-ECFKISCT

K---A---

--GIA----PSMEVMM

AEFIMETEN---

-K--

>Msex-GOBP2_MsexOBP6_Msex205759_M73798_AF323972_

---------------------------MVNR-----------------------------
--------------------------------------LI--LVV-------------VV
VFITDS-V------------------------M---GTAE--------VMSH-VTAHFGK
ALEECREES-----------------GLPV-E-------VM----D-EF-KHFWRED--F
--EVV------HR-ELGCAIICMSNK--FELL----------------------------
-QD---D--T-R---------IHHVNMHDYIKSFPNG----Q-VLSEK--MVQ-LIHNC-
-----EKQ------YDD----IA-D--------------D---CDRVVKVA-ACFKKDAK
K---E---

--GIA----PEVAMIE
AVIEKY--

>Msex-PBP-A_PBP1_MsexOBP1_Msex205761_M21797_AF323972_

----------------MK--VA--------------------------------------

---------------------------------------V--V----AI---------VV

YLAVGN-V------------------------D---SSPD--------VMKN-LCLNFGK

ALDECKAEM-----------------NLSD-S-------IK----D-DF-ANFWVEG--Y

--EVS------NR-DTGCAILCLSKK--LDMI----------------------------

-DP---D--G-K---------LHHGNAMEFAKKHGAD----E-AMAKQ--LLD-IVHNC-

-----ENS------TPP----ND-D--------------A---CLKTLDIA-KCFKKEIH

K---L---

--NWA----PNMDLVV

GEVLAE--

------------------------------V-----------------------------

>Msex-PBP-B_PBP4_MsexOBP4_Msex205762_

---------------------------MKEA-----------------------------

-------------------------------------GVR--FKI--LL---------FL

IFPVVTGN------------------------F---KGKQ--------IMRS-VAETFGR

TVFECQNEV-----------------LMKFGS-------GI----LNDI-FRYWHEG--Q

--PLE------DR-DLGCIFRCILLK--LELV----------------------------

-ND---N--G-R---------LIDANADGFFQANGAD----E-SMTKH--LIE-LYHSC-

-----YQT------MRF----PQDD------------------CMLILEIG-KCCREGVR

N---A---

--HWT----PGSK---

>Msex-PBP-C_PBP3_MsexOBP3_Msex205760-RE_AF117580_

----------------MA--VIP---IF--------------------------------

---------------------------------------T--V----LL---------MM

T-AVKE-I------------------------A---PSSD--------AMRH-IANGFLK

VLDQCKHEL-----------------GLTD-Q-------IV----V-DL-YQFWKLQ--Y

--ALL------NR-DTGCAIICMSKK--LDLL----------------------------

-DG---T--G-R---------MHHGNTQEFAVSHGAT----D-EVASK--VVV-IIRDC-

-----EKQ------QEG----EQ-D--------------D---CVRVLEVA-KCFRTAIH

E---L---

--NWA----PNMEVVV

DELLTE--

------------------------------I-----------------------------

>Msex-PBP-D_PBP2_MsexOBP2_Msex205763_AF117588_

----------------MV-STKW---CF--------------------------------

---------------------------------------L--L----VT---------IA

VLTMEV-V------------------------S---ASQE--------VLKQ-MSVGFSK

VLQTCKTEL-----------------SVGD-H-------II----Q-DF-YNYWRED--Y

--DLL------NR-DFGCMVICMAVK--HDLI----------------------------

-ND---Q--L-T---------MHHGNAHAFAKTHGAD----D-DTAQQ--LVT-ILREC-

-----EAK------HQS----VE-D--------------V---CNRALEMA-KCFRTKIH

E---L---

--KWA----PAMEVVL

EEIMTS--

------------------------------V-----------------------------

>Msex-ABP1_Msex200462_AF117591_

---------------------------MSVI-----------------------------

-------------------------------------SFF--VLC--------------F

GVLAVSVG------------------------A---VSEN--------ERNQ-ISQSILP

HIVKCSQEY-----------------GVSE-G-------QI----KDAK-ESVN------

-----------PLGLNPCFLGCVLKS--AGII----------------------------

-DK---N--G-L---------FDVEATKEKSKKYISS-----EKDVTN--FDK-IIKDC-

-----TEV------NQK----NVSD--------------GNKGCDRAKELV-TCFLAKRG

D---F---

--SVF----TF-----

>Msex-ABP2_Msex200465_AF393491_note:samegenelocusasOBP12_

----------------M----------DRKD-----------------------------

-------------------------------------LCL--LII-------------AF

IL----AD------------------------G---VDSM--------SKQQ-LKNSGKM

FKKQCMGKN-----------------KVTE-D-------EI----GEID-KGRF------

---VE------QQ-NVMCYIACIYQM--SQVV----------------------------

-KN------N-K---------LNYEASLKQIDIMYPP------ELKDT--AKG-ALEAC-

-----KDI------AKK----NKDL------------------CEASFKTA-KCMYEYSP

K---D---

--FLFP----------

>Msex-ABP3_Msex209425_AF393488_

--------------------MQKQRGAMITA-----------------------------

-------------------------------------TLH--VVF------------A-L

LGFVYGAK----------------------------NKPV--------FSEE-IKEIIQT

VHDECVGKT-----------------GVSE-E-------DI----ANCE-NGIF------

---KE------DV-KLKCYMFCLLEV--AGLA----------------------------

-DE---D--G-T---------VDYDMLVSLIPE----------EYSER--ASK-MIFAC-

-----NHL------DTP----EKDK------------------CQRSFDVH-KCTYEKDP

E---F---

--YFLF----------

>Msex-ABP4_Msex200467_AF393490_

----------------M----------FRYI-----------------------------

-------------------------------------FCA--ICL-------------IL

IL----FD------------------------A---SYAM--------SRQQ-LKNSGKM

MKKSCIPKN-----------------DVTE-D-------EV----GQIE-QGKF------

---IE------DR-RVMCYIACIYTM--TQVV----------------------------

-KN------N-K---------LSYDAIVKQVDMMFPP------EMRTA--VKT-AAENC-

-----KDI------AKK----YKDI------------------CEASYWTA-KCMYDFDS

K---N---

--FVFP----------

>Msex-ABP5_Msex207487_AF393498_

-------------------MCKPMYYVVFSI-----------------------------
-------------------------------------IYL--SIVLAQK---------AD
NGNTKIAN------------------------V---QRND--------QGSM-DDVDVED
IMNQCNETF-----------------RIEM-A-------YL----Q-AL-NESGSFP---
---VE------TDRTPKCFLLCVLDN--TGVM----------------------------
-TK---D--G-D---------FDPERTAALFAGERAG------KVMDG--IQD-MAAAC-
-----ADR------KEK-------C--------------K---CEKSYNYL-KCLMTMEI
E---K---

--YAN----NN-----

>Msex-ABPX_Msex202108_AF117577_

---------------------------MISS-----------------------------
-------------------------------------LVH--VLT---------------
----LLAA----------------------------GVLA--------LDEE-QAELARM
VRENCVDEI-----------------GVDE-G-------LL----AKVD-DGADL-----
---MP------DP-KLKCYLKCTMEM--AGMI----------------------------
-SD------G-V---------VDVEAVLGLLPD----------DVKLR--TTD-IVRAC-
-----DTQ------KGA------DD------------------CDTAFLTQ-TCWQQANR
A---D---

--YIFI----------

>Msex-OBP8_Msex213843_

-------------------MCKPMYCVVFSI-----------------------------

-------------------------------------IYL--SIVAAQK---------AD

NGNTKIAN------------------------L---QSND--------QDSM-DNVDVED

IMNQCNETF-----------------RIEM-A-------YL----Q-AL-NESGSFP---

---DE------TDRTPKCFLLCVLDN--TGVM----------------------------

-MK---D--G-D---------FDPERTAVLFAGERAG------KVMDG--IQD-MAAAC-

-----ADR------KEK-------C--------------K---CEKSYNYL-KCLMTMEI

E---K---

--YAN----NN-----

>Msex-OBP9_Msex200460_

---------------------------MYVL-----------------------------

-------------------------------------KFF--ACI--------------V

LIGCIKAK--TLDD-LKNNYLE

MIVKCTSKY-----------------PITD-E-------DL----ELLK-TGDM------

---GD------KE-STKCLFACVYKK--TGMM----------------------------

-DD---K--G-M---------LDVDQTNKIVQTYFSD----NSEELKK--GLA-YTEAC-

-----KSV------NDA----PVTD--------------GDRGCDRAALLF-KCTTENLP

ALGGL---

--AVK----YCFFLEL

NDETIMPLA---

>Msex-OBP10_Msex200461_Mad10HM167934_

---------------------------MIRA-----------------------------
-------------------------------------VVF--CCC-------------MV
ALMPFSAN------------------------A---MTDE--------QKEK-IHEHFEK
LGLGCLKEN-----------------TITE-D-------DI----KDLR-AKKV------
---PS------GE-NAGCFLACMMKE--IGVL----------------------------
-ND---E--G-M---------LEKGRAMELAKEVFDD-----AEELKK--IEE-YMHSC-
-----SSV------NSE----SVGD--------------GEKGCERAMLAY-KCMVENAS
K---F---

--GFD----IVTNILH
SKLILSYLNINLHSS---

>Msex-OBP11_Msex200464_

---------------------------MWKI-----------------------------

-------------------------------------SYF--LFV------------FGV

AANLKHAY------------------------A---VTEE--------ERLM-LDSLMKP

KVLACVEEF-----------------GLKD-F-------SI----EDIR-KDHE------

--------------IDPCLLQCFLKK--AEVF----------------------------

-VD------G-M---------INLEKADETLREVIND-----EDEVEQ--IME-KGKEC-

----ADEA------NGS----DVSG--------------DDEDCARVAIFH-SCLREKNG

L---F---

--MAT----S------

>Msex-OBP12_Msex200465_note:samegenelocusasABP2_

--------------------------MPKHD-----------------------------
-------------------------------------VTE--DQVGGIEQGKFLENRNVM
CYIACVYS------------------------M---SQAM--------SKQQ-LKNSGKM
FKKQCMGKN-----------------KVTE-D-------EI----GEID-KGRF------
---VE------QQ-NVMCYIACIYQM--SQVV----------------------------
-KN------N-K---------LNYEASLKQIDIMYPP------ELKDT--AKG-ALEAC-
-----KDI------AKK----NKDL------------------CEASFKTA-KCMYEYSP
K---D---

--FLFP----------

>Msex-OBP13_Msex200468_

---------------------------------------M--LFL-------------VV

AKFFILLS------------------------L---GEAM--------TMKQ-IRNTGKM

MRKQCQPKN-----------------NVED-E-------KI----DPLG-KGVF------

---IN------EK-EVKCYMACIMKM--ANTI----------------------------

-KN------G-K---------INYEAAMKQADLLLPE------DIKEP--AKE-ALTSC-

-----RKV------ADS----HKDV------------------CDAAFYIS-KCIYEYNP

D---I---

--FYF-----------

>Msex-OBP14_Msex200463_

--------------------------------G---ITEK--------QRLH-IREELIE
IGTECIIKN-----------------PITI-G-------DV----RSFR-NKQF------
---PN------GP-NAGCFIACVFNK--AGIMLGLLEINNFPTVQTQDALLRAYLIKQEL
FDD---E--G-L---------VSQKTATEKATKVFDD-----ETELKN--YEQ-FIAVC-
-----DKV------NEE----SVSD--------------GQKGCERAKLAF-QCLIQNSK
Q---V---

--FIP----YNLLLGS
R---

>Msex-OBP15_Msex201454_

---------------------------MRRK-----------------------------
-------------------------------------LLN--------------------
--------------------------------I---KENP--------FTSV-VRKALIN
TARSCMVHV-----------------NATQ-E-------DL----E-YL-RKDP------
---PF------PE-KASCIVKCLLEK--IGVV----------------------------
-KN------N-K---------YSKSGFMMVVTPLVFA----NKKKLDH--MKT-VSENC-
-----ESE------IVH----KESS--------------P---CDAGNEVT-TCIFKYAP
E---L---

--HLR----G------

>Msex-OBP23_Msex203518_

---MYA-HDKLSDM

VAEQCLNEM-----------------YPKS---------------KRIE----I------

-----------QESDEPCIIFCVLKK--FGIM----------------------------

-ST---N--G-V---------INLEVFRKRVQNSHQH------EQRNT--MND-IGSNC-

-----LES------AEA-TQHKQDV------------------CKKAKVFN-DCTHLYRI

L---L---

--K-------------

>Msex-OBP24_Msex203519_

----------------------MLVLKFLLC-----------------------------

-------------------------------------ALT--SAL--AM---------KQ

HAPSGTMV------------------------D---FTDA--------KVQG-HLDALVR

MAQSCVIKV-----------------RATP-K-------DV----RAYF-TNSS------

---PV------SR-SGQCFAACMLEQ--SDVV----------------------------

-NH------G-K---------INRELLVHLASLVNGK----NSRVVRK--LNS-VSRLC-

-----LDS------IDG----MSDR------------------CQLASTYN-DCLNENMI

E---F---

--AFP----LDIAEEA

VRKMPFHLIQPKNLPQEMRPANY-------------------------------------

>Msex-OBP27_Msex208382_

-----------MKRAKRRQPRQETNMFKIDF------------------------------
-----------------------------------QSL--MLF--LIFGAAFILVIAF
QPLSKEEH------------------------I---ERYN--------KMSE-DVEPFRK
NLTECARQV-----------------KASM-V-------DV----EHFL-KRIP------
---QS------TM-QGKCFVACILKR--NNII----------------------------
-TK------N-K---------VNVKNLLEANRAVYGD----DSEVLAR--LKT-AVKEC-
-----SEV------VEG----VFEI------------------CEYASVFN-DCMHMKME
H---L---

--LDR----MTMERRM
EALGQMTSNPDEWSDDEDEMLKLVKDEL--------------------------------

>Msex-OBP29_Msex211541_

-------MMGVDAIHDPQTKIDKDTIITRNL-----------------------------
-------------------------------------KLE--KKG--QAKTSVQNRKKDI
EREPDWSY------------------------Q---PIPP--------EVST-HVEQFKK
NMTECLKEV-----------------QAND-K-------RP----VKRL-SPKT------
---ES------PI-HGECLIACVLKR--NGVI----------------------------
-DN------G-K---------INKNNLLTLVSKFYAK----DTKLMKK--LDK-NLEHC-
-----IEI------STR----NRDE------------------CVLASQLN-ACTNDLMA
S---N---

--KHK----IIVNY--

>Msex-OBP_Y_Msex200466_

-----MPKH-----------------DVTE-D-------QV----GGIE-QGKF------
---LE------NR-NVMCYIACVYSM--SQAV----------------------------
-KN------N-K---------IMYDNMIKQVDMMFPP------DIKDA--VKD-SIENC-
-----RPV------AKK----YKDV------------------CEAAFWTA-KCMYDYNP
A---N---

--FVFP----------

>Msex-ABP6_Msex203889_AF393499_

---------------------------MKVTNGL--------------------------

-------------------------------------FLV--FLV--SV----------L

ACGIAASP------------------------K---KIYR--------IPPQ-SSEKIVE

EVLKCVQKM-----------------GLES-T-------VV----NLLK-EGKY------

---TE------DD-RVIETLMCSNQN--LGNV----------------------------

-NG---D--G-K---------VNIDKVMNEIF-----------SNKPE--IRS-ALVAC-

-----EKD------GGK------SS------------------LETFKNX----------

>Msex-ABP7_Msex203892_AF393500_

---------------------------MQAC-----------------------------
-------------------------------------LFL--TLV---------------
-LAVVGLN------------------------A---HNVH--------LTDG-QKEKANE
HIAACIKET-----------------GIKP-E-------VI----AEAK-KGHY------
---SE------DE-AMKKFILCFFHK--AGIV----------------------------
-NA---D--G-K---------LNLDVAIAKLPPG---------VDKTE--ATK-NLEGC-
-----KDN------GGK------DA------------------AETAFAIF-KCYKDATK
T---H---

--VLF-----------

>Msex-ABP8_Msex201708_AF393501_

---------------------------MKAL-----------------------------

-------------------------------------LVL--AAC---LV----------

------------------------------------LAQA--------LTDE-QKEKLKK

HKSECLSET-----------------KVEE-Q-------LV----NKLK-AGDYK-----

---AE------ND-NLKKYALCMMMK--SELM----------------------------

-TK---E--G-K---------FKKDVALSKVPNP---------ADKPM--VEK-LIDTC-

-----LAN------KGN------TP------------------HQTAWNYV-KCYHEKDP

K---H---

--AIF----L------

>Msex-OBP7_Msex203888_

---------------------------MYFK-----------------------------

-------------------------------------ILV--LCG--IL----------A

IKNGICDE------------------------R---RAVS--------FVPE-VADHITK

TIKYCMVKH-----------------DNDP-K-------II----ELVR-QGSY------

---GV------DE-PFKKFIHCAYYK--SGYA----------------------------

-NE---D--G-H---------VLVNKVIKAFPK----------DAN----IEE-VTKKC-

-----STI------KGE------DA------------------EDTTYQFF-KCFELNAP

I---R---

--LAL----E------

>Msex-OBP16_Msex201707_

---------------------------METF-----------------------------

-------------------------------------QFA--VFC--------------V

AFYTFVVS------------------------S---PIKE--------FYPKRDVATLKK

YQMECVEDT-----------------KVDP-D-------LV----IRFK-AGDWR-----

---SE------QP-SLKNWVLCILNK--MGLM----------------------------

-TM---D--G-V---------YRLDEAMARVGT----------KDKDM--AEK-LIDQC-

-----LST------TAL------PA-------------------PDIAWKYV-HCLHVNDP

L---G---

--NYS----SISILTP

>Msex-OBP17_Msex203890_

---------------------------MQSC-----------------------------

-------------------------------------LFL--TLL---------------

-VAVVGIN------------------------A---DTVL--------LTDI-HKEKSNA

NIAACIKES-----------------GVKP-E-------IL----AEAK-KGNY------

---SE------DE-AMKKFLLCFFNK--SGIM----------------------------

-NA---D--G-K---------LNLDVALANLPPG---------VDKNE--ATK-ALEEC-

-----QHK------NGK------DA------------------PDTAFTIF-KCYRAATK

T---Q---

--VLF-----------

>Msex-OBP18_Msex202300_

---------------------------MYQY-----------------------------

-NGYRSSS------------------------Q---SPRR--------YKRE-RRVDNTG

QRSQYNPNT-----------------QRNSGY-------ED----TYRN-EEKNSSE---

--NNT------NTDNKACALQCFLEN--LQMT----------------------------

-AQ---D--G-M---------PDKYLVTHAITKNVKN-----DDLQDF--LQE-SIDEC-

-----FQI------LEN---EDSDD--------------K---CEFSKNLL-LCLSEKGK

A---

--NCD----DWKDDMH

F---

>Msex-OBP21_Msex202698_

--------------------------MIKQ------------------------------

-------------------------------------MCA--FLF----------ALSVI

CSVNLAAS------------------------K---SIYV--------FPPE-KAEMILE

NAIKCITES-----------------GLQT-F-------VG----QEIK-QGKY------

---TD------DE-RTLGALVCANEK--IGYS-----------------------------

-NE---S--G-R---------LNIDKIMIDLF-----------PLKPE--IRS-DLEAC-

-----NKD------YGL------DP------------------VGTFKSFL-ICFRKRVP

F---R---

--VVL-----------

>Msex-OBP22_Msex203248_

---------------------------MSWL-----------------------------

-------------------------------------HAV--VAL---AL-------AAS

AAPATTSC------------------------K---NCIA--------LGKE-EKAMFRA

HSDACLPQS-----------------GVEP-K-------VV----ESML-NGQL------

---VE------SA-ALRRHVYCVLMK--CKLV----------------------------

-SK---E--G-K---------LMKNAMLGKMAMR---------SDGKN--ATK-VLEGC-

-----ADQ------TGD------TP------------------EDLAWNLF-RCGYDKKT

M---L---

--FDYMPTSGASSGDI

DNISK---

>Msex-OBP30_Msex213730_

--MYINMDFRVKLVTRCLTLKRHSYCKMKIT-----------------------------

-------------------------------------ASL--VFV--LL----------N

VVSIYGES------------------------K---EIYF--------FPKE-AAVTFLE

ATLKCIADS-----------------NYDS-S-------VI----DQIM-QGKY------

---IE------DD-KTVNALICACVA--TGFG----------------------------

-YP---D--G-K---------VNVEKIMKESV-----------PTRQD--LRP-FIEDC-

-----NRE------SGK------TP------------------AQTFRGIV-KCYREKLP

V---Q---

--LRFSN---------

>Msex-OBP31_Msex214143_

---------------------------MQEV-----------------------------
-------------------------------------EYA--DERRNVT---------DI
LKETDVPN------------------------S---TSFN--------DEGF-TTRNLED
AALDSTKIQ-----------------IIRK-Y-------ND----TDTI-SRRKKRS--E
PLFDK------PD-TNQCLSQCVFAN--LQVV----------------------------
-DS---R--G-I---------PRETELWNLVQSAVTS-----QQSRAA--LRD-QIRAC-
-----FQE------LQS---EAEDN--------------G---CSYSNKLE-RCLMLRFA
D---R---

--KMD----EGNANEK

KADQ--

>Msex-OBP32_Msex203893_

----------MLCMHHCKLTIFVTTINMQSY-----------------------------

-------------------------------------LFL--TLV---------------

-LAVVGIN------------------------A---DIVL--------LTDV-QKEQSNA

NIAACIKES-----------------GVKP-E-------IL----AEAK-KGNY------

---SE------DE-AMKEFLLCFFNK--TGIM----------------------------

-NA---D--G-K---------LNLDVALANLPPG---------VDKNE--ATK-ALEEC-

-----QHK------NGK------DA------------------ADTAFTIF-KCYSAATK

T---Q---

--VLF-----------

>Msex-OBP33_Msex203891_

---------------------------MKAA-----------------------------
-------------------------------------QNL--FGI--IL-------FSVF
AAVIAAST-----------------------K---KIFH--------LSPE-SGEKLVE
GIIKCIAKL-----------------NFDP-S-------LI---NLIK-EGKY------
---LE------DE-RLIKAIICMNVD--SGVG----------------------------
-TA---D--G-R---------LNVDAVMERIF-----------SNNAE--IRK-GLICC-
-----EKE------YDG------TP-----------------VGNLRGTL-TCLKETLP
F---K---

--IRM-----------

>Msex-OBP35_Msex214323_

---------------------------MRTA-----------------------------
-------------------------------------IFL--LLV-------------SV
ISCYAAEE------------------------K---DTYY--------YPKA-PSEIFLN
AAKKCVEDL-----------------SYNS-S-------IM----DQIM-QGKY------
---IE------ED-KTLNVLICAAVN--TGYG----------------------------
-NA---D--G-K---------LNVEKVVKELY-----------PERQD--VWP-IIEKC-
-----NLE------QVT-----STP------------------LETFKGIV-VCLKNNLP
F---K---

--IRFPM---------

>Msex-OBP36_Msex214586_

---------------------------MKVT-----------------------------
-------------------------------------ILV--ILI-------------IG
VSCDAAFT------------------------K---PIYR--------FPPK-QSEMYVE
AVVKCVAKL-----------------GYDL-A-------IL----DQIR-QGKY------
---TD------DD-KSVEALVCANND--IGYG----------------------------
-LP---N--G-Q---------LDADKTIQDLF-----------PTKPE--IKS-VFDKC-
-----DKD------YGV------DP------------------AGNFKAFL-LCFKDEIP
F---K---

--VII-----------

>Msex-OBP40_Msex214420_

---------------------------MRAA-----------------------------
-------------------------------------IFL--LLV-------------SV
ISCYAAEE------------------------K---DTYY--------YPKA-PSEIFLN
AAKKCVEDL-----------------GYTS-S-------IM----DQIM-QGKY------
---IE------ED-KTLNVLICAAVN--TGYG-----------------------------
-NA---D--G-K---------LNVEKVVKELY-----------PEKQD--VWP-IIEKC-
-----NLD------QKT-----STP------------------SETFKGIV-VCLKTNLP
F---I---

--IRFPM---------

>Msex-OBP19_Msex202657_

------------------MKLRTKWNGIYTS-----------------------------
-------------------------------------SLK--IVF--IKYVPAKLPSLHL
MVISSQGP------------------------P---PFLKDIP-----EKCR-GPPPVVE
KPHECCKVE-----------------PFFEAS-------DF----TECG-YKNSNED---
-VGFK------RGPPDCSKQLCLMKK--YNLA----------------------------
-KD------D-Q---------VDFEALKKFLDDYAEK----YPAFKSA--VEK--AKEC-
-----VKE------DLP----GPP-------------------SVCLANRIVFCIGSVVM
----F---

--ECPAE--NWSETEG
CKTLKDHMTECKPFFQRQ--

>Msex-OBP20_Msex202656_

---------------------------MATD-----------------------------
-------------------------------------KEGINADL---------------
--LRQD-RSKATLK
PISACCDIP-----------------ELGDAK-------PL----SECS-NP--------
-----------KLPGPCKDVQCAFEK--SGFL----------------------------
-TD---N--L-T---------LKKEVYKKHLRQWAKN----HDGWSDA--VEK-AISDC-
-----VDK------DLR----QYLD------------------IPCKAYDVFTCTGIAML
----K---

--KCP------DAAWK
C---

>Msex-OBP25_Msex207317_

---------MQTLRETRPRCTAERFLIMFKI-----------------------------
-------------------------------------VLI--CAF--GI---------AA
SNADAPGD--PPKV-YCGEIPY
KIFECLGSP-----------------RIIK-P-------ES----SMKC-D---------
-----------KSISECDKMRCIFRE--EGWM----------------------------
-VN------N-V---------VDKSKVGASFDQFGKD----NPDWAAA--IGA-AKTEC-
-----LAQ------ELP----AQGV--------------F---IGCPAYDILHCTLTTLI
----R---

--NANPA--KWSSSEE
CKYPRQYAGACPICPSNCFVPAIPTGSCNACLSLPRTP----------------------

>Msex-OBP28_Msex210958_

---------------------------MYRL-----------------------------

-------------------------------------ALL--LVL--SC---------VL

LVQAD---------------------------D---EWMD--------FMQK-CGQLIHP

AGIRCCKKK-----------------YVST---------DL----TEDL-KECMQLP---

-----------GKPPSCEIEICVAKK--RGFA----------------------------

-SD---D--G-T---------INKAAFKALLSKMDDK---------DL--QAS-IVEDC-

-----VDG------DIS----EYGP--------------D---DLCDLKRLHTCVALHLV

----V---

--DCP----EWSNEGA

CSGSADVAADCIKSMQ--

>Msex-OBP34_Msex214192_

EQRCRNPP------------------------T---APQK--------IERV-ITLCQDE

IKLSILREALDVIKEEHTMFLITEALDVIKEE-------HT----MPAQ-RKRNKRE--V

PFTHD------EKRIAGCLLQCVYRK--VKAV----------------------------

-DG---F--G-F---------PTLEGLVGLYSDGVNE-----RGYFMA--VLE-ASREC-

LMKNHDKF------SRT----TPMD--------------NGRNCDISFDIF-ECISDRIG

E---Y---

--CGN--------AGL

>Bmor-GOBP1_OBP1_NM_001044031_

--------------------------------------MW--KLV--VV---------LT

VNLLQG-A------------------------L---TDVY--------VMKD-VTLGFGQ

ALEQCREES-----------------QLTE-E-------KM----E-EF-FHFWNDD--F

--KFE------HR-ELGCAIQCMSRH--FNLL----------------------------

-TD---S--S-R---------MHHENTDKFIKSFPNG----E-ILSQK--MID-MIHTC-

-----EKK------FDS----EP-D--------------H---CWRILRVA-ECFKDACN

K---S---

--GLA----PSMELIL

AEFIMESEAD--

-K--

>Bmor-GOBP2_OBP2_NM_001044033_

---------------------------MFSF-----------------------------
--------------------------------------LI--LVF--------------V
ASVADS-V------------------------I---GTAE--------VMSH-VTAHFGK
TLEECREES-----------------GLSV-D-------IL----D-EF-KHFWSDD--F
--DVV------HR-ELGCAIICMSNK--FSLM----------------------------
-DD---D--V-R---------MHHVNMDEYIKGFPNG----Q-VLAEK--MVK-LIHNC-
-----EKQ------FDT----ET-D--------------D---CTRVVKVA-ACFKKDSR
K---E---

--GIA----PEVAMIE
AVIEKY--

>Bmor-PBP-A1_PBP1_OBP3_NM_001044029_

----------------MS--IQG---QI--------------------------------
---------------------------------------A--L----AL---------MV
YMAVGS-V------------------------D---ASQE--------VMKN-LSLNFGK
ALDECKKEM-----------------TLTD-A-------IN----E-DF-YNFWKEG--Y
--EIK------NR-ETGCAIMCLSTK--LNML----------------------------
-DP---E--G-N---------LHHGNAMEFAKKHGAD----E-TMAQQ--LID-IVHGC-
-----EKS------TPA----ND-D--------------K---CIWTLGVA-TCFKAEIH
K---L---

--NWA----PSMDVAV
GEILAE--

------------------------------V-----------------------------

>Bmor-PBP-A2_PBP2_OBP4_AM403100_

----------------MK--

---------------------------------------L--Q----VV---------LV

VLTVEM-V------------------------C---GSRD--------VMTN-LSIQFAK

PLEACKKEM-----------------GLTE-T-------VL----K-DF-YNFWIED--Y

--EFT------DR-NTGCAILCMSKK--LELM----------------------------

-DG---D--Y-N---------LHHGKAHEFARKHGAD----E-TMAKQ--LVD-LIHGC-

-----SQS------VAT----MP-D--------------E---CERTLKVA-KCFIAEIH

K---L---

--KWA----PDVELLM

AEVLNEVSWKS---

>Bmor-PBP-B_OBP5_Gongetal_2009_

---------------------------MKQR-----------------------------
-------------------------------------LRV--LLL-------------RF
CILQTVLS------------------------E---SGVD--------VVKN-LSLSFAR
FFLECDEER-----------------HFQP---------EV----RLKV-MTFWYSE---
--SST------WDRDVGCAFLCIFKK--MEID----------------------------
-NP------Q-D---------PSYRTHLELLSFANSE----DNKIANQ--MVE-IFYAC-
-----GEN------TET------DP------------------CLWALEQV-KCYKNRIN
Q---L---

--GLT----PTF----

>Bmor-PBP-C_PBP3_OBP6_NM_001083626_

----------------MA--RYN-------------------------------------

---------------------------------------I--V----VA---------VL

VLGVVG-A------------------------R---GSSE--------AMRH-IATGFIR

VLDECKQEL-----------------GLTD-H-------IL----T-DM-YHFWKLD--Y

--SMM------TR-ETGCAIICMSKK--LDLI-----------------------------

-DG---D--G-K---------LHHGNAQAYALKHGAA----T-EVAAK--LVE-VIHGC-

-----EKL------HES----ID-D--------------Q---CSRVLEVA-KCFRTGVH

E---L---

--HWA----PKLDVIV

GEVMTE--

------------------------------I-----------------------------

>Bmor-OBP7

--------------------------------------AV--------TEEE-LKIEFTK
LVMKCTKDH-----------------PVDM-S-------EL----MQLQ-QLIA------
---PK------KT-ESKCLLACAYKL--NGVM----------------------------
-TS---Q--G-L---------YNLEHAYKIAEMSKNG----DEKRLEN--GKK-VADIC-
-----VKV------NDV----EVSD--------------GEKGCERAALIF-KCTLENAP
KVFKF---

--GSS----EYNCQ--

>Bmor-OBP8_BAI44701_

---------------------------MLRV-----------------------------
-------------------------------------VVI--CVC---------------

-FLVIAPY------------------------G---INAS--------SLDD-LKMVYKN

VIKECVGDY-----------------PITA-A-------DL----KLIK-ARQI------

---PN-------D-DIKCVFACAYKK--TGMM----------------------------

-TE---E--G-M---------LSVEGIKDMSQKYLSD----NPEQLRK--SKE-FAEAC-

-----SSV------NDQ----QVSD--------------GTKGCERAALIF-KCSTEKIT

N---F---

--GFE----L------

>Bmor-OBP9

---------------------------MLRV-----------------------------

-------------------------------------VVI--CVC-------------FL

VVAPYGIN------------------------A---VSYE--------QKIK-IRDQLDR

AGFECFKDH-----------------KITE-D-------DI----KNLR-ANKP------

---AT------GE-NVPCFIACVMKK--TGVM----------------------------

-ND---Q--G-V---------IRKGPVLELAKKVLAD-----DKDIKK--LQD-YIHSC-

-----SHV------NSE----TVHD--------------KGKGCEFAMQAY-TCMSANAS

K---F---

--GFN----I------

>Bmor-OBP10

---------------------------MLRV-----------------------------

-------------------------------------VVI--CVC-------------FL

VIAPYGIN------------------------A---VSDE--------QKIK-IREQIDK

SGFECFKDH-----------------KITE-D-------DI----KNLR-ARKP------

---AT------GE-NVPCFIACVMKK--TGVM----------------------------

-ND---Q--G-V---------IHTEPVLQLAKKVLTD-----DKDIKK--LQD-YIHSC-

-----SHV------NSK----TVHD--------------KGQGCEFAIQTY-TCMSANAS

K---F---

--GFD----V------

>Bmor-OBP11_BAH36759_

---------------------------MSAN-----------------------------

-------------------------------------SFV--VLA--------------F

CALAVGVN------------------------A---LTEE--------QKAE-ITKSSLP

LIAECSKEF-----------------SVNQ-G-------DI----DAAK-KLGD------

-----------PSGLNSCFVGCFMKK--AGII----------------------------

-NA---S--G-L---------FDVAATIEKSKKYLTS-----EEDLKA--FEK-LTETC-

-----APE------NDK----PVSD--------------SDKGCERAKLLL-DCFVANKG

S---F---

--SVF----SL-----

>Bmor-OBP12_NP_001153664_

---------------------------MTSF-----------------------------

-------------------------------------MVF--FVL--------------S

VLTLKYSD------------------------A---LTDE--------QKNK-IQSKFIE

IGAECIVEH-----------------PISI-D-------DI----NSFK-NKKF------

---PS------GV-NAGCFVACIFNK--IGLF----------------------------

-DD---K--G-N---------LSHNSALEKAKGIFNA-----DEEVKN--LEE-FLNRC-

-----AKV------NGE----AVGD--------------GVKGCERAKLAY-NCLIENSL
E---F---

--GFN----IDF----

>Bmor-OBP13

---------------------------MLKI-----------------------------
-------------------------------------HVL--LCF--GM----------A
ILYFGSAK------------------------A---VTPE--------ESKA-FEAFAKP
VIEQCQKDF-----------------GMDK-E-------SF----AQKN-L---------
-----------DE-IDECLIACVVEK--FGIT----------------------------
-ND---E----K---------IDGDALKALVTKFVGN-----EEERNK--INK-IVEEC-
-----TED------ANK----SGDG--------------T---CNTSTILF-LCLLKNGK

D---L---

--WGF-----------

>Bmor-OBP14_NP_001140185_

----------------M----------ERKD-----------------------------
-------------------------------------FYL--LIV-------------VV
AL----TS------------------------G---VSSM--------SRQQ-LKNSGKM
LKKQCMGKN-----------------DVTE-E-------EI----GDIE-KGKF------
---IE------QK-NVMCYIACIYQM--TQII----------------------------
-KN------N-K---------ISYEASIKQIDLMYPP------ELKES--AKA-SAGRC-
-----KDV------SKK----YKDI------------------CEASYWTA-KCMYEDNP
K---D---

--FIFA----------

>Bmor-OBP15

----------------M----------FLKN-----------------------------
-------------------------------------IFI--ECV-------------LL
YFVMLNTS------------------------F---VNTM--------TKQQ-IKNSGKI
LKKACISKN-----------------DVTE-D-------QI----SDID-KGKF------
---IE------DK-NVMCYIACVYSM--SQVV----------------------------
-KN------N-K---------FVHDAMVKQVDMMFPT------EMRDA--VKA-SIANC-
-----RGV------AKN----YKDI------------------CEASFWTA-KCMYEFDP
A---N---

--FVFA----------

>Bmor-OBP16

------------------------------M-----------------------------
-------------------------------------RIS--FLF-------------LI
SVTIITFD------------------------S---VFAM--------TRAQ-VKKTMTI
MKNQCMPKN-----------------GVTE-D-------QV----GKIE-EGIF------
---LE------NH-NVMCYIACVYKT--IQVV----------------------------
-KN------D-R---------LDKDLISKQIDVLYPQ------EIRES--TKK-AVGDC-
-----INL------QEK----YDDW------------------CEGIFRST-KCLYEKDP
A---N---

--FIFP----------

>Bmor-OBP17

---------------------------------------M--------TRQQ-LKNSGKI
MKKTCMPKN-----------------DVTE-E-------EI----GQIE-QGKF------
---LE------QR-NVMCYIACIYTV--TQVV----------------------------
-KN------N-K---------LSYDAVIKQVDVMFPA------EMRPA--VKA-AAENC-
-----KDI------SKT----FKDI------------------CEASYWTA-KCMYDFDP
K---N---

--FVFP----------

>Bmor-OBP18

---------------------------------------M--ILI-------------VI

AKFLILIS------------------------L---CETM--------TMKQ-IKNTGKM

MRKSCQPKN-----------------NVDD-E-------KI----NPIN-DGVF------

---IE------EN-EVKCYIACIMKM--ANTM----------------------------

-KN------G-K---------LNFEAAMKQADLLLPD------EMKEP--TKE-AIVAC-

-----RKV------ADS----YKDV-----------------CDASFHVT-KCIYNHNP

S---V---

--FFFP----------

>Bmor-OBP19_NP_001140188_

--------------------------------M---TSAK--------TDVE-IKAWFLG

QAVECSKDH-----------------PVTT-E-------EL----RMHK--HEL------

---PD------SK-NAKCLMKCVFRK--CNWL----------------------------

-DS---K--G-M---------YDINAAYASSTKDFSD----DKTKQEN--ANK-LFDTC-

-----KSV------NEE----NVGD--------------GEEGCDRSLLLA-KCLTKAAP

Q---V---

--SIY----YS-----

>Bmor-OBP20_ABPX_NP_001037500_

---------------------------MAVH-----------------------------
-------------------------------------IFL--ILA---------------
--SYMALA----------------------------AHGQ--------LDDE-IAELAAM
VRENCADES-----------------SVDL-N-------LV----EKVN-AGTDLAT---
---IT------DG-KLKCYIKCTMET--AGMM----------------------------
-SD------G-V---------VDVEAVLSLLPD----------SLKTK--NEA-SLKKC-
-----DTQ------KGS------DD------------------CDTAYLTQ-ICWQAANK
A---D---

--YFLI----------

>Bmor-OBP21

---------------------------MITA-----------------------------

-------------------------------------SLH--VIF------------A-L

LAFVYGGK----------------------------DKPV--------LSEE-IKEIIQT

VHDECVGKT-----------------GVSE-E-------DI----TNCE-SGIF------

---KE------DV-KLKCYMFCLLEE--AGLV----------------------------

-ND---D--G-T---------VDYEMFTSLIPE----------EYFDR--ATK-MIFSC-

-----KEL------DTP----DKDK------------------CERAFEVH-KCSYEKDP

D---F---

--YFLF----------

>Bmor-OBP32

---------------MYSHKYLNDFTNIPEI-----------------------------
-------------------------------------LII--LLS--SVALMSYGYNTKL
FSHSLGSE----------------------------PSLS--------ILYA-RDKKSDK
VTNECLMEM-----------------YPKN---------------LYKY-PLRI------
-----------DRNDIPCIIHCVLKK--FGII----------------------------
-SN---D--G-F---------INIKNYYRRVQAIHRY------DPRIL--ISD-VGETC-
-----AQN------ING-MNLDHDV------------------CKKAKVFN-DCTQLYAI
SYREP---

--EDW-----------

>Bmor-OBP33

---MYA-HDKLSDM

IADQCLNEM-----------------YPRS---------------KRLE----I------

-----------EESDEPCIIFCVLKK--FGIM----------------------------

-SP---T--G-V---------INLEAYRKRVQLPEQL------AQRNS--IND-FGSAC-

-----LES------AEA-TQHKQDV------------------CKKAKVFN-ECTHLYKI

L---L---

--K-------------

>Bmor-OBP34

-------------------MEKMILLNVFAV-----------------------------
-------------------------------------VLP--CVL--AS---------RT
RGSSGTLV------------------------D---FTDP--------KVQG-HLDALVR
MAQSCVIKV-----------------RATP-K-------DV----RAYF-TNSS------
---PV------SR-SGQCFATCMLEQ--SDII----------------------------
-NH------G-K---------VNRDLLVHLAGLVNGK----NSRVVRK--LNS-VSRLC-
-----LDS------ISG----MTDR------------------CQLASTYN-DCLNENMI
E---F---

--AFP----LDIAEEA
VRKMPFHLIQPK--

>Bmor-OBP35

--GMST-HVLDFKR

NMTECLKEV-----------------QNND-K-------RP----IKRL-SPKQ------

---ES------PI-HGECLIACVLKK--NGVI----------------------------

-QN------G-K---------VNKDNLMALVSKFHAK----ETKLMKK--LEK-NLDRC-

-----INI------SVK----NHDE-----------------CSLASQLN-DCTNDIMA

S---S---

--KQK----ILFNY--

>Bmor-OBP36_NP_001140186_

-------------------------MAVSEI-----------------------------

-------------------------------------SRI--LTF--LTIVSFIYIVYSF

KPLTKDEH------------------------I---ERYN--------KMNE-DIEPFRK

NLTECARQV-----------------KASM-A-------DV----EKFL-KRIP------

---QS------NM-EGKCFVACILKR--NSLI----------------------------

-KN------N-K---------LSQENLLEVNRAVYGD----DSEVMSR--LKT-AILEC-

-----SKI------VED----IFEI------------------CEYASVFN-DCMHMKME

H---I---

--LDK----ITMERRM

EALGQMSSNPDEWSEEEDEMLKLVKDEL--------------------------------

>Bmor-OBP37_NP_001140187_

---------------------------MFYP-----------------------------
-------------------------------------FRF--TLLFYGL---------FV
IYLVRAEP------------------------E---KENH--------FTLA-LKKTLFS
TARSCMSHV-----------------NANE-T-------DL----E-YL-RKDP------
---PF------PD-KAACIIKCLLEK--IGVV----------------------------
-KN------N-K---------YSKMGFLTAVSPLVFT----NKKKLDH--YKS-VSENC-
-----EKE------INH----DQTT--------------E---CELGNEVV-SCIFKYAP
E---L---

--HFK----T------

>Bmor-OBP38_CAS90131_NP_001140191_

-------------------MANLVLLLTFVL-----------------------------
-------------------------------------MTL--SMARLKS---------TE
APKSKTAL------------------------F---NDQD--------NMGY-EELDMEE
IMSACNESF-----------------RIEY-A-------YL----E-SL-NDSGSFP---
---DE------TDKTPKCYIRCVLEK--TEIL----------------------------
-SE---N--G-V---------LNPATAALVFAGERNG------KPMSD--LEE-MAVAC-
-----ADR------HEK-------C--------------K---CEKAYNFV-KCLMYMEI
D---K---

--YEK----KN-----

>Bmor-OBP39

----------------------MVRKISALL-----------------------------

-------------------------------------CCF--CVL--GISMCDSAISTDN

EQRCKNPP------------------------T---APQK--------IERV-ITLCQDE

IKLSILREA----------------LDVIKEE-------HT----MPAE-RKRNKRE--V

PFTHD------EKRIAGCLLQCVYRK--VKAV----------------------------

-DG---F--G-F---------PTLEGLVGLYSDGVNE-----RGYFMA--VLE-ASREC-

LMKNHDKF------SRT----TPMD--------------NGRNCDVSFDIF-ECISDRIG

E---Y---

--CGT--------SGL

>Bmor-OBP22

---------------------------MLKV-----------------------------
-------------------------------------FVV--VVC--TL-------GASQ
LCAALYTQ------------------------K---VAVS--------FPKD-KTTIVVE
AMKSCIAKT-----------------GANP-N-------VI----EVIS-SGKV------
---SE------DE-KFKEFFYCACND--IGVV----------------------------
-NP---D--G-H---------IKVKECIELFPK----------ETQPL--VEP-VIKNC-
-----DK-------EGV------NK------------------YDTLFKYL-KCFQETSP
V---R---

--VTL----A------

>Bmor-OBP23_NP_001153665_

---------------------------MTSK-----------------------------
-------------------------------------VLL--SCV--VL-------AVLA
TTVLAEDS------------------------R---KLVS--------FAPE-VAKKLKV
LIQECLNEN-----------------GLGE-D-------AI----EVIR-AGEY------
---RE------DE-PFQNLVYCAYKK--FGAL----------------------------
-DE---N--N-R---------IISQVAAASFPK----------DID----VVT-VIESC-
-----GKE------DGN------TP------------------VEQVFKYF-KCFQKNSP
V---R---

--MQL----Y------

>Bmor-OBP25

---------------------------MKSV-----------------------------

-------------------------------------VLI--CLA---------------
-FAVFNCG------------------------A---DNVH--------LNED-EREKANW
YTAECGVET-----------------GVST-E-------VI----NAAK-IGKY------
---SK------DK-AFKKFVLCFFKK--SAIL----------------------------
-NS---D--G-T---------LNMVVALAKLPSG---------VNKSE--AQS-VLEQC-
-----KNK------TGQ------DA------------------ADKAFAIL-QCFHKGTK
T---H---

--ILF-----------

>Bmor-OBP26_NP_001159622_

---------------------------MKSV-----------------------------
-------------------------------------VLI--CLA---------------

-FAVFNCG------------------------A---DNVH--------LAET-QKEKAKQ

YTSECVRES-----------------GVST-E-------AI----NAAK-IGKY------

---SK------DK-AFKNFVLCFFNK--SAIF----------------------------

-NS---D--G-T---------LNMDVALAKLPPG---------VNKSE--AQS-VLKQC-

-----KNK------TGQ------GA------------------ADKAFEIF-RCYYKGTK

T---H---

--ILF-----------

>Bmor-OBP27_NP_001106744_

---------------------------MKSV-----------------------------

-------------------------------------VLI--CLA---------------

-FAVFNCG------------------------A---DNVH--------LTET-QKEKAKQ

YTSECVKES-----------------GVST-E-------VI----NAAK-TGQY------

---SE------DK-AFKKFVLCFFNK--SAIL----------------------------

-NS---D--G-T---------LNMDVALAKLPPG---------VNKSE--AQS-VLEQC-

-----KDK------TGQ------DA------------------ADKAFEIF-QCYYKGTK

T---H---

--ILF-----------

>Bmor-OBP28

---------------------------MLKV-----------------------------

-------------------------------------FIV--TFF--AF----------Q

LSAIARLQ------------------------ANGCVAVP--------FPKD-KTIIIVE

AMKSCIAKT-----------------GANP-N-------FI----DVIR-SGKV------

---SE------DE-KFKEFYYCTCND--TGFV----------------------------

-NP---D--G-H---------IKVKECIELFPK----------ETQPL--VEP-VIKNC-

-----DKE------EGV------NK------------------YDTLFKFL-KCFQETSP

V---R---

--VAL----A------

>Bmor-OBP29

---------------------------MTGP-----------------------------

-------------------------------------AAA--AVL---LA------LLAA

AGQATTGC------------------------K---NCVI--------LGKE-ERAMFRS

HSDACLAQS-----------------RVEP-R-------LL----ESMM-NGEL------

---ID------DA-ALRKHVYCVLLS--CKMI----------------------------

-GK---D--G-K---------LLKAAILGKLAAR---------PAGRD--VTK-VLEAC-

-----AEQ------PGA------SP------------------EDVAWNIF-RCGYNRKA

V---L---

--FDYMPAGGASSGNT

ENHP--

>Bmor-OBP30

---------------------------MRSF-----------------------------

-------------------------------------VIL--LNYGLLCCGQFMAEDYYY

DIVTRDPD----------------------------DLMR--------EKEN-EVRALRA

FQADCAEDV-----------------QVKP-D-------LV----VNLK-SGDWQ-----

---TE------DV-SLKKWALCVLMK--LGLM----------------------------

-TA---Q--G-V---------FKMNEAMSKIPDM---------NDKII--AEK-LIDDC-

-----LSL------QAT------TP------------------HDAAWNYI-KCHHQKDP
E---G---

--NFS----SLNIF--

>Bmor-OBP31_NP_001040212_

---------------------------MKTF-----------------------------
-------------------------------------IVF--VVC---VV----------
------------------------------------LAQA--------LTDE-QKENLKK
HRADCLSET-----------------KADE-Q-------LV----NKLK-TGDFK-----
---TE------NE-PLKKYALCMLIK--SQLM----------------------------
-TK---D--G-K---------FKKDVALAKVPNA---------EDKLK--VEK-LIDAC-
-----LAN------KGN------SP------------------HQTAWNYV-KCYHEKDP

K---H---

--ALF----L------

>Bmor-OBP40

---------------------------MSEF-----------------------------
-------------------------------------IQP--SWR-------------TQ
CNFRLNWD--NRNR-LSIDISH
GAATTQTPV-----------------PTTKPK-------AL----RDFM-----------
-----------VVPQSCDKTTCVFKK--LNIV----------------------------
-SD---K--G-V---------VDVKSFIKLLDKFTNS----YPVWNSA--KAR-VITTC-
-----LRK------SLI----AYD-------------------GGCELNNILACTFDVLS
E---

--NCP----LNGNNQT

C---

>Bmor-OBP41

---------------------------MLTI-----------------------------

-------------------------------------LFL--LPI--VV---------GV

LSGNIPE---QPRV-YCGELPN

TIYSCLGNP-----------------KIIQ-P-------EV----SEKC-N---------

-----------KPISECDKTRCIFKE--SGWA----------------------------

-KN------N-V---------IDKKKVSDYFEQFAKD----NPDWSAA--VQN-FKTTC-

-----LSD------SLK----PQGV--------------D---TNCPAYDIIHCALISFI

----K---

--FASPS--QWSTSEQ

CVYPRQYAGACPVCPERCFAPSVPNGSCNACLALLRTP----------------------

>Bmor-OBP42_NP_001159621_

---------------------------MMGY-----------------------------

-------------------------------------ACV--FVI--LA---------VL

QAISAEDP------------------------P---GLPPFLKDA---PEKC-KSPPRVK

NPNECCISE-----------------PFFKEA-------DF----IECG-IEKPGSE---

-----------RGPPDCSKQNCLLKK--YNLL----------------------------

-KN---D--E-T---------PDIEAIKSLLDKYIEK----NPSFKSS--VEK--AKEC-

-----LRE------DLP----GPP-------------------QICLANRMTLCIGTVLL

----M---

--ECPDE--KWNTTDD

CKAFKDHMTECQKYFPK---

>Bmor-OBP43

---------------------------MKVC-----------------------------

-------------------------------------VLF--AIF---------------

--TVAQ-AAKATLK

PISACCNIP-----------------ELGNPE-------PL----AECS-NP--------

-----------KLPGPCKDIQCVFEK--SGFL----------------------------

-TE---N--K-T---------LIKEAYKTHLRQWAKE----HEGWSVA--VEK-AISDC-

-----VDK------DLR----QYLE------------------FPCSAYDVFTCTGIAML

----K---

--KCP------NEHWT

C---

>Bmor-OBP44

---------------------------MSRL-----------------------------
-------------------------------------VLF--FTI--LV-----------
--------------------------------V---LQEF--------IINL-YFNFITE
IDSCCVKKY----------------PKLFDSE-------FI----TECY-NTQR------
-----------KANDKCERDMCVARK--LNLL----------------------------
-TE---E--D-S---------INKDALLRFVEEGFKT----EIDLVNA------IKKKC-
-----FEE------DIS----NIGK--------------P---EMCEVAKYKICITSRMA
----E---

--DCP----KWDSKGI
CSSAQQKVENFMKMLS--

>Apol-PBP1_X17559_

----------------ML--RKI---SL--------------------------------
--L----LL---------PV
FVAINL-V------------------------H---SSPE--------IMKN-LSNNFGK
AMDQCKDEL-----------------SLPD-S-------VV----A-DL-YNFWKDD--Y
--VMT------DR-LAGCAINCLATK--LDVV----------------------------
-DP---D--G-N---------LHHGNAKDFAMKHGAD----E-TMAQQ--LVD-IIHGC-
-----EKS------APP----ND-D--------------K---CMKTIDVA-MCFKKEIH
K---L---

--NWV----PNMDLVI
GEVLAE--

------------------------------V-----------------------------

>Apol-PBP2_AJ277266_

----------------M---------SL--------------------------------
---------------------------------------S--L----FL---------AM
LVTINL-V------------------------Q---ASPE--------IMKN-LCMNYGK
TMDQCKQEL-----------------GLPD-S-------VI----N-DL-YNFWKDD--Y
--VMT------DR-LAGCAINCLSTK--LDIV----------------------------
-DP---D--G-N---------LHHGNAKEFAMKHGAD----D-GMAQQ--LVD-IIHRC-
-----EKS------TPP----ND-D--------------K---CTKTMDIA-MCFKKEIH
K---L---

--NWV----PNMDLVV
CEVLAV--

------------------------------V-----------------------------

>Apol-PBP3_AJ277267_

----------------MI--AKT---FN--------------------------------
---------------------------------------L--L----VI---------VY
LSTNTG-V------------------------D---ASQE--------IMKT-MTLTFTK
GLDACKKEM-----------------DLPD-T-------VD----V-DF-NNFWKED--Y
--VVT------NR-DAGCAIVCLASK--INLV----------------------------
-DS---M--G-I---------LIHGSAHEFAKQHGAD----D-NMAKQ--LSD-TLHTC-
-----ETI------IGT----GN-D--------------E---CTRALHVA-NCFKVEMH
K---L---

--DWA----PSMDLII
GELLAE--

------------------------------I-----------------------------

>Aper-GOBP1_Y10970_

---------------------------MAHT-----------------------------

--------------------------------------LQ--TVV--LL---------LG

TSILHP-I------------------------L---ADVN--------VMKD-VTLGFGQ

ALEKCREES-----------------QLTE-E-------KM----E-EF-FHFWSED--F

--KFE------HR-ELGCAILCMSRH--FNLL----------------------------

-TD---S--S-R---------MHHENTDKFIKSFPNN----E-VLSKH--MVN-LIHSC-

-----EQQ------HDA----DL-D--------------H---CWRILRVA-ECFKRSCQ

E---A---

--GVA----PSMELLM

AEFIMESEI---

-N--

>Aper-GOBP2_X96772_

---------------------------MGYK-----------------------------

--------------------------------------LL--LMY--------------I

AIVIDS-V------------------------I---GTAE--------VMSH-VTAHFGK

ALEECRDES-----------------GLSP-E-------IL----N-EF-KHFWSED--F

--DVV------HR-ELGCAIICMSNK--FSLL----------------------------

-KD---D--T-R---------IHHVNMHDYVKSFPNG----E-VLSAK--MVN-LIHNC-

-----EKQ------YDD----IT-D--------------E---CDRVVKVA-ACFKVDAK

K---E---

--GIA----PEVAMIE

AVIEKY--

>Aper-PBP1_X96773_

----------------ML--GKI---SL--------------------------------

--L----LL---------PV

FVAINL-V------------------------H---SSPE--------IIKN-LSQNFCK

AMDQCKQEL-----------------NIPD-S-------VI----A-DL-YNFWKDD--Y

--VMT------DR-LAGCAINCMATK--LDVV----------------------------

-DP---D--G-N---------LHHGNAKEFAMKHGAD----A-SMAQQ--LVD-IIHGC-

-----EKS------APP----ND-D--------------K---CMKTIDVA-MCFKKEIH

K---L---

--NWV----PDMDVVL

GEVLAE--

------------------------------V-----------------------------

>Aper-PBP2_X96860_

----------------MI--RKV---LL--------------------------------

---------------------------------------S--V----LL---------AV

LMTINL-G------------------------Q---ASPE--------VMKN-LCMNYGK

AMDQCKQEL-----------------NLPD-S-------VI----A-DL-YNFWKDD--Y

--VMT------DR-LAGCAINCLSTK--LDIV----------------------------

-DP---D--G-N---------LHHGNAKEFAMKHGAD----D-GMAHE--LVD-IIHGC-

-----EKS------SPP----ND-D--------------K---CIKTMDIA-MCFKKEIH

K---L---

--NWV----PNMDLVV

GEVLAE--

------------------------------V-----------------------------

>Aper-PBP3_AJ277265_

----------------MI--AKT---FN--------------------------------

---------------------------------------L--L----VI---------VY

LSTNTA-V------------------------D---SSQD--------VMKS-MTLTFTK

GLDACKKEM-----------------DLPD-T-------ID----V-DF-NNFWKED--Y

--VVT------NR-NAGCAIMCLASK--VDLV----------------------------

-DS---M--G-I---------LIHGSSHEFAKQHGAD----D-NMAKQ--LSD-TLHSC-

-----EKT------IGT----LN-D--------------E---CLRALNVA-NCFKVEIH

K---L---

--DWA----PSMDLII

GEILAE--

------------------------------I-----------------------------

>Hvir-GOBP1_X96862_

---------------------------M--------------------------------
--------------------------------------PG--VLR--AL---------LL
LAAAAP-L------------------------L---ADVN--------VMKD-VTLGFGQ
ALDKCREES-----------------QLTE-E-------KM----E-EF-FHFWRDD--F
--KFE------HR-ELGCAIQCMSRH--FNLL----------------------------
-TD---S--S-R---------MHHDNTEKFIQSFPNG----E-VLARQ--MVE-LIHSC-
-----EKQ------FDH----EE-D--------------H---CWRISHLA-DCFKSSCV
Q---R---

--GIA----PSMELMM
TEFIMEAEA---

-R--

>Hvir-GOBP2_X96863_

---------------------------MTSK-----------------------------

--------------------------------------SC--LLL------------VAM

VTLTTS-V------------------------M---GTAE--------VMSH-VTAHFGK

ALEECREES-----------------GLSA-E-------VL----E-EF-QHFWRED--F

--EVV------HR-ELGCAIICMSNK--FSLL----------------------------

-QD---D--S-R---------MHHVNMHDYVKSFPNG----H-VLSEK--LVE-LIHNC-

-----EKK------YDT----MT-D--------------D---CDRVVKVA-ACFKVDAK

A---A---

--GIA----PEVTMIE

AVMEKY--

>Hvir-PBP1_X96861_

----------------MM--SVR-------------------------------------
---------------------------------------L--M----LV---------VA
VWLCLR-V------------------------D---ASQD--------VMKN-LSMNFAK
PLEDCKKEM-----------------DLPD-S-------VT----T-DF-YNFWKEG--Y
--EFT------NR-HTGCAILCLSSK--LELL----------------------------
-DQ---E--M-K---------LHHGKAQEFAKKHGAD----D-AMAKQ--LVD-MIHGC-
-----SQS------TPD----ATDD--------------P---CMKALNVA-KCFKAKIH
E---L---

--NWA----PSMELVV
GEVLAE--

------------------------------V-----------------------------

>Hvir-PBP2_AM403491_

--------------------PKW---VF--------------------------------

---------------------------------------A--R----AF---------CL

VLMMGS-A------------------------M---SSKE--------LLTK-MTGGFTK

VVDHCKTEL-----------------NVGD-H-------IM----Q-DM-YNFWREE--Y

--QLV------NR-DLGCMIMCMTAK--LDLV----------------------------

-GD---D--Q-K---------MHHGKAEEFAKSHGAD----D-ALAKQ--LVG-LIHGC-

-----ETQ------HQA----IE-D--------------H---CSRTLEVA-KCFRTKIH

E---L---

--KWA----PSMEVIM

EEIMTA--

------------------------------A-----------------------------

>Slit-GOBP1

--------------------------------------ML--LLL--AL---------PL
LAAVLP-L------------------------R---ADVN--------VMKD-VTLGFGQ
ALDKCRQES-----------------QLTE-E-------KM----E-EF-FHFWRDD--F
--KFE------HR-ELGCAIQCMSRH--FNLL----------------------------
-TD---S--S-R---------MHHENTEQFIQSFPNG----E-VLARQ--MVE-LIHAC-
-----EKQ------HDH----ED-D--------------H---CWRILHVA-ECFKQACV
Q---R---

--GIA----PSMEMMI
TEFIMEAEA---

-R--

>Slit-GOBP2

---M

ATVTSS-V------------------------M---GTAE--------VMSH-VTAHFGK

ALEECREES-----------------GLSA-E-------VL----E-EF-QHFWRDD--F

--EVV------HR-ELGCAIICMSNK--FSLL----------------------------

-QD---D--S-R---------MHHVNMHDYVKSFPNG----H-VLSEK--LVG-LIHNC-

-----EKQ------FDS----MT-D-------------D---CERVVKVA-ACFKVDAK

A---A---

--GIA----PEVAMIE

AVMEKY--

>Slit-PBP1_EF396284_

----------------MA-NARW---RF--------------------------------

---------------------------------------V--F----VV---------YA

LYLTSA-V------------------------L---GSQD--------LMAK-MTKGFTR

VVDDCKTEL-----------------NVGD-H-------IM----Q-DM-YNYWRED--Y

--QLI------NR-DMGCMLLCMAKK--LDLM----------------------------

-DD---Q----T---------MHHGKTEDFAKSHGAD----D-DVAKK--LVS-VIHEC-

-----EQQ------HTG----IA-D--------------D---CMRVLEVA-KCFRTKIH

E---L---

--KWA----PSMEVIM

EEVMTA--

------------------------------V-----------------------------

>Slit-PBP2

----------------M---SLR-------------------------------------

---------------------------------------V--A----LV---------VA

ASLLVV-V------------------------Q---ASQD--------VMKN-LAINFAK

PLDDCKKEM-----------------DLPD-S-------VT----T-DF-YNFWKEG--Y

--ELT------NR-QTGCAILCLSSK--LEIL-----------------------------

-DQ---E--L-N---------LHHGRAQEFAMKHGAD----E-AMAKQ--IVD-MIHTC-

-----AQS------TPD----VAAD--------------P---CMKTLNVA-KCFKLKVH

E---L---

--NWA----PSVELIV

GEVLAE--

------------------------------V-----------------------------

>Slit-PBP3

----------------MG--SRN---VF--------------------------------

---------------------------------------V--A----LV---------VL

TVAMRE-T------------------------E---PSKD--------PMKY-IASGFVK

VLEECKHEL-----------------NMND-H-------LI----A-DL-FHYWKLE--Y

--TLL------NR-DTGCAIICMGKK--LDLL----------------------------

-DA---N--G-R---------MHHGNAQEFAKKHGAG----D-EVASQ--IVQ-IIHEC-

-----EKK------HER----DD-D--------------E---CLRVLEVA-KCFRTGIH

E---L---

--NWQ----PNVEVIV

SEVLTE--

------------------------------I-----------------------------

>Asel-PBP_AB285328_

----------------MQ--RSW---CFYCR-----------------------------

-------------------------------------LLV--V----LG---------VF

LALVQE-R------------------------E---CSQE--------VMHN-LSKGFAE

VLEDCKKQE-----------------NVGD-H-------IM----Q-DF-YNFWHEE--Y

--SLV------NR-EMGCIILCMAGK--LDLL----------------------------

--D---G--D-T---------MHHGNAHEFAKKHGAD----D-ALAKQ--LVG-LVHEC-

-----EQA------SAS----VE-E--------------R---CARALETT-KCFRGKIH

G---L---

--KWA----PSMRVVM

EEVMAD--

------------------------------M-----------------------------

>Asel-PBP2_AB327273_

----------------MR--TNK-------------------------------------

---------------------------------------V--V----LI---------LL

MSMIAI-V------------------------T---PSQD--------VMKT-LTINFGK

PMEICKKEL-----------------DLPE-A-------VT----K-EF-LNFWRDG--Y

--EVT------NR-LTGCAIMCISEK--LELL----------------------------

-DE---G--Y-K---------LHHGNAKDFAMKHGAD----A-GMAQQ--LVD-IIHGC-

-----NES------TPD----NT-D--------------H---CLKTVAVA-MCFKHKIH

E---L---

--DWA----PNADLII

AEVLAE--

------------------------------V-----------------------------

>Eobl-GOBP1_FJ156732_

---------------------------MAR------------------------------

--------------------------------------LA--SSV--LA---------VV

AVAAAA-V------------------------V---ADVQ--------VMKD-VTLGFGQ

ALEVCREES-----------------QLSQ-D-------VM----E-EF-FHFWRED--F

--KFE------SR-AVGCALQCMSRH--FNLL----------------------------

-TD---S--S-R---------MHHENTHRFIESFPNG----S-VLAKQ--MVS-LIHGC-

-----EQQ------HEA----EP-D--------------H---CWRILRVA-ECFKRRCQ

E---A---

--GIA----PSMEIIM

AEFIMETEA---

-K--

>Eobl-GOBP2_FJ156733_

---------------------------MKSV-----------------------------

--------------------------------------LV--ATV--------------V

LSVVGL-A------------------------M---GTAE--------VMSH-VTAHFGK

ALSECREES-----------------GLTP-E-------VL----E-EF-QHFWRED--F

--EVV------HR-ELGCAIICMSNK--FSLL----------------------------

-QE---D--S-R---------IHHVNMHDYVKGFPNG----Q-VLSAK--MVE-LIHNC-

-----EQQ------YDD----IT-D--------------D---CARVVKVA-ACFKRDAK

K---E---

--GIA----PEVTMIE

AVMEKY--

>Pxyl-GOBP1_EU368114_OBP1_JN088193_

---------------------------MERR-----------------------------

--------------------------------------WC--LLV--LA---------AA

AAGLPGVV------------------------R---GTVE--------VMKD-VTLGFGE

ALEQCREQS-----------------QLTE-E-------MM----E-EF-YHFWRED--F

--KFE------AR-AVGCAIHCMSRY--FNLL----------------------------

-GE---Q--Q-R---------MHHDNTHKFIQSFPNG----E-VLSHQ--MVG-IIHTC-

-----EQQ------HDA----ET-D--------------H---CWRILRVA-ECFKRESQ

A---Q---

--GLA----PSMEMLM

AEFIMEADV---

>Pxyl-GOBP2_EU368115_OBP2_JN088194_

---------------------------MASV-----------------------------

--------------------------------------WS--LVV--CG---------LM

MAALPA-A------------------------R---GTAE--------VMSH-VTAHFGK

TLEECREES-----------------GLSG-E-------IM----E-EF-HHFWRED--F

--EVV------HR-ELGCAIICMSNK--FQLM----------------------------

-QD---D--A-R---------MHHENMHDYIKSFPKG----D-LLSET--MVR-LIHNC-

-----EKK------YDD----ID-D--------------E---CSRVVKTA-ACFKKDAQ

A---E---

--GIA----PELTMIE

AVLEKY--

>Pxyl-PBP2_AB263118_

----------------MV--KMITKKPA--------------------------------

---------------------------------------C--L----MM---------VL

MCALKK-V------------------------E---SSAD--------VMKG-LSENFGK

ALGDCKKEL-----------------DLPD-S-------IM----T-EF-YNFWKDD--Y

--VLS------DR-STGCAIICLSSK--LDLL----------------------------

-DP---D--G-N---------LHHGNAKDFALKHGAD----E-GMAGQ--LVG-MIHEC-

-----EKA------APD----NP-D--------------A---CLKVLDIA-NCFKKKIH

E---L---

--KWA----PSMDVVV

AEVLAD--

------------------------------V-----------------------------

>Pxyl-PBP1_FJ201994_

----------------MW--FPV---RL--------------------------------

---------------------------------------S--A----VL---------LI

TLAVMD-T------------------------Q---GSKE--------TMKD-ITSGFFK

VLNECKHEL-----------------NLPD-H-------LV----G-DF-YHYWRQE--Y

--ALL------DR-DLGCAILCMSRK--LELI----------------------------

-DA---S--G-K---------LHHGNTQEFAEKHGAD----N-SMASK--LVE-VLHSC-

-----EKQ------HEA----VS-D--------------E---CQRALEVA-KCFRSSVH

E---L---

--GWA----PTIDVII

EEVLTD--

------------------------------M-----------------------------

>Pxyl-OBP3_JN088195_

IAMNCTKKY-----------------PLEV-H-------EI----LDLQ-KSKV------
---PT------KK-TAKCLLACAYRL--EGSM----------------------------
-NE---K--G-L---------LDYEHMMKTADLLADG----DEKRL--------------

>Pxyl-OBP4_JN088196_

---EEFIQ
LGMECAKQH-----------------QVTP-E-------EV----QLMH-QHVI------
---PD------GR-GARCLVACVFKK--KDLI----------------------------
-ND---K--G-M---------LDIDAAHSMADKEHLD----DPTMIE-------------

>Dple-GOBP1_DPOGS210500_mod_EHJ71301_AB_DpOBP1_DPGLEAN01448_

---------------------------MTSR-----------------------------
-------------------------------------SGR--RLL--LL---------LL
VQLAVQ-L------------------------K---ANVD--------VMKD-VTLGFGE
ALKLCREES-----------------QLTE-E-------KM----E-EF-FHFWRDD--F
--KFD------DR-AVGCAIKCMSSH--FDLL----------------------------
-TD---S--H-R---------MHHRNMDNFIKSFPNG----E-VLSQQ--MVT-LIHEC-
-----EQQ------HDS----EE-D--------------H---CWRILRVA-ECFKSSCK
K---H---

--GIA----PTMELLM
AEFVMESEA---

-N--

>Dple-GOBP2_DPOGS210512_mod_EHJ71306_AB_DpOBP2_DPGLEAN01461_

---------------------------MAVR-----------------------------
--------------------------------------GL--LLL--SA---------VL
AATSVT-V------------------------D---GTAE--------VMSH-VTAHYGK
SLEECRKET-----------------GLSK-E-------IL----E-EF-KHFWSDD--F
--EIV------HR-PFGCTLICMSNK--FALL----------------------------
-QD---D--A-R---------MHHINMNDYVKGFPEG----D-VLADK--LVQ-MIHKC-
-----EKE------YDD----IK-D--------------D---CDRVVKVA-ACFRADAK
K---E---

--GIA----PEVAMIE
AVMEQY--

>Dple-PBP-B_DPOGS210513_EHJ71307_AB_DpOBP5_DPGLEAN01462_

-------------------------------------MAI--MMF--AI---------FI
LSIFIPSV------------------------N---LNQD--------VMKS-LSYKFGT
KLFECGERT-----------------NYTR---------AM----ARDI-LHIWEES--Y
--DLN------HD-ETGCLVLCAMVR--LELL----------------------------
-DQ---Q--G-N---------MIVENTEGFIRANGGD----D-SMVSF--LIQ-LYSMC-
-----REK------TSS----ISNG-----------------CKAAIELS-KCFRAAIQ
Q---I---

--GWV----PDTTSLL
VISYD---

>Dple-PBP-C_DPOGS210514_EHJ71308_AB_DpOBP6_DPGLEAN01463_

----------------MN--KSC-------------------------------------
---------------------------------------V--F----VF---------AL
IFVNVQ-K------------------------V---QSNE--------VMKG-ITSSFFK
VLDECKREL-----------------GLTD-N-------VL----T-DL-YYFWKQD--H
--PLM------HR-DTGCAIVCMSQK--LNLL----------------------------
-DT---I--G-K---------LHHGNAQEFAINHGAG----E-QMAKK--LVT-MVHEC-
-----EQQ------FME----QE-D--------------S---CLRALDVA-KCFRTAMH
D---V---

--NWA----PKFDIIV
TEVLTEVK--

>Dple-PBP-D_DPOGS210497_EHJ71309_AB_DpOBP3_DPGLEAN01445_

----------------MALFC-W---RL--------------------------------

---------------------------------------L--F----AA---------GV

LSLAQG-T------------------------L---ASQE--------IMKK-LTTGFVK

AMEECKAEL-----------------NLGD-H-------II----Q-DF-MNYWREE--Y

--ELL------NR-DTGCAIMCMASK--HDLI----------------------------

-TE---D--M-K---------IHHENAHEFAKSHGAD----D-DLAKQ--LVQ-MIHDC-

-----EKQ------FTD----IT-D--------------D---CSKTLEIS-KCFRTKIH

E---L---

--KWA----PSMETIL

EELMTE--

------------------------------T-----------------------------

>Dple-OBP2_DPOGS207887_EHJ65656_AB_DpOBP19_DPGLEAN06529_

---MKKWFLE

LTVECSKEH-----------------PVTK-E-------EI----QMLK-DHKI------

---PD------NK-NVKCLMGCVFRK--IGWL----------------------------

-DD---N--G-M---------FSFNNAYKTSEEEYPD----DKTKLEK--AKN-LYSLC-

-----EKV------NTA----EVSD--------------GKEGCERSSLLA-KCLIENSS

K---M---

--GFV----VQ-----

>Dple-OBP3_DPOGS207885_EHJ67765_AB_DpOBP9_DPGLEAN10496_

------------------------------------MTAE--------QKQM-VHQHFEQ
VGMECIKTH-----------------EITA-E-------DV----TNLR-TRKI------
---PT------GE-NASCFLSCIFKH--VGIM----------------------------
-DD---N--G-L---------LQKESAIELAKKVFDD-----EEELKL--IED-YLHSC-
-----SSV------NTA----TVSD--------------GEKGCERSLLAY-KCMIENAS
Q---F---

--GID----L------

>Dple-OBP4_DPOGS207878_EHJ67766_AB_DpOBP11_DPGLEAN10492_

---------------------------MMSL-----------------------------

-------------------------------------KYL--FVF--GV----------L

AVSLRSAR------------------------A---ISDE--------DRNK-IHTAVLP

HVAECSANF-----------------GVTE-D-------DI----KAAK-EAGT------

-----------LGTFNPCLMGCVLKK--IQVI----------------------------

-DD---K--G-L---------FDADKAVELSNKYFSD-----EADKKK--AEE-IITTC-

-----KAV------NDK----EVGD--------------GEKGCERAGLLF-QCFLPFKD

A---F---

--IN------------

>Dple-OBP5_DPOGS207877_EHJ65653_AB_DpOBP14_DPGLEAN06527_

---------------------------------------M--------TRQQ-IKNSSKM
LKKNCMGKN-----------------DVTE-D-------MV----GEID-KGKF------
---IE------DK-NVMCYIACIYQM--SQLV----------------------------
-KN------N-K---------LNYEASIKQVDLMFPP------DMKEA--MKA-SIENC-
-----KDI------SKK----YKDI------------------CEASYWTA-KCIYDDNP
K---N---

--FIFA----------

>Dple-OBP6_DPOGS207886_EHJ65655_AB_DpOBP18_DPGLEAN06528_

---------------------------------------M--YIL-------------AV

VKFLAALT------------------------I---CEAM--------TMKQ-IKSTGKM

MRKTCQPKN-----------------NVAD-D-------KI----DDIG-TGVF------

---IE------ER-EVMCYVACIMKM--ANAI----------------------------

-KN------N-K---------LNYEAAIKQADLLLPD------EIKEP--TKE-AITAC-

-----RKV------ADS----YKDI------------------CEASFHVT-KCIYNHNP

S---I---

--FFFP----------

>Dple-OBP7_DPOGS207876_EHJ65654_AB_DpOBP16_DPGLEAN06526_

---------------------------------------M--------TRAQ-LKKTMTI
MKNQCMPKH-----------------NVNN-D-------KV----GQIE-QGVF------
---IE------DH-DVMCYIACIYKT--IQVV----------------------------
-KN------N-R---------LDKDLISKQIDALYPP------ELKES--TKE-AVSKC-
-----IKE------QAK----YEDP------------------CAGIFYAC-KCLYEDNP
A---N---

--FIFP----------

>Dple-OBP8_DPOGS208192_ABPX_EHJ75781_AB_DpOBP20_DPGLEAN20037_

---MAELAKM

LRDNCLEET-----------------GADV-A-------LI----DKVN-QGATL-----

---MP------DG-KLKCYIKCVMET--AGMM----------------------------

-SG------G-E---------VDVEAVVAVLPE----------ELQRH---AD-TMRAC-

-----GTK------RGL------DD------------------CDTAFLTQ-ACWQAGCP

K---D---

--YILI----------

>Dple-OBP14_DPOGS205435_combinedwithDPOGS205437_EHJ74349_AB_DpOBP32_DPGLEAN22247_

---------------MNTWSI------LFTL-----------------------------

-------------------------------------TII--LLG--TI---NCYKHSKY

LTKSLDSE----------------------------PSLS--------ILYA-RDKKSDT

ITNECLMEM-----------------YPRN---------------LYKY-PLHI------

-----------DRNDIPCIIHCVLKK--FGIM----------------------------

-SN---D--G-I---------INIRNYYRRVQAIHRY------DPRVL--ISD-VGETC-

-----AQN------ING-MNLDHDV------------------CKKAKVFN-DCTQLYVI

SYKDL---

--ED------------

>Dple-
OBP15_DPOGS205438_sameasDPOGS205436_EHJ74350_AB_DpOBP33_DPGLEAN22248_

---MYA-HEKISDM

VAQQCLTEM-----------------YPKG---------------KKIE----L------

-----------QESDESCIIYCVLKK--FGII----------------------------

-NG---N--G-Q---------FNLDIYRKRVQMAHQL------DSRNL--MND-KGGAC-

-----VES------AEA-TQHKQDV------------------CKKAKVFN-DCTHLYRM

I---F---

>Dple-OBP16_DPOGS205439_EHJ74351_AB_DpOBP34_DPGLEAN22249_

-----------------------MQLYVFLC-----------------------------

-------------------------------------LIG--HVL--AS---------KS

HGYSGTLV------------------------D---FTDP--------KVQG-HLDALVR

MAQSCVIKV-----------------RASP-K-------DV----RAYF-TNSP------

---PI------TR-SGQCFAACMMEQ--SDVI----------------------------

-NH------G-K---------VNRELLIHLASLVNGK----NSGVVRK--LHS-ISRLC-

-----LDS------IEG----MSDR------------------CQLASTYN-DCLNENMI

E---F---

--AFP----LDIAEEA

VRKMPFHLIQPK--

>Dple-OBP17_DPOGS200530_EHJ67147_AB_DpOBP36_DPGLEAN02358_

--MTKEEQ------------------------I---DRFN--------KMNE-EVEPFRQ

NLTDCARQV-----------------KASM-I-------DV----EHFL-KRIP------

---QS------SM-QGKCFVACILKR--NSII----------------------------

-VN------N-K---------ISRENLLEANRAVYGQ----DSEVMTR--LNT-AIVEC-

-----SDV------VEG----IFEV------------------CEYSSVFN-DCMHMKME

H---I---

--LDK----VTMERRM

EALGQMTSDPDVWTDEDDEILKLIKDEL--------------------------------

>Dple-OBP18_DPOGS216124_EHJ64212_AB_DpOBP35_DPGLEAN1205_

--MFLRFLFPLMFFVYVE

TDQAVENNRMVGVDTVHDIKIDKDTIITRNM-----------------------------

-------------------------------------NLK--KKNEKSS----EAQIEQK

NVSPDWSY------------------------S---SFPE--------DVKK-HVDQFKR

NMSECLKEV-----------------HASD-K-------RP----VKRL-SPKK------

---ES------PV-HGDCLIACVLKR--NSVI----------------------------

-EN------G-K---------IHKENLIALVKKFYEK----DEKLMKK--LER-NLDRC-

-----IET------SAR----DKDD------------------CAIAARLN-ECTNDIMT

S---N---

--KHK----IVVNY--

>Dple-OBP19_DPOGS203084_EHJ68962_AB_DpOBP37_DPGLEAN13103_

---------------------------MNLY-----------------------------

-------------------------------------GVH--PYV--------------K

LSPERFYL------------------------W---TDSP--------IYAV-VHKTLSM

TARSCMVSA-----------------NASE-V-------DL----E-PL-REDP------

---PF------PE-KSACILNCLLEK--INIV-----------------------------

-KS------G-K---------FSKSGFMTTISPLVFT----NKKKMDH--MKT-VSENC-

-----DKE------VNH----DVSS------------------CELANEIT-SCIYKYAP

E---L---

--HFK----N------

>Dple-OBP20_DPOGS212737_EHJ78968_AB_DpOBP38_DPGLEAN10161_

--------------------MRVLVVAAVTL-----------------------------

-------------------------------------ITI--TLT--RCVPRTDKMQNNV

KDLTSTTP------------------------N---SMEE--------QMDK-ISNDLRP

VMAECNETF-----------------RIEM-Y-------YL----E-TL-NTTGSFP---

---EE------LDRTPKCFLRCVLEK--AEVA-----------------------------

-SA---D--S-Q---------FDVSRTADVFDQIRVL-------PHDD--LVK-MATTC-

-----SDR------AET-------C-------------K---CERAYQYL-KCLMGMII

N---K---

--YDT----T------

>Dple-OBP1_DPOGS207879_DUPLEX_EHJ67764_EHJ67763_AB_DpOBP7_DPGLEAN10494_AB_DpOBP8_DPGLEAN10493_

--MECSKDYPLTDEDIAQLKDK

QFPDKDDVRCLFACAYKKTGMMDDQGKLSVD-----------------------------

-------------------------------------GVNNLAKKYFSDDQDKLQKSQKF

TEACAGVNDEAVTDGEKGCERAALIYKCSIEQA---SQGL--------TDEE-VKKEFIK

EVMTCTKDI-----------------TVDM-F-------DL----MELE-QLKV------

---PT------KT-NVKCVLACAYKR--VGTM----------------------------

-NK---E--G-K---------YDIKEAYKISETMMKG----DDKRIEN--GKK-LADLC-

-----SKV------NEA----DVRD--------------GNKGCERAALLF-KCVIENAP

K---L---

--GFK----V------

>Dple-OBP9_DPOGS203667_EHJ74653_AB_DpOBP21_DPGLEAN11049_

---------------------------MPSL-----------------------------
-------------------------------------SLL--LYL-------------AM
LTLCYGAK----------------------------EKPE--------LSEE-IKEIIQH
VHNECVGKT-----------------GVAE-E-------DI----ANCE-NGIF------
---KE------DM-KLKCYMFCLLEE--GSLV----------------------------
-DD---D--G-N---------VDYDMMVSLIPD----------QYTDR--VLN-MINGC-
-----KHLVLSISCDDS----ETSG------------------MQVPLEAP-KPSSSPSA
P---H---

--PRRGLDSDIGPVEN

TPRIFA--

>Dple-OBP10_DPOGS201921_EHJ66992_AB_DpOBP5_DPGLEAN08164_

---------------------------MKFL-----------------------------

-------------------------------------VLI--CVC---------------

AIFISRSN------------------------A---QNAH--------LTSA-QKEKVKQ

YTAECIRES-----------------GVKH-E-------VL----ADAK-KGNL------

---NE------DE-GLKRFIFCFFQK--SGIV----------------------------

-TA---D--A-K---------LNMEVALSKLPKD---------IDKVA--AGK-VLSEC-

-----KNK------NGK------NH------------------ADTAFQIF-KCYHKATK

Q---H---

--VLF----E------

>Dple-OBP11_DPOGS214842_EHJ69393_AB_DpOBP30_DPGLEAN14493_

---------------------------MSTK-----------------------------
-------------------------------------FYC--ILA-------------IV
LFFFVQVR----------------------------NTAS--------ITNY-KSPLIAE
YVEHCLRES-----------------RANP-E-------HI----IFLK-NGVIH-----
---SP------NY-ALKNWLLCYLSR--TGVM----------------------------
-SP---E--G-V---------LKQHVVMKKVAK----------QDKDL--VEK-IIDKC-
-----LFK------TPH------EP------------------VDTAWKYL-TCFRKRQP
Q---Y---

--ARE----INHI---

>Dple-OBP12_DPOGS214841_EHJ69392_AB_DpOBP31_DPGLEAN14492_

---------------------------MKTF-----------------------------
-------------------------------------IVL--AAI---LV----------
------------------------------------AAQA--------LTDE-QKQKLNK
HKSECLAEF-----------------KPDE-Q-------LV----EKLK-AGDFK-----
---TE------NE-PLKKYVLCMLMK--SELM----------------------------
-TK---E--G-K---------FKKDVALAKVPNP---------EDKPA--VEK-LIDAC-
-----LAN------KGN------TP------------------QQTAWNYA-KCYHEKDP
N---H---

--SIF----Q------

>Dple-OBP13_DPOGS209054_EHJ75212_AB_DpOBP29_DPGLEAN13901_

-------------------------------MCYWISTTGRDSEDDVVRILPSDVYKTND
EYRHIWSPVHGDAVVFVRPLLFHRLVVVLLQ-----------------------------
-------------------------------------VSL--QLS---QDDMLTLLVVLL
LHQVTTES------------------------K---SFMP--------LGKA-ERAMFLS
HSEACLEQS-----------------GAER-A-------QV----ERLV-GGAP------
---ED------SP-ALRRHVLCVLRR--CKLL----------------------------
-RK---D--G-R---------LDKHALRDRISA-------------SN--DTK-ILEGC-
-----SDQ------SGD------TP------------------EDLAWHLF-RCGLNKKV
----L---

--FEHMTAAAPNEA--

>Dple-OBP27_DPOGS208339_EHJ75008_AB_DpOBP26_DPGLEAN03483_

--------------------------------------MEEEISNALQDCSQLNDKLNIK
VNIEKRQRRHNDFRYYAFRIDANSKQEINQY-----------------------------
-------------------------------------NHE--RRN--TS---------TE
NDKNINGN------------------------D---SGIN--------FSRY-PYGGIEE
DLINSGNRQ-----------------TIDDDR-------KS----NLKQ-QSRFKRN--E
PLVSK------DD-VDKCLSQCVFAN--LQVV----------------------------
-DS---K--G-I---------PREAMLWNKIQSSVTS-----EQSETL--MRE-QIRSC-
-----FQE------LQS---ESEDN--------------G---CVYSNRLE-RCLMLHIS
D---R---

--NRN--------STQ
TYTTSIK---

>Dple-
OBP21_DPOGS210117_sameasDPOGS210118_EHJ65319_AB_DpOBP39_DPGLEAN12415_

----------------------MVSKISVLL-----------------------------

-------------------------------------CCI--YVF--GISLSDSAISADS

ESRCRNPP------------------------T---APQK--------IERV-ITLCQDE

IKLSILREA----------------LDVIKEE-------HT----MPAQ-RRRNKRE--V

PFTHD------EKRIAGCLLQCVYRK--VKAV----------------------------

-DG---Y--G-F---------PTLEGLVGLYSDGVNE-----RGYFMA--VLE-ASREC-

LMKNHDLF------SRT----VPMD--------------NGRNCDVSFNIF-ECISDRIG

E---Y---

--CGN--------SGL

>Dple-OBP23_DPOGS209968_EHJ77172_AB_DpOBP41_DPGLEAN05855_

---------------------------MMKY-----------------------------

-------------------------------------LFV--LIC--LS---------IA

TCEADDI---ASPV-LCGVTPN

AIYQCLGTP-----------------KVVK-K-------EA----SSQC-D---------

-----------SDLGECEKMSCIFSK--SGWM----------------------------

-KD------N-K---------VDKEEFAAHFDQFAKE----NPDWKVA--VEH-MKSNC-

-----LSS------DLP----PQGV--------------H---LNCPAYDVMICAFANFI

----K---

--GAPAS--QWSSSSH

CEYPRRFAASCPVCPTACFASAIPIGSCNACLSLPRSP----------------------

>Dple-OBP24_DPOGS201299_EHJ67714_

---------------------------MAEM-----------------------------

-------------------------------------THL--LTL--FV---------LF

LMSTVSTA------------------------D---EVDE--------SCFE-LFDPEEP

EQECCETDF-----------------ETDD---------EV----EYEE-DFSDCPT---

--DFS------TDEGKCDTIKCYYKH--DGVW----------------------------

-KD------D-D---------IDDEAVKTKLQSIDSK----NPPAQRA--AER-ILKHC-

-----LNG------KYM----KYGT--------------D---DACPAMKYFLCSYINTV

----V---

--ECD----SWNKTET

CAKHSEYASKCKVSLG--

>Dple-OBP25_DPOGS202589_EHJ67714_AB_DpOBP42_AB_DpOBP43_DPGLEAN20949_

--------------MLNLLRIALFIVAVNADLLNHERTKGATLKPISVCCDIPELGDPKP
LAECSNMKLQGPCSDVQCVFEKSGFLLDKQT-----------------------------
-------------------------------------LNK--EGYRNHLMKWLEGHKEWK
DGIEKAISDCVDVDLRQYLDYPCKAYDVFTCTG---IAMLKVVLSDVPPHCR-GPPPGGA
NPSDCCKLP-----------------KVFTEE-------DF----KECG-IEKPTKE---
-----------RRVPECSKQICLLKR--YELM----------------------------
-KD---D--T-N---------VDKDAVAAFLDKYSEG----DEDTQSA--VEA-TKQKC-
-----LNN------DLP----NIP-------------------EICEPSKIVFCVAGSML
----M---

--NCP----KWDESED
CQKLKDHFENCNKYFPKK--

>HmelGOBP1_HMEL004625_AB_HmOBP1_

---------------------------M--------------------------------
--------------------------------------IS--LLT--LS---------LA
CGVLTG-L------------------------D---ATME--------VMKD-VTLGFGE
ALQSCREES-----------------QLTE-D-------KM----E-EF-FHFWRDD--F
--KFE------DR-EVGCAMKCMSSH--FNLL----------------------------
-TD---S--H-R---------MHHENTDKFIKSFPNG----E-VLSKR--MIS-IIHTC-
-----EQQ------FDA----LE-D--------------H---CWRILRIA-ECFKVACK
K---E---

--GIA----PTMELLM
AEFIMEADP---

-S--

>HmelGOBP2_HMEL022500_mod_AB_HmOBP2_

---------------------------MAVR-----------------------------

--------------------------------------CY--VVVFLAL---------LA

LATLPR-V------------------------R---STAE--------VMSH-VTAHFGK

SLEECREES-----------------GLSS-D-------IL----D-EF-QHFWSEE--F

--QVV------HR-ELGCAIICMSNK--FLLM----------------------------

-HD---D--A-R---------MHHVNMHDYIKSFPQG----E-LLSEK--MVN-LIHNC-

-----EKQ------FDD----IE-D--------------E---CSRVVKVA-ACFKESAK

E---E---

--GIA----PEVAMIE

AVLEKY--

>HmelPBP_C_HMEL016107_AB_HmOBP6_

----------------MF--KR--------------------------------------
---------------------------------------L--T----VV---------VL
VFVGLN-K------------------------V---TSNE--------TLRG-ITASFLK
VLEECRQEL-----------------NIGD-N-------VL----A-DM-YYFWKLD--R
--TLI------HR-DTGCAIVCMSKK--LNLL----------------------------
-DT---S--G-K---------LHHGNAEEFALQHGAA----D-DMAKK--LVT-TVHEC-
-----EQK------HEL----EE-D--------------Q---CLRALEIA-KCFRGAMH
E---I---

--NWA----PKVDVAI
SEILTEV---

>HmelPBP_D_HMEL016108_AB_HmOBP3_

----------------MA-VQKW---QL--------------------------------
---------------------------------------I--L----VM---------CV
LMQACK-V------------------------S---PSQE--------IMHK-LTKGFAT
AFEQCKQEL-----------------NLGD-N-------IM----Q-DF-LNYWREE--Y
--ELL------NR-DTGCAIMCMAQK--HDLL----------------------------
-TE---D----G---------IHHEKVHGFTKSHGAD----D-ELAKQ--LVT-MIHEC-
-----EKS------NAG----VS-D-------------E---CMKTLEVA-KCFRTKIH
E---L---

--KWA----PDMETIL
EEIMTD--

------------------------------I-----------------------------

>Hmel-OBP3_HMEL005621_AB_HmOBP35_

---------MVGLKALHDIQINKDTIITRNM-----------------------------
-------------------------------------NLK--TDN--KI---------SH
NHDPDWSY------------------------S---SFPK--------EVKS-HVEQFKR
NMSECLKEV-----------------QLND-K-------RQ----VRRL-SPKK------
---ES------PV-HGECLIACVLKR--NGVI----------------------------
-EN------G-K---------IYKDNLLSLVRKFYGK----DEKLMKK--LEK-NVDRC-
-----IEA------SVK----NKDD------------------CTVASYLN-ECTNDLMA
N---N---
--KHK----IIVNY--

>Hmel-OBP6_HMEL007173_sameasHMEL022532_AB_HmOBP32_

---------------MENISYKG---KLFLL-----------------------------
-------------------------------------LFI--AFA--HVTLVDSYSH-KF
FSQNLDTE----------------------------PSLS--------IQYA-RDKKSDM
ITNECLTEM-----------------YPKN---------------IYRY-PLHI------
-----------DRNDVPCIIHCVLKK--FGIM----------------------------
-SN---D--G-V---------INTRNYYRRVRAIHRY------DPRVL--ISD-VGETC-
-----AQN------ING-MNLDHDV------------------CKKAKVFN-DCTQLYAI
SYRES---

--DD------------

>Hmel-OBP7_HMEL007174_AB_HmOBP33_

---MYA-HEKMSDI
VAEQCLNEM-----------------YPKG---------------RRIQ----F------
-----------EESDEPCIIYCVLKK--LGIM----------------------------
-NS---N--G-Q---------INVDM----------------------------------

>Hmel-OBP8_HMEL007175_AB_HmOBP34_

-----------------------MLIHAVLA-----------------------------

-------------------------------------LIG--YTL--AA---------QH

RSFSGTMV------------------------D---LSDP--------KVQG-HLDALVR

MAQSCVIKV-----------------RASP-K-------DV----RAYF-TNSP------

---PI------TR-SGQCFAACMLEQ--SDVI----------------------------

-NH------G-K---------VNRELLVHLAGLVNGK----NSRVVRK--LYG-ISRLC-

-----LDS------IEG----MSDR------------------CQLASTYN-DCLNENMI

E---F---

--AFP----LDIAEEA

VRKMPFHLIQPK--

>Hmel-OBP12_HMEL008322_AB_HmOBP38_

--------------------MKIIIIITLAL-----------------------------

-------------------------------------LTF--SVA--QCGSSVGNRMSTD

DITTTTMA------------------------T---TMED--------DDAR-SNFDVMS

VMVDCNDTF-----------------RVEM-S-------YL----E-SL-NKSGSFP---

---DE------TDKTPKCFVRCVLEK--SDIV----------------------------

-SG---D--S-Q---------FNVTRTAEVFSQIRDT-------SQND--IIK-MATAC-

-----SDR------PEK-------C-------------K---CERSYQYL-KCLLETTI

E---I---

--NEM----KSSK---

>Hmel-OBP13_HMEL008689_ABPX_AB_HmOBP20_

---------------------------MQGF-----------------------------

-------------------------------------IVH--CSI---------------

--VAILVV----------------------------GASA--------LEGE-MAELAKM

LRDSCIEET-----------------GADI-A-------LI----DKVN-DGADL-----

---MP------DP-KLKCYIKCVMET--AGMM----------------------------

-SQ------G-A---------VDVEAVVAVLPP----------ELQRH---AD-KLRAC-

-----GTK------AGA------ND------------------CDTAFLTQ-ACWQGGCK

E---D---

--YILI----------

>Hmel-OBP14_HMEL010224_AB_HmOBP7_

---------------------------MKTL-----------------------------

-------------------------------------KLL--ILL---------------

--FVFFFG------------------------D---SQGM--------TDDE-MREEFMR

ITMICSKDY-----------------KVDM-K-------DL----LSLQ-QLNI------

---PT------KK-DVKCLLACAYKK--TGSM----------------------------

-NK---E--G-L---------YDIEASYRIAEMTKNG----DPKRLEN--AKK-LVDIC-

-----AKV------NDE----TVSD--------------GEAGCDRAGLIF-KCVVENAP

K---V---

--KNN-----------

>Hmel-OBP15_HMEL010226_AB_HmOBP9_

--------------------------------A---ITDE--------QKAM-IHSHFEM

LGKECIKDN-----------------LISA-D-------DI----KNLR-AKKI------

---PS------GE-NAPCFLACMFKK--LGIM----------------------------

-DD---A--G-L---------LQKETVLDLARKVFND-----EDEIKL--IGD-YLHSC-

-----SHI------NTE----SVGD--------------GDKGCDRSMMAY-KCMIENAS

Q---V---

--LFT----FN-----

>Hmel-OBP16_HMEL010227_AB_HmOBP12_

---------------------------MFLI-----------------------------

-------------------------------------VLI--F-----------------

-------Q------------------------G---YSDE--------ERTK-IYATMLP

HILQCSTEY-----------------GITE-D-------EL----KASK-ENEK------

-----------FGSINPCFMGCIFKK--IHVI----------------------------

-NK---E--G-I---------FNVEKAEKLSENFLVH-----DEDKKK--ASA-VIKAC-

-----ATI------NDE----DVSD--------------GEKGCDRAKLLF-ECLLPFRQ

Q---V---

--ILK----LIMCIEK

ATKT--

>Hmel-OBP17_HMEL010228_AB_HmOBP11_

---------------------------MTSL-----------------------------

-------------------------------------QLV--FLV--FG---------VV

AVSLGSVS------------------------A---FSDE--------ERNK-IYAGMLP

LVLECSKDY-----------------GLTE-D-------DL----KAAK-ESGS------

-----------IGSINPCLMACVFKK--INVI----------------------------

-ND---K--G-L---------FDVDKAGELSQKFLTE-----TDDQQK--ATE-IIKTC-

-----ASV------NEK----DVSD--------------AEKGCDRSKLLF-DCLLPFKG

Q---V---

--KYN----IVIILII

YSSR--

>Hmel-OBP18_HMEL010229_AB_HmOBP14_

---------------------------------------M--------TRQQ-LKNSSKL

LKKNCMAKN-----------------DVTE-D-------LV----GDIE-KGKF------

---IE------DQ-KVMCYIACIYQM--SQLV-----------------------------

-KN------N-K---------LNVEASIKQVDMMFPP------EMKDA--TKA-SIENC-

-----KDV------SKK----YKDL------------------CEASFWTA-KCLYEDNP

K---N---

--FFFA----------

>Hmel-OBP19_HMEL010230_AB_HmOBP16_

---------------------------------------M--------TRPQ-LKKTLTI
MKNQCMPKH-----------------RVTN-E-------KV----GQIE-QGVF------
---VE------DH-DVMCYIACVYKT--AQVV---------------------------
-KN------K-R---------LDKDLVSKQIDILYPA------EIREA--VKL-STVKC-
-----IPV------QYN----YEDE------------------CEGIFYSV-KCLYEDNP
A---N---

--FIFP----------

>Hmel-OBP20_HMEL010231_AB_HmOBP17_

---------------------------------------M--------TRQQ-LKNSGKL
MKKSCMPKN-----------------DVTE-D-------QI----GQIE-QGKF------
---LE------ER-NVMCYIACVYTM--TQVV----------------------------
-KN------N-K---------LNYEATIKQVDLMFPP------DMKDA--VKA-AVEHC-
-----KDI------SKK----YKDI------------------CEVSYWTA-KCMYDFDP
N---N---

--FVFP----------

>Hmel-OBP21_HMEL010232_AB_HmOBP18_

---------------------------------------M--------TMKQ-IKNTGKM
MRKTCQPKN-----------------NVAD-E-------KI----DPLN-KGEF------
---IE------EK-EVMCYVACIMKM--ANTI----------------------------
-KN------N-K---------LNYEAAIKQADMLFPD------EIKEP--AKE-AITAC-
-----RKV------VDD----YKDL------------------CESSFYTT-KCIYNYNP
S---I---

--FFFP----------

>Hmel-OBP22_HMEL012883_AB_HmOBP36_

------------MGNSIKKDEEQHAMFRIKF-----------------------------
-------------------------------------YYI--VIF--LIIFTAFILVMAF
TPLTKDEQ------------------------I---DKFN--------KMNE-GVEPFRR
NLTECARQV-----------------KASM-V-------DV----ENFL-KRIP------
---QT------SL-QGKCFVACILKR--NSII----------------------------
-KN------N-K---------ILKENLLEANKAVYGE----DSEVLSR--LKL-AINEC-
-----SDV------VAN----IFEI------------------CEFSSVFN-DCMHMKME
H---I---

---LDK----VIMERRM
EALGQMTGDPDVWTDEEDELLKLVKDEL--------------------------------

>Hmel-OBP23_HMEL013351_AB_HmOBP21_

---------------------------MQSA-----------------------------

-------------------------------------ALLAAVFL------------A-L

ITFGFGQK----------------------------EKPE--------FSEE-IKEIIQH

VHNECVAKT-----------------EVTE-E-------DI----ANCE-NGIF------

---KE------DI-KLKCYMFCLLEE--GSLV----------------------------

-DE---N--D-N---------VDYDMMISLIPE----------QYTDR--VSK-MITAC-

-----KHL------DTP----DKNK------------------CQRAFDVH-KCSYDSDP

K---V---

--NF------------

>Hmel-OBP36_HMEL006421_sameasHMEL017002_AB_HmOBP37_

-----------------MSSVVKPHLRLESP-----------------------------
-------------------------------------FIQINCYPLLTIRRPRNWIRQLA
VNNWQDIC------------------------NERVLENP--------IAAM-IHKSLTA
VAHSCMEKI-----------------NGTE-E-------DL----E-YL-RKDP------
---PF------PE-KSACIIVCLLKK--IGVV----------------------------
-KD------D-N---------FSKTGFVTAVTPLVFH----NRKKLEH--MKN-VSEKC-
-----DKE------ITT----PEDT------------------CQLGNSIT-ACIFKYAP
E---L---

--HYK----T------

>Hmel-OBP39_HMEL022521_AB_HmOBP19_

--------------------------------------AR--------SETE-IKKWFIQ
QAVECSKEH-----------------PVTG-E-------EL----QQMKTKHKI------
---PE------SM-SAKCLVACIFKR--IEWI----------------------------
-DE---K--G-M---------FVKEKAYKTSEKDYLN----DQVKLDK--AKE-LYESC-
-----SKV------NSE----TVTD--------------GEKGCERSNLLA-ICLTESAA
Q---V---

--RLV----KS-----

>Hmel-OBP43_HMEL022602_AB_HmOBP8_

--KYAE

IIMTCAKDY-----------------PITP-E-------DI----EQLK-NSKL------

---PD---------NAKCLFACAYKT--SGMM----------------------------

-DD---Q--G-K---------LSVEGVNKLAHTFLAD----DPERLKK--AEQ-FTDAC-

-----KSV------NEE----EVSD--------------GDKGCERAALIF-KCSIEKAP

QVINN---

--KIE----M------

>Hmel-OBP2_HMEL003711_AB_HmOBP13_

---------------------------MSIL-----------------------------

-------------------------------------LCF--LFL---------------

SIALIDGK------------------------T---VNIP--------VDPD-ITSQLVQ

ISADCIASN-----------------GLTE-E-------VL----KQVM-EWKL------

---EN------NE-PTKKLLFCFGTK--LNTT----------------------------

-DK---N--G-H---------VILNEALKLAVS----------KKRPV--FGD-AIKRC-

-----NDQ------EGS------DK------------------YDTLFKII-ICMRDQEN

I---F---

--LRF-----------

>Hmel-OBP4_HMEL006095_AB_HmOBP29_

---------------------------MAVM-----------------------------

-------------------------------------MVM--SAV---VS-------AMV

VHQVITQY------------------------IYFKQIKN--------LGKD-EKAMFRA

HSEACLPQS-----------------QVDP-K-------LL----DNLL-QGEL------

---VE------DA-ALKRHVYCVLLK--CKVI----------------------------

-SK---D--G-K---------LQKTAVLGKMAHR---------ADSKN--ITK-VLERC-

-----ADQ------PGD------TP------------------EELAWNLF-RCGYNKKA

V---L---

--FDYMPAQVNDGADN

DG--

>Hmel-OBP9_HMEL007611_AB_HmOBP30_

---------------------------MLVF-----------------------------
-------------------------------------ILF--SAH---------------
----NMEN----------------------------KCDE--------DVSP-TCVPSVD
PIEECIEKL-----------------HVNR-A-------LV----EKLK-SGNSK-----
---LF------DK-NLKRWLLCFFEK--TCVM----------------------------
-TP---D--G-V---------LRQDVVLKDIPD----------QDKSK--IEK-ITSIC-
-----LYQ------KLH------FA------------------VDTAWNYL-NCFREKDP
K---Y---

--SVI----ANKI---

>Hmel-OBP10_HMEL007612_

---------------------------MKTF-----------------------------
-------------------------------------IVL--AIC---FV----------
------------------------------------AAQA--------LTDE-QKDKLKK
HKSECLAET-----------------KVDE-K-------LV----DKLK-TGDFE-----
---IE------NE-PLKNYTLCMLVK--SGLM----------------------------
-SM---D--G-E---------FKKDVALAKVPNA---------ADKQL--VGQ-LIDLC-
-----LAN------KGA------AP------------------EETAWNYS-KCYHQKDA
K---H---

--SIF----Q------

>Hmel-OBP11_HMEL007613_AB_HmOBP31_

---------------------------MKTF-----------------------------
-------------------------------------IVL--AIC---FV----------
------------------------------------AAQA--------LSDE-QKDKLKK
HRSECLAET-----------------KADE-Q-------LV----NKLK-TGDFK-----
---TE------NE-PLKKYSLCMLIK--SELM---------------------------
-TK---D--G-K---------FKKDVALAKVPNA---------ADKPA--VEK-LIDLC-
-----LAN------KGN------TP------------------HQTAWNYS-KCYHEKDP
K---H---

--SIF----Q------

>Hmel-OBP24_HMEL015914_AB_HmOBP25_

---------------------------MRAV-----------------------------
-------------------------------------WCL--LAV---------------
SLAVVSGK------------------------VLD-TLVI--------VPPE-RVPGIVQ
ASSKCIEEM-----------------NLDK-D-------TM----QKFF-SWQL------
---GD------SE-STRKYMYCLGVK--SGYI----------------------------
-AD---D--G-S---------MVKKEVLGLAG-----------SHGGN--IDG-VIDEC-
-----NNL------KYS------DK------------------YEAVFKIV-MCFHEKSK
L---E---

--FKV-----------

>Hmel-OBP25_HMEL015916_AB_HmOBP24_

-------------------------------------TII--------LPPA-RAFDIVQ
ASSKCIEEL-----------------NLEK-N-------IL----QKYL-AWEL------
---SD------CE-STRKYMYCLGTQ--SGYI----------------------------
-AN---D--G-S---------IVKNKAFEVVG-----------SHRNR--VNG-IIDEC-
-----HEF------KHN------DK------------------YEAVYRNV-MCFNKKSR
L---H---

--FKV-----------

>Hmel-OBP26_HMEL015917_AB_HmOBP22_

-------------------------------------IVI--------LPPA-RVPGIAQ
ASSKCIEEL-----------------NLEK-D-------TL----QKFL-AWEL------
---SD------SE-STRKYVYCLGTE--SGYM----------------------------
-AK---D--G-S---------IIKNEVLEVVG-----------NHRNR--VDG-VIDEC-
-----NER------KYN------DK------------------YEAVFHNV-ICFNEKSG
L---H---

--FKF-----------

>Hmel-OBP27_HMEL015918_AB_HmOBP23_

----------------------MDTGKMRAV-----------------------------

-------------------------------------WCL--FLV---------------

TLATVYAG------------------------NVKVEYVD--------IPKD-YIPAVEK

ASFECIKKL-----------------KLEEEQI------SL----QGFL-NWEL------

---SE------SD-NTKKYIFCLANG--SGFF----------------------------

-AD---D--G-S---------ILKDKVLAIMG-----------KYRDR--VDK-VIDEC-

-----SKV------KYD------NK------------------YEEVYRME-VCFRDLSG

L---Y---

--FRM-----------

>Hmel-OBP28_HMEL015920_AB_HmOBP27_

---------------------------MNTL-----------------------------

-------------------------------------WFF--LFL---------------

SIALVKGK----------------------------ALFH--------VPPE-YAGEILK

AAADCIDST-----------------GAGV-D-------AV----QKVI-SANL------

---EN------TE-PFKKFLYCFSSK--SGYV----------------------------

-DS---D--G-H---------FIVDQMTKLIGNH---------KDKAK--FID-NLNLC-

-----NKS------EGG------NT------------------IDTIYQIA-VCFKDNSP

I--Y--

--FTV-----------

>Hmel-OBP29_HMEL015921_

---------------------------MKTF-----------------------------

-------------------------------------WCF--LFL---------------

SIALVSGR----------------------------AIVN--------VSQE-FTGDILK

TAVDCTDSS-----------------GAGV-D-------AL----QKMA-SANF------

---ED------TE-PFKKFLYCFASN--SGYV----------------------------

-DS---D--G-H---------FIMDKMTKLIGNH---------KDKAK--YVD-AINLC-

-----NKR------KGG------RT------------------IDTIYELA-NCFKDHSP

I---Y---

--FTL-----------

>Hmel-OBP30_HMEL015922_

-------------------------------------MFD--------IPKE-FVPDIIK

ASAECADKL-----------------GLDTLN-------IL----SKFF-SGEL------

---KD------SE-SVRQYMYCLGTT--SGYV----------------------------

-ND---D--G-R---------LNKDRLGKVVG-----------EHKSK--VES-VVDEC-

-----NKA------KAS------DK------------------YETVYKAV-VCFRENSG

L---Q---

--FKI-----------

>Hmel-OBP31_HMEL015923_

---------------------------MLMR-----------------------------

-------------------------------------KNP--ICI-------------YI

SIIYLGVQ------------------------YVLVEYIK--------VPKE-FIDDAIN

GSGQCAEEL-----------------GLPG-D-------TL----NKLL-SNNF------

---ED------SQ-AMRKYIYCLGIA--LDVG----------------------------

-DG---T--G-----------SLKHSLSKYASND---------RRKAE--ITK-TIDEC-
-----NKE------KAS------DK------------------YEKAYKVS-TCYLNTSS
V---Q---

--FKV-----------

>Hmel-OBP32_HMEL015924_AB_HmOBP10_

------------------------------------TYFK--------IPKE-FIEDAIK
GSVHCTEEL-----------------GLPV-D-------TL----NMFL-SDNF------
---ED------SP-TMRKYIYCLGLA--VDVS----------------------------
-EE---D--G-----------SLKHSLSKYANND---------KRKAE--VIQ-RVDEC-

-----NKQ------EAN------DK------------------YEKVYKVS-TCYLNTSS

V---Q---

--FKV-----------

>Hmel-OBP33_HMEL015925_AB_HmOBP23_HMEL015918_

------------------------------------AYIK--------VGKE-FTEDAIK

GSVHCTDEL-----------------KLPV-E-------TL----QTFL-TSKY------

---ED------SL-PMRKYIYCLGIM--LDVG----------------------------

-DE---N--G-----------NLKHSLSKYAGNN---------KRKAE--ILE-TIDEC-

-----NKL------EAS------DK------------------YEKALKVS-TCYLNKSS

L---L---

--FEIKKD--------

>Hmel-OBP34_HMEL015927_AB_HmOBP15_

------------------------------------AYIK--------VGKE-FTEDAIK
GSVHCTDEL-----------------GLPV-D-------TL----QTFL-TSKY------
---ED------SP-LMRKYIYCLGIM--LDVG----------------------------
-EE---D--G-----------SLKHSLSKYAGND---------KRKVE--VLK-TIDEC-
-----NKL------EAS------DK------------------YEKAIKVS-TCYLNKSP
L---L---

--FEIKKD--------

>Hmel-OBP35_HMEL015928_AB_HmOBP5_

---------------------------MKVL-----------------------------
-------------------------------------IIC--VGL---------------
---FALIE------------------------A---HNIH--------LSHG-QKEKVKE
YAAECMKES-----------------GAKP-E-------VL----ADAK-KGHL------
---VD------DE-GLKKFILCFFQK--TGVL----------------------------
-SS---D--A-K---------LNTDVALSKLPAG---------IDKVT--AAK-VLNDC-
-----KNK------KGA------TH------------------ADTAFEIF-KCYYTHTK
Q---H---

--ILF----EK-----

>Hmel-OBP37_HMEL018118_

-------------------------------------IIA--------MPPA-RISRIVM
ASSKCIEEL-----------------NLEK-D-------TM----QKFF-AWQL------
---SD------SE-STRKYTYCLGTG--SGYI----------------------------
-DS---D--G-S---------IIKNEVLDIVG-----------SHRDR--VNA-IIDEC-
-----NKS------KYN------DK------------------YETAFRSV-MCFNEKSG
L---Q---

--FKV-----------

>Hmel-OBP41_HMEL022561_

----YLKES-----------------AAKS-K-------IL----ANTN-KRHL------
---ID-----------------------YEEL----------------------------
-SS---D--D-K---------LNTDIALLKLPA------------KVT--AAK-VFNDC-
-----ENK------QGA------TR------------------AYTAFEIC-NVTTLLHN
QLLYL---
--KNK-----------

>Hmel-OBP42_HMEL022573_

---YAGVILK
AAADCIEST-----------------GVGA-D-------AV----QKVI-AANL------
---EN------TE-PFKKFLYCFSSK--SGYV----------------------------
-DG---D--G-H---------LIVDQMIKLVGNH---------KDKAK--FID-VINLC-
-----NKS------KGG------NT------------------LDTMFQTT-VCFKQNSP
I---Y---

--LTL-----------

>Hmel-OBP1_HMEL003176_AB_HmOBP41_

---------------------------MSVR-----------------------------
-------------------------------------VLF--IFI--IV---------TA
CQANIQV---SPPV-TCGYLPR
AIHECIGSP-----------------HIVK-P-------EI----SAQC-S---------
-----------KSISECERMTCVFQK--SGWM----------------------------
-SG------N-A---------VDKDKVKSYFDQFSTD----NPQWALA--VNH-VKAAC-
-----LNM------DLP----SQGV--------------Y---LNCPAYDILTCVFSGFI
----K---

--NAQPD--QWSSSES
CSYPRQFASACPYCPSDCFAAQVPIGSCNACLALPRSP----------------------

>Hmel-OBP5_HMEL006666_AB_HmOBP43_

---------------------------MLRI-----------------------------
-------------------------------------PLV--AIF---------------
---ILK
PISACCDIP-----------------EMGDVG-------PL----SECS-NP--------
-----------RLPGPCNEVQCVFEK--SGFL----------------------------
-ID---K--Q-T---------LNKEAYRAHLRQWVEG----HKGWEDA--VEK-AIKDC-
-----VDR------DLR----QYLD-----------------NPCKAYDVFTCTGIAML
----K---

--VRK-----------

>Hmel-OBP38_HMEL022514_AB_HmOBP40_

-----------------------------EF-----------------------------

-------------------------------------IQP--KWR-------------TD

CGFKLKWD--DINR-LSIFNES

DVTTTTTEA-----------------TSLRNK-------DV----K--------------

-----------IVPLPCDKQNCIFSK--LNIT----------------------------

-ND------G-V---------IDKDAFSRLLDTMTHR----HTQWTSA--KAK-VVTQC-

-----LNK------PLL----GYE-------------------EDCEINRILACTYDILT

----E---

--VCY-----------

>Hmel-OBP40_HMEL022553_AB_HmOBP42_

---------------------------MHQT-----------------------------

-------------------------------------VVF--CLL--IF---------QV

VTAEPPPP--QCRG-PPAGLQK

NPKECCKFP-----------------KVFKEE-------DF----KECG-IEKPSEE--D

GSFHH------RGPPDCDKQICLLQI--NNLM----------------------------

-KD---D--T-T---------IDKDAVTAFMQKWGDA----NGDFKDA--VDV-AIDRC-

-----VKG------DLP----GPP-------------------ELCEATKMIFCIGSTMF

----M---

--NCP----KWEDTDD

CKKVKEHIEECKPYFN--

>Hmel-OBP45_HMEL009897_AB_HmOBP39_

----------------------MVQRISALL-----------------------------
-------------------------------------WCL--YIF--SISLSDSAISAET
ETRCRNPP------------------------T---APQK--------IERV-ISLCQEE
IKLSILREA----------------LDVIKEE-------HT----MPAQ-RRRNKRE--V
PFTHD------EKRIAGCLLQCVYRK--VKAV----------------------------
-DG---Y--G-F---------PTLEGLVGLYSDGVNE-----RGYFMA--VLE-ASREC-
LMRNHDKF------SRT----VPMD--------------NGRNCDISFDIF-ECISDRIG
E---Y---

--CGN--------SGL

>Bany-OBP_CN1_GW3I2KR01B64MG_35Frame3

-------------------------DRRTGV-----------------------------
-------------------------------------RMF--RNV--FL---------ML
CSVSVG-L------------------------Y---ADVE--------VMKD-VTLGFGE
ALQHCREES-----------------QLSE-D-------KM----E-EF-FHFWRDD--F
--KFE------DR-ELGCAIKCMSAH--YDLL----------------------------
-TD---S--H-R---------MHHENTDRFIKSFPNG----E-VLSQQ--MVQ-LIHEC-
-----EQQ------HDA----EE-D--------------H---CWRILRVA-ECFKGACQ
R---H---

--GIA----PTMEMLM
AEFIMEAESRTSTALVD--FTSLRNIIYTSILIYKAEEFVCLFERANLINYWSDLKNYFS
ARPIYRGRLAIYYPSIPTGPRTTRVKPRGVSYYHKLTNGLDTKAGRIHPFFVFGILMRNF
DLEFRTLRFSSSGMHIVSSARIRYVREEFVQFENKNVFAGGSPPYRQRRTTKRFRVPYDV
VWIFIPLLTSPAFILDCIT---
-----IRDCSQGL-------------------------TC--------------------
---KEK
RK--

>Bany-OBP_CN2_GZKG99Z02D6PVR_35Frame1

-----------------CAIMCMAS---IRPI----------------------------

-AD---D--M-S---------LHHQNAHEFAKTHGAD----D-DMAKQ--LVN-MLHDC-

-----EKV------HDS----IV-D--------------N---CSKTLEIS-KCFRTKIH

E---L---

--KWA----PSMEEIL

EEVMTEVMIENWLEITRLNDVFLYSRFANRQMVLGNHNIVRGHSRFIVFKITV--LNTKG

RRKQKYNI----------------------------DSLHNLVVNISNLNTGQSSGNLLQ

-----------KKTRKT-TSKALITKWLDYITRNNTNDIILYL-----------------

--------------------VEYNP----------YIISHNNFIEVV-------------

---------ASYFR--

>Bany-OBP_CN3_GE679317_35Frame2

------SSGQRRVAITAGDRYNNMATRARWR-----------------------------

-------------------------------------FLA--V----VV---------FV

VTHGHR-A------------------------I---ASQE--------IMKK-LTTGFSK

ALEECKQEM-----------------GFGE-H-------IM----K-DF-YNYWKED--Y

--ELL------NR-ETGCAIMCMASK--FDLI----------------------------

-AD---D--M-S---------LHHQNAHEFAKTHGAD----D-DMAKQ--LVN-MLHDC-

-----EKV------HDS----IV-D--------------N---CSKTLEIS-KCFRTKIH

E---L---

--KWA----PSMEEIL

EEVMTEVMIENWLEITRLNDVFLYSRFAN-QMVLGNHNIARGHSRFSFILSFLRHLYLIQ

KADASRNIILLRHLYLIQKADASRNIILILSFFFLLNLICYPNVLVDSLTFGSQLINRYR

ITLVRVVVTCCKKTRKTLTSKALTTKLILYCTYLVEYNPYMHLRITQLYRGGRFVFQFVS

TVMLIWFRDGSVKLLESYKTKLYFP----------YYVPHYGLTNIISLKICLCVLDILF

IFNELEIIVKHIAIELQRLLEKTPLITTAFRSTAAMSLHFTLASCLTMLSETTYLLCLWR

LRSFGRENGAAYFNSMRGLNCLSAAAKTVGLRAPAVYDNIFCFGQHLYVF--------LV

KVFDTRSNSMIIC-----LLNVFHCNLMIACLLNVFHCNSMITCTLSLQLPNNTTNCLMF

YGFKYLTLLPATCQIAFRLFNPHVLCVFVVWNVNQTSIWCTDKKKTLLIPLSLPLSYRNH

STVGKYLDTILPLQFHHFKRNIILYL

>Bany-OBP_CN4_GE686547_53Frame2

--------------QTNNRTNEDYGRTTYLR-----------------------------

-------------------------------------LVG--R----YF---------TF

VNNMGT-S------------------------Y---SFPR--------NHEE-TNHRVSK

ALEECKQEM-----------------GFGE-H-------IM----K-DF-YNYWKED--Y

--ELL------NR-ETGCAIMCMASK--FDLI----------------------------

-AD---D--M-S---------LHHQNAHEFAKTHGAD----D-DMAKQ--LVN-MLHDC-

-----EKV------HDS----IV-D--------------N---CSKTLEIS-KCFRTKIH

E---L---

--KWA----PSMEEIL

EEVMTEVMIENWLEITRLNDVFLYSRFPN----ISSLDVGTKSYCRSIFIYRF--DNCSY

KRPTQAEIY---------------------------FLVTFGSQLINRYRIILGRVVVTC

-----------CKKHAKPLLKPLLNDLYYIVPSIPLHLTFTYH----------------K

LIISRWSLRISVCLLNGYANMVMRW----------VSIMIRKLQNIIFSILCSSLWPHYY

PVKDLFMCFRYSFHIVEDYRKAYLSHRVTTSTREDTTHHYYLPINCG---------DIAT

LHISFVLAYYVERNVIYGAYEVLVAKTALHILIVEDLTNVLVLQKILKSACVELSVCDML

FVLDSISSTYFLVTQFKLVLSRTYLILVVTQFKFVFSRTYFIVTQFKLVLYNNPSS----

----YQITRRPACFMDFRNIRFSQQLVRHLGYLILMYCVYFKWCGMLTNKQVFGIQKKKI

ITKKK---------------------

>Bany-OBP_CN5_GE680338_53Frame3

--XLRPGV

KRHCGHYGRGSPSLSNM----------IVPN-----------------------------

-------------------------------------YCG--GFL-------------VL

VLYFIHFP------------------------S---NYAM--------TRQQ-LKNSGKL

MKKSCMPKN-----------------EVTE-E-------QI----GQIE-QGKF------

---IE------ER-NVMCYVACVYSM--TQVV----------------------------

-KN------N-K---------LNHEAIIKQVDMMFPP------DMRDA--VKV-AVENC-

-----KDI------GKK----YKDI------------------CEASYWTA-KCMYDYDP

K---N---

--FVFPNCNNTYFKKE

KKEKK---

>Bany-OBP_CN6_GS8R7LM03EULY6_53Frame1

---XXXX

LRPGIHFFSERFRVCKM----------DRID----------------------------

------------------------------------FSL--LII------------VT

VL----AS------------------------G---IDSM--------TRQQ-LKNSSKM

LKKSCMGKN-----------------DVTE-D-------LV----GDIE-KGKF------

---IE------ER-NVMCYIACIYQM--SQII---------------------------

-KN------N-K---------LNYEASVKQVDMMFPP------EMKDA--MKA-SIEKC-

-----KDI------SKK----YKDI------------------CEASYWTA-KCIYEDNP

K---N--

---FIFAFDFYNKRYLS

IKNLLKNTKKKKE--

>Bany-OBP_CN7_GE682000_35Frame2

----------------YQRRVAITAGEIDTENTE--------------------------

-------------------------------------MFK--VVPIVVF---------LF

ISISIN-E------------------------I---TTNE--------TMKN-ITSGFLK

VLDDCKHEL-----------------NLSE-N-------VL----A-DM-YHFWKAD--H

--ALL------HR-DTGCAIVCMSKK--LNLL----------------------------

-DT---S--G-K---------LHHGNAQEFAVNHGAA----S-DMASK--LVS-VVHEC-

-----EQK------HDA----EE-D--------------P---CLRALEIA-KCFREVMH

E---M---

--NWA----PRMDIII

SEVLTEIINEETKRIINKEVKRMKHANKQTSYVTYNIFLSRLFFGIVNVYCLFIFYFLNP

LFNFFINNFYFCNFMFIVVLSYFYPGKTNWDTVMIPGSISYKLNYSFLLYIYFGYLLQLH

MTSMYCMLQRPSFLEFFRILNKNQICVHIYIK----------------------------

>Bany-OBP_CN8_GW3I2KR01AEGGI_53Frame1

---FCK

VLDDVSMSL-----------------NAKT-Y---------------PIXTTSGSGS---

--RFA------PP-RHRVRHRMHEQK--AEPA--------------------------

-RY---S--G-K---------LHHGNAQEFAVNHGAA----S-DMASK--LVS-VVHEC-

-----EQK------HDA----EE-D-------------P---CLRALEIA-KCFREVMH

E---M---

---NWA----PRMDIII

SEVLTEIINETKRIINKEVNVSMQISKEVNIRLLTKIFFVGYFLELVILCLLLYLVNSDT

PGKRIG------------------------ILLFPGSISYKLNYSFLLYIYFGYLLQLH

MTSMYCMLQRPSFLEFFRILNKKSNLRTY-----------------------------

>Bany-OBP_CN9_GE686922_35Frame1

-AASDMASKLVSVVHECEQKHDAGEDPCLRA---------------------------

-------------------------------------LEI--AKC--FR---------EV

MHEMNWAP------------------------R---MDII--------ISE--VLTEIIK

LKNRNNKRS----------------KTYEA-CK------AN----K-LI-LGYLLKY--F

FKVIF------WNSRLLFIYILFFES--FVRF----------------------------

-PS---Y--M-S---------TNLLNVLSILKVFFLI----L-IKDGQ--LMS-TNQGA-

-----RAI------ADV----DQ-S-------------R---PTNWGKRS-VGGARRLR

S---S---

--RAS----FEILELL

KSVIKLRKVNKICYCAKRKFYFHTGLNLTPLFNFFINNFYFCYFMFIVVLSYFYPRKTNW

D---

---------------------------TVVIPCLFHINNITVSYCIFTLITYCSYIRVCI

VCCNGRASWNRILN--

>Bany-OBP_CN10_GW3I2KR01E2YNH_53Frame3

-----NDSF-----------------RVEM-S-------YL----A-SF-NKSGSFP---
---DE------TDKTPKCFMRCVLEK--SGVA----------------------------
-SP---A--S-Q---------FNVKRTAEIFPQIRDI-------AEED--IVK-IATEC-
-----TDR------PET-------C--------------K---CERSYQYL-KCLMETVI
E---I---

--YDV----KNNFPIF
GFPNKHTYPLL---------LLFFHGKIYKTLLKIKK--ICYYNRFIINFFILMCTYSNV
KSIKRK--

>Bany-OBP_CN11_GE680903_35Frame3

-----YDSL-----------------RRGG-N-------VT----AGSF-NKSGSFP---

---DE------TDKTPKCFMRCVLEK--SGVA----------------------------

-SP---A--S-Q---------FNVKRTAEIFPQIRDI-------AEED--IVK-IATEC-

-----TDR------PET-------C--------------K---CERSYQYL-KCLMETVI

E---I---

--YDV----KIKPFPS

LVVPICILITASAFVFFPIYLFVLFNFKQKIYKTLLKFIKKNVTIIALFSIFDLYCNVHI

KAMFKNIKKKNFSINKYTGRYFKNYLFYVMSFLYNLYRLGNKHCNSLKSPLINKSLLNH-

>Bany-OBP_CN12_GS8R7LM03EMD3J_53Frame2

-----------------AITAGERPLRPGEE-----------------------------

-------------------------------------AMS--ILI-------------AV

VKFLLALT------------------------L---CDAM--------TMKQ-IKSTGKM

MRKTCQPKI-----------------NVAD-E-------KI----DPLN-EGVF------

---IE------EK-EVMCYIACIMKM--ANAV----------------------------

-KN------N-K---------LNYEAAMKQADILFPE------EIKQP--AKD-AITAC-

-----RKV------ADS----YKDI------------------CEASFFVT-KCIYNHNP

A---I---

--FYFPLLNLWHKGKK

K---

>Bany-OBP_CN13_GE678549_53Frame3

--PLRPGSYK

SESVTKLKIRVGAIVYCSELNKIYKSMSNTF-----------------------------

-------------------------------------WFF--AFL---------------

SVALVNGK------------------------K---ATVT--------IPPE-YAGELIT

ASVDCVRDT-----------------GVDP-D-------ML----NQIV-QWKL------

---QE------ND-NVKKFIFCVATA--TGYG----------------------------

-DS---D--G-H---------VVVDKAEKLLSNSH--------KKKEE--FKN-ILKEC-

-----NKI------SGS------DK------------------YDTLYKTA-KCNREKQP

I---V---

--FSLKKLRVAYEGIN

KMKTQGCLLTSKFSLNKGLCLREINI-KILRKE---------------------------

>Bany-OBP_CN14_GS8R7LM02B1WGH_53Frame1

--LRPGRSHYGYLVSKLL

SSIVLNYIKHKINKINKINKINKYKTMSNTF-----------------------------

-------------------------------------WLF--AFL---------------

SVVTVYGK------------------------R---ATVT--------IPPE-YAAELVT

ASVGCVRNT-----------------GVDP-N-------IL----DQIV-QWNF------

---QE------TD-NVKKFIYCMSLA--SGFG----------------------------

-DS---D--G-H---------LIIDKVARLVSDSN--------KKKDE--LIN-ALKEC-

-----NTL------SGS------DK------------------YDTLFKTV-KCNREKQP

I---V---

--FSFIYLKHIIAMNT

TGGFCCLLRRYYQNQVSSYLKGCVWKTKIKGSKKK-------------------------

>Bany-OBP_CN15_GE680950_35Frame2

YGRGNGFGLRAEKQRNIDAAMTSAALLVFLFLVSFGHGAKESRNLARKSKEIIQHVHNE
C

VDKSSVSTQSGHYGRGRNKGITPLHLRRCLCFCSSFRLVTERKKSRNLARKSKKYNMFT
T

SVSIKPPRRTRIAKTAYLRRTHYLRLRGGETKEYRRRYDICGAACVSVPRFVWSRSERKA

GTRGNQRNNTTCSQRVCRNRSNRGGHSELRKRHIGGHKIEVLHVLFTGRRESCCWQCFY
S

TNASLWEKSSSNKYLLRGEKQRNIDAAMTSA-----------------------------

-------------------------------------ALL--VFL------------F-L

VSFGHGAK----------------------------EKPE--------LSEE-IKEIIQH

VHNECVDKT-----------------GVTE-E-------DI----ANCE-NGIF------

---KE------DT-KLKCYMFCLLEE--GSLV----------------------------

-DD---D--G-N---------VDYDMMISLIPE----------KYTDR--VGK-MINAC-

-----KHL------DTP----DKDS------------------CQRAFDVH-KCSYAADP

N---F---

--YFLFYSTNASLWEK

SSSNKYVLNIFAWFKKKKKKKLCVNCFKCCINRVYCIIYYIKCNNNRNLVKKEK------

>Bany-OBP_CN16_GS8R7LM03C9PJO_35Frame1

--------------HDS----IV-D--------------N---CSKTLEIS-KCFRTKIH

E---L---

--KWA----PSMEEIL

EEVMTEVMIENWLEITRLNDVFLYSRFANRQMVLGNHNIARGHSRFSFILYFL--VNI--

--------------------------------------IWESINKILNLNTGRSSDNLLQ

------------KTRKT-TCKALITK-LDYITRNNTNDIILYLVEYNPYIISHNNYFKVV

ASYFSLSPKRLCYGLDEMGQLNYDKVTKLNYIFHIMFLTMASLILLASERFVYVFIFFSY

LMSRLSSIFRPLSYNVYRRHHSSLLPSDKLWRYRYTSHFRASLLCTKR--------NLWR

LRNFGRENGAAYFNSMRGLNCLSAAAKTVSLRAPAVYDNIFCFGQHLYVFLSNSMIACL
V

NVFDTRSNSMIVC-----LLNVFHCNLMIACLLDVFHCNSMIACTLSLQLPNNTTTYLMF

YGFKYLTLLPATCQIAFRLFNPHVLCVFVVWNVNQTSVWYTKKKNYYKGK----------

>Bany-OBP_CN17_GS8R7LM02B5M46_35Frame1

-----ITAGVSTQSGHYGRDCEKARGTMASC-----------------------------

--------------------------------------WY--VMV--VV---------VM

SMVSRP-V------------------------A---GTAE--------VMSH-VTAHFGK

SLEECREES-----------------GLSP-E-------IL----E-EF-QHFWSED--F

--EVV------HR-ELGCALICMSNK--FSLM----------------------------

-ED---D--A-R---------MHHVNMHDYIKSFPKG----E-LLSAK--MVE-LIHNC-

-----EQE------YDD----IK-D--------------D---CSRVVKVA-ACFKVSAK

D---E---

--GIA----PEVAMIE

AVLEKYVHGIKSRTLFYFSFSH--VTRATDLQFFFYSLQVSSLQSHLMVSDDAIDESRQT

CVEDENLYPFRFLHDIVLERIAWRYVFVGRVVTSHGQSLPPARPGPIKKTSISPAGNRTQ

DLRLLNSPRTPLRYGGRQKCNCTVLSNRNFNQNHKNCTIKTVKNCRSVGYITYYFEITII

RITNFLFNRSFRRIEKKCTQLYYNNHFPFLCYCYLLRLVSKLTDVIITIVIDFENELDFH

KNNL---------VFIFSLLQNEKFTIHYLVHLIYCKLWTKGLNN---IVLSNLYVKIDY

RINFVRNRDYGESLRENGHFGILIKKQFKIKKKKKEKLCV--------------------

-D--

>Bany-OBP_CN18_GE654902_53Frame2

------VSTQSGHYAAITAGGEKARGTMASC-----------------------------

--------------------------------------WY--IMV--VV---------VM

SLVSRP-V------------------------A---GTAE--------VMSH-VTAHFGK

SLEECREES-----------------GLSP-E-------IL----E-EF-QHFWSED--F

--EVV------HR-ELGCALICMSNK--FSLM----------------------------

-ED---D--A-R---------MHHVNMHDYIKSFPKG----E-LLSAK--MVE-LIHNC-

-----EQE------YDD----IK-D--------------D---CSRVVKVA-ACFKVSAK

D---E---

--GIA----PEVAMIE

AVLEKYVHGIKSRTLFYFSFFKYNLQTFSFNCAVLIVQFSSLSELESPRRMRGVRPIVHH

AGPMRIGKHAENENSLVCRFPHDIFLHRLRHVIFFLKMHTTEKLECMPRTEFEPTPFGI-

------GGRGHIHWAITARYSLILSKFQSKSLEKLYSNCKKLPVCKLHNLLLKQLFVSLN

FCNLI--------------KVFEDSNAPNKCNTTMIIFHSFVIAIY--DLVNQRSELLSI

LNKMNWTSTKIISYNLANIYFQPFKKLRKVYNSLSSALNL-LIMDRPEYCAKYMQKIIDD

RISLYETETMANHCMKT-DTDLVYRSNLKIKKEKKKLCVD--------------------

-T--

>Bany-OBP_CN19_GS8R7LM02B0493_53Frame1

--------------------------------------GH--YGR---------------

-------G------------------------R---ESAR--------YHGV-VLVHYGG

DGDVYGVQT-----------------SGRY-S-------GD----E-PC-YCAFREI--F

--RRV------SR-RVGSLTRDSGRV--PTFL----------------------------

-ER---G--L-RGGAPAGLCPHLHVNMHDYIKSFPKG----E-LLSAK--MVE-LIHNC-

-----EQE------YDD----IK-D-------------D---CSRVVKVA-ACFKVSAK

D---E---

--GIA----PEVAMIE

AVLEKYVHGIKSRTLFYFSFFKYNLQTFSFNCAVLIVQFSSLSELESPLRMRKVRPIVHH

AGPMRIGRLYTRRELRKLSGFLTIFSFTVDTYFNFLKCTQLKSWRCMPRTGFETTPSGI-

------------------GGRGHIHWDITARYSLILSKFQSKSLEKLYSNKKLPVCRLQP

ITITIICITSFRRIEKKCTQLYDNNHFPFLCYFYLLRLVSKLTEVITIVIDFDYYELVFH

KNNL---------VQFSKHLFSAFYKTKENLQFIICTFIVNYVLKAIILCVTYMQKYIID

DRISLETETTANHCMKT-DTDLVYRSNLKLKKKKKKICVY--------------------

-H--

>Bany-OBP_CN20_GE680902_53Frame3

FFFVTTSPCREWVICAPAAFLGCGSRFSGSLSGIEPFPVTRDNHGSRRNYHRKLIRQTFE

RCVERRVAITAGGISTMNRNMNFVYAITFAL-----------------------------

-------------------------------------ITF--SVT--ECATKEKDKPNNA

KVETTTAS------------------------V---SVEE--------RDKS-EEFNLME

VMVECNDSF-----------------RVEM-S-------YL----A-SF-NKSGSFP---

---DE------TDKTPKCFMRCVLEK--SGVA----------------------------

-SP---A--S-Q---------FNVKRTAEIFPQIRDI-------AEED--IVK-IATEC-

-----TDR------PET-------C--------------K---CERSYQYL-KCLMETVI

E---I---

--YDV----KNTFAIF

GFPNKHIYPPLLFSLIYLFVLFNFKQKIYKTLLKFKKKIVILIALFSIFDFILMCAYSNV

KSKKEK--

>Bany-OBP_CN21_GE660702_53Frame2

-------------------AITAGDTISDIQ-----------------------------

-------------------------------------EDT--RVFYKMFIKKLFFYNFVI

LITFCNFN------------------------N---IHAK--------NADD-LKQNFMH

ILMECAKDY-----------------PITG-D-------DV----EQFK-NKQV------

---PD------NE-NMRCLLACAYKK--TGMM----------------------------

-DD---Q--G-M---------LSVEGVNKYTQEYLGD----DSNKLEK--AHD-FTDVC-

-----KSV------NDV----SVSD--------------GSKGCDRAALMF-KCSVDKAA

E---F---

--GFD----IVLYNPI

REEKELAHLERRIYAEPKTQIVIKLDIVCNVLYLLLRLSCIHLLLLQLYQIDHLDGKLNW

QMRIIEPLKLIIYFIAFKNTAFYIIDFIFDAFLMLSCQLFSLLYKSMLGIVSIQFLLLLN

IFKGKNIKIQRLAVCISLFYLVTLLVCFFKLIMAISSCVLKISTRNPFNTTGTVNSFFNY

YYVFVVGIKTNIEVVKKKEKRK--------------------------------------

>Bany-OBP_CN22_GS8R7LM03DK2TO_35Frame1

-----------YLRSGVFVVVKSSVTYWSFV-----------------------------

-------------------------------------VWY--VMV--VV---------VM

SMVSRP-V------------------------A---GTAE--------VMSH-VTAHFGK

SLEECREES-----------------GLSP-E-------IL----E-EF-QHFWSED--F

--EVV------HR-ELGCALICMSNK--FSLM----------------------------

-ED---D--A-R---------MHHVNMHDYIKSFPKG----E-LLSAK--MVE-LIHNC-

-----EQE------YDD----IK-D--------------D---CSRVVKVA-ACFKVSAK

D---E---

--GIA----PEVAMIE

AVLEKY-IHQIGNSIPDLILLFSSCNPGYRPSVFFTLYKLALDYNLTWSVMTQSRWKRAN

LLGGGNPYPFRFQHDIVLERIAWRYVFVDRVVTSHGQRPTSQTWTNNLNRPSWGSNPGP
-

------PSHTTAPRRPSKVLRSFNTVLSNRIFIKIFRKTVQFKLKKTAGLVVPITITIIC

ITKFLYFNRSFRRIEKKCTQLYNNNHFPSLCYCYLLRLVSKLTEVI--IVINFENELEFH

KNNL---------IHLANIYFQAFYKTKKSLQFIICTFIVNYVLKAIILCVTYMQKYIID

DRISLETETTANHCIEN-GT-------TRIVITKKKDXXX--------------------

-X--

>Bany-OBP_CN23_GS8R7LM03D9HP1_53Frame2

-------------------------------------EKD--TEL--TPEQRREEQSKYG

IFFDDDYN------------------------Y---LQHL--------KDTR-EEFNLME

VMVECNDSF-----------------RVEM-S-------YL----A-SF-NKSGSFP---

---DE------TDKTPKCFMRCVLEK--SGVA----------------------------

-SP---A--S-Q---------FNVKRTAEIFPQIRDI-------AEED--IVK-IATEC-

-----TDR------PET-------C--------------K---CERSYQYL-KCLMETVI

E---I---

--YDV----KIKPFQS

LVVPICILTLPFPLLLLFSSNFFLILKVLKNIQNTVKIYKKKLLLSSLYFQFLIYT----

>Bany-OBP_CN24_GW3I2KR01A0OH8_53Frame3

----------TAGGHCQSRTGRKLCDRRTGV----------------------------

-------------------------------------RMF--RNV--FL---------ML

CSVSVG-L------------------------Y---ADVE--------VMKD-VTLGFGE

ALQHCREES-----------------QLSE-D-------KM----E-EF-FHFWRDD--F

--KFE------DR-ELGCAIKCMSAH--YDLL---------------------------

-TD---S--H-R---------MHHENTDRFIKSFPNG----E-VLSQQ--MVQ-LIHEC-

-----EQQ------HDA----EE-D-------------H---CWRILRVA-ECFKGACQ

R---H---

--GIA----PTMEMLM

AEFIMEAESRTTTALVSLHFTSKYHLYFYTNISRVLNALISGTTGPIKIISVLDSPFIEG

YYP--------------TGARTTRVKPRGVSYYHKLTNGLDTKAGRIHPFFVFVILMRNF

DLEFRTLRFSSSGMYIVSSARIRYVREEFVQFENKNVLAGGSPPYRQRRTTKQFRVPYDV

VWIFIPLLTSPAFILDCIITDYQVRLRANLRKRKKNVHVRHLMVFYIDFMHSVFRTLKTD

SENVGIRKYVKGVYAAGLNVNLLRTSGRPHHLREKLKNTCMFIMETDAWSTHYSHNPG
LI

YLKYVAIQTRLPEPVVYTSLLMHGTVSGTTKYTTSKYFYYTYAAPIVCNVHIKAMFKNI

KE--

>Bany-OBP_CN25_GW3I2KR01AJ48K_53Frame3

------------XXXITAGDCEKARGTMASC-----------------------------

--------------------------------------WY--VMV--VV---------VM

SMVSRP-V------------------------A---GTAE--------VMSH-VTAHFGK

SLEECREES-----------------GLSP-E-------IL----E-EF-QHALYQE--L

SQRAV------ISKDGRTNTLARVRH--QGLQ----------------------------

-PR---GEGGGV---------LQSERQGRRNRSSGYD----RSGLRKV--LSS-LVGNIP

DLILLFFV------PSDLQFLRSFN------------------CTVFIIIARVSSQNERG

A---N---

--SPPRWPNADWQTLH

TQRIKETLWCAGFHTIFSFTVDTYFNFLKCTQLKSWSVCRGPDSNLRPPESEAEVIFTG-

------------------LSRLGTVLSNRNFNQNHKNCTVKIEKKLPVCRLHNLLLNNNY

SYHFFVISKFSKNRMKKCTQLYYNNHFPFLCYCYLLRQVSKTEVIIVINFEN--------

>Bany-OBP_CN26_GW3I2KR01BW94F_53Frame1

--M----E-EF-FHFWRDD--F
--KFE------DR-ELGCAIKCMSAH--YDLL----------------------------
-TD---S--H-R---------MHHENTDRFIKSFPNG----E-VLSHT--FF--------

>Bany-OBP_CN27_GZKG99Z02C1ZKR_53Frame3

XXXT--

-------------IYGPGVNRLLVRPSMTSA-----------------------------

-------------------------------------ALL--VFL------------F-L

VSFGHGAK----------------------------EKPE--------LSEE-IKEIIQH

VHNECVDKT-----------------GVTE-E-------DI----ANCE-NGIF------

---KE------DT-KLKCYMFCLLEE--GSLV----------------------------

-DD---D--G-N---------VDYDMMISLIPE----------KYTDR--VGK-MINAC-

-----KHL------DTP----DKDS------------------CQRAFDVH-KCSYAADP

K---F--

--YLLF-STLLMLADW

EKSYKILF--

>Bany-OBP_CN28_GE681934_35Frame2

---YGRGQ

LQSYRRYEVQSSIVLSI----KKYKSMFNTF-----------------------------

-------------------------------------WFF--AFL---------------

SVALVNGK------------------------K---ATVT--------IPPE-YAGELIT

ASVDCVRDT-----------------GVDP-D-------ML----NQIV-QWKL------

---QE------ND-NVKKFIFCVATA--TGYG----------------------------

-DS---D--G-H---------VVVDKAEKLLSNSH--------KKKEE--FKN-ILKEC-

-----NKI------SGS------DK------------------YDTLYKTA-KCNREKQP

I---V---

--FSLKKLGVYQNEVD

AR------LSSDFQVFKRVVFRGNKLNNIVNIT---------------------------

>Bany-OBP_CN29_GE684370_53Frame2

---------------------M----V-E-------------------------LIHNC-
-----EQE------YDD----IK-D--------------D---CSRVVKVA-ACFKVSAK
D---E---

--GIA----PEVAMIE
AVLEKY--

>Bany-OBP_CN30_GE679318_35Frame1

--LED--Y

--ELL------NR-ETGCAIMCMASK--FDLI----------------------------

-AD---D--M-S---------LHHQNAHEFAKTHGAD----D-DMAKQ--LVN-MLHDC-

-----EKV------HDS----IV-D--------------N---CSKTLEIS-KCFRTKIH

E---L---

--KWA----PSMEEIL

EEVMTEVMIENWLEITRLNDVFLYSRFANRQMVLGNHNIARGHSRFSFILYFL--VNI--

--------------------------------------IWESINKILNLNTGRSSDNLLQ

------------KTRKT-TCKALITK-LDYIT----------------------------

>Bany-OBP_CN31_GW3I2KR01B8IJW_53Frame2

---------------------ESTPPECAVAKTHGAD----D-DMAKQ--LVN-MLHDC-
-----EKV------HDS----IV-D--------------N---CSKTLEIS-KCFRTKIH
E---L---

--KWA----PSMEEIL
EEVMTEVMIENWLEITRLNDVFLYSRFAN-QMVLGNHNIARGHSRFIVFKIPVLNTKGRR
KQKYNI------------------------------DSLTFGSQLINRYRIILVRLVVTC
-----------CKTRKTLTSKALTTKLILYCTLNITLTFNIYV--------SHNNYIEVV
ASYFSLSPKRLCYGLDEMGQLNYDKVTKLNYIFHIMFLTMASLILLACERFVLLCFYYFF
FYIVGDYREAYLGHVTTSTREEHHSSLLPSVQ----------------------------

>Bany-OBP_CN32_GE680582_35Frame1

--HHRHHHHHHNVP

TRRHGTFALSRPGPIYLRSGAREREGTMASC-----------------------------

--------------------------------------WY--IMV--VM---------VM

SMVSRP-V------------------------A---GTAE--------VMSH-VTAHFGK

SLEECREES-----------------GLSP-E-------IL----E-EF-QHFWSED--F

--EVV------HR-ELGCALICMSNK--FSLM----------------------------

-ED---D--A-R---------MHHVNMHDYIKSFPKG----E-LLSAK--MVE-LIHNC-

-----EQE------YDD----IK-D--------------D---CSRVVKVA-ACFKVSAK

D---E---

--GIA----PEVAMIE

AVLEKYVHKQVGNSIPDLILLFFSCNLGYRPSVFFYSLQVSPLQSHLMDSVITGTCHDEE

TRFLMSIFPGCMDRGHDGIEVASKTRGCKKK-----------------------------

>Bany-OBP_CN33_GS8R7LM03DRG7F_53Frame1

----------------------------------FFFFQLNNILVAIIFLKSIRHNDLQL

LFYIAQTVVCIHQTVDNFWASKGGIFSKDSMSTSFKLLYSSLSLFSLFFSKSFSLKGFFF

FLALLDFGSLPPRAGSGFARLLPSLDVVAVSQAPSPESNPDSPLPVTTMVVAENYHRKVD

KADIKMRRRYQRRVAITAGDCEKARGTMASC-----------------------------

--------------------------------------WY--VMV--VV---------VM

SMVSRP-V------------------------A---GTAE--------VMSH-VTAHFGK

SLEECREES-----------------GLSP-E-------IL----E-EF-QHFWSED--F

--EVV------HR-ELGCALICMSNK--FSLM-----------------------------

-RT--------------------------ISRAFPKV------SCYQQ--RWNYITVSK-

-----SMT-----------------------------------TSRMTAAA-WRWRRASK

A---

---PRTKESL

LKWL--

>Bany-OBP__CN3435_GW3I2KR01CVXZX

-----------------TAGGEKARGTMAPC-----------------------------

--------------------------------------WY--IMV--VV---------VM

SMVSRP-V------------------------A---GTAE--------VMSH-VTAHFGK

SLEECRE------------------------E-------IL----E-EF-QHFWSED--F

--EVV------HR-ELGCALICMSNK--FSLM----------------------------

-ED---D--A-R---------MHHVNMHDYIKSFPKG----E-LLSAK--MVE-LIHNC-

-----EQE------YDD----IKDD------------------CSRVVKVA-ACFKVSAK

D---E---

--GIA----PEVAMIE

AVLEKYVHGIKSRTLFYFSFLSITFRPSVKTVKNCRSVSYITYYLNNNYSYHIFVISKFS

KIRMHLTNVIQQFSIPLLLLFIKTSINRGLNNYCNRFIKIGLPQNNL-------------

-------------VQFSKHLFSAFKTKESLTITLICTFIVNYGLK---------------

>Bany-ABPX_CN36_GW3I2KR01AZ1VU1_53Frame2

---XXXXYGRGDIIALVNQS

ALPRGITSMAITAGEVTFNWFNFYSNKMASK-----------------------------

-------------------------------------LVL--WMV---------------

--LSCLVL----------------------------GAFG--------MDAE-MAELAKM

LRESCVDET-----------------GVDV-G-------LI----DKVN-AGADL-----

---MQ------DN-KLKCYIKCVMET--AGMM----------------------------

-SG------G-D---------VDVEAVIAVLPE----------ELKKH---AN-TMRSC-

-----GTQ------KGT------DD------------------CDTAFKTQ-ECWQKGNK

Q---D---

--FFLILSNTYY----

>Bany-OBP_CN37_GE680035_53Frame1

------------GHLRPGEKQRNIDAAMTSA-----------------------------

-------------------------------------ALL--VFL------------F-L
VSFGHGAK----------------------------EKPE--------LSEE-IKEIIQH
VHNECVDKT-----------------GVT--E-------DI----ANCE-NGIF------
---KE------DT-KLKCYMFCLLEE--GSLV-----------------------------
-DD---D--G-N---------VDYDMMISLIPE----------KYTDR--VGK-MINAC-
-----KHL------DTP----DKDS------------------CQRAFDVH-KCSYAADP
N---F---

--YFLFYSTNASLWEK
SSSNKYVLNIFAWYVKKEK-------------------------------KKRK------

>Bany-OBP_CN38_GZKG99Z02C7YYH_35Frame2
YLRSGGS---

------------KLKVIISTSVLLYYSCRSS-----------------------------
-------------------------------------CLC--CFL------------VSS

FVLGHGAK----------------------------EKPE--------LSEE-IKEIIQH

VHNECVDKT-----------------GVTE-E-------DI----ANCE-NGIF------

---KE------DT-KLKCYMFCLLEE--GSLV----------------------------

-DD---D--G-N---------VDYDMMISLIPE----------KYTDR--VGK-MINAC-

-----KHL------DTP----DKDS------------------CQRAFDVH-KCSYAADP

N---F---

--YFLFYSTNASLWEK

SSSNKKVLEIKIITTIGF--------------------------------KKEK------

>Bany-OBP_CN39_GZKG99Z02CZ4PM_53Frame3

----------------TAGDCEKARGTMASC-----------------------------

--------------------------------------WY--IMV--VM---------VM

SMVSRP-V------------------------A---GTAE--------VMSH-VTAHFGK

SLEECREES-----------------GLSP-E-------IL----E-EF-QHFWSED--F

--EVV------HR-ELGCALICMTIS--RAFP-----------------------------

-KV------SCY---------QQRWNYITVSKSMTTS----RMTAAAW--RWR-RASKAP

RTKESHLK------WRK----RSWK---------------SIKFTSRELNPG-PYFTLVFL

S---I---

--TFR----PSVKTVK

NCRSVSYITYYLNNNYLYHFSKNRMKEMHLTNVVQQSFPLLLLFIKTSINRGLNNYCNRF

LIGFP---

>Bany-OBP_CN40_GZXD38A01B7FTS1_53Frame3

-----------------TAGGEKARGTMASC-----------------------------

--------------------------------------WY--VMV--VV---------VM

SMVSRP-V------------------------A---GTAE--------VMSH-VTAHFGK

SLEECQEL------------------SQRA-V-------IS----KDGR-TNTLARV--R

HQGLQ------PRGEGGGVLQSERQG--RRNR----------------------------

-TS--------G---------YDRSGLGKVLSSLVGNIPDLILLFFKY--NLQTFSFNC-

-----AVL------IVQFSSLSELE--------------S---PRRMRGVRPIVHHAGPM

R---I---

--GKH----AENEKLL

LGNSRFPHDIFLH---

>Bany-OBP_CN41_GZXD38A01B7FTS2_53Frame1

---LRPGARK

REVPWRRVG------------------------------TL---------WWWW------

--------------CLWCPDQWLVQL--RAML----------------------------

---------------------LRISGNLKSVKSFPKG----E-LLSAK--MVE-LIHNC-

-----EQE------YDD----IKDD------------------CSRVVKVA-ACFKVSAK

D---E---

--GIA----PEVAMIE

AVLEKYVHGIKSRTLFYFSFLSITFRPSVLTAQFLYSFHHYLSSSLLAEEGLGQSTTLAQ

CGLANTQRIKKNYYVIAGFLTIFSF-----------------------------------

>Bany-OBP_CN42_GZXD38A01BULAD_53Frame2

--------------------------------------IT--------AGERIGHVHSYS

GRKISVSTY-----------------TLRR-E-------KI----DPLN-EGVF------

---IE------EK-EVMCYIACIMKM--ANAV-----------------------------

-KN------N-K---------LNYEAAMKQADILFPE------EIKQP--AKD-AITAC-

-----RKV------ADS----YKDI------------------CEASFFVT-KCIYNHNP

A---I---

--FYFPLLNLW-----

>Bany-OBP_CN43_GZXD38A01B8Y5N_35Frame1

-------------LFTVRGSRRRLPIGRSLF-----------------------------

-------------------------------------GYY--LLVGCIG---------LF

LLVSRP-V------------------------A---GTAE--------VMSH-VTAHFGK

SLEECREES-----------------GLSP-E-------IL----E-EF-QHFWSED--F

--EVV------HR-ELGCALICMSNK--FSLM----------------------------

-ED---D--A-R---------MHHVNMHDYIKSFPKG----E-LLSAK--MVE-LIHNC-

-----EQE------YDD----IK-D--------------D---CSRVVKVA-ACFKVSAK

E---R---

--RKSNRSGTRKAMIE

AVLEKLQLSRELNPGPYFTLVF--------------------------------------

>Bany-OBP_CN44_GZKG99Z02C6VHV_35Frame1

LFTVRGK---

------------KLKVIISTSVLLYYSCRSS-----------------------------

-------------------------------------ALL--VFL------------F-L

VSFGHGAK----------------------------EKPE--------LSEE-IKEIIQH

VHNECVDKT-----------------GVTE-E-------DI----ANCE-NGIF------

---KE------DT-KLKCYMFCLLEE--GSLV----------------------------

-DD---D--G-N---------VDYDMMISLIPE----------KYTDR--VGK-MINAC-

-----KHL------DTP----DKDS------------------CQRAFDVH-KCSYAADP

N---F---

--YFLFYSTNASLWEK

SSSYKICFRNNNYDVVK---------------------------------KEKR------

>Bany-ABPX_CN45_GE680205_53Frame2

--------RPLRPGHVTFNWFNFYSNKMASK-----------------------------

-------------------------------------LVL--WMV---------------

--LSCLVL----------------------------GAFG--------MDAE-MAELAKM

LRESCVDET-----------------GVDV-G-------LI----DKVN-AGADL-----

---MQ------DD-KLKCYIKCVMET--AGMM----------------------------

-SG------G-D---------VDVEAVIAVLPE----------ELKKH---AN-TMRSC-

-----GTQ------KGT------DD------------------CDTAFKTQ-ECWQKGNK

Q---D---

--FFLILQYILCVCIY

KTLYFKKKK---

>Bany-OBP_CN46_GZKG99Z02D3ZH1_53Frame1

---GHYGRGK

QRNIDAAMT-----------------SAAL---------LV-------------------

--------------FLFLVSFGHGAK--EKPV----------------------------

-DD---D--G-N---------VDYDMMISLIPE----------KYTDR--VGK-MINAC-

-----KHL------DTP----DKDS------------------CQRAFDVH-KCSYAADP

N---

--FLLFYSTNASLWEE

VVKICFELNIRIGLKEK---

>Bany-OBP_CN47_GZKG99Z02DM7MM_35Frame2

---------------------------AACV-----------------------------

-------------------------------------SVP--------------------

RSFGHGAK----------------------------KKTG--------TARK-IKEIIQQ

VHNECVDKT-----------------GVTE-E-------DI----ANCG-NGIF------

---KE------DT-KLKCYMFCLLEE--GSLV----------------------------

-DD---D--G-N---------VDYDMMISLIPE----------KYTDR--VGK-MINAC-

-----KHL------DTP----DKDS------------------CQRAFDVH-KCSYAADP

N---F---

--YFLFLVLYCLTMGE

VVKICFELNIRIGLKKKKEKRK--------------------------------------

>Ppol-OBP_Gene0006371_D2SNQ6_HELVIOdorantbindingprotein

--------------------------------------MS--ILT-------------VL
VKFLVTVT------------------------I---CEAM--------TMKQ-IKSTGKM
MRKSCQPKN-----------------NVED-E-------KI----DPLN-DGVF------
---IE------EK-DM--
-KN------G-K---------LNYEAAIKQADMLLPD------EIKEP--TKA-AITAC-
-----RKV------ADS----YKDV------------------CEASFYVT-KCIYKENP
D---I---
--FFFP----------

>Ppol-OBP_Gene0006368_C0SQ83_BOMMOOdorantbindingprotein

---------------------------MYKL-----------------------------
-------------------------------------YLY--VNL------------CLI
ALMPFKSH------------------------A---MTAE--------QKAA-IHEHFEE
LGTECMKDY-----------------PITE-E-------DV----NNLR-AKKI------
---GT------GE-NVPCFLSCLLKK--VGIM----------------------------
-DD---K--G-M---------LQKETALEYAKKVFND-----AEELKL--IEG-YLHSC-
-----SHI------NGE----TVTD--------------GEKGCDRALLSF-QCMLENAS
Q---F---

--GFD----V------

>Ppol-OBP_Gene0006898_C0SQ81_BOMMOOdorantbindingprotein

--------------------------MLYF------------------------------

-------------------------------------VVV--LSL-------------MS

MVALGEGS------------------------K---PSIQ--------LPAI-VVNVAKE

AASTCLTET-----------------GASQ-E-------VS----DNFF-KLKF------

---GT------DP-DSKSFLYCVARK--TNYA-----------------------------

-DE---D--G-----------HLNEALLTLFEGS----------EHKDG--VAK-VMETC-

-----NKN------QES------NK------------------IDTMHKTV-ECFYKNTP

V---H---

--LAV-----------

>Ppol-OBP_Gene0010513_D4AHN1_9MUSCOdorantbindingprotein1

---MYA-HDKASDV

VADQCLSEM-----------------YPKG---------------KRVE----F------

-----------QESDEACIIYCVLKK--FGIM----------------------------

-NA---N--G-V---------INLEAYRKRVLLAHQL------DQRKL--MSDSSGSSC-

-----AES------AEG-VQHKQDV------------------CKKAKIFN-DCTHLYKI

L---L---

>Ppol-OBP_Gene0006369_C0SQ80_BOMMOOdorantbindingprotein

---------------------------MSPL-----------------------------

-------------------------------------HSV--FLL--LG----------L

AVSLRHVR------------------------A---LSQE--------EIAA-IKTGLRP

LIAECGKEF-----------------GVEE-A-------DI----KKAK-ESGK------

-----------IESLDPCLFACIGKK--MGMI----------------------------

-ND---K--G-E---------FDVEKSSETVKKFVTD-----KDEQKK--ILE-IIEKC-

-----ASV------NDE----AVSD---------------DKGCDRAILLH-KCMAPYKD

Q---F---

--DFS----K------

>Ppol-OBP_Gene0005282_OBP2_BOMMOGeneralodorant-bindingprotein2

---------------------------MGLL-----------------------------
-------------------------------------HWL--SCV--LV---------VL
ALGASP-A------------------------A---ATAE--------AMSH-VTAHFGQ
MLEDCREES-----------------GLTV-D-------MM----A-AF-AHYWSDE--F
PTSSV------QR-EFGCALICMAHK--FSLL----------------------------
-KD---D--V-R---------MHRVNMDDYIRSFPDG----E-LLSDK--MID-MIHEC-
-----ERQ------HDA----ME-D--------------D---CDRIMNIS-LCFRRAAL
R---D---

--GIA----PSLAMVE
AVLDQYT---

>Ppol-OBP_Gene0002871_B8ZWK2_BOMMOOdorant-bindingprotein2_Precursor_

---------MRILRKKSVPSNRIKMNQNTNF-----------------------------

-------------------------------------VTV--MMF--LTFISVFYLVVTF

KPLTKEEH------------------------I---EKIN--------KMDS-EVEPFRR

NISECARQV-----------------KAGK-G-------DV----ENFL-KRIP------

---QA------TM-EGKCFVACILKR--NSII----------------------------

-KK------N-K---------IDHNSLLEANKAVYGE----DREVMTR--LKA-AIVEC-

-----NRA------VEG----IFEI------------------CEYASVFN-DCMHIKME

H---I---

--LDK----LTMERRM

EALGQMSNSPDEWTDEEDEMLKLVKDEL--------------------------------

>Ppol-OBP_Gene0005278_OBP1_BOMMOGeneralodorant-bindingprotein1

---------------------------MRC------------------------------

--------------------------------------AA--ALA--AL---------AM

LATAPA-G------------------------L---ATSD--------IMKD-VTLGFGE

ALQHCREES-----------------QLTE-D-------KM----E-EF-FHFWRED--F

--RFD------ER-ALGCAIQCMSRH--FDLL----------------------------

-TD---A--H-R---------MHRENTDRFIKSFPNG----E-ALSQQ--MVD-MIHAC-

-----EAQ------HDA----EP-D--------------H---CWRILRVA-ECFKASCQ

Q---R---

--GIA----PTMEQIM

AELIMEAAD---

-R--

>Ppol-OBP_Gene0010531_B8ZWK3_BOMMOOdorant-bindingprotein3_Precursor_

---MVA

TAKSCVNHV-----------------NATQ-E-------DL----E-YL-RNDP------

---PY------PE-KSSCIIMCLLEK--VGVV----------------------------

-KN------N-K---------YSKTGFMMAVSPLVLA----KKSKLEH--MKT-VSENC-

-----DKE------VNH----HDIV--------------P---CQLGNEII-TCVYKYAP

E---L---

--HLK----S------

>Mcin-OBP_gene1_MCINX010046-RA_ABP5

--------------------MKTLLKITLIA-----------------------------
-------------------------------------VTF--SMT--KCEKKIEN---KM
NPVVLTTA------------------------A---SMED--------GDGK-TFVDVMG
VMIDCNDTF-----------------RVEM-P-------YL----D-SF-NKSGSFP---
---DE------TDRTPKCFMRCVLEK--VEVV----------------------------
-SE---D--G-Q---------FDVNRTAEVFPQIRDV-------PQEE--IIE-MATPC-
-----AER------SES-------C--------------K---CERSYQYM-KCLLEKVI
D---K---

--YDT----T------

>Mcin-OBP_gene2_MCINX009685_OBP

---------------------------MLKF-----------------------------
-------------------------------------TVY--SCL-------------GA
PKIVSS--DVAS-QCNSSLP
EVYSCLGAP-----------------KIVS-P-------DV----ASQC-N---------
-----------SSLPECERMTCIFRK--SGWM----------------------------
-DG------E-K---------VDKAKLSAHFDQLAKN----NPEWAAG--VEN-AKITC-
-----LTT------DLP----AQGI--------------H---LNCPAYDSTVCTFASFI
----K---

--NTQPS--QWKSNPK
CSRARQFAASCPICPNDCFAPLVPVGSCNACLSLPRSP----------------------

>Mcin-OBP_gene3_MCINX014308-RA_OBP

---------------------------MFRL-----------------------------

-------------------------------------FTS--VTL-------------FI

LCVNGDIL--AQER-SRGATLK

PISACCDIP-----------------ELGEPK-------HL----AECS-NP--------

-----------KLQGPCGDVQCVFEK--SGFL----------------------------

-ID---K--N-T---------LNKETYKAHLRKWLEG----HKGWEDA--IDK-AIKDC-

-----VDK------DLR----QYLD------------------YPCRAYDVFTCTGIAML

----K---

--KCP------NESWN

C---

>Mcin-OBP_gene4_MCINX016480-RA_GOBP2

---------------------------MAVW-----------------------------
--------------------------------------YL--ILT--AV---------VL
SAIPKP-V------------------------K---STAE--------VMSH-VTAHFGK
SLDECREES-----------------GLSP-E-------VL----D-QF-QHFWSED--F
--EVV------HR-ELGCALICMSNK--FSLM----------------------------
-QD---D--A-R---------MHHVNMHDYVKSFPQ------------------------

>Mcin-OBP_gene5_MCINX014306-RA_OBPLOC100307012precursor

IVIAEPTP--PQCR-GPPPGMT
HPEKCCNFP-----------------SLFKDE-------DF----EECG-IERFSDD---
EDHSK------RGPLDCSQEKCLLNK--YKMM----------------------------
-KD---D--E-E---------IDKDATIEFLDKWAGD----NQDYKDS--VEK-AKEKC-
-----LKT------EIL----DAK-------------------LPCRPTTLFRCIKYAIF
----V---

--ECTQHM-QWEDTDN
CKKLKDLIEECKPYITQP--

>Mcin-OBP_gene6_MCINX017046-RA_GOBP19a

---------------------------------------M--------TRQQ-LKNSGKL
MKKSCMPKN-----------------DVTE-E-------QV----GKIE-QGHF------
---LE------ER-NVMCYIACIYTM--TQVV----------------------------
-KN------N-K---------LSYEAVIKQVDLMFPP------DMKDA--VKA-TADKC-
-----KDI------GKK----YKDI------------------CEASYWTA-KCMYEQDP
K---N---

--FLFP----------

>Mcin-OBP_gene7_MCINX015625-RA_OBP3

---KTLKA
TARSCMVSI-----------------NATE-A-------DL----E-YL-RDDP------
---PF------PH-KSACVITCLLEK--VGIA----------------------------
-KN------G-K---------YSKTGFMVTVSPLVLH----NMKKLEH--MKN-VSENC-
-----EKE------IKP----NEDP-----------------CQLGNEIT-ICVFKYAP
E---L---

--HFK----S------

>Mcin-OBP_gene8_MCINX017047-RA_GOBPLush

--------------------------------------MM--------TRAQ-LKKTMTI

MKNQCMPKH-----------------GVTN-D-------KV----GKIE-QGVF------

---IE------DH-DVMCYILCVYKT--IQVV----------------------------

-KN------N-R---------LDKDLISKQVNALYPP------EIKEP--VSK-SIEKC-

-----IIV------QDK----YEDP------------------CEGVFYST-KCLYEDNP

A---N---

--FIFP----------

>Mcin-OBP_gene9_MCINX016478-RA_GOBP1

----------------MAWNSKW---RS--------------------------------

---------------------------------------A--V----FI---------CL

LLHVNW-A------------------------S---PSQE--------IMKN-LTAGFGK

ALETCKQEL-----------------NLGD-H-------II----Q-DF-YNYWREE--Y

--DLV------NA-DTGCAIMCMASK--LDLI----------------------------

-TD---D--R-K---------LHHGNAHEFAKSHGAD----D-DMAKQ--LVV-MIHEC-

-----EKT------HAE----VS-D--------------D---CARTLEIA-KCFRTKIH

V---L---

--KWA----PSMETII

EELMTE--

------------------------------V-----------------------------

>Mcin-OBP_gene10_MCINX002679-RA_GOBP1

---------------------------M--------------------------------

--------------------------------------AR--RMV--VL---------ML

VSKLLV-V------------------------R---GNVE--------VMKD-VTLGFGE

ALQHCREES-----------------QLTE-D-------KM----E-EF-FHFWRDD--F

--RFE------DR-ELGCAIKCMSTH--FNLL----------------------------

-TD---S--H-R---------MHHENTDKFIKSFPNG----E-ALSQL--MVE-VIHAC-
-----EQQ------HDA----EA-D--------------H---CWRILRVA-ECFKSSCQ
A---R---

---GIA----PTMELLM
AEFIMETEA---

-R--

>Mcin-OBP_gene11_MCINX002972-RA_GOBP56dlike

---------------------------MSKI-----------------------------
-------------------------------------VVF--WIF---------------
-CLSFYLM----------------------------IAGS--------NAGG-ITKSVDY
HLEECIAES-----------------KADR-D-------SI----KKLR-AGNWR-----
---KS------DK-ALKKWALCYLSK--HDVM----------------------------
-ST---N--G-V---------LNQDVVLKNIPI----------KDKPH--VEK-IINKC-

-----LYK------QAH------DP------------------IETAWNYL-TCFIKNER

K---Y---

--AKL----ANLI---

>Mcin-OBP_gene12_MCINX014307-RA_LOC100307012precursor

---------------------------MLRI-----------------------------

-------------------------------------ASS--CLL--II---------LQ

VVIAELPP--PQCR-GPPPGVT

HPEKCCNFP-----------------RMIKDE-------DL----EDCG-IEKYSDD---

EDHSK------RGPLDCSKQKCLLNK--YNLM----------------------------

-KD---D--E-E---------IDKDAAIEFLDKWAGD----NQDYKDV--AEK-AKEQC-

-----LTT------ELP----GPK-------------------LPCGPTKILFCVKAVMF

----M---

--ECSQRM-QWEDTDN
CKKLKDHIEECKPYFTRT--

>Mcin-OBP_gene13_MCINX008882-RA_GOBP56a

---------------------------MYPI-----------------------------
-------------------------------------LCL--VLL---------------
TTSMVYGK------------------------T---TYVS--------IPQD-QLPNLLN
SSMACMANS-----------------GIDS-E-------TM----QRIL-AWKF------
---DN------TE-GVRKFIYCFATT--SGYG----------------------------
-DE---N--G-H---------LIKEKMMKLVTNN---------KRKNE--YGN-VIDEC-
-----NKT------KGD------NK------------------YDTMYKTT-TCFYNNSP
I---L---

--LKL-----------

>Mcin-OBP_gene14_MCINX001051-RA_GOBP19a

--------------------------------------MI--VFI-------------AI
VKILVFLN------------------------F---CDAM--------TMKQ-IKSTGKM
MRKTCQPKN-----------------NVAD-E-------KI----DPMV-KGEF------
---IE------EK-EVMCYVACIMKM--ANAI----------------------------
-KN------G-K---------LNYEAAMKQADLLLPD------EIKEP--AKE-ALTAC-
-----RKV------ADS----YKDV------------------CESSFFLT-KCIYNHNP
S---V---

--FYFP----------

>Mcin-OBP_gene15_MCINX002973-RA_GOBP56like

---------------------------MKTF-----------------------------

-------------------------------------IVL--AVC---LV----------

------------------------------------AAQA--------LTDE-QKEKLKK

HRSECLTET-----------------KADE-Q-------LV----NKLK-TGDFK-----

---TE------NE-PLKKYALCMLIK--SELM----------------------------

-TK---D--G-K---------FKKDVALAKVPNE---------ADKPA--VEK-LIDTC-

-----LAN------KGN------TP------------------HQTAWNYA-KCYHEKDA

K---H---

--SVF---Q------

>Mcin-OBP_gene16_MCINX017048-RA_GOBP19a

----------------M----------DRRD-----------------------------

-------------------------------------FSL--LII-------------VT

VL----AS------------------------G---ADSM--------TRQQ-LKNSSKM

LKKNCMGKN-----------------DVTE-D-------MV----GNIE-KGKF------

---IE------DR-KVMCYIACIYQM--SQIV----------------------------

-KN------N-K---------LNYEASIKQVDMMFPA------EMKDS--MKA-SIDNC-

-----KDI------SKN----YKDI------------------CEASYWTA-KCIYDDNP

K---N---

--FIFA----------

>Mcin-OBP_gene16b_MCINX017048-RB_GOBP19a

---------------------------------------M--------TRQQ-LKNSSKM
LKKNCMGKN-----------------DVTE-D-------MV----GNIE-KGKF------
---IE------DR-KVMCYIACIYQM--SQIV----------------------------
-KN------N-K---------LNYEASIKQVDMMFPA------EMKDS--MKA-SIDNC-
-----KDI------SKN----YKDI------------------CEASYWTA-KCIYDDNP
K---N---

--FIFA----------

>Mcin-OBP_gene17_MCINX000396-RA_PutativeOBPA10

--------------------------------M---QFVT--------IVLS-LLMTLVL
GYDEKYDKI-----------------DVDK---------IL-------------------
---GD------DA-LLTSYLNCFLDK--GPCN----------------------------
-EE---Y--A-S---------EYKEMLPEVISDGCAKC---TKKQKQA--FRK-VLKEL-
-----SKN------KLD----VLLE------------------VQKKYDPS-GQYKDNIK
----K---

--VME----ED-----

>Mcin-OBP_gene18_MCINX017054-RA_PutativeOBPA10

---------------------------MKSI-----------------------------
-------------------------------------ILL--FCF---------------
---A-AFVIAEE
TYDTSSDNI-----------------NLDE---------LL----GNER-----------
--------------LITSYSKCLINQ--GPCT----------------------------
-PE------V-K---------KLKDVLPEVLETRCAKC---SEKQKQK--GKQ-LISEI-
-----KKK------NPE----IWKQ------------------LVSFYDPQ-GKYQEAFQ
D---F---

--LKS-----------

>Mcin-OBP_gene19_MCINX014913-RA_PutativeOBPA10

---------------------------MKFF-----------------------------

-------------------------------------IHW--CFQ---------------

--------------------------------F---TLVI--------CFAY-QYNCENS

TYTTMYDGI-----------------DLDE---------IL----TNDR-----------

--------------LLAGYVNCLLEK--GPCT----------------------------

-PD------G-K---------ELKNNLPDAIENDCSKC---TERQREG--ADK-VMHYI-

-----IDH------KPE----EWEE------------------LEKKYHSD-GSYKLNYL

L---S---

--KQK----LSLDSQE

NKNVSNGDNGDDDDDDNDKDHQGGQQLK--------------------------------

>Mcin-OBP_gene20_MCINX014305-RA_noname_PblastOBP

---------------------------MLTN-----------------------------

-------------------------------------EQF--LFK--KK-----------

--PEKC-YGTDELM

ERKCCIFPP-----------------FFKR---------DL----ARAC-GAIFALV---

---FT------DKDSNCDHWKCVLNK--YGIL----------------------------

-DT---D--D-T---------VNNEKYYSHLDKWVNL----NPEFSNV--MTK-AKVYC-

-----KEQ------NRL----AIPL------------------NVCEFFDFQSCIRNYVL

----L---

--DCP----KIINNQK

CLEWKEFYDECREFFI--

>Mcin-OBP_gene21_MCINX001722-RA_PBP1-like

--------------------------------------AK--------TDHE-LKEEFTK

IVMACMKDS-----------------PVDI-L-------DL----MPLQ-QLIV------

---PT------KK-EVKCLLACAYKK--LGTM----------------------------

-NS---Q--G-L---------YDIEKAYEYAELVKNGSPTEDEKRLKN--ARK-LVDIC-

-----SKV------NDE----SVSD--------------GQKGCDRAALIF-KCGVENAP

K---L---

--GFK----LQ-----

>Mcin-OBP_gene22_MCINX001052-RA_PBP2-partial

--------------------------------------AR--------TDIE-IKKWFIQ
QALDCTKEH-----------------SATS-E-------EI----QMIK-EHKI------
---PS------SN-NAKCLMACMFKK--LNWI----------------------------
-DE---K--G-M---------FSDKNAYKWSENEYPS----DHAKLEN--AKK-LYELC-
-----MK---

>Mcin-OBP_gene23_MCINX012604-RA_noname_PblastOBP

---------------------------MPFL-----------------------------

-------------------------------------FQI--------------------

----RADC------------------------K---NCVV--------LGKA-EKAMFRA

HSEACLAQS-----------------QVEP-K-------LV----DGLL-RGEL------

---VE------DP-RLKRHVYCVLLK--CKMI----------------------------

-SK---D--G-K---------LQKAAVLGKLATR---------GEGKN--VTK-VLENC-

-----ANQ------QGE------SP------------------EDLAWNIF-RCGYDKKA

V---L---

--FDYMPAAPSGTEDN

DTK---

>Mcin-OBP_gene24_MCINX006010-RA_noname_PblastOBP

---------------------------MEIL-----------------------------
-------------------------------------RIL--AVL--SLGISVNTYQHKF
FSQSLDTE----------------------------PSLS--------IQYA-RDKTSDV
ITQECLMEM----------------YPRN----------------LYKY-PLRI------
-----------DRNDIPCIIHCVLKK--FGIM----------------------------
-SN---D--G-I---------INIRNYYKRVQAIHRY------DPRIL--ISD-VGDTC-
-----AQN------ING-MNLDHDV------------------CKKAKVFN-DCTQLYVI
SYRDP---

--DE------------

>Mcin-OBP_gene25a_MCINX005697-RA_PBP1

---------------------------MEGY-----------------------------
-------------------------------------FGL--CVV---------------
--LMSLTV----------------------------RTLA--------LDGE-MAELAKM
LRDNCVDET-----------------GVDA-A-------LI----DKVN-AGADL-----
---MG------DG-KLKCYIKCVMET--AGMM----------------------------
-SE------G-A---------VDVEAVIAVLPP----------ELLKH---AD-QLRTC-
-----GTQ------KGS------DD------------------CDTAFLTQ-SCWQKASK
S---D---

--YILI----------

>Mcin-OBP_gene25b_MCINX005697-RB_PBP1

---MAELAKM

LRDNCVDET-----------------GVDA-A-------LI----DKVN-AGADL-----

---MG------DG-KLKCYIKCVMET--AGMM----------------------------

-SE------G-A---------VDVEAVIAVLPP----------ELLKH---AD-QLRTC-

-----GTQ------KGS------DD------------------CDTAFLTQ-SCWQKASK

S---D---

--YILI----------

>Mcin-OBP_gene26_MCINX011317-RA_PBP3

---------------------------------------E--------LSEE-IKEIIQH
VHNECVGKT-----------------GVAE-E-------DI----ANCE-NGIF------
---KE------DV-KLKCYMFCLLEE--GSLV----------------------------
-DD---D--G-F---------VDYDMMVSLIPE----------QYTDR--VAK-MINAC-
-----KHV------DTL----DKDK------------------CQRAFDFH-KCSYSQDP
N---F---

--YFLF----------

>Mcin-OBP_gene27_MCINX001721-RA_PBP5like

---------------------------MLKS-----------------------------

-------------------------------------SIK--FLF---------------

CFVVLLKF------------------------C---ALAE--------ISED-LKQTFSE

MIMQCAKDV-----------------PLAE-A-------DI----EQLK-NRQM------

---PD------SE-DAKCFLPAPIKQ--QEWM----------------------------

-DD---E--G-M---------LSVEGVNSIAQKYYAD----DPERLEK--AKM-FTEAC-

-----KEV------NDV----NVSD--------------GNRGCERAALIF-KCSIEKGP

K---Y---

--DFH----F------

>Mcin-OBP_gene28_MCINX001718-RA_PBP5like

---------------------------MKSL-----------------------------
-------------------------------------CLL--VFV--VA----------L
AINLDNTR------------------------A---LSDD--------EKNT-IHSEILP
FIAECSKEY-----------------GVTE-E-------QL----KEAK-ESGK------
-----------VEAINPCLMGCIFKK--INVI----------------------------
-DD---K--G-L---------FNPDKAEEITKKFLTQ-----EEEQKK--ALD-IIKSC-
----TASV------NEK----DVSD-------------GAEGCERAKLLY-DCFIPFKG
Q---F---

--A-------------

>Mcin-OBP_gene29_MCINX001719-RA_PBP5like

---------------------------MTSL-----------------------------

-------------------------------------SLV--LFV--VG----------L

AINLRNVQ------------------------S---LSDE--------DKEN-IHAGIIP

YVAECSKEF-----------------GVTE-E-------QI----NEAK-KSGQ------

-----------VDGIDPCLMGCIFKK--VGII----------------------------

-DD---K--G-L---------FNPEKSEEITKKFLPN-----EDDQKK--ALE-VINSC-

-----KSV------NDE----DVSD--------------GEKGCDRAKLLH-ECFAPIRD

E---F---

--VKA----G------

>Mcin-OBP_gene30_MCINX001720-RA_PBP2like

---------------------------MYKT-----------------------------

-------------------------------------LSL--TCL------------FVM

NFVFFEIA------------------------A---MTAD--------QKAM-IHQHFEE

LGIECIKVH-----------------SITE-D-------DI----NNLR-TKKI------

---PS------GE-NAPCFLACMFKK--LGLL----------------------------

-DD---G--G-L---------LQKENALELAKKVFND-----DEELKL--IED-YLHSC-

-----SHI------NSE----SVSD--------------GNKGCERSMLAY-KCMIENAS

Q---F---

--GFD----L------
