# Supplementary material for: Mosaic Evolution of Molecular Pathways for Sex Pheromone Communication in a Butterfly
Source: Genes (Basel). 2022 Jul 31;13(8):1372. doi: 10.3390/genes13081372 (PMC9407440; doi:10.3390/genes13081372)
Supplement: Supplementary file 1 [file genes-13-01372-s001.zip › Supplementary File S3.pdf]

Supplementary File S3: Phylogenetic reconstructions for the delta desaturase gene family
Aligned amino acid sequences used for the delta-desaturase phylogenetic reconstruction:

Dpl_ctg10

----------------------------MEA---------SPAASDV------------------------EKLSK---------------LRE---
-----ANWPPVLFFIYIHILSFFGLWLLFVEA----KLM-
TILFLVFLVSVGLLGMTTGAHRLWAHRTYEANSALRIALMLFQTLAGIGPIYDWVQYHR
LHHAYFKTESDPYNPKNGFLHAHFFTRLQKLSPKMTALKDAIDMS--
DLQNDGIVMFQQRFYWVLYGIIFLLLPLNAPMEYWDESILSTFFIIGILRYAFILHGSWLIE
SGVCVWGLQEGEKYPPDSNTVFILNRTF---WPEYHYIYPQDYKSGEYG-
TYGSGCSTAFIRMFAVMGLAKDLRTLESATLQ-KALAEYARTKQ------------------------------
-----------PLDVCFKNALNNQVLPEEHYLKRD-----------------------------------

Hme_ctg10_HMEL002311-PA

----------------------------MAD---------SSS--------------------------MDLSRLSK---------------SRE-----
---ANWPAVLFYIHVHLLSLYGIWLIFFEA----KLL-
TILLLVVLTLTATLGVTVGSHRLWAHRTYKANLELRIFLMLCQTLAGHGPIYDWVQHHR
LHHAHFKTDSDLHDSRKGFLYAQFITRFSKLSPYQEELKNSIDMS--
DLEADSVVMFQRRYYWVLYAVIFLILPLNAPLEYWNDSVLSTVFVMGFVRYALVLHAS
WLLESGVCLWGLKEGEKYPPDSNSVFILNKTF---WPEYHYIYPQDYKSGEFG-TY----------
----

Ofu_Z/E14

----------------------------MAD---------IDATNHYDLQDKSEDDKPEDTEGEL---TIVGTDYSY---
------------THR--------IIWPIVMVYVVMHIGAITGLLLVLGGN----
VKIASIIWAVFYSLVATEGAHMGAHRCFSHRAFKAKPLLKVILLIMQTISGQHSTYIWCR
DHRQHHRYSDTDGDPHNSKRGMFYCHVGWLMTSRHPLCKKLRKTIDMS--
DLQQDPLVMFQYRYFRSLFFTFGFLLPVWVPMHFFQESFTNAVFVCFFLRYVYALHVTY
FINSLAHKYGTRPYDKTIQPVETWFVSLLSLGEGWHNYHHAYPWDYKAAEIG--
MPLNSTASLIRLCASLGLAYDLKSVDPETLN-KRIMNKGDGTY-----------------------
EVKYLQEHVTAIGPLHPLNPSYRGTCPDPEIKLKVRMKP-------------------------------------

Onu_Z/E14

----------------------------MAD---------IDATNPYDLQDKSEDDKPEDTEGEL---TIVGTDYSY---
------------THR--------IIWPIVMVYVVMHIGAITGLLLVLGGN----
VKIASIIWAVFYSLVATEGAHMGAHRCFSHRAFKAKPLLKVILLIMQTISGQHSTYIWCR
DHRQHHRYSDTDGDPHNSKRGMFYCHVGWLMTSRHPLCKKLRKTIDMS--
DLQQDPLVMFQYRYFRSLFFTFGFLLPVWVPMHFFQESFTNAVFVCFFLRYVYALHVTY
FINSLAHKYGTRPYDKTIQPVETWFVSLLSLGEGWHNYHHAYPWDYKAAEIG--
MPLNSTASLIRLCASLGLAYDLKSVDPETLN-KRIMNKGDGTY-----------------------
EVKYLQEHVTAIGPLHPLNPSYRGTCPDPEIKLKVRMKP-------------------------------------

Bmo_13_AGO45858

----------------------------MTA---------EVQNPKSVDENGNLNGTTKFDSFLI-STKYIGTDYSY--
-------------KHQ--------IVWKNAIGFLILHLLALWGVVLIVTGN----
IGLKTFLWTVLFMYAATIGITIGAHRFFTHKTFKGTPLLKVFLLCLQTVAGQNSTFIWARD
HRLHHRYSDTDADPHNSKRGFFFCHIGWLMMKKHPYVIELGRRIDMS--
DLQADKMIMFQKKYYYYLYFVVAFLIPVFVPIYFFNEHWLSSFLVCYCARYILQLNGTW
LVNSAAHLYGTRPYDRKLQPVESWFVSCISLGEGWHNYHHAFPWDYKAAEES--
MHFNSNATIIRFFERIGLAYDLKIASPDMVR-NRIVRSGDGTH-----------------H-----------------
------

VLGNDEAKSAVSAIGPLHPLNPTYNSTLKSPDAILKPEGLPLFHEKDVLINDLTRRNVAG

Bmo_4_AGO45856

----------------------------MTA----------VSENGNLNNTTKLNSLLINSK-------YIGTDYSY---------
------KHE--------IVWKNAIGYLILHLLCLWGVGLIVTEL---EGLK-
SCLWTILHMYAGSTGVTVGAHRLFTHKSFKATPLLKVFLLCLQTIAGQNSTFIWVRDHR
LHHRYSDTDADPHNSKRGFFFCHIGWLMMKKHPYVIELGRRIDMS--
DLQADKMIMFQKKYYYYLYFMVAFLIPVFVPIYFFNEHWLSSFLVCYCARYILQLNLTW
LVNSAAHMYGTRPYDRKLQPVESWFVSCISLGEGWHNYHHAFPWDYKAAEES--
MHFNCNATIIRFFERIGLAYDLKTASPDMIR-NRILRSGDGTH-----------------Y-----------------
------

VLGNDKAKSAVSAIGPLHPLNPTYNSTLKSPDAILKPEGLPLFHENDVLISDVIRGNIAE

Bmo_12_AGO45857

----------------------------MTAVSENENLNNTTKLNSLLINSK-----------------YIGTDYSY---------
------KHE--------IVWKNAIGFLILHLLGLWGVGLMVTGN----
IGLKSCLWTILYMYAGSTGVTVGAHRLFTHKSFKATPLLKVFLLCLQTIAGQNSTFIWAR
DHRLHHRYSDTDADPHNSKRGFFFCHIGWLMMKKHPYVIELGRTIDMS--
DLQADKMIMFQKKYYYYLYFVVAFLIPVFVSIYFFNEHWLSSFLVCYCARYILQLNGTW
LVNSAAHLYGTRPYDRKLQPVESWFVSCISLGEGWHNYHHAFPWDYKAAEES--
MHFNCNATIIRFFERIGMAYDLKTASPDMIR-NRILRSGDGTH-----------------H----------------
----

VLGNDEAKSAVSAIGPLHPLNPTYNSTLKSPDAILKPEGLPLFHENDVLINDIIRGNIAG

Dpl_ctg8

----------------------------MTV-----N---TDILHPNHYNTDVKISKQPNSNFDNDIIKNIGTDYSY-
--------------KHQ--------VVWKNAIGFFILHLLGIWGHLIVFTGG---IYWQ-
TFMWTSLLLFTSTEGITIGAHRLWSHRTFKATPLLKTILMIFQTLAGQNSIFTWCRDHRLH
HRYSDTDADPHNAKRGFFFSHIGWLLCKKHPYVKELGKRIDMS--
DLQNDWMIMAQKKYYYYLYLIFAVIIPVSVPYYYFGESLKNSLLVCYFARYVFQLNGT
WLVNSAAHLYGTRPYDKKLQPVESWFVSFISFGEGWHNYHHAFPWDYKAAELS--
MHFNQSAKFIRIFEKLGLAYDLKTASPEMVY-NFEMF---

Hme_ctg8

----------------------------MSP-------------NSVIESETQNGSSIIEDKNIIITKRIGTDYSY------------
---RHE--------IVWKNAIGFLILHILAVWGLFILFTGG----
ISLKTYLWSAFLMVCGSEGVTIGAHRLFSHRSFKATPLLKAVLLFFFTMSGQNSAYIWCR

DHRLHHRYSDTDADPHNSKRGFFFSHVGWLMCKKHPYVKSLGKRIDMS--
DLEADWMIMFQKKYYYYLYVISAIIIPVCIPYYYFGESFKNSLLFCYFTRYLAQLNGTWL
VNSAAHLYGTRPYDKKLQPVESWFVSFISLGEGWHNYHHTFPWDYKAAEW--
NMHFNSSATFIRWFEKLGLAYDLKTASSKMVR-RLI---

Dpl_ctg5

----------------------------MRQ---------KTKHSIADQLNEDIIDNKSDSDFDL----MKYEALEF-----
----------KPR--------IKWPDLLVQLCLHSTTVYGFYLILVNR----
VKLYTILFVFFTIYTSGFGITAGVHRLWSHRAYKANLPLRILLALLFTVTGQRDIYTWALD
HRVHHKYTETVADPHDVRRGFWFAHVGWLVLTPHPAVEDRRISLRKTSLDLLADPVVR
WQKIFFIPLFVLLNVFLPIAIPVYFWHESYINSFVLSFVTRFTITLNIAYSVNSFAHMWGNK
PYDRFIKSVENRIVSLAALGEGWHNYHHVFPWDYRTSELG---
MINISTTFIDAFAKIGWAYDLKVATNEMIR-NRARRNGDRSL-----------------R-----------------
------HLEEPDPSVSSE---

Bmo_6_AGO45854

MAPAQQNIEVCTEVMEPALELRLTSDGCKDEN-----
NVEHSKNNNTVKQNNILNEKTNNETDFDI----SKYEAMEF---------------TPR--------
IRWPDLVVQVSLHLVSIYGIFLIVTNE----
VKLLTTLFALATIYTSGFGITAGVHRLWSHRAYKARTPLRILLAFLFTITGQRDIYTWALD
HRVHHKYSETVADPHDVRRGFWFAHVGWLVLTPHPAVEDRRVALRKCSLDLIEDPVVR
IQQKIFIPLFLLLNILIPIWIPWYFWNESLVTSFVISFVLRFTTTLNIAFSVNSFAHLWGNKPY
DRFIKPAENSVVSLAALGEGWHNYHHVFPWDYRTSELG---
RINVSTNFIDFFAKIGWAYDLKAATSSMIE---IELRGAVMA---------------------------------------
--L--

Hme_ctg5

---------------------------MHLIN-----KLEHIENNNTVLAQEYSDKI--NDTDFNI----SNYEAVEF--
-------------RPQ--------IKWPDLIVQVMLHLVSIYGCFLVITSE----
VRFLTTLFAFATIYTSGFGITAGVHRLWSHRAYRARLPLRILLALLFTITGQKDIYTWALD
HRVHHKYSETVADPHDVRRGFWFAHVGWLVLTPHPAVENRRKALKKTSIDLINDPVVR
LQRRFFIPLFIILNVLVPVGVPVYFWEEALLYSFVVSFVTRFTITLNIAFCVNSFAHMWGN
KPYDRFIKPVENSVVSLAALGEGWHNYHHVFPWDYRTSELG---
RLNLSTKFIDTFARIGWAYDCEFVV---FLSF----

Ape_E6

----------------------------MK----------IIKFDEILR--------------------RFERVFAF---------------DND--
------IKWGSVFVIFGYHALAVYWCYNYSLPV----KWQ-
TVIFAYCTYVLSGMGITLGVHRYWSHRCFKATKPLQIILFLMFALTGQTSIRIWVRNHRL
HHKASDTPGDPHNATRGLFYSHIGWLMMKKTDQLIEQGKKIDMR--
DIDSDPLLRWYDRNVFMVNLMAAFVLPTMVNIWCLGESWRCAVAWQCFVRYLGVLH
AQMTINSLAHKFGNRPFNSRIKPAENMFVSYITFGEGFHNYHHVFPYDYRTT--
GDYFILDYGKILLKFFEKIGWAYNLRKASPETIA-SAVGR---
------LNCAESDDLLKS---

Bmo_15_XP_004933819

MIIVSGAGITCGVHRLWTHRSYKAKAPMKLLLMACFCSSGQNSIYNWVRDHRLHHKFA
DTDADPHNVKRGFFFSHIGWLMVKKNESVLTKGKLIDMS--
DIESDSHLMFEHKYHNQLTILFCFLIPTLMNIFLIGEDWKCAVAWQCFIRYLYVLHCELTV
NSLAHMYGYKPYNVNIEASENILVSVLTYGEGYHNFHHVFPFDFRAAET--
MDFFSLSTKIISTFEKIGWTYDLKQASPEMIEAARDKLGGDGSK----------------------------------
---------AHN---

Bmo_14_AGO45859

--MERDKKVMEKKITPER--SLSEIFR----TFEKSLGF-----------
----QND--------IKWPSFIIITLYHVIGAYWCYNFAFPV----KWQ-
TVVFAAIMYVATGFGITGGAHRLWTHKSYKAKLPLKLFLLLCFSAAGQNSLYQWVRDH
RIHHKFSDTDADPHNANRGLFFSHIGWLMMKKNNEVILRGKQIDMS--
DIENDPFLRFYDKNFNSLKLVFCYIPPTMLGILLWNEEWKCAVAWQCFIRFLGMFHSELT
VNSLAHTFGYKPYNKNIVPAENRFVSICTLGEGWHNYHHAFPFDYKAAEH--
FDFFNFGTKFIKLFEKIGWAYDLKEATPEMIN-AIAKKLGDGTP-----------------V--------------
---------HFPILVTKTN---

Dpl_ctg6

-----------------------------MEN---------INLKHLPPISETFR---------------AFEKSLGF---------------
KND--------IKWTSAILLTLYHIAAVYWCYHYALPV----KMQ-
TVIFAAIMYVATGFGITGGAHRLWTHKSYKATLPLKLFLLMCFASAGQNSLEQWVRDH
RLHHKHSDTNADPHNANRGLFFSHIGWLMMKKNKEVIIRGKQMDMT--
DIHEDPILIFYNKHFAYFRLLFCFVLPFMINVYILKESWQCTIAWQWFIRFLSMFHSELTVN
SLAHAYGYRPYNKNIVPRENRFVATCTLGEGWHNYHHAFPFDYKAAEH--
FDLFNWGTLFIQAFEKLGLAYGLRMATPEMIS-NLSKRLGENAQ---------------------------------
----

Hme_ctg6

-----------------------------MEK---------INKRSTLSLSEIFR---------------SFEKNLGF---------------
KND--------IKWTTAILIVLYHVAAVYWCSLYTFPV----KWQ-
TVVLAAIFYVITGFGITGGAHRLWTHKSYKATLPLKLFLLMCFSCAGQNSLEQWVRDHR
VHHKFSDTDADPHNSNRGLFFSHIGWLMMKKNAQVRNSGKLIDMT--
DITNDPLLKFYNKYFSYFKLFFCYVLPVSINVYLFGESWQCAVAWQWFIRFLSMFHSELT
VNSLAHAYGYKPYNKNLIPTENRFVATCTLGEGWHNYHHAFPFDYKAAEH--
FDMFNWCTRFIQGFEKLGWAYDLREATPEMIN-SIAQRLGDGTP-----------------V-------------
-----------HFPLKSQNEL---

Hme_ctg7

--MGF---------------LTP--------
IRWTMVITITLFHIITLVGFFNSVFTEGRLPMWK-
TIYFATFVGVFGGFGVTGGVHRYWTHRSYKAKLPLQIILMSGFAISGQNNIFNWVRDHR
VHHKLSETSADPHNAHRGFFFSHVGWLMMKKHPHVISEGNKIDMT--

DILNDTVVQFHTRYFVWIKLLLCFLLPTAIPIYFWGESVVASLIT-
TAVRYMLGLHMTWAVNSFAHIWGNKPYDTNITPVENWKVSFVAMGEGWHNYHHTFP
WDYKAAELA--YFINITTILIDLFATIGWAYDLKKAAPSLIK-AVVKSRGRQTK----------------
---------------------------KMS--

Bmo_7

------------------------------MAP-----
LNIVDTPYSDLTKLEDSIENKPNNQANKPNNFEKTNTVDPYDEVKLNEPIFLAPIRKWEK
RMGFVTPIRWTNAILITTFHVVTVVWGFYTLFYEKQKPMWQ-
SVIFGIFMGQLAGFGVTAGAHRYWCHRSYKAKLPLQLILALCYSAAGQNTIYEWVRDHR
VHHKFSETSADPHDANRGFFFSHIGWLMMKKHPDVVKQGSKIDMS--
DIIHDPVVIFCTKYFILFKITFCWIIPSALPVYAWGELWNIAILSQAFWRYMLSLHFTWSVN
SFAHLWGNKPYDRYIMPCENKGVSIVAMGEGWHNYHHTFPWDYKAAELG--
VPNNTTTFLLDLFARIGWAYDLKQASPALVR-AVAKQRGECKE---------------------------------
----

Dpl_ctg7

---MGF----------------ITP--------
IRWTNAILISSFHIMAVITFLYTFSSG-MVPTLE-
TVFFGFLCGWIGGFGVTAGAHRYWTHRAYKAKIPLQIILIIAYSVAGQNSIPNWVRDHRV
HHKMSETSADPHDANRGFFFSHVGWLMMKKHPDVVKEGKKIDMS--
DILEDPLVRFHTKYFNTFKILFCFVIPILIPTYMWKETLFISVTA-
SLVRYMSNLNCTWAVNSVAHIWGQKPYDINITPVENWKVSIVAMGEGWHNYHHTFPW
DYKASELA--YFINVTTFLIDIFGKIGWAYDFKVASPALVK-AVIRKRGPRF--------------------
----

Bmo_3_AGO45851

------------------------------MPP-----QV--DPIPSGVVFESDTQTD--DLGLDKD--VSVLKKATPK--
-------------KHE--------LVWFNIIWFLFLHISSLYGLYLVFTSA----KWQ-
TNVFAFGVHLMCAIGIGAGSHRLWTHRSFKARTPLRIVLMIWQTMGFQDCIFEWARDHR
THHKYADTDADPHNAERGLFFSHMGWLCCKKSPEVIEGGRRIDLS--
DLYADPVVMFQKKHYMKMMPILCFVLPTVIPVYFWDETWLNAFFIPTILRYTCGINVVW
SVNSFAHTFGYRPYDKSLNPRENIGVWMICV-
EGFHNYHHTFPWDYRATEHPLYNMLTPTIVFIDAMAMIGQAYDLKTVPHDIIK-
QRTHRTGDGTH----------------Q-----------------------
LWGWDDPEFNEKLKEKYSRVSYAERKCG---------------------------------

Dpl_ctg4

------------------------------MPP-----QA--DPTPSGVLFETDTQTP--DLGLDKD--VSILKKARPR---
------------KYE--------YVWFNIMWFLFLHVASAYGIYLVFTSA----KWQ-
TNVFAFLVHLCCAIGIGAGSHRLWTHRSFKAKTPLRILLMIWQTMGFQDCIFEWARDHR
THHKYADTDADPHNAERGLFYSHMGWLCVKKSPEVLEGGRRIELK--
DLYDDPVVMFQKRHYLKMMPILCFVFPTIIPVYFWNETWLNAFLIPTILRYTCGINIVWS
VNSFAHVFGYRPYDKSLNPRENIAVWMFCV-
EGFHNYHHTFPWDYRATEHPILNMLTPTIMFIDLMAKLGQAYDLKTVTPEIIK-

QRAQRTGDGTH-----------------H-----------------------
LWGWDDPEFTEKMKLRYGITNSERKTA---------------------------------

Hme_ctg4

-----------------------------MPP-----LV--DPTPSGVLFESDTQTA--DLGLDKD--VTGLKKAAPR--
-------------KYE--------YVWFNIFWFLFLHASSAYGIYLGLTSA----KWQ-
TNVFAFAVHLMCAIGIGAGSHRLWTHRSYKARTPLRTLLMIWQTMGFQDCIFEWARDH
RTHHKYADTDADPHNAERGLFYSHMGWLCCKKSPEVIEGGRRIDVS--
DLYDDPVVMFQKRHYMKMMPVLCFVLPTIIPVYFWGETWLNAFFIPTILRYTIGINVVW
SVNSFAHKYGYRPYDKSLNPRENIAVWMVCV-
EGFHNYHHTFPWDYRATEHPILNMLTPTIMFIEGMAKIGQAYDLKAVSPDIIK-
QRSQRTGDGTH-----------------H-----------------------
LWGWDDPEFTEKLKSRYGVVNKPERKCA--------------------------------

Dpl_ctg9

-----------------------------MTA---------SAFLHDPMHQKN---------IGELSQLDPPRQRRDY--------
-------EWQ--------IVWRNVLAFVYLHMATLYGIYLILIGK----
TKILTLIFAVTISAMSAMGVTAGAHRLWAHRAYKARWPLRIFLAVMQTMAFQNHIYEW
VRDHRVHHKFTETDADPHNAKRGFFFSHMGWLMVRKHKAVFEKGASVDMS--
DLEKDPIVMFQKKTYLVLMPLLCFVVPAWLPVVLWNENPWVSWYVASMLRYTISLHFT
WLVNSAAHIWGNRPYDQYIGATDNKAVAICAFGEGWHNYHHVFPWDYKAAELG-
NYSTNLSTALIDFAAKYGLAYDLKTVSIEMIR-KRVSRTGDGSH-----------------
PLSSKKESLNQH----DHHHPENPVWGWGDEDMTEEEKKLAEIAHKLN------------------------
----

Hme_ctg9_HMEL015726-PA

-----------------------------MTA-----ST--FLRANGSYAKADCNTP------SLI----TQGKRRDY--------
-------EWQ--------IVWRNVLAFVYLHVAAAYAFYLIFSGR----
IWTSTVVFALLFAAMSALGITAGAHRLWAHRAYKARWPLRVFLALMQTMAFQNHIYE
WVRDHRVHHKYTETDADPHNARRGFFFSHMGWLMVRKHKEVFEKGGSVDMS--
DLEKDPIVMFQKRTYLFVMPLLCFILPTWIPVALWNESPWNSWYVAAIMRYTVSLHFTW
LVNSAAHIWGNRPYDKYIGATDNKTVAICAFGEGWHNYHHVFPWDYKAAELG-
NYSTNFSTALIDVAAKYGLAYDLKTVSAEMIR-KRVARTGDGSH-----------------PL--
DKKSKQDD----GHHHPENPIWGWDDKDMTEDEKQLAEIAHKLS-------------------------------
-----

Has

-----------------------------MAA---------MSSTSLLLANTMLSSK--
LQDHDDLRYAEPRKPNRDY---------------EWQ--------
VVWRNVLAFVYLHVSAVYGLYLMLTGK----
VKSYTILFGALFAIMSGMGVTAGARRLWAHRSYKARWPLRVFLALMQTMAFQNHIYE
WVRDHRVHHKFTETDADPHNAKRGFFFSHIGWLMVRKHKDVFEKGATVDMS--
DLEQDPIVMFQKKTYLVVMPILCFVIPAWIPVHFWDENPWTSWYTAAITRYTIALHFTW
LVNSAAHIWGNRPYDKNIGATDNKMVAICAFGEGWHNYHHVFPWDYKAAELG-
DYSTNLSTALIDFAAKHGYAYDLKTVSADMIR-KRVNRTGDGSH-----------------P-----

WTK--GKVEGDHYHPENPVWGWEDTDMTEEEKQFAEIVHRKTE-------------------------------
----

Dpu

-----------------------------MEF---------IMAGSKIIAKNVDHDE--DPRIVSA----PQMKNRDY------
---------EWH--------IVWRNVLAFIYLHVAAAYGLYLMFTGK----
VNAFTILFAFSFAAMSAMGVTAGAHRLWAHRAYKARWPLRLYLALMQTMAFQNHIYE
WVRDHRVHHKFTETDADPHNARRGFFFSHMGWLMVRKHKDVFEKGATVDMS--
DLEKDPIVMFQKKTYLVVMPLLCFLIPSYIPVWLWNEDPWSSWYVASITRYTLTLHFTW
LVNSAAHIWGTRPYDKNIGATDNVAVAIGAFGEGWHNYHHVFPWDYKAAELG-
NYRTNLSTALIDIAAKYGLAYDLKTVSKEMIE-QRVSRTGDGSH-----------------P-----
WAQQTEDCSDEHHHPENPVWGWDDTDMPEEDKQLAKIVHRKTE----------------------------
----

Lca_NF

-----------------------------MAPNILGSSLFLADTTLGKNVDDDHSSN---FSASDN----
YSGKRRDY---------------EWQ--------IVWRNVFGFIYLHTGMLYGFYLIFTGQ----
VKFFTVLFAVLFAILSAMGVTAGAHRLWAHRAYKARWPLRLILAFLQTMAFQNHLYEW
VRDHRVHHKFTETDADPHNAKRGFFFSHIGWLMIRKHKDVFEKGASIDMS--
DLEADPIVMFQKKTYLVVMPILCFVLPAYIPIVWWGEVPWTSWYVATILRFTVSLNFTW
LVNSAAHIWGNRPYDENIGATDNKTVAILAFGEGWHNYHHVFPWDYKAAELG-
NYSTNMSTAIIDYAAKHGWAYDLKTVSVDMIR-KRVARTGDGTH-----------------
PSNDDTKKKLEE----DHHHPDNPVWGWDDTDLKDEDRKFAEIAHPKEQ----------------------
----

Cpa_Z9

-----------------------------MTT-----GT--SLALAGTYFAAKNKKVDDDITTPLT---RISGKRRDY-
--------------EWQ--------IVWRNVLAFVYLHVGLLYGFYLILTGR--VKLW--
TPVFAVTFTVLSALGVTAGAHRLWAHRAYKARWPLRLILALLQTMAFQNHIYEWVRDH
RVHHKFTETDADPHNAKRGFFFSHIGWLMVRKHKEVFEKGASIDMS--
DLEKDPIVMLQKKTYLVVMPILCFLLPSWIPVYFWGEDPWTSWYVASIWRYTMSLNFT
WLVNSAAHIWGNKPFDKNIGATDNLTVAICAIGEGWHNYHHVFPWDYKAAELG-
NYRTNISTAIIDLAAKYGWAYDLKTVSTQMIL-NRVTRTGDGSH-----------------
PSVSGDSKQLEETEHDHHHHPENPVFGWTDADISEEDRMLVEITHKIDENDD---------------
----

Obr_tdes

-----------------------------MAP-----N---TLKEDVMIVNEE--------TSEKL----VV-GAAPR-----------
----KYK--------VVWFIVAYATYVHIAAVYGLYLAVTSA----MWP-
TIALTFINNILSILGLTAGVHRLWTHKSYKAKLPLQIFLMLCHTASNTFTSISWIRDHILHH
KYTDTDADPHNSTRGFFFSHIGWAMVKKQPEARAKGKSIDLS--
ELYANPVLRFQQKNAVWLTLLVAYIIPSLVPL-IWNETFTVAYHM-
NLLRVTVVFNTFLLINSVAHMWGTRPYDETILPAQNKTVSFFTLGEGFHNYHHVFPHDY
RTAELG-DNFLNLTTKFIDFCAWMGQAYDRRYVPDDVIA-ARMKRTGETNE----------------
-------------------------------KNT--

Bmo_11_ABD36148

-----------------------------MAQ---------NQTYNGLIVTEIEDSS---LEHDSV----KPSVVGPR--------
-------EYE--------IVYKNIFIHIYLHVTMFYGLYLCCTTA----KWP-
SVGFAYLCYVAATIGVTAGAHRLWSHKSYKARLPLQILLMVFLSLANQRAT-
HWVRDHRVHHKYSDTDADPHNASRGFFYSHIGWLFVRKHPEVKKKGKLIDLS--
DLFDNPALMFQHRYSKTFIPIVCFGLPTILPVILWDELLVVAWNL-
TIMRYVINFHVFFLVNSVAHIWGNKPYDKTIKSAENNLVAFATLGEGYHNYHHVFPWD
YRCTELG-RTWLNYTKLFIDLCAKVGLAYDLKVVSDDVIL-RRVKRTGDRSH---------------
--------------------------FIEN--

Bmo_8_AGO45853

-------------------------MSRFQAP-----S---NEDRDDVFSEEE-------TRLEKL----IAPQAEPR--------
-------EFK--------VVYRNIIVLLYYHMAGIYGLYLGLIAA----KWA-
TVISAFITYCISVIGVTAGSHRLWSHKSYKATKPLQLFLMLCQSVSNQHSVAYWVMVHR
LHHKYSDSDADPHNATRGLFYSHMGWLMVRKHPEIEKRGKLLDLS--
DIYGNPYLKFQDTHYYWFLPLMSFLLPTIIPMYFWGETCSVAWNV-
NMCRYVMNLNAIFLVNSVAHMWGYKPYDKNIAPSQNLSVSVLSLGEGFHNYHHVFPW
DYKCDELS-SFRTNPTTVFIELCAKLGLAYDLRSASDDLIE-GRAKRTGDGSY----------------
-------------------------LTLKKSR---

Dpu_Z/E11_1

-----------------------------MAP-----N---ITNEEIIVSETDHDQS------------KRPQAEPR-------------
-QYH--------IVYRNIAVFLYYHIAGIYGLYLCFGNA----MWT-
TIFWNVFTFIIAGLGVTAGAHRLWSHKAYKATRPLQIFLVLAQSVANQHSVAHWARDHR
LHHKYSETDADPHNSTRGFFYSHVGWLMVRKHPEVTRRGKTVDMS--
DIYNNPYLKLQDKHSYWFIPLLSFFIPTYIPT-LWGESLTNAWHI-
NMFRYILNLNATFCVNSVAHLWGQKPYDKHIAPSQSPKVNVVTLGEGFHNYHHCFPWD
YRSDELG--LTFNFTTGFIDICEKLGLAYDLRSASEEVIA-ARAERTGDGSR---------------------
--------------------IKSL--

Pex_tdes

-----------------------------MVP-----D---VLREAERLQDDA-----------KL----VAPQAEPW-----------
----KFQ--------LSYTNVVIFGYLHISGIYGLLFCVSKA----HWA-
TIFFSFILLVASVIGVTAGAHRLWSHRSYKANLPLQIILMLFHSLSGQYTAFNWARDHRLH
HKYSDTDADPHNATRGFFYSHIGWLLVVKHPEVRKRGEAIDLS--
DLLRNPVLTFQRKNVVLILALLCYIMPTAVPMYFWGETFHNAWHI-
MALRFVLCLNFISLINSAAHTFGNKPYDKSIMPTQNMSVTLATLGEGFHNYHHVFPFDYR
AAELG-NNTFNLTTKFIDFFAMIGWATALKTVGHESIA-RRAQRTGDGSL---------------------
--------------------TWKSDCEVVPGGLKSG---

Lca_Z9

-----------------------------MPP-----N---VTEANGVLFENDVQTP--DMGLEVA----PVQKADER---
------------KIQ--------LVWRNIIAFAYLHLAAVYGAYLFFTSA----KWQ-
TDIFAYILYVMSGLGITAGAHRLWAHKSYKAKWPLRLILVAFNTLAFQDSAIDWARDHR
MHHKYSETDADPHNATRGFFFSHIGWLLCRKHPELKRKGQGLDLS--

DLYADPILRFQKKYYLLLMPLACFVLPTIIPVYLWGESWKNALFVAAMFRYTFILNVTW
LVNSAAHKWGGKPYDKNIQPAQNISVAIFALGEGFHNYHHTFPWDYKTAELG-
NNRLNFTTSFINFFASFGWAYDLKTVSDEIIQ-QRVKRTGDGSH----------------H-------------
---------LWGWGDQDIPAEEAQAALRINRKDD----------------------------------

Has_Z9

----------------------------MAP-----NI--SEDVNGVLFESDAATP--DLALSTP----PVQKADNR----
-----------PKQ--------LVWRNILLFAYLHLAAQYGGYLFLFSA----KWQ-
TDIFAYILYVISGLGITAGAHRLWAHKSYKAKWPLRVILVIFNTVAFQDAAMDWARDHR
MHHKYSETDADPHNATRGFFFSHIGWLLVRKHPDLKEKGKGLDMS--
DLLADPILRFQKKYYLILMPLACFVMPTVIPVYFWGETWTNAFFVAAMFRYAFILNVTW
LVNSAAHKWGDKPYDKSIKPSENLSVAMFALGEGFHNYHHTFPWDYKTAELG-
NNKLNFTTTFINFFAKIGWAYDLKTVSDDIVK-NRVKRTGDGSH----------------H-----------
-----------LWGWGDENQSKEEIDAAIRINPKDD----------------------------------

Sli_Z9

----------------------------MAP-----NI--SEDVNGVLFESDAATP--DLALARP----PVQKADNK---
------------PKQ--------LVWRNIILFAYLHLAALYGGYLFLFSA----KWQ-
TDIFAYILYVISGLGITAGAHRLWAHKSYKAKWPLKVILIIFNTVAFQDAAMDWARDHR
MHHKYSETDADPHNATRGFFFSHIGWLLVRKHPDLKEKGKGLDMS--
DLLADPVLRFQKKYYLLLMPLACFVMPTMIPVYLWGETWTNAFFVAAMFRYAFNLNV
TWLVNSAAHKWGDKPYDKSIKPSENMSVAMFALGEGFHNYHHTFPWDYKTAELG-
NNKLNFTTAFINFFAKIGWAYDMKTVSEDIVK-NRVKRTGDGSH----------------H----------
------------LWGWGDENQPKEEIEAATRINPKDD----------------------------------

Poc_Z9

----------------------------MAP-----N---VTEENGVLFESDAVTP--DLAMARA----PVQQADST---
------------PRV--------YVWRNIIAFAYLHLAAVYGFYLLLVSA----KWQ-
TDIFAYLLYVASGLGITAGAHRLWAHKSYKAKWPLRVILTLFNTIAFQDSAIDWARDHR
MHHKYSETDADPHNATRGFFFSHIGWLLVRKHPELKRKGKGLDLS--
DLYADPILRFQKKYYLLLMPLACFVLPTVIPVYLWSETWTNAFFVAALFRYTFILNVTWL
VNSAAHKWGDKPYDKDIKPSENMSVSLFAFGEGFHNYHHTFPWDYKTAELG-
NHRLNFTTKFINFFAKLGWAYDMKTVPDTIVQ-QRVKRTGDGSH----------------H----------
------------LWGWGDKDHAQEEINAAIRINPKDD----------------------------------

Epo_Z9

----------------------------MAP-----N---VTEENGVLFESDAATP--DLALARE----PVQQADSS----
-----------PRV--------YVWRNIILFAYLHIAAVYGGYLFLFSA----KWQ-
TDIFAYLLYVASGLGITAGAHRLWAHKSYKAKWPLRLILTIFNTTAFQDSAIDWARDHR
MHHKYSETDADPHNATRGFFFSHIGWLLVRKHPELKRKGKGLDLS--
DLYADPILRFQKKYYLILMPLACFILPTVIPVYLWNETWSNAFFVAALFRYTFILNVTWL
VNSAAHKWGDKPYDKSIKPSENLSVSLFAFGEGFHNYHHTFPWDYKTAELG-
NHRLNFTTKFINFFAKIGWAYDMKTVSHEIVQ-QRVKRTGDGSH----------------H-----------
-----------LWGWGDKDHAQEEIDAAIRINPKDD----------------------------------

Ave_Z9

-----------------------------MAP---------NVTENGVLFESDAATP--DLALGTA----PVEQADDS-----
----------PRV--------YVWRNIILFAYLHLAAIYGGYLFLFSA----KWQ-
TDIFAYFLYVASGLGITAGAHRLWAHKSYKAKWPLRVILTTFNTIAFQDSAIDWARDHR
MHHKYSETDADPHNATRGFFFSHIGWLLVRKHPELKRKGKGLDLS--
DLYADPILRFQKKYYLLLMPLACFILPTVIPVYLWNETWTNAFFVAALFRYTFILNVTWL
VNSAAHKWGDKPYDKSIKPSENMSVSLFAFGEGFHNYHHTFPWDYKTAELG-
NHRLNFTTKFINFFAKIGWAYDMKTVSNEIVQ-QRVKRTGDGSH-----------------H-----------
-----------LWGWGDKDHAQEEINAAIRIHPKDD----------------------------------

Cro_Z9

-----------------------------MAP-----N---VTEENGVLFESDAATP--DLALART----PVEQADDS----
-----------PRI--------YVWRNIILFAYLHLAAIYGGYLFLFSA----KWQ-
TDIFAYFLYVASGLGITAGAHRLWAHKSYKAKWPLRLILTIFNTIAFQDSAIDWARDHRM
HHKYSETDADPHNATRGFFFSHIGWLLVRKHPELKRKGKGLDLS--
DLYSDPILRFQKKYYMILMPLACFILPTVIPVYMWNETWSNAFFVAALFRYTFILNVTWL
VNSAAHKWGDKPYDKSIKPSENMSVSLFAFGEGFHNYHHTFPWDYKTAELG-
NHRLNFTTKFINFFAKIGWAYDMKTVSQEIVQ-QRVKRTGDGSH-----------------H-----------
-----------LWGWGDKDHAQEEINAAIRINPKDD----------------------------------

Dpl_ctg3

-----------------------------MAP-----N---SSDANGVLFESDAATP--DLALPNT----PIQQADSY-----
----------PRK--------YVWRNIIAFAYLHIAALYGGYLFLFSA----KWQ-
TNIFAYILYVMSGLGITAGAHRLWAHKSYKAKWPLRVILVIFNTLAFQDSAIDWARDHR
MHHKYSETDADPHNATRGFFFSHIGWLLVRKHPELKRKGKGLDLS--
DLYADPILRFQKKYYLLLMPISCFILPTIIPVYLWGETWTNGYFVAAMFRYAFILNVTWL
VNSAAHKWGDKPYDKNIQPSENMSVSLFALGEGFHNYHHTFPWDYKTAELG-
NNRLNFTTTFINFFAKIGWAYDLKTVSDEIVK-QRVNRTGDGSH-----------------H------------
----------LWGWGDKDHSKEEIRAAIRINPTDDKEE-------------------------------

Hme_ctg3

-----------------------------MAP-----N---SGDANGVLFESDAATP--DMALPIT----PIQQADQY-----
----------PRK--------LVWRNIIAFAYLHLAALYGGYLFLFSA----KWQ-
TDIFAYILYVMSGLGITAGAHRLWAHKSYKAKWPLRVILIIFNTLAFQDSAIDWARDHR
MHHKYSETDADPHNATRGFFFSHIGWLLVRKHPELKKKGKGLDLS--
DLYADPILRFQKKYYLILMPIACFILPTLIPVYLWGESYINAYFVAAMFRYAFILNVTWLV
NSAAHKWGDKPYDKNIQPSENISVSVFALGEGFHNYHHTFPWDYKTAELG-
NNRLNFTTNFINFFAKIGWAYDLKTVSDEIVQ-KRVKRTGDGTH-----------------H-----------
-----------LWGWGDKDHAADEKNAAIRINPKEDKDD-------------------------------

Bmo_1_AFK13829

-----------------------------MAP-----N---VKDANGVLFENDADTP--DLGLSST----PVQQADNY---
------------PKK--------LVWRNIILFTYLHIAALYGGYLFLFHA----KWQ-
TDLFAYILYVMSGLGITAGAHRLWAHKSYKAKWPLRVILIIFNSLAFQDSALDWARDHR
MHHKYSETDADPHNATRGFFFSHIGWLLVRKHPELKRKGKGLDLS--
DLYADPILRFQKKYYLILMPIACFILPTVIPVYFWNESWINAFFVATLFRYTFILNVTWLV
NSAAHKWGGKPYDKNIKPSENISVSVFALGEGFHNYHHTFPWDYKTAELG-

NNRLNFTTNFINFFAKIGWAYDLKTVSDEIIK-NRVNRTGDGSH-----------------Y--------------------------LWGWGDKDLDKEEIKQAIRINPKDN----------------------------------

Mse_Z9

-----------------------------MPP-----Y---TSDANGVLFESDAATP--DLALSST----PVQQADNR---------------PKQ--------LVWRNIILFAYLHIAALYGGYLFLVHA----KWQ-TDIFAYLLYVMSGLGITAGAHRLWAHKSYKAKWPLRLILVVFNTMAFQDSAIDWARDHRMHHKYSETDADPHNATRGFFFSHIGWLLVRKHPELKRKGKGLDLS--DLYADPILRFQKKYYLILMPITCFVMPTVIPVYLWGESWVNAFFVAALFRYAFILNVTWLVNSAAHKWGDKPYDKSIKPSENISVSMFALGEGFHNYHHTFPWDYKTAELG-NNRLNFTTNFINFFAKIGWAYDLKTVSDEIIQ-QRVQRTGDGSH-----------------H--------------------------LWGWGDKDHDKEEVNAAIRINPKDD----------------------------------

Onu_Z9

-----------------------------MAP-----NIKDGADLNGVLFEDDASTP--DYALATA----PVQKADNY---------------PRK--------LVWRNIILFAYLHLAAVYGAYLFLFSA----KWQ-TDIFAYILYVISGLGITAGAHRLWAHKSYKAKWPLRLILIIFNTVSFQDSALDWSRDHRMHHKYSETDADPHNATRGFFFSHIGWLLVRKHPELKRKGKGLDLS--DLYADPILRFQKKYYLLLMPLGCFIMPTVVPVYFWGETWTNAFFVAALFRYTFILNVTWLVNSAAHKWGHKPYDSSIKPSENLSVSLFALGEGFHNYHHTFPWDYKTAELG-NNRLNFTTNFINFFAKIGWAYDLKTVSDEIIQ-NRVKRTGDGSH-----------------H--------------------------LWGWGDKDQPKEEVNAAIRINPKDE----------------------------------

Dpu_Z/E9

-----------------------------MAP-----K---EADVNGVLFESDATTP--DMALPTT----PVQQADNY---------------PKK--------LVWRNILLFAYLHLAALYGGYLFLFSA----KWQ-TDIFAYILYIMSGLGITAGAHRLWAHKSYKAKWPLRLILVLFNTLAFQDSAIDWARDHRMHHKYSETDADPHNATRGFFFSHIGWLLVRKHPELKKKGKGLDIS--DLYADPILRFQKKYYLLLMPLGCFILPTVIPVYLWNETWSNAFLVAALFRYAVILNVTWLVNSAAHKWGDKPYDKSIKPSENLSVALFALGEGFHNYHHTFPWDYKTAELG-NNRLNFTTTFINFFAKIGWAYDMKTVSDEIIQ-NRVKRTGDGSH-----------------H----------------------LWGWGDKDHSKEEINAAIRINPKDD----------------------------------

Onu_Z9_b

-----------------------------MPP-----QG--AERDSFVLFESDAKDP----DVITH----MAPQAEKR---------------QTQ--------IVWRNVAIFTYLHLGFLYGAYLMLTTV----MWK-TRLFCLILYVCSGLGITAGAHRLWAHKSYKAKLPLRIILTLFNTLAFQDAVVDWARDHRMHHKYSETDADPHNATRGFFFSHVGWLLVRKHPQIKAKGHTIDMS--DLRADPVLRFQKKYYMYLMPLICFIMPSVVPA-LWGETVWNGYFTCAVFRYVAVLNGTWLVNSWAHLWGDKPYDRHINPVETKVVSVAAIGEGFHNYHHTFPWDYKAAELG-NYTFNITKFFIDTMATIGWAYDLKTVSTDVIQ-KRITRTGDGSH-----------------P-----------------------VWGWGDKNIPEEDKKVTTVINPEKEE----------------------------------

Bmo_2_AF182406

----------------------------MPP-----QR--KQEASWVLYEADANNL--PEDAPPH----VPPSAEKR---
------------PWK--------IVWRNVILFFILHVGGVYGGYLFLFKA----MWR-
TSIFAIFLYLCSGLGITAGAHRLWAHKSYKARLPLRILLTIFNTIAFQDAVVDWARDHRM
HHKYSETDADPHNATRGFFFSHIGWLLLRKHPEIKAKGHTVDVN--
ELRNDPILRFQKKYYQILMPLACFIMPTYVPT-
LWGETVWNSFYVCAIFRYVYVLNITWLVNSAAHMWGSKPYDKNINPVETRPVSLVVLG
EGFHNYHHTFPWDYKTAELG-DYSLNLSKLFIDFMAKIDWAYDLKTVSTDVIQ-
KRTKRTGDGSH-----------------P----------------------
VWGYDVGEVATEDKTDTTNLVNSKVL---------------------------------

Epo_Z9_b

----------------------------MPP-----QG--QPPAAWVLEESDATTD--DKDVAVA----VPPSAEKR--
-------------KLS--------IVWRNVILFVLLHTGAVYGGYLFFTKA----MWA-
TKFFAFFLYLCSGLGITAGAHRLWAHKSYKARLPLRILLTLFNTIAFQDSVLDWARDHR
MHHKYSETDADPHNATRGFFFSHVGWLLVRKHPQIKAKGHTIDMS--
DLCSDPVLRFQKKYYLTLMPLFCFILPTYIPT-
LWGESLWNAYFVAAIFRYCYVLNVTWLVNSAAHKWGDRPYDKNINPVETKPVSLVVF
GEGFHNYHHTFPWDYKTAELG-GYSLNISKLFIDTMAKIGWAYDLKSVSPDIVE-
KRVKRTGDGSH-----------------E----------------------
VWGWDDKDVPAEQKAAATIINPEKTE---------------------------------

Tni_Z9

----------------------------MPP-----QG--QTGGSWVLYETDAVNT--DTDAPVI----VPPSAEKR---
------------EWK--------IVWRNVILMGMLHIGGVYGAYLFLTKA----MWL-
TDLFAFFLYLCSGLGITAGAHRLWAHKSYKARLPLRLLLTLFNTLAFQDAVIDWARDHR
MHHKYSETDADPHNATRGFFFSHVGWLLVRKHPQIKAKGHTIDLS--
DLKSDPILRFQKKYYLTLMPLICFILPSYIPT-
LWGESAFNAFFVCSIFRYVYVLNVTWLVNSAAHLWGSKPYDKNINPVETRPVSLVVLGE
GFHNYHHTFPWDYKTAELG-DYSLNFTKMFIDFMASIGWAYDLKTVSTDVIQ-
KRVKRTGDGSH-----------------A----------------------
VWGWDDHEVHQEDKKLAAIINPEKTE---------------------------------

Has_Z9_b

----------------------------MPP-----QG--QTGGSWVLYETDAVNE--DTDTPVI----VPPSAEKR---
------------EWK--------IVWRNVILMGMLHIGGVYGAYLFLTTA----MWR-
TCIFAVVLYICSGLGITAGAHRLWAHKSYKARLPLRIMLTLFNTLAFQDAVIDWARDHR
MHHKYSETDADPHNATRGFFFAHVGWLLVRKHPQIKAKGHTIDLS--
DLKSDPILRFQKKHYLFLMPLVCFILPCYIPT-
LWGESLWNAYFVCSIFRYVYVLNVTWLVNSAAHLWGAKPYDKNINPVETRPVSLVVLG
EGFHNYHHTFPWDYKTAELG-DYSLNLTKLFIDTMAAIGWAYDLKTVSTDVIQ-
KRVKRTGDGSH-----------------P----------------------
VWGWDDHEVHQEDKKLAAIINPEKTE---------------------------------

Sli_Z9_b

----------------------------MPP-----QG--QTGGSWVLYETDAVNV--DTEAPVI----VPPSAEKR---
------------EWK--------IVWRNVILMGLLHIGGVYGAYLFLTTA----MWR-

TSLFAVFLYICSGLGITAGAHRLWAHKSYKARLPLRILLTLFNTLAFQDAVIDWARDHRM
HHKYSETDADPHNATRGFFFSHVGWLLVRKHPQIKAKGHTIDLS--
DLKNDPILRFQKKHYLILMPLVCFILPCYIPT-
LWGESLWNAYFVCSIFRYVYVLNVTWLVNSAAHLWGAKPYDKNINPVETKPVSLVVLG
EGFHNYHHTFPWDYKTTELG-DYPLNLTKLFIDFMAAIGWAYDLKTVSSDVIQ-
KRVKRTGDGSH-----------------A----------------------
VWGWDDHEVHQEDKELAAIINPDKTE---------------------------------

Hme_ctg2

----------------------------MPP-----QD--QQTGSWVLYEKD-LNK--NA--STS----VPPTAEKR-----
----------KWQ--------IVWRNVILFLLLHIGGVYGAYLFLMKA----MWA-
TRIFAVVLYLCSGLGITAGAHRLWAHKSYKAKLPLRILLTIFNTIAFQDSVLDWARDHRM
HHKYSETDADPHNATRGFFFSHVGWLLVRKHPEIKSKGHTIDMN--
DLWSDPILRFQKKYYMLLMPVACFILPSYIPT-
LWGESLWNGYFVAAIFRYVYVLNATWLVNSAAHKWGSKPYDKNINPVETKPVSLVVL
GEGFHNYHHTFPWDYKTAELG-DYSFNFSKLFIDGMSSIGWAYDLKTVSREVIE-
KRIKRTGDGSH-----------------Q----------------------EFGYEDLSSSSEDKFNKE------------------
----

Ban_delta9-like_Lienard

----------------------------MPP-----QG--QQQGSWVLYESD-LNK--DATPPPL----VPPSAEKR----
-----------KWK--------IVWRNVILFMLLHLGGLYGAYLFLTQA----MWT-
TRIFTVLLYLSSGLGITAGAHRLWAHKSYKAKLPLRMLLTLFNTLAFQDSVLDWARDHR
MHHKYSETDADPHNATRGFFFSHVGWLLVRKHPEIKAKGHTIDMS--
DLWADPVLRFQKKYYLILMPLVCFILPTYIPT-
LWGESLWNAYFVSAIFRYVYVLNVTWLVNSAAHKWGSKPYDKFINPVETKSVSLVVLG
EGFHNYHHTFPWDYKTAELG-NYSLNFSKLFIDTMAKIGWAYDLKTVSPDVIE-
KRVKRTGDGTH-----------------Q----------------------EWGFEELHKELHEHSQ-------------------
----

Ban_delta11-like_Lienard

----------------------------MAP---------SATETTTITETE-------ETLPKL----VAPQAAPR-------------
--KYD--------IIYFNLLTFGYAHLATLYGVYLACTTA----TWK-
TLMFHHVMFILAAVGITAGTHRLWSHRAYKAKMPLQIILMVLNSFAFQNSAIQWVRDH
RMHHRYSDTDADPHNATRGFFYSHMGWLLVRKHPEMLRRGKFIDMS--
DIYANPVLRFQKKYAIPVFGTLCFALPTLIPMYFFGETLNTAWHL-
TIMRYVFNLHMTFLVNSAAHLWGNKPYDKNMLPAQNLPVSFIAFGEGFHNYHHAFPWD
YKTAELG-NNWLNFSTKFIDFFAWIGWAYDLKEVPLEVAR-ARAERTGDGSN--------------
-------------------------LWGFRDKTE--

Ban_delta9-like_Arun_ACO11.6.7

----------------------------MPP-----QG--QQQGSWVLYESD-LNK--DATPPPL----VPPSAEKR----
-----------KWK--------IVWRNVILFMLLHLGGLYGAYLFLTQA----MWT-
TRIFTVLLYLSSGLGITAGAHRLWAHKSYKAKLPLRMLLTLFNTLAFQDSVLDWARDHR
MHHKYSETDADPHNATRGFFFSHVGWLLVRKHPEIKAKGHTIDMS--
DLWADPVLRFQKKYYLILMPLVCFILPTYIPT-

LWGESLWNAYFVSAIFRYVYVLNVTWLVNSAAHKWGSKPYDKFINPVETKSVSLVVLG
EGFHNYHHTFPWDYKTAELG-NYSLNFSKLFIDTMAKIGWAYDLKTVSPDVIE-
KRVKRTGDGTH-----------------Q----------------------EWGFEELHKELHEHSQ-------------------
----

Ban_delta11-like_Arun_LOC692458.1.1

----------------------------MAP-----SA--TETTTITETEETLPKL-------------VAPQAARR-------------
--KYD--------IIYFNLLTFGYAHLATLYGMYLACTTA----TWK-
TLMFHHVMFILAAVGITAGTHRLWSHRAYKAKMPLQIILMVLNSFAFQNSAIQWVRDH
RMHHRYSDTDADPHNATRGFFYSHMGWLLVRKHPEMLRRGKFIDMS--
DIYANPVLRFQKKYAIPVFGTLCFALPTLIPMYFFGETLNTAWHL-
TIMRYVFNLHMTFLVNSAAHLWGNKPYDKNMLPAQNLPVSFIAFGEGFHNYHHAFPWD
YKTAELG-NNWLNFSTKFIDFFAWIGWAYDLKEVPLEVA-RARAERTGDGSN--------------
-------------------------LWGFRDKTE---

Bmo_5_AGO45855

----------------------------MVE---------TKMEDGKQAATP----------------AEEINLSN--------------
KRE--------ANWPAVLFFIHIHLLSLYGIYLLIYEV----KWM-
TVIFLITLTFPAILAMTTGAHRLWAHRTYKASTELRIVLMLLQTLAGVGPIYNWVRHHRL
HHAHFATENDPFDYNQGFAYAHIFTRMRKLSPYQEKLKESIDMS--
DLENDSVVMFQKTLYWFMYATLFLLLPLNAPLEYWEDSVLSSVFVIGFLRYFILLHASW
LIESAICVWGLKPGEKSPPDSNSVFIITRTF--WPH-YHYLIPYDYKSGEYG-
TYDSGCSSAFIRVWAALGIATGLRTVETNTIQ-KALAEMAKTKK------------------------------
----------PLQICLENALKDQSLSDQHYLKRE-----------------------------------

Dpl_ctg2

----------------------------MPP-----QG--QQSESWVLNETDLNKD----ADTPI----VPPSAEKR------
---------KLQ--------IVWRNVILFALLHIGGVYGAYLFFTQA----MWS-
TRLFAVVLYLCSGLGITAGAHRLWAHKSYKARLPLRMLLTLFNTIAFQDSVIDWARDHR
MHHKYSETDADPHNATRGFFFSHVGWLLVRKHPEIKAKGHTIDMS--
DLKSDPVLRFQKRHYLLLMPLACFILPTYIPT-
LWGETLWNAYFVCAIFRYVYVLNVTWLVNSAAHKWGDKPYDKNINPVETKPVSLVVL
GEGFHNYHHTFPWDYKTAELG-HYQLNFSKLFIDFMATIGWAYDLKTVSTDVIE-
KRVKRTGDGSH-----------------K----------------------EWGQEIKEKISQE---------------------------
----

Poc_Z10

----------------------------MPP-----N---SEETVLCEKE----------DHEKL----VAPQAATR------------
---KHE--------LAIVPISLFTYWHVAGLYGLYLIFAEA----KWQ-
TVVFTLFTYNAGILGITAGSHRLWAHKTYKAKRPLETLLMVFHSLTSQNTVRHWARDH
RFHHKYSDTDADPHNATRGFFYSHVGWLLVKKHPEVLRRSKTIDMS--
DIYNNPVLRFQKNYGLPVITLFAYVLPALIPMYCWEETLNNAWHI-
NLLRIIANLHASCLVNSAAHAFGNKPYDKHIAATQISTLSFITLGECFHNYHHVFPWDYR
TAELG-NNWLNMTTLFIDFFAWVGWAYDLKTASDGMVE-ARAKRTGDGTN---------------
------------------------LWGWGDEDLGREEGGEEVFYGWGDRDMKDTSGVRVYSQE----------
----

Onu_Z/E11

-----------------------------MVP-----Y---ATTADGHPEKDE--------CFEDN----EIKSNSLP-----------
----KLE--------ILYFNVMTFTFLHLSALYGLYLGFTSV----KWA-
TIGLGIIFYFFAEIGITAGAHRLWSHRSYKAKLPLEILLMVFNSMAFQNTALSWARDHRV
HHKCPDTNGDPHNANRGFFYSHVGWLMTKKSDEVIKQGKLCDVA--
DLYSNPVLRFQKKYAVPFIGTLCFVLPTLIPMYFWGETLNNAWHF-
NMFRYVINLNATFCVNSVVHKWGYKPYDKNICPTQNVLLNLAVLGEAFHNYHHVFPW
DYRAAELG-NQKMNPTTLFIDFFAWIGWAYDLKTASKEMIK-SRSERTGDGTD-------------
----------------------------LWGHSADKLK--

Ape_Z11

-----------------------------MAP-----Y---TEKEDELITCEG--------SIEKL----IAPQANKR-------------
--KHQ--------IVYMNLFTFGYWYIAGLYGLYLFFTST----KWS-
TIILNIFLIIASGLGITVGAHRLWAHKCFKAKLPLQIILMILNTLAFQNTAITWVRDHRMHH
KFTDTDADPHNATRGFFFSHVGWLLVKKHPELMKRSKHIDMS--
DIYGNPVLTFQKKYAFPLVATFTFILPTVVPIYFWDESLNNAWHA-
TVFRYAYNLNITFLVNSAAHMWGYRPYDKNIQPTQNIFTTLCTFGEGFHNYHHTFPWDY
RAAELG-NNYLNLSTKVIDFFAWIGWAYDLKTVPADIVN-SRAKRTGDGTD-----------------
------------------------LWGGNHKHTLDEKENRELDDYVHKSAF---------------------------------

Mse_Z11/10-12

-----------------------------MAP-----N---FGTEMSAHIDAE--E-----SYGKL----IPPQAAPR-----------
----KYK--------YLYANMIYFAYWHIAGLYGIYLAFTSA----KWA-
TIILAYLLFVAGDIGVTAGAHRLWAHKSYKAKLPLQILLMLFSTMAFQNTVITWVKDHR
MHHKYSDTDADPHNATRGFFYSHVGWLMVKRHPEAIKRGKSLDMS--
DIYNNPVLKFQKKYAIPLVTTIAFVLPTIIPMYFWDESFSVAWHM-
TMLKYIFGLNAAFLVNSVAHMWGYKPYDKNIAPTQSYIATFATLGEGFHNYHHVFPWD
YRASELG-DNYLNFTTKFIDFFAWIGWAYDLKAAPEDLVQ-
KRIERTGDGTKLLNLTTKFIDFFAWIGWAYDLKTVPEDLLQKRMERTGDGTNLWGRGD
KNMKKDYVKSTDDHE---------------------------------------

Bmo_Z11/10-12_BAD18122

-----------------------------MPP-----NSVDKTNETEYLKDNH-------VDYEKL----IAPQASPI--------
-------KHK--------IVVMNVIRFSYLHIAGLYGLYLCFTSA----KLA-
TSVFAIVLFFLGNFGITAGAHRLWSHNGYKVKLPLEILLMVFNSIAFQNTIFTWVRDHRL
HHKYTDTDADPHNATRGFFFSHIGWLLVRKHPMVKIAGKSLDMS--
DIYCNPLLRFQKKYAIPFIGTICFIIPTLAPMYFWGESLNNAWHI-
TVLRYIFSLNGTFLVNSAAHLWGYKPYDKSLKATQSGMANAFTFGEGFHNYHHVFPWD
YRADELG-DRYINLTTRFIDFFAWMGWAYDLKTASTNIIE-KRALRTGDGTY----------------
-KR----------------------PNGMN--

Lca_Z11

-----------------------------MPP-------YPEEVDTKHIFEEDISHEESKPALKPL----VAPQADNR----
-----------KPE--------IVPLNLITFGYGHLAAIYGIYLCFTSA----KWA-
TIVFAFVLYICAELGITAGAHRLWSHRSYKAKLPLRLILLLFNTLAFQNTAIDWVRDHRM

HHKYSDTDADPHNATRGFFFSHVGWLLTRKHPEVKKRGKDIDMM--
DIYNDSLLKFQKKYAIPFVGLVCFVIPTLMPMYFWNETLNNSWHIATMLRYIVNLNMTF
LVNSAAHIWGYKPYDKSIKPVQNITVSILILGEGFHNYHHVFPWDYRTSELG-
NDFLNFTTLFINLFAKIGWAYDLKTASDKVVA-ARRKRTGDGTN------------------------------
----------LWGWEDKSLMKKATAATVLYPNKYLNLKD------------------------------

Dpu_Z/E11_2

-----------------------------MPP---------NTETFKKLKISEENLE----QFPKL----VAPQAAPR----------
-----KYE--------IIYINVLTFSYWHLAAFYGIYLCFTSA----KWT-
SVFFVFLYCTMGEVGVTAGSHRLWSHKSYKAKLPLQIILMTLSSIAFQNSVIDWVRNHRT
HHKYSDTDADPHNATRGFFFSHIGWLLVRKHSQTKLRGKTIDMS--
DIYNNSVLRFQKKYAIPFIGTICFVLPVLIPMYCWNESFINAWHL-
NMLRYCLSLNFTFLVNSAAHLYGNKPYDKNILPAENKAVSFLTLGEGFHNYHHTFPWD
YRAAELG-NNKLNLTTKFIDFFAWVGWAYDMKTVSDDMIR-SRAKRTGDGSI--------------
--------------------------IWDDGKETIEMGDVSDAIVIKNE------------------------------------

Epo_E11

-----------------------------MAP-----N---VEEIETDLTETE-------EKWEKL----VAPQAAPR----------
-----KHE--------ILYTNLLIFGYGHLAGLYGLYLCFTSA----RLQ-
TIILAFILHAMAILGITAGAHRLWTHRSYKATMPLQIILIIFNSLSFQNSAINWVRDHRSHH
KYCDTDADPHNAARGLFYSHIGWLLVKKHPEVKKRGKMTDMS--
DVYRNPVLRFQKKYAVPFIGTICFVLPTIIPMYFWGESLNNAWHI-
TLLRYIFSMHTIFLVNSVAHLWGNRPYDKNILPADNRTLSIATLGEASHNYHHTFPWDYR
STELG-YLPTNFTTNFIDFFAWIGWAYDLKTTSGEIIN-SRIQRTGDGTH-----------------------
-------------------SRSKKNISTQDE---

Ave_Z/E11

-----------------------------MAP-----N---AEDIETNMPETE-------ENWETL----VAPQAAPR---------
------KYQ--------IVYKSLLTFGYGHLAGLYGLYLCFTSA----KWQ-
TIGLAIILHAMAILGITAGAHRLWTHRAYKATVPLQIILIIFNSLSFQNSAFTWIRDHRLHH
KYSDTDGDPHNATRGFFYSHIGWLLVRKHPEVMKRGRMTEMS--
DIYSNPIIMFQKNYAIPFIGTVCFVLPTIIPMYFWGETLNNAWHI-
TVLRYIFSLNCIFLVNSAAHLYGYKPYDKNILPAENKAASIASFGEAFHNYHHVFPWDYR
ASELG-NITMNWTIYFIDFFAWIGWAYDLKTASDETIK-SRIKRTGDGTD---------------------
-------------------FSGQQIYAR--

Cpa_E11

-----------------------------MAP-----N---VEDMESDMPESE--------KWEKL----VAPQAAPR--------
-------KYE--------IIYTNLLTFGYGHIAGLYGLYLCFTSA----KWQ-
TVILAIILNEMAILGITAGAHRLWSHRSYKAAVPLQIILMIFNSLAFQNSAINWVRDHRMH
HKYSDTDGDPHNASRGFFYSHVGWLLVKKHPEVKKRGKMIDMS--
DIYSNPVLRFQKKYAIPFIGMICFVLPTIIPMYFWGETLSNAWHI-
TMLRYVFSLNSIFLVNSAAHLYGYRPYDKNILPAENKIALIACLGDSFHNYHHVFPWDYR
ASELG-NIGMNWTAQFIDFFAWIGWAYDLKTASDENIN-SRMKRTGDGTD------------------
----------------------ISGQKYSCESSEVLQ---

Tni_Z11

--------------------------MAVMAQ---------TVQETATVLEEEARTV-------TL----VAPKTTPR------
---------KYK--------YIYTNFLTFSYAHLAALYGLYLCFTSA----KWE-
TLLFSFVLFHMSNIGITAGAHRLWTHKTFKAKLPLEIVLMIFNSLAFQNTAITWAREHRLH
HKYSDTDADPHNASRGFFYSHVGWLLVKKHPDVLKYGKTIDMS--
DVYNNPVLKFQKKYAVPLIGTVCFALPTLIPVYCWGESWNNAWHI-
ALFRYIFNLNVTFLVNSAAHIWGNKPYDKSILPAQNLLVSFLASGEGFHNYHHVFPWDY
RTAELG-NNFLNLTTLFIDFCAWFGWAYDLKSVSEDIIK-QRAKRTGDGSS-----------------
GV----------------------IWGWDDKDMDRDIKSKANIFYAKKE------------------------------------

Tpi_Z11/11-13

-----------------------------MAP---------NTRENETIYDEV------EHKLEKL----VPPQAGPW---------
------NYK--------IVYLNLLTFSYWLIAGAYGLYLCFTSA----KWA-
TIIFEFILFFFAEMGITAGAHRLWTHKSYKAKLPLEIFLMVLNSVAFQNTATDWVRDHRL
HHKYSDTDADPHNAARGLFFSHVGWLLVRKHDEVKKRGKFTDMS--
DIYNNPVLKFQKKYAIPFIGAVCFILPTVIPMYFWGESLNNAWHI-
CILRYAMNLNVTFSVNSLAHIWGNKPYDKDIKPAQNFGVTLATFGEGFHNYHHVFPWD
YRTSELG-DNKFNFTTKFINFFERIGLAYDLKTVSDDVIA-QRAKRTGDGTH------------------
-----------------------LWDCADKNNNDVVQTKAQIDTLCTKHE---------------------------------

Che_Z/E6

-----------------------------MPP---------NTKEKEMNKLAGISGRNEE----------LAPQAAPR---------
------KFE--------IVYRNIVTFSFMHVAGLYGLYLCFTSA----KWQ-
TIVLAFVLYTFAEIGVTAGAHRLWAHKTYKAKTPLQIILMVLNSIAFQNSAIGWVRDPRL
HHKYSDTDADPHNATRGFFYSHVGWLLVRKHPEVIKRGKTMEMS--
DIYSNPVLWFQKKYAVPFIGIICFGLPTLVPMYFWGETLNNAWHI-
TMLRYIANLNVTFLVNSAAHMFGNKPYNKNILPAQNISVSILTFGEGFHNYHHVYPWDY
RTAELG-NNMLNMTTLFIDFFAWIGWAYDLKTVSSAAIE-SKAKRTGDGTN------------------
-------------------------LWGWGDKDMLREDQKAAEILFARLK------------------------------------

Sli_Z11/10-12

-----------------------------MAQ---------CVQTTTILEQKEEKTV-------TL----LVPQAGKR----------
-----KFE--------IVYFNIITFAYWHIAGLYGLYLCFTST----KWA-
TVLFSFFLFVVAEVGVTAGSHRLWSHKTYKAKLPLQILLMVMNSLAFQNTVIDWVRDH
RLHHKYSDTDADPHNASRGFFYSHVGWLLVRKHPDVKKRGKEIDIS--
DIYNNPVLRFQKKYAIPFIGAVCFVLPTLIPVYGWGETWTNAWHV-
AMLRYIMNLNVTFLVNSAAHIYGKRPYDKKILPSQNIAVSIATFGEGFHNYHHVFPWDY
RAAELG-NNSLNFPTKFIDFFAWIGWAYDLKTVSKEMIK-QRSKRTGDGTN-------------------
-----------------------LWGLEDVDTPEDLKNTKGE--

Has_Z11

-----------------------------MAQ---------SYQSTTVLSEEKEPTL-------TH----LVPQASPR------------
---KYQ--------IVYPNLITFGYWHIAGLYGLYLCFTSA----KWA-
TILFSYILFVLAEIGITAGAHRLWAHKTYKAKLPLELLLMVFNSIAFQNSAIDWVRDHRLH
HKYSDTDADPHNASRGFFYSHVGWLLVRKHPEVKKRGKELNMS--

DIYNNPVLRFQKKYAIPFIGAVCFALPTMIPVYFWGETWSNAWHI-
TMLRYIMNLNVTFLVNSAAHIWGNKPYDAKILPAQNVAVSVATGGEGFHNYHHVYPW
DYRAAELG-NNSLNLTTKFIDFFAAIGWAYDLKTVSEDMIK-QRIKRTGDGTD--------------
-------------------------LWGHEQKCDKGSGVNDKLS--

Mbr_Z11

----------------------------MDQ---------SVRTTTILRGKEEPTL-------TW----LVPQASPR-----------
---KYQ--------IVYPNLFTFGYWHIAGLYGLYLCFTSA----KWQ-
TMIFSFILFVLAEIGVTAGAHRLWAHKTYKAKLPLQILLMILNSIAFQNSAIDWVRDHRLH
HKFSDTDADPHNATRGFFYSHVGWLLVRKHPEVKRRGKELDMS--
DIYNNPVLRFQKNYAIPFIGAVCFGLPTLIPVYCWGETWSTAWHI-
TMFRYVMNLNVTFLVNSAAHIWGKKPYDKKILPAQNIAVSIATCGEGFHNYHHVFPWD
YRAAELG-NNSLNVTTKFIDFFAWIGWAYDLKTVSQDMIK-HRALRTGDGSN---------------
-------------------------LWGLEDKYVCEKNEGKDE---

Yev_Z/E11

----------------------------MPP---------NSANSEPLLHDEVKEE----QLEKL----VAPQAGPR--------
-------KFQ--------IVYPNLITFGYGHISLLYGAYLMFTFA----KWQ-
TIVFAHVMIILSGFGITAGAHRLWAHRTYKAKMPLQILLMLLNTLAFQNTAMDWVRDH
RLHHKYSDTDADPHNATRRFFYSHVGWLLVRKHKEVKRRGKLIDLS--
DIENNPVLSFQKKYAIPLVSLVTFVMPTVIPMYFWGETLTNAWHVATLYRYVFVLNATF
LVNSAAHYWGYKPYEKSILPSQNHFVSLFALGEGFHNYHHVFPWDYRTAELG-
NNTLNPTTKFIDFFAWIGWAYDLKAVPDDMIN-NRKSRTGDGSS------------------------------
----------LWGWGDKDQPKEEMNAAIIKYPDENDKDK------------------------------

Ypa_Z/E11

----------------------------MAP---------NSANSEPLLHDEVKEE----QLEKL----VAPQAGPR-------
--------KFQ--------IVYPNLITFGYGHISLLYGAYLMFTFA----KWQ-
TIVFAHVMIILSGFGITAGAHRLWAHRTYKAKMPLQILLMLLNTLAFQNTAMDWVRDH
RLHHKYSDTDADPHNATRGFFYSHVGWLLVRKHKEVKRRGKLIDLS--
DIENNPVLSFQKKYAIPLVSLVTFVMPTVIPMYFWGETLNNAWHVATLYRYVFVLNVTF
LVNSAAHYWGCKPYEKSILPSQNHFVSLFALGEGFHNYHHVFPWDYRTAELG-
NNTLNPTTKFIDFFAWIGWAYDLKAVPDDMIN-NRKSRTGDGSS------------------------------
----------LWGWGDKDQPKEEMNAAIIKYPDENDKDK------------------------------

Bmo_10_AGO45852

----------------------------MSP---------NIATIVPSQETE-------NEYEKL----EPPQASER--------------
-KYE--------IIYFNIFTFTFAHLSALYGVYLCFTSA----KRE-
SLILGYIIGVLAGFGVTAGAHRLWTHRTYKAKMPLQILLMLFYSFAYQNSAVHWIRDHR
LHHKYSDTDADPHNATRGFFYSHMGWLLVKKHPEVKRRGRSLDMS--
DIYSNRVLMFQKKYAIPITGTICFVIPTLLPMYLWGESFKNAWHI-
TILRYIITLHVAFLVNSAAHIWGYKPYDKRILPTQNLFVSFMAFGEGFHNYHHVFPWDYR
TAELG-NNYLNLTTMFIDFFAWLGWAYDLKSVPVSLAE-SRARKTGDGTG------------------
---------------------LWGWGDKDMSEDLKRQTVGEN--------------------------------------

Dpl_ctg1

------------------------------M-----S---CDETEESKSDD---------VFEKL----VAPQAAPR--------------
-KYE--------IIHSNLLTFSYGHLAALYGTYLCVTVA----SWK-
TLLFHFIIFVLAAIGVTAGAHRLWTHRAYKAKLPLQIILMLMNTLAFQNTVIDWVKKHRL
HHRYSDTDADPHNATRGFFYSHVGWLLVKRHEEVRRREKLIDMS--
DIYDNPVLTFQKKYAVPFIGTLTFVLPTLIPMYFFGETFWTAWHL-
TVLRYILNLNGTFLVNSAAHMFGNKPYEEHIKPSQNLFVSFVSFGEGFHNYHHVFPWDY
RTAELG-NNYLNLTTKFIDFFAWLGWAYDRKTIPDDLIR-SRSKRTGDGTN-------------------
----------------------SWGFSKTE--

Bmo_9_BAD18124

-----------------------------MEA---------KQNNLAPTLEEE-------AQFEKL----IAPQASDR-----------
----KHE--------IIYANLITFAYGHISALYGLYLCFSSA----KWA-
TIIMAYVILIAAEVGVTAGAHRLWTHRAYKAKRPLQIILMVMNSFAFQNSAITWIRDHR
MHHRYSDTDADPHNATRGFFYSHIGWLLVRKHPEVKRRGKTIDMS--
DIYSNPVLVFQKKYAIPFIGAVCFVIPTLVPIYFWGETLTNAWHI-
TLLRYIISLHVTFLVNSAAHLWGTRAYDKRIFPAQNLIVSLLAVGEGFHNYHHVFPWDYR
TAELG-NNYLNLTTKFIDFFAWLGWAYDLKSVPDSAVQ-SRAARTGDGTN------------------
-----------------------SWGWPEEDANEDILKQTPPL---------------------------------------

Hme_ctg0

-----------------------------MSP---------YIESTTTTTTEEPLVD----HYEKL----VAPQADNR---------
------KYE--------IVYQNLITFGYGHAAALYGLYLACTSA----TWS-
TIIFHYILFAAASIGITAGAHRLWTHRAYKAKMPLQIILLVMNSLAFQNTAITWVRDHRLH
HRYSDTDADPHNATRGFFFSHMGWLLVRKHPEVKRRGKFIDMS--
DIYANPVLTFQKKYALPFIGTICFVLPTIIPMYFWGETLSTAWHL-
AVLRYVCNLHNAFLVNSAAHMWGNKPYDKSIQPAQNLAVSILAFGEGFHNYHHVFPW
DYKTAELG-NNYLNATTYFIDFFSRLGWAYDLKSVSEDHIQ-SRSSRCGDGSD---------------
--------------------------SWGLKS---

Hme_ctg1

-----------------------------MSP------------CTGTLIGTTTEEPLVD-HYEKL----IAPQAEPR----------
-----KYD--------IVYESVVTVVYLHAAALYGLYLACSTA----TWS-
TLIFHNILFALATLGVTAGAHRLWTHRAYKAKMPLQLILIVLNSLAYQYTAITWIRDHRL
HHRYSDTDADPHNATRGFFFSHMGWILVRKHSEVKRRGKFIDMS--
DVYANPVLRFQKKYALPFIGTICFVLPTIVPIYFWGESLNTAWHL-
AILRYVCNLHNTCFVNSLAHLWGNKPYDKSIKPSENLVISFLTLGEGFHNYHHVFPWDY
KTGELG-NNFLNVTTYFIDFFAWLGWAYDLKSVSEEHIQ-SRSSRCGDGSN-------------------
----------------------SWGIKGY--
