# Supplementary material for: Mosaic Evolution of Molecular Pathways for Sex Pheromone Communication in a Butterfly
Source: Genes (Basel). 2022 Jul 31;13(8):1372. doi: 10.3390/genes13081372 (PMC9407440; doi:10.3390/genes13081372)
Supplement: Supplementary file 1 [file genes-13-01372-s001.zip › Supplementary File S4.pdf]

Supplementary File S4: Phylogenetic reconstructions for the reductase gene family
Aligned amino acid sequences used for the reductase phylogenetic reconstruction:

>Ase_pgFAR

---MPV
LTSRED-EKLSVPEFYAGKSIFV----------------TGGTGFLGKVFIEKLLYCCPD
IDKIY-----MLIREKKNLSIDERMSKFLDDP--LFSRLKEER---PGDL-EKIVLIPGD
ITAPNLGLSAENERILLEKVSVIINSAATVKFNEPLPIAWKINVEGTRMLLALSRRMKR-
IE---------VFIHISTAYSNASSD--RIVV-------DEILYPAPADMDQVYQLVK--
--DGVTEEETERLLNGLPNTYTFTKALTEHLVAEHQTY--VPTIIIRPSV----------
---------------VASIK--DEPIRGWLC--NWFGATGISVFTAKGLNRVLLGKASNI
VDVIPVDYVANLVIVAGAKSGG----Q-KSDE-------LKIYNCCSSDCNPVTLKKIIK
EFT-EDTIKNKS--HIMP-LPGWFVFTKYKWLLTLLTIIFQMLPMYLADVYRVLTGKIPR
----------YMKLHHLVIQTRLGIDFFTSHSWVMKTDRVRELFGSLS-LAEKHMFPCDP
SSIDWTD--YLQSYCYG---VRRFLEKK-----K--------------------------

>Hsub_pgFAR

---MVV
LTSKE--TKPSVAEFYAGKSVFI----------------TGGTGFLGKVFIEKLLYSCPD
IGNIY-----MLIREKKGLSVSERIKHFLDDP--LFTRLKEKR---PADL-EKIVLIPGD
ITAPDLGITSENEKMLIEKVSVIIHSAATVKFNEPLPTAWKINVEGTRMMLALSRRMKR-
IE---------VFIHISTAYTN--TN--REVV-------DEILYPAPADIDQVHQYVK--
--DGISEEETEKILNGRPNTYTFTKALTEHLVAENQAY--VPTIIVRPSV----------
---------------VAAIK--DEPIKGWLG--NWYGATGLTVFTAKGLNRVIYGHSSNI
VDLIPVDYVANLVIAAGAKS------S-KSTE-------LKVYNCCSSACNPITIGKLMS
MFA-EDAIKQKS--YAMP-LPGWYIFTKYKWLVLLLTILFQVIPAYITDLYRHLIGKNPR
----------YIKLQSLVNQTRSSIDFFTNHSWVMKADRVRELFASLS-PADKYLFPCDP
VNINWRQ--YIQDYCWG---VRHFLEKK-----T--------------------------

>Has_pgFAR

---MVV
LTSKE--TKPSVAEFYAGKSVFI----------------TGGTGFLGKIFIEKLLYSCPD
IGNIY-----MLIREKKGLSVSERIKQFLDDP--LFTRLKEKR---PADL-EKIVLIPGD
ITAPDLGITSENEKMLIEKVSVIIHSAATVKFNEPLPTAWKINVEGTRMMLALSRRMKR-
IE---------VFIHISTAYTN--TN--REVV-------DEILYPAPADIDQVHQYVK--
--DGISEEETEKILNGRPNTYTFTKALTEHLVAENQAY--VPTIIVRPSV----------
---------------VAAIK--DEPIKGWLG--NWYGATGLTVFTAKGLNRVIYGHSSYI
VDLIPVDYVANLVIAAGAKS------S-KSTE-------LKVYNCCSSACNPITIGKLMS
MFA-EDAIKQKS--YAMP-LPGWYVFTKYKWLVLLLTILFQVIPAYITDLYRHLIGKNPR

----------YIKLQSLVNQTRSSIDFFTSHSWVMKADRVRELFASLS-PADKYLFPCDP
TDINWTH--YIQDYCWG---VRHFLEKK-----TTNK-----------------------

>Har_pgFAR

---MVV
LTSKE--TKPSVAEFYAGKSVFI----------------TGGTGFLGKVFIEKLLYSCPD
IENIY-----MLIREKKGLSVSERIKQFLDDP--LFTRLKDKR---PADL-EKIVLIPGD
ITAPDLGINSENEKMLIEKVSVIIHSAATVKFNEPLPTAWKINVEGTRMMLALSRRMKR-
IE---------VFIHISTAYTN--TN--REVV-------DEILYPAPADIDQVHQYVK--
--DGISEEDTEKILNGRPNTYTFTKALTEHLVAENQAY--VPTIIVRPSV----------
---------------VAAIK--DEPLKGWLG--NWFGATGLTVFTAKGLNRVIYGHSSYI
VDLIPVDYVANLVIAAGAKS------S-KSTE-------LKVYNCCSSSCNPVTIGTLMS
MFA-DDAIKQKS--YAMP-LPGWYIFTKYKWLVLLLTFLFQVIPAYVTDLSRHLIGKSPR
----------YIKLQSLVNQTRSSIDFFTNHSWVMKADRVRELYASLS-PADKYLFPCDP
TDINWTH--YIQDYCWG---VRHFLEKK-----SYE------------------------

>Hvi_pgFAR

---MVV
LTSKE--TKPSVAEFYAGKSVFI----------------TGGTGFLGKVFIEKLLYSCPD
IVNIY-----MLIREKKGLSVSERIKQFLDDP--LFTRLKDKR---PADL-EKIVLIPGD
ITAPDLGITAANEKMLIEKVSVIIHSAATVKFNEPLPTAWKINVEGTRMMLALSRRMKR-
IE---------VFIHISTAYTN--TN--REVV-------DEILYPAPADIDQVYQYVK--
--EGISEEDTEKILNGRPNTYTFTKALTEHLVAENQAY--VPTIIVRPSV----------
---------------VAAIK--DEPLKGWLG--NWFGATGLTVFTAKGLNRVIYGHSNYI
VDLIPVDYVANLVIAAGAKS------N-TSSE-------LKVYNCCSSSCNPVKIGTLMS
MFA-DDAIKQKS--YAMP-LPGWYIFTKYKWLVLLLTFLFQVIPAYITDLSRHLVGKSPR
----------YIKLQSLVNQTRSSIDFFTNHSWVMKADRVRELYASLS-PADKYLFPCDP
VNINWTQ--YLQDYCWG---VRNFLEKK-----T--------------------------

>Dpl-ctg3

-------MYPKIAESYDGQSVFM----------------TGGTGFLGKVLLEKLLYSCPG
INKVY-----LLVREKQNATVQQRIQKLIEEP--LFARLREER---PEAL-EKISPIAGD
ISEPKLAIKTEDEQLLAEEVSIVFHVAATIKFNEPLDVAMNVNVAGTGRVLNLAQKMKN-
IK---------AFVYVSTAYSN--TD--RKIV-------EEVIYPAPASLNEVKKLLE--
--IGITEEQVKELIKGRPNTYTFTKALAENLVADNHGN--IPAVIIRPSI----------
---------------VTSSK--VEPVVGWID--NWFGASALLTTISKGLNRVILSDSENS
LDLIPVDYVSNLTIVAAARC------E-CSKN-------VTVYNCCTSGNNPIKMGHLAK
LII-ADSKEHKF--NDVP-MPTIH-FTQYKWVLILVTLLFQTIPAYIADLFLLLTGKKTR
----------YTKVQAKVLFIRDTLEFFTSNNWSIRAPQTIALANSLS-SSDRILFPFNP
TEIVWNE--YIPTYCQG---IRQYLCKK--------------------------------

>Ban_wFAR1

-------MYQAIAECYEGQSVFM----------------TGGTGFLGKVLLEKLLYSCPG
IEKVY-----LLVREKQGATAQQRVQKLLEQP--LFSRIKEEK---PQAF-EKIIPIVGD
ISEPQLGIKAEDEELLAEKVSIVYHVAATIKFNEPFEQAMNVNVAGTGRVLNLSKRMKN-
VK---------AFVYVSTAFSN--TD--RKVI-------EEIIYPAPASLNEVKKLLE--
--IGITDAQVKELIRGRPNTYTFTKALAENLVADNHGN--VPAVIIRPSI----------
--------------VSSSK--REPMVGWID--NWFGASALITTIMKGLNRVLLSSRDNS
LDLIPVDYVSNLIIVAAAKC------K-CSKN-------VGVYNSSTSGENPLRIGRLAE
LII-ADSNKHKF--YDVP-MPMVY-FTRFKWVVLLITLFFQTLPAYIADIFLLIRGKKTR
----------YVKIQSKVSFVRDTLEYFTSNTWSISSRQTTALSQSLS-PSDRYLFPCDP
TDIDWKD--YIPTYCQG---IRQFLCKT-------------------------------

>Ban_wFAR2

------------------------------MYRNVNNNYKGHSVYTNGAGSRVKSLLSS
ATDNTN-EYQSIAECYKGQSVFI----------------TGGTGFVGKVLLEKLLYSCPG
IDKVY-----LLVRETQGATAHQRMQKLLEEP--AFSRIKEEN---PQAF-EKVIPIVGD
ITQPQLGIMAENEELLIKEVSFVYHVAATTKFNETLDIAMNVNVAGTGRVLDLSKRMEN-
IK---------AFVYVSTAYSN--TD--REVV-------EEVLYPAPVSLNEVHKLLK--
--IGITDAQVKELIKGRPNTYTFTKALAENLVADNHGH--VPAIIVRPSI----------
--------------VSSSK--KEPITGWID--SWYGATFLATVTMKGFNRVFVSSYEYN
LDFIPVDYVSNLIIVAAARC------K-CSDK-------VDVYNSSTSGENPLKIGAFFD
DII-AYSCKHKF--YDIP-LPMAY-LTRYRWVMFLITLLLQTLPAYIADLFLLIVGKKPR
----------YVKLASKISAAHEVLDYFPSRTWSMSARQTTALFQSLS-PSDRDQFPCDP
TDIDWKE--YIVTYCQG---IRQFLCKS-------------------------------

>Ban_GS8R7LM02B1WGH_gene9

--------------------------------------MSNTFWLFAFLSVVTVYGKRAT
---------~~~-VTIPPEYAAELVTASVGCVRNTGVDPNILDQIVQWNFQETDNVKKFI
YCMSLASG~F~GDSDGHLIIDKVARLVSDSNKKKDELINALKECNTLSGSDKYDTLFKTV
KCNREKQPIVFSF
>Ban_GS8R7LM03D3OA9_gene11

-------MQDKPQVPLIVLNNGRTCPILGLGTWKQLGNVMLRQSAPGDVMRAVKDAIDVG
YRHIDCAFVYGNEKEVGEALKTKFQEGVVKREDMFVTSKLWNTYHRPDLVEGALKASL
AN
LGLEYLDLYLIHWPEAYQEGGDIFPKDAEGKVIPSPVDFVDTWKAMEAQVDKGLVRSIG
L
SNFNKRQTDRVMKAARIKPAVVQIEVNPYINQHKLIQHCRSLGLAITAYSPLGSPDRPWA
KPGEPHLLEDPRLQVIAKRYNKTVAQILIRYAIDRWLIVIPKTVTRSRLQQNMEVFDFQL
SPQDMELLQKLECNGRFCQMDDAHHPDYPFHDEY
>Hmel-ctg1

-------MYKSVAECYEGESVFI----------------TGGTGFLGKVILEKLLYSCPG
INKVY-----LLVRGKANATVQQRIQNILEQP--LFSRLKKEN---PQAL-EKIVPIVGD
ISEPKLGLKAEDEELLLNTVSIVFHVAATIKFNEPLEVAVNINVGGTARVLNLCQRVPG-
IK---------AFVYVSTAYSN--TD--RKII-------EEVVYPTPASLSEVKKLIE--
--IGITEDQVQQLIKGRPNTYTFTKALAENVVVENKGR--VPTIIVRPSI----------
---------------VSSSK--GEPIVGWID--NWFGASALITTIAKGLNRVLLASPLNT
LDLIPVDYVANLIIVAAAKC------E-CTKE-------VAVLNSCTSGENPIVMRRLAD
LLI-ADSHKHKF--YDVP-MPSVH-FTRFRWVVFLMTLVLQTIPAYIADIFLLIRGKRTK
----------YMKIQNRVSLVRNTLEYFTSNNWLICAPKTRALAESLS-SEDKKQFPCNP
RDIDWDL--YIPSYCQG---IRQFLCKS--------------------------------

>Hmel-ctg7

----MK-IYQPIADCYEGQSVFI----------------TGGTGFLGKVLLEKLLYSCPG
INKIY-----ILIREKANVSIEERLQIIFEQP--IFKRIKCEN---PEAL-LKIVPIRGD
VSLPDLGMTVEDKHLLIDTVSRVFHLAATVKFNEPLNIALNINVDGTQHTLSFCKNMKN-
IK---------SFVYVSTAFSN--AD--KKII-------EEKTYPVSKTLSEIKNLID--
--NGLDENLIKELIGGKPNNYAFTKAMAENVIVEGHGN--VPVVIVRPSI----------
---------------VSPSI--SEPLSGWID--NWFGATSLIVAAGKGFNRVVHTSADNT
LDLIPVDYVSNAIIVAAAKC------K-WTKD-------VPVYNSCSSRRNPLRMQKLGD
LTI-ADSYKNKF--YDIP-LPFVR-FTSSKLIVFLIALFMETIPAYIADFFLLLTGKKCR
----------YVKAVGKLVIIREKLHFFITNSIIIQSQKTRNLAESLS-PSDKRIFPFNP
IDIDWDN--YIPIYCHG---IREFLLKK--------------------------------

>Hmel-ctg2

----MC-EYTPVADFYNGESILI----------------TGGTGFLGKVVLEKLLYSCPG
INKIY-----ILIREKQNSDGPHRVQGILNQQ--IFDRVKNAR---PLAP-VKVIPIVGD
ISEPDLGLSVADDKNIRDNVSILFHVAANIRFNEPLEIAMNVNLMGTSRTLELFRRLEN-
KK---------AFVYVSTAYTN--LD--RKYI-------EEIIYPAPFSIKELKTLLD--
--IGIDEDQVKELTKCKLNTYIFTKAMAESLVAQN-CD--LPVTVVRPSI----------
--------------ISASI--SEPKPGWID--TWMGASGVMTTIAKGFNRVLLSNPNNF
LDLIPVDYVSNLMIVAAAKG------I-KTGE-------VAVYNASTSGENPISIKEFMA
LNI-SDSVKNKF--NDFP-KPGVY-YTTKKWIVILITVLLQVLPAYIADLLLFIMRKNTR
----------YVKLQAKVTLARDIVEFFTLNNWRIRATKTCTLAHSLS-PADRNEFPFNP
SDINWKD--YIPLYCYG---IRNFLLNV---K----------------------------

>Hmel-ctg4

--MLRTKYKLCS
ILLRE--KHTSVAECYEGQSAFI----------------TGGTGFVGKVLVEKLLYQCPG
IEKIY-----LLLREKKNVAPHLRLQQTLEEP--IFSRIRNEK---PEVL-DKIVPIIGD
IAKPELAMRIEDQKLLFEKVTTVFHVAASVSFDDPLDVAMNINVGGLERVFHLCHQMTA
-
LR---------VLLHVSTAYCN--TD--KKVI-------EEIVYPPPASLDKVNDMIA--
--KGINNDQVKMLINKQPNTYAFAKAIAETMIVEKRGN--IPTVIVRPTI----------
--------------VSASK--NEPFVGWVD--NWFGMTGLMALTLLGRNRVLMCKSENV
MDLTPVDYVSNLIIVAAAMKST----S-NDNS-------LSVYNCATSKENPLKAEKVRK
LVV-DYSYKNKI--YDVP-YPYLH-YTTSKWVEWLIITTFNTIPAYLTDTVLLWRGKKPR
----------YINFHSKISRMRNVLEFFSSNSWEISASRTRALSASLT-PKDKENFKFDP
IDIDWND--YFPKYCGG---VSRYMLKR---ER---------------------------

>Hmel-ctg5

--MFRLKQRFSG
VSARF--IHTPVAKCYDGQSVFI----------------TGATGFLGKVLLEKILYSCPG
INKVY-----LLIRENRESDVHQRIKKILDQP--LFSRLRNDY---PNAI-QKIVPIAGN
ISKPQLGIKNEDIRILQNNVSIVFHSAATVKFDEPLDVSTNVNVNGTQRVLNLCHSMKY-
IK---------ALVYVSTAFIN--KY--CDGL-------DEKLYEPPISVTILQEIIK--
--AGVSEDQLQKLLEGRPNTYTFTKAVAESLVAEQHGN--IPTAIIRPSA----------
--------------ITCSK--SEPIAGWLD--NWFAVNTILMMIGNGWIRCLLSKPENV
YDIIPVDYVANLMMVAAVRC------D-GKNG-------VEVFNCTSSRENPLTTQVFIN
SII-QHSSKHKN--YDFP--PHVY-LTSSKTIWNLITLIFETLPVNVADLYLTVKGKKPR
----------YKKIQNKMNQVRGVLEYFSKHNWSLPATRTRALAKNLS-PNDRETFPFDP
RSFNWEE--YMPMYCES---VRKYLLKK--------------------------------

>Hmel-ctg6

--MFRLKQRFSG
VSARF--IHTPVAKCYDGQSVFI----------------TGATGFLGKVLLEKILYSCPG

INKVY-----LLIRENQESDVHQRIKKILDQP--LFNRLKNDY---PHAI-QKIVPVAGN
ISKPQLGIKDEDIQILQDKVSIVFHSAATVKFDEPLDVSTNVNVNGTQRVLNLCHSMKH-
IK---------ALVHVSTAFIN--KN--SDGL-------DEKLYDSLISVTKLQEMID--
--AGVSEDQLQKLLDGRPNTYTFTKAMAESLVAEQHGN--IPTAIIRPSA----------
---------------ITCSK--SEPIVGWLD--NWFVVNAFLMMIGNGWLRCLLSKPEHV
MDIIPVEYVANLMIVAAVRC------D-GKNG-------VEVFNCTSSRENPLTTQVFIN
SII-QHSSKHKN--YDFP--PHVY-LTSSKTIWNLITLIFETLPVNVADLYLTVKGKQPR
----------YKKIQNKMNQVRGILEDFLKYNWSLPAPRTRALAKSLS-PNDRETFPFDP
RSFNWEE--YMPMYCES---VRKYLLKK--------------------------------

>Bmo_swdb1

-MIAHD-SYKSIPDFYAGKTVFI---------------TGGTGFLGKVLVEKLVYSCPA
LNKVY-----VLIRETPKQNVLQKLKQLLDSP--IFTRLKNER---PEDL-NKIVPIAGD
LCKPNLGLEPSDEEKLIDEVSVVIHSGATVKFNDALEYAMKINYQGTEQVMRLCRKIKN-
IK---------VFVYISTTYAH--TQ--LDII-------EEVIYPPPARVAEVYETIKRH
G---NNEKNISKILNGQPNTYAFTKALAENLVAENHGD--IPTVVIRPSI----------
---------------ITAGI--TEPLEGWVD--TWNGATGIMHLIGKGLCRVLPGNEQNI
LDLIPVDFVCNLALVAAAKCNR--------------------------------------

----------FFRTQSKLYSIVKTLRCFIFKSWVFKCERVCALNAELS-DLDRAVFNCDP
STIHWEQ--YAQIYFDG---VTKFLLEK-----PKNLRTDASNN---------------

>Dpl-ctg2

-MVPTS-NYTPVSDFYNGKTIFV---------------TGGTGFLGKVLIEKLLYSCPG
ISKIY-----MLVREKRNLTATDRMKKFFDCP--VFDRLKKEK---PENL-KKLKPVNGD
LSQPCLGIDPKELQVIKDEVTIVFHFAATVKFNEPLSIAMKINVEGTEQVIDLCHSLKK-
IE---------VFVYMSTIFSN--TDDKLNSV-------EERLYRSPKEVDEIYKMIKE-
--NDPREVFNPEVLDGRPNTYTFTKAVAENIVAQKRGN--LPTIMIRPSV----------
---------------VTPAK--EEPVRGWVA--NWMGPTATLLYLSRGWIRCHYGEDDFT
FEIIPVDYVVNLTIISAAKFKS-----------------------SSNHQVESWPTFCF
FIV-GVIITPSI----DIPFPWIT-FSRSLWFLGIISFFMQLLPAYIADLYLFICGHPTR
----------YVKMLKKYSQNMSALHYFSSRTWYMTANRSQELIDGLS-AEDQKIFPCSP
ASIDWSE--YMATYLHG---VYRFLLKS---HY--------------------------

>Hmel-ctg3

---MAS
LSNTIP-NNSSISQYYANKSIFI---------------TGGTGFLGMILIEKLLYSCPD
IKKIY-----ILIRRKKSVLPEERLKQLINGK--IFDRIRKKC---PDNL-SKIVLVKGD

LIEFDLGLNPKELKMLQDEVSVVFHLAATLKFNEPLSVAMKVNVEGTREVLRVSQSMK
N-
IE---------IFVYMSTIFSN--ADHERSTI-------VEELYPSPKDINEVYEMINQ-
--NNPNETFCPELLDRRPNTYTFSKAIAENIVAENRGA--LPTIIIRPSV----------
---------------VTSCY--NEPMEGWIA--NWMGPTPIFTLVGQGLIRCLYGKTHYT
FEIIPADYVVNLTLCATAKH------Q-GGKE-------LSIYHSCSTADNPITLAETSE
YVS-KESVKQKF--NLLP-YPGMM-LIENNWLLYLLHFIFHVCPAFIADLLLFISGHPLR
----------YVKLLQKMTYCMKVLKYFSSRSWAMKATNARGLLNSLS-DVDKREFPCDP
VNIKWDE--YFPIYLFG---IRKYLLKM-----K--------------------------

>Yro_pgFAR

--MVQL-KEDSVAAFYAEKSIFI----------------TGGTGFLGKVLIEKLLYSCKA
VDQIY-----VLIRKKKDQTPSERIAQLLESE--LFSRLRKDD---PSAL-KKVVPVVGD
LTMPNLGLSAAVEDLIVSKVTVIFHVAATVKFNERMKNALVNNVEATREVINLCHRLEK
-
VD---------AFIHVSTAYSN--TD--QKVV-------EERVYPPPAPLSEVYAFVKN-
--YGDDMDIIQNLLNGRPNTYTYTKALAEDIVLKEHGG--IPTAIIRPSI----------
---------------VLSVL--KEPIPGWLD--NWNGPTGLLHASSQGVHCSMLGSGSNV
ADLIPVDIVTNLMIVVASRC------R-KSNG-------LKVYNSCSGTTNPITYQAFTK
MFL-DSCISRGW--NKVP-FPLLI-FVKWAFLNRVLKFLLVIVPFFLIDVYLRFFGK-PN
----------YMRMITYTKKAEDLMTFFTSHEWQFKDGNVRDLINMMS-PEDRKIFYCDP
EEIQWKP--YFDDYCVG---VFKYLLKR-----KV-------------------------

>Ypa_pgFAR

--MVQL-KEDSVAAFYAEKSIFI----------------TGGTGFLGKVLIEKLLYSCKA
VDQFY-----VLIRKKKDQTPSERIAQLLESE--LFSRLRKDD---PSAL-KKVVPVVGD
LTMPNLGLSAAVEDLIVSKVSVIFHVAATVKFNERMKNALANNVEATREVINLCHRLEK
-
VD---------AFIHVSTAYSN--TD--QKVV-------EERVYPPPAPLSEVYAFVKN-
--YGDDMDIIQNLLNGRPNTYTYTKALAEDIVLKEHGG--IPTAIIRPSI----------
---------------VLSVL--KEPIPGWLD--NWNGPTGLLHASSQGVHCSMLGSGSNV
ADLIPVDIVTNLMIVVASRC------K-KSNG-------LKVYNSCSGTTNPITYQAFTK
MFL-DSCISRGW--NKVP-FPMLI-FVKWAFLNRVLKFLLVIVPFFLIDVYLRFFGK-PN
----------YMRMITYTKKAEDLMTFFTSHEWQFKDGNVRDLINMMS-PEDRKIFYCDP
VEIQWKP--YFDDYCVG---VFKYLLKR-----KV-------------------------

>Yev_pgFAR

--MVQL-KEDSVAAFYAEKSIFI----------------TGGTGFLGKVLIEKLLYSCKA

VDQIY-----VLIRKKKDQTPSERIAQLLESE--LFSRLRKDD---PSAL-KKVVPVVGD
LTMPNLGLSAAVQDLIVSKVSIIFHVAATVKFNERMKNALANNVEATREVINLCHRLEK-
VD---------AFIHVSTAYSN--TD--QKVV-------EERVYPPPAPLSEVYAFVTN-
--NGDDMDIIQNLLNGRPNTYTYTKALAEDIVLKEHGG--IPTAIIRPSI----------
---------------VLSVL--KEPIPGWLD--NWNGPTGLLHASSQGVHCSMLGSGSNV
ADLIPVDIVTNLMIVVASRC------K-KSNG-------LKVYNSCSGTTNPITYQAFTK
MFL-DSCISRGW--NKVP-FPMLL-FVKWAFLNRVLKFFLVIVPFFLIDVYLRFFGK-PN
----------YMRMITYTKKAEDLMTFFTSHEWQFKDGNVRDLINMMS-PEDRKIFYCDP
VEIQWKP--YFDDYCVG---VFKYLLKR-----KV-------------------------

>Dpl-ctg1

---MASCLGG
HFGLNQ-DYIPVADFYADKSIFV----------------TGGTGFMGKVLVEKLLRSCPK
IKKIY-----LLMRPKRGQDVASRLTELTQSP--LFESLRKER---PQEL-NKIVPIVGD
ITEPELGISPADQEMLCQKVSVVFHSAATVKFDEKLKLSVTINMLGTQQLVQLCHRMLG-
LE---------ALVHVSTAYCN--CE--RERV-------EETVYSPPAQPEHVVTLVQTL
N-DELVDRITPDLVGDRPNTYTFTKALAEDMLIKECGN--LPVAIVRPSI----------
---------------VLSSV--REPVKGWVD--NWNGPNGIIAAVGKGVFRTMLGNGTRV
ADLVPVDTVINLMIVCAWRTHL----R-RGDG-------VVVYNCCTGQQNPITWQRFVK
TSF-KYMRKHPF--SEVVWYPGGD-ITSSRFQHGILSLLQHRLPAVLIDLVARITGSKPV
----------MVRVQNKLEKASACLEYFTTRQWAFADNNVQALCRSLS-PEDRDTFDFDV
TNINWDG--YIESYVLG---IRRFLFKE-----SPHTLPKSRTIMRRLHIVHVLGQVLAV
LFLWRFVFLRSASLRSVWRSIVELLTRAARMLAVA-------------------------

>Bmo_swdb12

---MASCVGA
NYSSGS-DYTPVAEFYAEKSVFV----------------TGGTGFMGKLFVEKLLRSCPK
IKKIY-----LLMRPKRGQDVASRLTELTQSP--VFETLRKER---PQEL-NKIVPIVGD
ITEPELGISQTDQMLLCQMVSAIFHSAATVKFDEKLKLSVTINMLGTQQLVQLCHRMLG-
LV---------ALVHVSTAYCN--CE--RERV-------EETVYAPPADPGHVVALVQTL
P-DELVDRITPHLVVHRPNTYTFTKALAEDMLIKESGN--LPVAIVRPSI----------
---------------VLSSV--REPVKGWVD--NWNGPNGIVAAVGKGIFRTMLGSGDKV
ADLVPVDTVINLMIVCAWRTHL----R-RDQG-------VVVYNCCTGQQNPITWKRFVK
SSF-KYMRKHPF--TEVVWYPGGD-ITSNRLKHNVLTLMQHRAPAVLMDLVSRTTGNKPM
----------MVRVQNKLEKAAACLEYFTTRQWSFADDNVQALCLLLS-PEDRRTFDFDV
KNIDWDA--YIESYVLG---IRRFLFKE-----SPETLPKSRSLLKRLHIIHILTQVAAV
FFIWRFLFSRSEVLRGLWRRIVDLLTRAARLLAIA-------------------------

>Dpl_FAR3

--MSLD
DNDLDS-LPDRIAETFAGLTLLV----------------TGGTGFMGKVLVEKLLRKCPD
IAKIM-----LLVRPKKGKSPKQRLEEMLNDE--LFAKLRSLRGGVEPLL-EKLQIVTGD
VSAPDLAISDTDRLDVIENVHIVVHAAATIRFDEELKKAVFLNVRGTKLILDLAKQCKK-

LK---------LFIHISTAYCH--LH--EKLL-------EEKPYPPPANPHKIIEAMEWM
T-DEAVATITPNLLSKLPNSYAFTKALGEALAVEAMEH--IPVIVLRPSI----------
---GAGKGVIRTMYCKSNSY
ADYLPVDVFINGIMICVWNYIK----L-GDTK-------SNVINFTSSAEIKVTWLEMID
AGREIIMNRVPL--NNVVWYPGGS-MKHSRLYHNICALFFHWIPAIIIDTLLFCLGYKPV
----------LMRVHRRISKGFEVFEYYTNNQWDFKSDIAQTVRQKLN-PRERRDYKVDA
IGLDISK--YFEDCIRA---ARIFILKE-----SDDTLPSARRHMKIMWFVDIVAQFLFW
ALMMYWISGWMASFYSFVVGSSNITPKAIVE-----------------------------

>Hmel-ctg10

---MASEGYS
SSELES-MPDRIAETFTGLHLLV----------------TGGTGFMGKVLVEKLLRKCPD
IGKIM-----LLVRPKKGKTPKQRLEEMLNDE--LFGKLRALRGGVEPLL-EKLEIVNGD
ASAPDLAISEADRQTIIDNVNIIIHAAATIRFDEELKKAVLLNVRGTKLVFELGKKCKN-
LQ---------LFIHISTAYCH--LH--EKLL-------EERPYPPPADPHKIIDAMEWM
D-EETIATITPKLLSKLPNSYAFTKALGEALAVEAMEH--IPVIILRPSI----------
---------------VIPIW--QEPLPGWTD--NINGPTGLLIGAGKGVIRTMYCKSNSY
ADYLPVDVFINGIMICTWNYIK----N-GDTQ-------RTVVNFTSSAEIKVTWLEMID
AGREIIMKRVPL--NGVVWYPGGS-MKHSRLYHNICALFFHWIPAILIDTLLFCLGYKPV
----------LMRVHRRISKGFDVFEYYTNNQWDFKSDIAQTVRKSLN-PKERRDYKVDA
IGLDILK--YFEDCIKA---ARIYILKE-----TDDTLPAARRHMLM-------------

>Ban_EST6

---MSSEGYT
SSELES-MPDRIAETFSGMNLLI----------------TGGTGFMGKVLVEKLLRKVPD
IGKIM-----ILVRPKKGKTPKQRLEEMLNDA--LFEKLRTLRGSIAPLL-EKMQIIDGD
MSAPDLAISEANRQMIYDEVDMIIHAAATVRFDEELKKAVLLNVRGTKLILEIAKKCKN-
LK---------LFIHISTAYCH--LH--EKLL-------EERPYPPPADPHKIIDAMEWM
D-DDTIATITPKLLSKLPNSYAFTKALGEALAVEAMEH--IPAVILRPSI----------
---------------VIPIW--QEPVPGWTD--NINGPTGLLIGAGKGVIRTMYCKGNSY
ADYLPVDVFINGIMIVAWHYL---------------------------------------

>OnuZ3

---MSSEGYS
ASEVDA-MTDRIAATYAGLSVFV----------------TGGTGFMGKVLIEKLLRKCTE
IRQIY-----LLVRPKKGKTPKQRLEELFQGE--LFEQLRNLRGGVEPIL-EKVTLISGD
VSEPDLAMSEADRQLLIENVDIIIHAAATIRFDEELKKAVLLNVRGTKLIVELAKTCKK-
LK---------LFIHISTAYCH--LH--EKLL-------EEKPYPPPADPHQIIQAMEWM
D-EETVAQLTPKLLDKLPNSYAFTKALGEAVVVEAMEADLIPAMILRPSI----------

---------------VIPIW--QEPLPGWTD--NINGPTGLLIGAGKGVIRTMYCKSNSY
ADYLPVDVFINGIMISAWNYIT----Y-GDKK-------SRVVNFTSSAEIKVTWLEMID
AGREIIMNRVPL--NGVVWYPGGS-MKHSRLYHNICALFFHWIPAILIDILLFCLGYKPI
----------LMRVHRRISKGFEVFEYYTNNQWDFKSDIAQTVRLRLN-TRERRDYKVDA
VGLDIMK--YFEDCIKA---ARIYILKE-----YDDTLPAARRHMRVMYWVDVIAQCLFW
GLILYWISGWFPSITSVFATSDPAPTVMAA------------------------------

>Her_EST1

---MASEGYS
PSELES-MPDRIAETFTGLHLLV-----------------TGGTGFMGKVLVEKLLRKCPD
IGKIM-----LLVRPKKGKTPKQRLEEMLNDE--LFGKLRALRGGVEPLL-EKLEIVNGD
ASAPDLAISEADRQAIVDNVNIIIHAAATIRFDEELKKAVLLNVRGTKLVFELGKKCKN-
LQ---------LFIHISTAYCH--LH--EKLL-------EERPYPPPADPHKIIDAMEWM
D-EETIATITPKLLSKLPNSYAFTKALGKALAVEAMEH--IPVIILRPVY----------
---------------WXYHT--GRTVTGLEG--KSNGATGMLMGGCKGFXEAVFRKG---

----------------------------TRNQLDAAKDIGAAIKTH--------------

>Bmo_swdb6

---MSSEGYS
AEEVDS-MPDRIAASFSNMTVLI-----------------TGGTGFMGKVLVEKLLRKCDD
VKNII-----LLVRPKKSKNPKQRLQQMFNGE--LFEQVRNMRGGVQGLL-KKVCLVSGD
ASAPDLAISPEDRQSLVENVDIIIHAAATIRFDEELKKAVLLNVRGTKLMVELAKECKK-
LQ---------LFIHISTSYCH--LH--EKLL-------EEKPYPPPADPHQVIQAVEWM
D-EETIAAMTPKILNKLPNSYAFTKALGEALAVEAMDH--IPVMILRPSI----------
---------------VIPIW--QEPIPGWTD--NINGPTGLLIDD-----QCVFTQLNL-
--LGPV--MVNLSIKDLRGHIA----R-PDKK-------SSVINFTSSAEIKVTWSEMID
AGREIIMNRVPL--NGVVWYPGGS-MKHSRLYHNICVFFFHWIPAVFIDILLFCLGYKPV
----------LMRVQRRINKGFEVFEYYTNNQWDFKSDIAQTVRQRLN-TRERRDYKVDA
GGLDISK--YFEDCIRA---ARIYILKE-----YDDTLPAARRHMKVMWFVDLVTRCLFW
GFVFYWLYSWTTSFGSSATTSNVFPAAVVMES----------------------------

>Dpl_FAR4

MQFIGD-MSRSVTEFYDGKCIFV-----------------TGATGFVGKVLVEKILRSLPG
VKNVY-----LLMRQKKGTSGDDRLKDLWNSR--IFDNLRANN---PDAF-NKIKLISGD
LLKEDLGICNDDRGVIQENCNIVFHSAACVRFDQKLKDAVETNTTATLRLLKLAETMKF-
SK---------SQLNPYKIFAS--NF--ICLI----------------------------
----------------PPTKHSS-------------------------------------

>Dpl-ctg6

----MT-PSIGVAEFYKNKTIFV----------------TGGTGFLGKVLVEKLLRCCPD
LKRIY-----LLMRPKKGLNVNERIDDYFNCR--VFEIIHEKS---PKIF-DKVTVIPGD
ILQHNLGISIEDWEKLQRETEIVFHCAACVRFDMPIRDAVNLNTLGTDRVLKLADDMEK-
LE---------VFVHVSTSYCR--SD--LQKL-------GERLYPAKHRPQDIIDIVKWM
D-DELLTLIQPKLIEPQPNTYSYTKSLSEDLVAQKAGK--YPIVIARPSI----------
---------------VTAAE--KEPLPGWVD--NMNGPTGLLVGCGKGVIRTMHCHASYQ
ADAVPVDYVANACVLLGYLTAI----D-KPKE-------IRVCNITQSDQNPITWDEAIN
LGR-VHLHEFPS--SVCLWYPGGS-TKNSKLHHMIASFFFHLLPAYFIDLLMLLMGKKTF
----------MVKVQKRVSYGMKVLEYYTTNEWFFENDFYKSLKTRIS-KQDNEVFYTDF
STFNWSD--YMRKYMKG---AREYCCKE-----DPSTLPQARKLLNR-------------

>Hmel-ctg11

----MA-PSIGVPEYYVGKNIFI----------------TGATGFMGKILIEKLLRSCPD
LNKIY-----FLMRPKRGQSVKERLDDFINCK--VFDSLHDKS---PKCF-DKLHVISGD
ILQDDLGISIEDWDLLQKEIQIVYHCAACVRFDMFIRDAVNMNTLGTMKVLKLTDGMKK
-
LE---------VFVHVSTAYCR--CE--VHTL-------EERLYPANHRPQNVMECIKWM
D-DDLLSYLQPKLIEPQPNTYAYTKSLTEDLVSQHVGK--YPIVIARPSI----------
---------------VIASH--KEPLPGWVD--NMNGPTGLLIGAGKGVIRSMHCNDSYL
ADVVPVDIVVNACILLSFITAM----E-KPKE-------IKICNITQSGYNPITWGEALD
MGR-VHVQEFPF--SVCLWYPGGS-PKSSKVHHLIAAFFFHLLPAYLVDMMCVLMGKKTF
----------MIKIQKRVNYGLEVLQYYTTKEWFFKNDYFRSLPSLVS-KADNETFYTDV
HV------------------SVSFLIIK---IS---------------------------

>Dpl-ctg11

----MA-PSIGVAEFYKHKTIFL----------------TGGTGFMGKVLVEKLLRCCPD
LNKIY-----LLMRPKKGQSTKERLDDYFNCR--VFDNLQERS---PKCF-DKLAVIPGD
ILQEDLGISIEDWDKLQRETEIVFHCAACVRFDMPIRDAVNMNTLGTNKVLKLADGMVN
-
LE---------VFVHVSTSYCR--CE--VHTL-------EERLYPAKHRPQDVMECVKWM
D-DELLTYLQTKLIEPQPNTYAYTKSLTEDLVSQKAGK--YPIVIARPSI----------
---------------VTAAH--KEPLPGWVD--NLNGPTGLLPQKSAGA----------K
GKGKPLQLSLPMKVIM-------------PKE-------VRICNVTQSGHNPITWGEALD

MGR-VHVQEFPF--SVCLWYPGGS-AKSSKVQHLLALFFTHLLPAYFVDLLMFLMGKKTF
----------MVKIQKRVSYGLNVLQYYTTKEWFFDNDYYRSLSKRIS-KDDNDVFYTNL
KTINWSA--YIRNYIKG---AREFCCKE-----DPSTLPQARKLQRQLFWLDKAVQVVIY
FLIAYFIYYYIIRFFVM---

>OnuZ4

----MA-PSMTVPEYYAGKTLFI----------------TGSTGFMGKVLIEKLLRSCPD
IKKIY-----LLMRPKKGHGSKERLDGFMNCR--VFDKLKSEH---PEQF-HKLQVVPGD
ILMEDLGLSVEDRDTLQRECQVLMHCAACVRFDMFIRDAVNMNTVGTKRVLDVASGM
KQ-
IE---------VFVHVSTAYCR--CE--VEVL-------EERLYPAKHRPKHVIETVNWM
D-DELLTHLQPKIIEPQPNTYAYTKSLTEDLVAQHAGK--FPIVIARPSI----------
---------------VTAAY--KEPMPGWVD--NLNGPTGLLVGAGKGVIRTMHCNENYA
ADIVPVDMVVNACILLGYTTAL----E-KPKE-------VQFCNIAQSGINGFTWGEALA
LGR-IHVMEYPF--SICLWYPGGA-PSASRLRHQVTLFLTHTLPAYTVDLLARALQQKPF
----------MVKMQKRIQSGLEVLQYYTTKEWHFRNENLRALRTKVT-EEDDETFYTDL
TVIDWNT--YIRDYIKG---AREFVMKE-----DPSTLPQARRLNRQLYYLDTFTKIFIY
SIIASSWMKRSISNILKMFWAA--------------------------------------

>Bmo_swdb5

----MA-PSASVAEYYAGKSIFI----------------TGATGFMGKVLVEKLLKSCPG
LNKLY-----LLMRPKKGQSSKERLDDFLGFK--LFGPLQKEN---PKCF-EKLHVVPGD
ILMDDLGISIADRELIQRECQVIFHCAACVSFGPELKKIVKLNTVATKRVLELAKDFNN-
LE---------AFIHVSTSYCR--CE--LDLL-------EEKLYPSPHRPEHIMDCMRWM
D-DKLVAHMQPK--

-----------------------------PQK-------IQICNITQSGLNPITWGEALD
MGR-VHVREYPF--SVCLWYPGGS-PKKTRIQHEIALFFTHLLPAYFVDLLLFLLGKKTF
----------MIKVQKRINYGLEVLQYYTTKKWLFKNDNFLALRTKIS-AEDDRIFYTDI
QEIHWND--YIREYIKG---AREFCCDE-----DPSTLPQARRLQKQ-------------

>Hmel-ctg12

-------MQPSVTEYYEGKSVLI----------------TGATGFVGKTLVEKLLRSCPG
VKTIY-----LLMRKKKGVSSQERIRDLCSNI--IFDHLREKQ---PEAF-EKIKLVAGD
ILEEGLGISNDDRQELQKNCNIVYHSAACVRFDQKLKIAIHINTVGTERVLKLAETMEN-
LE---------SFLHLSTAYCR--CD--LPVL-------DEFLYPAVHDPNKIMELVEWM
D-DDTLDFLEPRLISSEPNTYSYTKAITEDLVAEYSPK--LPIAIGRPSI----------
---------------VTASW--KEPMPGWVD--NLNGPTGLLTASGKGVLRTMLCNPSYT
ADAVPVDVVVNGCILIAYLTAI----D-KPKK-------MPIYNITLSGVQRLTWGEIIE
LGK-HWVLKYPY--TVALWYPGGN-IKSNRLYHYTMVFLTHLIPAYLIDMLLFLLGRKTF

----------MVNVYKRISHGMKVLQFYTTKEWHFRNSNFLSLKNRIN-KNENEIFYTDL
SELNLDA--YLKDYILG---TRKYIVKE-----DESTLPRARKLLKM------------

>Ban_EST4

-------MAPTVAEFYEGKHVFI----------------TGATGFVGKALVEKLLRSCPG
IQTIY-----LLMRPKKGMSGEDRLKELCNNL--IFDYLRDKQ---PECF-MKVKLIAGD
VLEEELGISNDDRQELQKNCNIVIHSAACVRFDKNLKDAVHMNTRGTLRVLKLAETMEK
-
IE---------AFVHLSTAYCR--CQ--LDEL-------EEKLYPAAHDPEKLMDLLEWL
D-DDTLAYLEPKLISSEPNTYSYT------------------------------------

>Ban_EST1

-----M-TSMGVREFYAGKKIFI----------------TGATGFMGKVLVEKLLRSCPD
VETIY-----ILVRRKKGQSPEARLEAFAKCG--AFEKLLEQD---AKAF-RKVRLVPGD
IPEDNLGIPDIIEQELQKECQIVVNNAACVSFNLPVRKAVQMNTMGTQKVLQLAEKMKK-
LE---------VFLHVSTSFCH--DY--IDVL-------EEKIYKAKHNPLDIINMLKWM
D-DDTLAALQPELIKPYPNTYGYTKSLSEDLVSQYSGK--FPIALA-------------

>Dpl-ctg17

-----------------MDLML-------------------------------------
------------------------------Q--LFEKLLEMN---PKVV-DKIKVINGD
IMEEGLGLSPQHLEELRNECQIVFNNAASVSFNLSLKEAVKTNVMGTQKVLALADTMKK
-
LE---------AFVHVSTSFCH--DH--LKVV-------EEKVYPAPHSPKNVIDIISWM
D-DETLATLEKKLVSPFPNTYGYTKCLTEQLISDHGAT--YPIAIARPSI----------
--------------VIPAF--KDPMPGWVD--NVNGPNGIMYAASRGVLHTIYCKQTTK
TDSIPVDMAINGLIILGCLTATE-------------------------------------

>Dpl-ctg5

-----M-TTDSIREFYKSKNVLV----------------TGGTGFMGKVLIEKLLFSVPD
IGNIY-----VLMRPKKGKSVNQRYEDMQRLP--IFDRLRNTK---PSSL-KKIVPLTGD
VLFDDFGLSESDMQKISEDVSIVFHFAATLKLEAPLYENVNMNTCGTQRALNVAKKLKN
-
LR---------LFIHLSTAFCY--PD--YAVL-------EEKMHAPPVKPSDIMHLLEWL
D-EKKVAILTPSLLGPHPNCYTFSKRLAENIVENEYEN--LPAVVVRPSI----------
---------------VCPSI--KEPVPGWVD--SLNGPVGLMLGAGKGVIRSMLCDGSLI
AQVVPVDTCINALIAIGMIEGKRE--D-KAEL-------MPVYNVNIGHQKPTTWGEVLQ
IGK-DYGRKYPL--AWPLWYPNGD-ITTNYVLHEFKRLFYHLLPAYCIDLLLFLLRQPRF
----------MVRVQDRISQGLQVLQYFTMRPWTFPCPNFDSIQSKLD-KEEQVIFNTDL
TTADRDA--YLQQCIEG---GRIFCLKE-----DPSKIRINRAYHNFLFVLDCLVKILFW
LLLLSFFAAYFTPLRDVLSYGEPVVKYLPFVGRVVCDKE---------------------

>Bmo_swdb8

----MS-SATSVRDFYAGRNILV----------------TGGTGFMGKVLVEKLLYSVPE
IGNVY-----ILMRPKKAKTVAQRLEDLQRLP--LFDRLKNER---PNAL-NKIKAIQGD
VLFEDFGLSNLEIENLSKEVTVVFHFAATLRLEAPLKDNVNMNTSGTLRTIKVAKKLKN-
LQ---------IFVHLSTAFCY--PD--YEVL-------GEKLYAPPAKPEHVMRLIEWL
D-DRQLTLLTPSLLGPHPNCYTFSKRLAENVVEQAYKD--LPIVIARPSI----------
---------------VCPSY--SEPMPGWVD--NLNGPVGVMIGAGKGVIRTMLCDGTLS
AQVIPVDIAINAIIAIGMIEGTKK--E-KSLT-------IPVYNVNIGHQKPMTWGDVLT
VAK-DYGRKYPL--SWPLWYPNGD-ITTNYTLHEIRRIFYHLVPAYCIDFLLFLMGQKRF

---LFVVHWVCKILFW
LLILSFVAAYFEPVRYVLSQGEPVVKYLPFVASAVFKN----------------------

>Hmel-ctg9

-------MASEVNEWYRGRKVLV----------------TGASGLMGKVLIEKLLYSVPD
IGCVY-----ALVRSKRGKSPESRIEDMWKLP--LFSRIREEK---PHVM-KKLVPVVGD
ILFDNLGIEKNQLEEIYNEVSIVFHFAATLRLEAPLKEGLEMNTKGTLRVLEIAKRMKN-
LV---------SFVHLSTAFCY--PD--HGRM-------AEKVHDPPADPHEVLRAASWL
S-EEQLNLLAPSMMQQHPNSYTYSKRLAEALVKESYAE--IPTVVVRPSI----------
---------------VTPAY--KEPTPGWVD--NLNGPIGLMIGAGKGVIRTMHCYGHYH
AEVIPVDIAINAIILIPYYINTKI--E-RPAD-------IPVFNLTSGDDRDTTWKEVLD
VGK-ATVRKFPF--EGPLWYPNGN-IRHNKLIHELCVFFYHIIPAYLIDFLMLIFGQKRF
----------MVRIQNRISVGLEVLQYFTTREWWFDTHNFKALATKLN-QMDFATFPMNL

AIIEVGP--YIETCMIG---GKLFCLKE-----KLENLPKARLQNNM-------------

>Her_EST2

-------MASEVNEWYRGRKVLV----------------TGASGLMGKVLIEKLLYSVSD
IGCVY-----ALVRSKRGKSPESRIEDMWKLP--LFSRIREEK---PHVM-KKLIPVVGD
ILYDNLGIEKNQLEEIYNEVSIVFHFAATLRLEAPLKEGLEMNTKGTLRVLAIAKRMKN-
LV---------SFVHLSTAFCY--PD--HGRM-------AEKVHDPPADPHEVLRAASWL
S-EEQLNLLASSMMQQHPNSYTYSKRLAEALVKESYAE--IPTVVVRPSI----------
---------------VTPAY--KEPTPGWVD--NLNGPIGLMIGAGKGVIRTMHCYGHYH
AEVIPVDIAINAIILIPYYINTKI--E-RPAD-------IPVFNLTSGDDRDTTWKEVLD
VGK-ATVRKFPF--EGPLWYPNGN-IRHNKLIHELCVFFYHIIPAYLIDFLMLIFGQKRF
----------MVRIQNRISVGLEVLQYFT-------------------------------

>Ban_EST5

-------MASEVNEWYRGRKVLV----------------TGASGLMGKVLIEKLLYSVPD
IGCVY-----ALVRSKRGKTPESRIEDMWKLP--LFSRIREEK---PHAM-KKLVPVTGD
IMYDELGIEKAQLEEIYDEVSIVFHFAASLRLEAPLREGLEMNTKGTLRVLDMAKKMKK-
LV---------SFVHLSTAFCY--PD--YERM-------AEKVHDPPANPHEVLRAASWL
T-DDQLNALAPSLMQLHPNSYTYSKRLAEALVRESYAE--IPTVVVRPSI----------
---------------VTPSH--KEPTPGWVD--NLNGPIGLMIGAGKGVIRTMHRYGHYH
AEVIPVDIAINAIILIPHYIHTKI--D-RPTE-------IPVFNLTSGDDRDTTWKEVLD
VGK-ATVRKFPF--EGPLWYPN--------------------------------------

>Bmo_swdb7

-------MTSQINEWYKGRKVLI----------------TGSSGLMGKVLIEKLLYSVPD
IGCVY-----ALVRSKRGKSPESRIEDMWKLP--LFTRIREEK---PHVM-KKLVAVPGD
IICDKLGIEKTKLEQIYNEVSVVFHFAASLRLEAPLKEGLEMNTKGTMRVLELSKNIKN-
LV---------AFVHLSTAFCY--PD--YERL-------AEKVHDPPTDPENVLQAASWL
T-EQQLNILAPSIYKKHPNSYTYSKRLAEALVKESYPQ--LPAVVVRPSI----------
---------------VTPAY--KEPIAGWVD--NLNGPIGLMVGAGKGVIRSMHCYGHYH
AEVIPIDIAINAIIVISYKIGNQS--E-RPPE-------IPTFNLTTGDDRNTTWKEVLD
IGK-ATVRKYPF--EGPLWYPNGN-IRHNKFIHDLCVFFYHLVPAYFIDFLMFLFMQKRF
----------MVRIQNRISVGLEVLQYFTTREWWFDTDNFKALATMLN-PVDKEVFPMDL
SVIEDEP--YIESCMIG---GKLYCMKE-----KLENLPKARLQNNILYVLDKITTVFFY

LILLYWVVLYCAPVREVLSYGGPFVRYLPLVVILKCNCNKCNCKKRHAESEPKIEAAHF
G
TIIWSLAMIKIKCLIL
>Dpl-ctg9

--MTFL
EDRDLD-NVPTIPEYYRGKVVFV----------------TGGSGFMGKVLIEKLLYSCPD
LEKIY-----LLLRPKKGVQPEERLSAIYSSH--CFDRLRNER---PGVFDSKVFFVAGD
VSDIGLGLSEDDRAILLKQTQILFHVAASVRFDDPLKVAVRLNLFGTKQVVDLAKDMKN
-
LE---------SFVHVSTSYAN--TN--REVI-------EEILYPALGDWRETLEICETA
D-DHTLKVLTPKFIGELPNTYVFTKQLAEHVVYEQKGK--MPIVIVRPSI----------
---------------VISSH--LEPVPGWID--NFNGPAGLMAASGKGILRTILSSPEIV
SDYIPVDVAIKACIVASWVRGTKKL-E-ATDD-------IPIYNDCVGKLNHMTMQDMVS
VGS-EIAQILPL--KNALWAYGVS-ITTSKFKYNLEVLLFHMLPALLVDMILWIIGKNPM
----------LVRVQRRIYTANMALHYYLTKQWTFSNRNLIALRSKIK-DDDMRDFYYEI
ENIDKYE--FYKQCCFG---GRKYLLKE-----TDDSLEESQAQYKRMVVLDKLVKMTFY
GCLIWWIMRREFIRDFIQIIH---------------------------------------

>Dpl-ctg14

--FMGKVLIEKLLYSCPD
LEKIY-----LLLRPKKGVQPEERLSAIYSSH--CFDRLRNER---PGVFDSKVFFVAGD
VSDTGLGLSEDDRAILLNQTQILFHVAASVRFDDPLKVAVRLNLFGTKQVVDLAKDMK
N-
LE---------SFVHVSTSYAN--TN--REVI-------EEILYPALGDWRETLEICETA
D-DHTLKVLTPKFIGELPNTYVFTKQLAEHVVYEQKGK--MPIVIVRPSI----------
---------------VISSH--LEPVPGWID--NFNGPAGLMAASGKGILRTILSSPEIV
SDYIPVDVAIKACIVASWVRGTKKL-E-ATDD-------IPIYNDCVGKLNHMTMQDMVS
VGS-EIAQILPL--KNALWAYGVS-ITTSKFKYNLEVLLFHMLPALLVDMILWIIGKNPM
----------LVRVQRRIYTANMALHYYLTKQWTFSNRNLIALRSKIK-DDDMRHFYYEI
ENIDKYE--FYKQCCFG---GRKYLLKE-----TDDSLEESQAQYKR-------------

>Hmel-ctg15

--MTFL
EDRDFD-DVPTIPEYYRGKTIFL----------------TGGSGFMGKVLLEKLLYSCTD
LDRIY-----LLLRNKKGVPPEHRLAAIYKSP--CFDRLRNEK---PGVFDSKVFFIAGD
VSEIGLGLSDDDRTLIINRTHIIFHVAASVRFDDHLKAAIKLNLRGTKQILELATEVRN-
LE---------CFTHVSTSYSN--TN--RELV-------EEIMYPPLADWRDTLKICDEL
D-DRVLQVLTAKYIGEMPNTYVFSKQLAEHVVYEYKGK--LPVVIIRPSI----------
---------------VVSSV--KDPIPGWIE--NFNGPTGIIVACGKGILRTIYSKPDII
SDYIPVDVAIKCFIASTWSRGTKKL-E-PTDD-------VPIYNSCAGSLNNITVHELIS
IGK-QIIASMPL--SNMIWCFGAT-ITSSLFIFNVQVLFLHLLPAVIIDMLLWIFGQKTM
----------LVKIQRRIYVANLALHYYTIHQWTFSNVNFVTLRSKIK-DKDQEDFYYDL

ENVDRYE--FFRQCCNA---GRKYLLHE-----TEDDIPKNRAHYARYLFLLYDCKHDRF
WVLFPLENIKYLTFSFLRSVSRGKVPR---------------------------------

>OnuZ1

--MGFL
EERNFE-DVPEIPEYYKGKTIFI----------------TGGSGFMGKALIEKLLYSCSD
LDRIY-----LLLRTKKGVKAEDRLAELYSTG--CFDRLREEK---PDIFSSKVFVVAGD
VMDPGLGLSEEDRALLVNRVNIIFHVAASVRFDDPLPFATKLNLGGTKEVVTLAKEVRN-
LS---------ALVHVSTVYSN--TN--RDVI-------EEVMYPPHADWKDTLEVCDVI
D-EHSLRVLTPKYLGDIPNTYTFTKQLAEHVVYEQKGQ--LPAVIIGPSI----------
---------------VISSV--EEPVPGWIE--NFNGPVGMLVACGKGIMRSIYTDPNLI
SDYMPVDVSIKSIVTAAWIRGTKKL-E-PPDD-------VQIYNCCAGKLNNITMQELVD
IGK-RLAASLPL--NDTLWNIGGS-ITTSRTIHYIKVLILHCLPAIFVDAILLLLGKKPL
----------LLKVQRRIYIANLALRYYITKEWTFDNTNFIQLRSRIK-EQDKKHFYYDI
ENVDKEE--YFRKSCIG---GRKYLLKE-----KDEDLPKAKAHHKRMVILDKTVQCIFY
GYLFWWVVNTHYIKGLT-DFLYEIFTM---------------------------------

>Bmo_swdb3

--MGLS
EERDMT-HVPTIPEYYKGKSIFI----------------TGGSGFIGKIVIEKLLYSCTD
LDRIY-----LLMRPKKSVSPDKRLAQLYSTP--CFDRLRKEK---PGVFQSKVLVVPGD
VMEDGLGVSDEDRKLLAERVNIVFHSAASVRFDDPLKYAVRLNLNGTKEVVELAKSMH
N-
LS---------SFVHVSTSYSN--TN--REVI-------EEVMYPPHADWRDTLEICEKL
D-EHTLRVMTPKYLGELPNTYTFSKQLAEHAVYEQRGQ--LPAVIVRPSI----------
---------------VIASA--DEPISGWIE--NFNGPVGIFVACGKGE-----------

>Dpl-ctg13

---MGF
LDRDFR-DVPGIAEYYKDKTIFI----------------TGGSGFMGKVLVEKLLYSCPD
LDRIY-----LLLRSNKGVDAEDRLKNLYSSR--CFDRLRNEK---PDVFDKKVSFIAGD
VGELGLGICDEDRALIINRVHIVFHVAASVRFNDSLKVAARLNLRGTREAVELAKEIRN-
LE---------AFVHVSTSYAN--TN--RQCI-------DEVIYPASGDWRDTLEVIENV
D-EHTLNVLTPKYLDKLPNTYVFTKQLAEHVVNEQKGK--LPIVIMRPSI----------
---------------VISSV--REPMVGWIE--NLNGPVAILIASGKGILHTMYTDPNLI
SDYMPVDIAIKAFIAAAWARGTKKL-E-PTDD-------IHLYNCSSSEIKALTMGQIVE
LGM-EISKKIPL--DSLVWHPCGG-LTSSKLVNYVKVLLLHLLPALLVDGILKLIGKKPM
----------LTKVQRRIYVANLALEYYVTQQWTFKNVNIVKLRSKIK-EEDLKEFFYEM
ETIDIHE--YFMNSCYG---GKLYILKE-----KLEDLPAARIHYRRMELLHKVVMIIFK

LSVLWFIYNTSFFRDVM-NMLYAVFVG---------------------------------

>Hmel-ctg16

--MGFL
EECDLN-NVPDIPEYYKGKTLLI----------------TGGSGFMGKVLIEKLLYSCPD
LDKIY-----MLLRSKKGVKPEDRLSAIYSSR--CFDRLRRER---SDIFKKKVFFISGD
VSEIGLGISDDDKALIVRTTNIIFHVAASVRFDDSLKVAANLNLRGTRELVELAKNMKN-
LE---------VLVHVSTSYAN--TN--RQCI-------DEVIYPAFADWRDTLEICEKL
D-NNTLNVLTPKYLGELPNTYVFTKQLAEHVLWEEKGK--LPIIIMRPSI----------
---------------VISSV--NEPLVGWVE--NLNGPVGMLVASGKGILRTLYTDPDLV
SDYIPVDVAIKAFIAGAWIRGTKKL-E-PTDD-------IEVYNCSTSHMKTMTIGEIVE
YGRNMIVHEVPL--EGMLWYVGGS-LTTSRLEYNLKVILLHLLPAIFVDLILWISGRKPM
----------LVKLQRRIYSANLALHYYVTQQWTFKNTTS--------------------

>Bmo_pgFAR

---MSH
NGTLDE-HYQTVSEFYDGKSVFI----------------TGATGFLGKAYVEKLAYSCPG
IVSIY-----ILIRNKKGSNTEERMRKYLDQP--IFSRIKYEH---PEYF-KKIIPISGD
IAAPKLGLCDEERNILINEVSIVIHSAASVKLNDHLKFTLNTNVGGTMKVLELVKEMKN-
LA---------MFVYVSTAYSN--TS--QRIL-------EEKLYPQSLNLSEIQKFAEEH
YILGKDDDEMIKFIGNHPNTYAYTKALAENLVAEEHGE--IPTIIIRPSI----------
---------------ITASA--EEPVRGFVD--SWSGATAMAASTLKGWNYIMYSTGEEN
IDLIPLDYVVNLTLVAIAKN------K-PTKE-------VTVYHVTTSDLNPISIRRIFI
KLS-EFASKNPT--SNAAPFAATTLLTKQKPLIKLVTFLMQTTPAFLADFWMKTQRKEAK
----------FVKQHNLVVRSRDQLEFFPSQSWLLRCERARVLSAGLG-DSGRAVFRCDP
SPIDWDQ--YLPIYFEG---INKHLFKN-----KF-------------------------

>Dpl_FAR1

--------MEVVPSPMNIRNIVLRMCCHPYLRKRERKGREKGKGRKG---------TC-N
VKKIY-----LLLRPKKGKTAIQRLNQILEDP--VYGILRSEQ---PDFA-SKLIPVEGD
VVDLNLGIEEESRKKIIEEVNIIFHGAATINFEETIKIAALTNIRGTREILNLAKSCKQ-
LK---------GPLNLQLGRKS--LA--LLKLLSLHHGRGTRTVESSRRLDQLVTTEDVE
E-EGTRVQINAKK---
---------------ILSNI--


```
>Dpl-ctg7
------------------------------------------------------------
------------------------------------------MEIARRIEEKGLEKSLRV
KQALES-GNSEVVTVYDGAVLFI----------------TGGSGFIGKQLVEKILRTC-N
VKKIY-----LLLRPKKGKTAIQRLNQILEDP--VYGILRSEQ---PDFA-SKLIPVEGD
VVDLNLGIEEESRKKIIEEVNIIFHGAATINFEETIKVAALTNIRGTREILNLAKSCKQ-
LK---------SLVHISTAYAH--AT--RSRI---KTEIKEDFYDSPLPPDALIQLAEDL
E-NEQLEKVIEPLRRDWPNTYTFTKAITEELVRQTATD--LPVCIVRPAI----------
---------------VISAY--KEPVPGWVDIKNAYGPSGMVLGVSLGVTHTVHADEDIM
LDFVPVDIVNNALIVAAWTTHQSYI-A-GEKQ-------IKIY-FVTGHRNPIYYRDVVN
VVK-EQARPLVS--PKAIWHSFAV-VTKYKLIYLLLTWLLHYIPGYIIDGVCVMIGEKPQ
----------FIKVYKKVYSVSSVFVYFTNNDWVFLDDNALRLYDQLN-SADKELFTCDM
QQVDMPA--MLMTWFYG---VSKFIIKD-----DVTQYEYAVRKQWWLRIANVMFLTLYF
YALYKLVAVTFACLFYFFNLSAGVYQTSV-------------------------------
----------------
>Dpl-ctg15
------------------------------------------------------------
------------------------------------------------------------
--------------------------------------------------------TC-N
VKKIY-----LLLRPKKGKTAIQRLNQILEDP--VYGILRSEQ---PDFA-SKLIPVEGD
VVELNLGMEEESRKKIIEEVNIIFHGAATINFEETIKIAALTNIRGTREILNLAKSCKQ-
LK---------SLVHISTAYAH--AT--RSRI---KTEIKEDFYDSPLPPDALIQFAEDL
E-NEQLEKVIEPLRRDWPNTYTFTKAITEELVRQTATD--LPVCIVRPAI----------
---------------VISAY--KEPVPGWVDIKNAYGPSGMVLGVSLGVTHTVHADEDIM
LDFVPVDIVNNALIVAAWTTHQSYI-A-GEKQ-------IKIY-FVTGHRNPIYYRDHQL
HQF-LVARPLVS--PKAIWHSFAV-VTKYKLIYLLLTWLLHYIPGYIIDGVCVMIGEKPQ
----------FIKVYKKVYSVSSVFVYFTNNDWVFLDDNALRLYDQLN-SADKELFTCDM
QQVDMPA--MLMTWFYG---VSKFIIKD-----DVTQYEYAVRKQWWLRIANVMFLTLYF
YALYKLVAVTFACLFYFFNLSAGVYQTSV-------------------------------
----------------
>Dpl_FAR5
------------------------------------------------------------
-------------------------------------MANQCGETKTLGIENKHSVGCNM
ETDKEVPKMSEVQNFYNGKNVLI----------------TGATGFLGKILVEKLLRSCSG
VENLY-----LLVRQKRGKDIYTRIEEIFEDP--VFDRLKAEV---PKFR-HKIVVIPAD
CEAAGLGLTISDRQTLIEKVNVVFHSAATVKFDEHLRAALATNVCAPLYLLGLAREIKK-
LE---------VFIHISTAYSN--SH--LSFI-------EEKFYPCEANSGKLQKMIDKM
S-DEQINKISPAVLGSWPNTYTFTKALAEKELRENSKN--IPLGIFRPAI----------
---------------VTSTL--KEPLKCWLD--NMYGPTGVAVGTATGMLRTVQCDKTVT
ADLVPVDSVVNCLIVAAYNVHREFVKS-PPQE-------PPIFNYVSSVENRITWGDFTD
LNM-ARIDKHPF--SNAVWYVSLT-LNKSSFVNAVCVLFLHLLPAALVDGLAVCVGKKPQ
----------MLKVYRKIHKFSSVLSYFCTKEIKFCNKRTRELWDNTS-QTDKQIFPFSM
AEVSWSS--YFDDYLAG---IRRYLFKE-----SDDTLPRARRKWTRLYYLHQIVRIVFC
LFAIYVICKLMLSHGNLHSLVYGQSLSLKCLVPDVAVRIKHTYINIKRS-----------
----------------
>Hmel-ctg20
------------------------------------------------------------
```


----------------------------------VFDRLKEEV---PKFR-HKIVAIPAD
CEAAGLGLTISDRQTLIEKVHVIFHSAATVKFDEHLRAALVINVKAPLYLLQLAKDMKG-
LK---------VFMHISTAYSN--CH--LSHV-------EEKIYPCEANSEKLHSIVDKL
T-DNEVNDILPTILGKWPNTYTFTKALAENEIKLNSKN--VPVAIFRPAI----------
---------------VTSTV--KEPLKCWLD--NMYGPTGVAVGTVTGMLRTVQCDPSVT
ADLVPVDSVVNCLMVAARNVSRQYEER-AVIE-------PPIFNYVSSVENRITWGDFTR
LNM-ARIDKQPF--SNAVWYASVT-LNKSTFMNQLYIFFLHLLPAALVDALAICIGKKPK
----------MLKVYRKIHKFSSVLMYFCTREITFCNQHTRSLWEKTS-ESDKQIFPFSM
GEVNWDD--YFDSYLPG---IRRYLFKE-----SDDTVPQARIKWKRYLLLY--------

>OnuZ5

--------------------MAPLLDSTPGDDAAFKIPAMAYSNDNDNDASLKTDNNNDI
DTRNNV-ELTQIQKFYNGKNILI----------------TGATGFLGKILVQKLLRCCPG
VENLY-----LLVRQKRGKDIYTRIEEIFDDP--VFDRLKEEV---PKFR-HKIVVIPAD
CEAAGLGLTLSDRQTLTEKVNVIFHSAATVKFDEQLRLAFNVNVKASLHVLRLARDIKG-
LD---------VLMHISTAYSN--SH--LDQV-------EEKFYPCNMDLETLNQTIEKM
T-DEEINSQLSKLLGEYPNTYTMTKALAENELRINSKG--IPLAIFRPAV----------
---------------VVSSA--KEPVKCWLD--NMNGPNGIVVGSGTGILRTLQCDMQGT
ADPVPVDMVVNCLMAAAVNVNAAYAQSNPPPE-------PPIFNYVSSVENKITWGEFMT
LNM-EQVDKYPF--SNAVWYISLR-LTKSWLLNRFYMFFLHLIPAVLMDGLTVCVGKKPK
----------MLKVYKKIHKFSDVLSYFCTKDIEFCNRRTQELWQKTS-EEDKQLFPFSM
AELDWRD--YFQDYYLG---IRRYLFKE-----NDDNLPQARIKWKRLYYLHQLVTMLFY
FLALYALWSTFSSFLL--

>Bmo_swdb9

---------------MAPVAIEENYIKTNELSTKISRIMEDYKESNYCGDCEHNIYNVNE
DTNQDV-NMSEVQRFYSGKNVLI----------------TGGTGFLGKVLIEKLLRSCPG
VENLY-----LLVRQKKGKDIYTRIEEIFEDP--VFDRLKTQV---PKFR-HKIVVVPAD
CEAAGLGLTLSDRHMLTEKVNIIFHSAATVKFDEHLRAALTTNVRAPLHLLRLARDMRK
-
LD---------VLMHISTAYSN--SH--LPKI-------EEKFYACEPTCQQLETTISKL
S-DEQINAMLPKILGPWPNTYTFTKALAEKEIRVNANG--VPVGIFRPAI----------
---------------VTSTC--KEPLKSWVD--NMYGPTGVAVGSGT-------------

--FPCV--------------

>Yev_FARI
---MSTETVDVANQRNSN
ANMADKKTLAKTTHLSLSNITKIENKLNYFEENLEKSRVCLDCSEKRLADTDQNSNDLEI

TENGRR-SNTDIQEFYNGKSILV----------------TGATGFLGKVLVEKLLRCCPG
VENIY-----LLVRQKKGKDIYTRIDDIFDDP--VFERLKREA---PKFR-HKIVAIPGD
CGAAGLGLTLNDRQTLTEKVNIIFHSAATVKFDEHLRTALVTNVCAPLHMLRLVRDMK
G-
LD---------VLMHISTAYSN--CH--LSQV-------RERFYPCSVDCDQLSGLVDKF
T-DSEIDSLLPTILGSWPNTYAHT------------------------------------
-----------------------------ID--NMCGLRGVVVGAATGVLRTLLCDKEVT
AEIVPVDMVVNCLLASARDVHLSYKQSPPPAE-------PPIFNYVSSVEQRITWGGFME
HNV-EQIGWSPL--NSAIWYFCLT-LNTSPLVNTLYEVFLHLVPAVLIDGLALCVGQSPK
----------MLKLYRKIRKFSSVLSYFSTKEIKFCNKRTRELWERTS-EDDKQLFPFSM
AQMDWSK--YFQGYILG---IRKYIFKE-----EDDSLPQAKRKWTRLYYFHQIVRVIFI
LFAVYLVWHIFSSVF---

>Dpl-ctg16

---MALSEQTDVKGLK
-IEEKM-EESQIQKAFAGSTILL----------------TGGTGFLGKLLVEKLLRSCPD
VKKIY-----LLTRPKKNKDIIKRLQEQFDDC--LYDKLRKER---PDFI-QKIQVVDGD
VSKIGLGINDEDRKNIINEVEYIFHGAATVRFDEALKTAVLINVRGTREMLVLARACTK-
LR---------ALVHISTAYSN--CP--LKEI-------DEKFYESPLSAEKMIDLVESM
D-DKTLNTITPGILGEFPNTYAYTKFLAEDIVQKNSYG--LPVAVNRPSI----------
---------------VVGTA--KEPLLGWID--NVYGPTGVVVGAGVGLIHTLNCDPKAV
ADLVPGDYVVNSVIAAAWKMARDHP-G-NHEEAPMDQL-
PVIYNYVSSEQKPLTWEMFMK
YNE-LYGIETPP--LQAVWAYLLI-LTPNIFFYKFCCLLMHWFPAYVVDGVCFLIGKKLM

>Hmel-ctg14

---MEVHTKTDIKSDK
HIMDNI-EDSQIQKTFAGSTIFL----------------TGGTGFLGKLMVDKLLRCCPK
IKKLY-----LLTRPKKNKEIMKRLQEQFDDV--LYDKLRKQM---PDFM-EKIGIIQGD
MGQPDLGISEEDRNKIINEVEFIFHGAATVRFDEALKTAVAINVRGTREILLLARSCTK-
LR---------AHVHISTAYSN--CP--LREI-------DEKFYDSNFSGDKLIDLVENI
D-EKTLDSITLGLLGEYPNTYAYTKSAAEDIVQKYSQG--LPVAVFRPSI----------
---------------VISTA--HEPVPGWID--NVYGPTGAVVGAAAGLLHVLLCNPSVI
ADLVPGDFVVNGCIATAWKTARDYP-G-NHEEAPVDNL-
PMTYNYVSSEQNPLTWEMFMK
YSE-VYGLRVPS--MQAIWMYLLW-LTPSTVMYNFYFFLLHWIPAYIIDFFLMIIGKKPI
----------LKKAYVKIKKFSAVIGYFAQREWKFHNGNTLKLYKEMD-ETDKQMFNFDM
ESLVWEE--YFQDYVKG---VRIYLLKD-----PLDTIPQSRRKHFKLMLIHYFLLSLIC
FGLFKFVW----FIAKLFI---

>Hmel-ctg17

---MEVQTKTDIKSDK
HIMDNI-EDSQIQAMFAGSTIFL---------------TGGTGFLGKLMVEKLLRCCPK
IKKLY-----LLTRPKKNQEIMTRLQEQFEDV--LYDKLRKQM---PDFM-KKIDLIQGD
MGKPDLDISDEDRNKIINEVEFIFHGAATVRFDEALKTAVAINVRGTREILLLARSCTK-
LR---------AHVHISTAYSN--CP--LREI-------DEKFYDSNLPGDKLIDLVENI
N-KETLASITPGLLGEFPNTYSYTKSAAEDIVRKYSQG--LPVAVFRPSI----------
--------------VISTA--QEPVPGWID--NVYGPTGVAVGAGVGLLHVLLCNPSAI
ADLVPSDFVVNGCIAIAWKTARDYP-G-NHEEAPEDNL-PIIYNYVSSEQNPMTWELFLK
KCE-VHRARAPL--MQATWMYLFW-LTSSEFMYNIYFVILHWVPAYITDFFLVIIGKKPI
----------LKKTYVKIKKFSAVIAYFAQREWKFHNGNILNLYKEMH-ETDKQMFNFDI
ESFVWEE--YFYEYVKG---IRLFLMKD-----SLDTIPQSRKKHFKLMLIHYLLLSLIS
IGLFKFVWCIAIFLCKLFR--------------------------------------

>Hmel-ctg18

-------------MFAGSTIFL---------------TGGTGFLGKLMVEKLLRCCPK
IKKLY-----LLTRPKKNKEIMTRLQQQFED---LYDKLKKQM---PDFM-EKIDIIQGD
MGKPDLGISEEDRNKIINEVEFIFHGAATVRFDEALKTAVAINVRGTREILLLARSCTK-
LR---------AHVHISTAYSN--CP--LREI-------DEKFYDSNLPGDKLIDLVENI
N-EETLASITPGLLGEFPNTYSYTKSAAEDIVRKYSQG--LPVAVFRPSI----------
--------------AASSAVFVEPSGTFVY--G-YFKVLVVVGAGVGILHVVLCNPSVI
ADMVPGDFVVNGCIATAWKTARDYP-G-NHEDAPVDNL-
PITYNYVSSEQNPMTWKLFMK
YIE-VYGMRVPS--IQAIWMCLCL-ITPYEFIYNFYFLVLHWIPAYITDFFLVIIGKKPI
----------LKKAYAKIKNFSAVIAYFAQREWKFHNGNTLNLYKEMD-ETDKQMFNFDM
ESLIWEE--YMEKYVMG---VRIYLLKD-----PVDTIPQGRKKQFKLMFKLLYITSYFH

>Ban_EST2

MMEDKY-KESQIQKLFSGASVLL---------------TGGTGFLGKLLVEKLLRSCPD
IKKLY-----LLTRPKKNKEIIKRLQEQFDDP--LYDKLRKVL---PDFI-EKIGIIEGD
VGQIGLGIGEEDRTTITKEVDFIFHGAATVRFDEALKTAVEINVRGTREMLHLARSCTK-
LR---------AYVHISTAYSN--CP--LKEI-------DEKFYDALLAGDKLIDLVENM
D-VKTLDSITPGLLGEFPNTYAYTKSAAEDIVQKYSKG--LPVAVFRPSI----------
--------------VIGTA--RE---------------------------------

>Hmel-ctg22

---YT-----ILTRPKKNKEIMKRLQEQLDDV--LYDKLRKQM---PNFI-QKISIIQGD
IEQPYLGISEEDRNKIINEVEFIFHGAATVRFDEALKTAVAINVRGTREILLLARSCTKT
AH---------AHVHISTAYSN--CP--LTEI-------DEKFYDRNLSGDKLIDLVENL
D-EKTLDSITPGLLGEFPNTYAYTKSAAEDIVRKYSQG--LPVAVFRPSI----------
---------------VISTA--HEPVPGWID--NVYGPTGVVVG-GVGILRVLLCNPSLI
ADLVPGDFVVNGCIATAWKTARDYP----EEGAPVDNL-PITYNYVSSEQNPLTWERFTN
YSE-MNRRKMPL--MQATWMCIFW-LEPSKFLYNFYFVILHWIPAYIIDFFLVIIGKKPI
----------LKKTYVKIKKFSAVSTYFTQREWKFHNGNTLKLYKEMN-KTDKQLFNFDM
KSLVWED--YFYEYVKG---IRIYLMKD-----PLDTIPQSRKKQFKLMLIHYFLLSVIC
FGLFKLVW----FIAKLFISVNPLSFRLQCDFFNRYKGFDVV------------------

>Bmo_swdb10

-----------------------------------MVADIQKYSPKMKPVGEVTGFDAMD
-KLEAL-GESQIGKMFAGTTVLL----------------TGGTGFLGKLMVDKLLRCCPD
LKKLF-----LLARPKKNKDIAKRLQEQFDDV--LYDKLRKER---PNFI-EKICIVEGD
VGAINLGLSQEDRTKIANEVEFIFHGAATVRFDEALKTAVEINVRGTREMCILARSCGK-
LK---------ALVHISTAYSN--CT--VMEI-------EERFYESSLPGDKLIDLVETM
D-EKVINSITPGLLGDFPNTYAYTKSVAEDVVQKYSKG--LPVALFRPSI----------
---------------VIGTA--KEPVAGWID--NVYGPTGVVVGAAVGLLHALICKPNAV
ADLVPGDMVVNACIATAWKTAKDYP-G-
NHEDAPPPDLTPPVYNFVSSTQKPLTWSKFMK
YNE-IYGLEVPT--VQAIYYYVFM-LTSSRFAYTLYCFFLHWIPAYIIDGVAVLIGKKPM
----------LRKAYTKISKFSEVISYFATREWKFHSGNTEGLVRELC-PADRQTFDFDL
SRLDWSE--YFYNYVRG---VRVYLLKD-----PVETVPAGLRRHNILKYSHYIFCAVLT
FVLVRLLW----AAWSLLDL--

>Bmo_swdb14

--MDSALALEIEQVSRQKAL
EEEALR-GESAVRQFYAGTTALI----------------TGGTGFIGKQLIEKLLRSC-D
VKRIY-----ILARSKKGLSMEERLQLQFKDP--VYDNLRSQQ---PDFA-QKVIPVEGD
ISELGVGLNDEDRMKLEQEVNVIFHVAATTRFVEPLKKATFINVRGTREILELAKCCKN-
LR---------CFVHVSTAFFN--AT--KSKI---NHTIEEKFYESPVPPEVMIQLAETV
E-ECRLNAITADLIRDWPNTYCFTKAIAEDVVRTMGKD--LPVAIVRPPV----------
---------------VICTL--NEPTPGWVDMSSVIGPSGIILTILLGVMHVLLMS-DIN
IPLAPLDYVNNAVIAAASEANKRYN-N-NEKE-------IKVYNISCSGCKIETSCLSDL
MNN-PNTQQYAT--PKAVWYCYGF-
ETGSDVVFWLSTWLLHYIPAYVADFVFNVTSMKRP
EG-----IKSFVKMYEKIYKMSLLYGYFLRNQWNLVDDNTVAMLNQMS-KTDRILFNFDI
SKIDWPR--YMLIWCIG---LRKFIVKD-----GLKDSEYGMKKQRWLKIAHISFYTLFF
GVVFSLLYLACRIFMYLCNLNTIAAQTLKI------------------------------

>Bmo_swdb15

--MDIALALEIEQVSRQKAL

EEEALR-GESAVRQFYAGTTALI----------------TGGTGFIGKQLIEKLLRSC-D
VKRIY-----ILMRSKKGKSMPERLQSQFNEP--VYDNLRSQQ---PDFA-RKVIPVEGD
ISELGVGLNDEDRMKLAQEVNVIFHVAATTRFEEPLKKATFINVRGTREILELAKCCKD-
LR---------SFVHVSTAFSN--AT--KSRI---NHNLEEKIYESPVPPEVMINLAKTV
E-ECRLNAITADLIRDWPNTYCFTKAIAEDVVRTMGKD--LPVVIV--------------
---LLTLMLGVMHVVLTS-DIS
IPIVPVDYVNNAVIATASDTNVRYN-K-NEKE-------IKVYNISCSGSKIETSCMSDF
LKN-PNTLEYAT--PKAVWYCYGF-GTGSDVVFWLSTWLLHYIPAYVADFVFNVTSMKRP
EG-----IKSFVKLYEKIYKMSMLYGYFLRNQWNFVDDNTVAMLNQMS-KTDRILFNFDV
AKIDCSR--FMAIWCIG---LRKFILKD-----GLKNSEYAK------------------

>Bmo_swdb16

--MDSALALEIEQVSRQKAL
EEEALR-GESAVRQFYAGTTALI----------------TGGTGFIGKQLIEKLLRSC-D
VKRIY-----LLIRSKKGKSMQERLKSQLNDP--VYDNLRSQQ---PDFT-RKVIPVEGD
ISELGVGLNNEDRMKLAQEVNVIFHVAATTRFEEPLKKATFINVRGTREIMELAKCCKD-
LR---------SFVHVSTAFSN--AT--KSRI---NHDLEEKIYESPVPPEVMIQLAETV
E-ECRLNAITADLIRDWPNTYCFTKAIAEDVVRTMGKD--LPVAII--------------
---ILTILLGVMHVLLMS-DIN
IPLAPLDYVNNAVIAAASEANKRYN-N-NEKE-------IKVYNISCSGCKIETSCLSDL
MNN-PNTQQYVT--PKAVWYCYAF-STGSDVIFWLLTWLLHYIPAYVADFVFNVTSMKRP
EG-----IKSYC----------------------------IAFITGFE-RDDRIRHSVTK
IKPNASHSAYRVARVQHIHTLRLCGVCE--------------------------------

>Bmo_swdb17

--MDSALALEIEQVSRQKAL
EEEALR-GESAVRQFYAGTTALI----------------TGGTGFIGKQLIEKLLRSC-D
VKRIY-----ILIRSKKGLSMEERLQLQFKDP--VYDNLRSQQ---PDFA-RKVIPVEGD
ISELGVGLNDEDRMKLAQEANVIFHVAATTRFEEPLKKATFINVRGTREIMELAKCCKN-
LR---------SFVHVSTAFFN--AT--KSRI---NHTIEEKFYESPVPPEVMIQLAETV
E-ECRLNAITADLIRDWPNTYCFTKAIAEDVVRTMGKA--LPVAIVRPPVGKYSRALLSE
IAKVETYKYVVTGHMFACTL--NEPTPGWLDMSSVIGPSGILLTAGLGVTHVVLIG-DIT
IALAPVDYVNNTVLATAWETDKRFK-T-NEKE-------SKIYNISNSRCTLEKIA----

>Hmel-ctg13

-------------------MNPTAPLLDFSEDHEFVSANHLDDYYSQEDQNDAKEAEKQR
LTKGKA-AKTDIQKFYKDQCIFL----------------TGGTGFLGKVIIEKLLRACGE
IDTIY-----VLVRSKKGKDPMTRIHEMLDDF--IFHAAHKIN---PKGI-HKVVPIVGD

MELPGIGLSDEDRRLLVSRVTIIINSAATVKFDEKLSIATGINVKGTKEVIKLAKECKN-
LI---------AITHVSTAFSN--TH--VDFI-------EEKFYESPMSVEAL-EAVSQL
D-DNVIESILPALLGNRPNTYCLTKAVAEEAIRTYGEG--MPICIIRPSI----------
---------------VISTY--EEPVRGWTD--SVYGPTGLVVGIGTGVLRTMYFDVNKV
ADMVPVDLTVNAIIVSAYQTAMNFKEN-QTSD-------IPIYNFVSGAQNPLKWGEFIK
YNM-KYGMDNPT--IKAVWYYGLT-PTNNFYFFVFLDFFLHYLPALLMDVFSAVTGKRRV
----------MLKFYTKLRKLANIMFYFSTQHWKFSDENVKDMWSSIS-AQDRAVFPFSI
GEISWDY--MAETFLLGKNTIRFSIRNK--------------------------------

>OnuZ2
MNHSLEASPLIPTEVTRDKMERWVEAQNKGEKTEVDVYGKPSEKQIKELENIRSLSRELQ
DNLHELENSVRIADVENQAMNPTAKILDYSEDHEFVSANQLDNYYAEEDKVDAKEEEK
RR
LTKGKA-GKTDIQQFYKDQCVFL----------------TGGTGFLGKVLIEKLLRSCTD
VDTIY-----VLIRNKKGKDARVRLHDMLDEF--LFHRALDEN---PKGI-HKVVPIIGD
MELPGLGISDEDRRTITSKVSIIINAAATVKFDEKLSVSTGINVRGTKEVLRLAKECRN-
LR---------AITHVSTAFSN--TH--VPYI-------EEKFYDPPMSVEAL-EAVSEI
N-EDLIDAILPTLIGKRPNTYCFTKAVAEEAVRKYGEG--LPICIVRPSI----------
---------------VVSTY--EEPVRGWTD--SVYGPTGLVVGTGTGVLRTMYMDLNKV
ADMVPVDLTVNAILASAWHAAKNFKEN-QTSD-------IPIYNFVSGAQNPIKWGKFIE
LNR-KYGIDKPT--TKAVWYYGLN-PTNSYFMFLFYNFFLHYLPALMVDAYCAVTGRRRA
----------MLKLYSKVMKMANILFYFSTRDWRFSDRGVRAMWASLS-EADRAVFPFSM
GDMSWDR--LCETFLIG---LRVYLIKD-----DLSTLPEARKKWNRLFYLHQLLKVVTF
ALVLNLAYLVLRPILSLVFGL---------------------------------------

>Dpl-ctg4

--------------------------------------MEWLNGNDPKIPNFN-----DL
NEPRNL-CEERIADYFQDSIILV----------------TGGTGFVGKALLEKLLRSCPG
IDTIY-----ILMRPKRGLSVEQRYKELLKNQ--VFDHIRTRW---PDRL-SKLYPITGD
VSAPNLGVSAEQ-
QQLLNKCHTVFHSAATVRFTDPLHAAAALNVQGTASLLQLAEDMPF-
LK---------ALVHVSTAYSN--AP--RSHI-------EERVYAPPYDPESIVRCTKML
P-AETVEVIAESLQGEHPNPYTLTKALAESIVYSHTN---LPVCIVRPSI----------
---------------VTAAY--QEPFPGWID--NIYGVTGIIMEISRGTYRSGYCRERYV
VDLVPVDMVVNSCILAAWRQGS----K-KPGR-------CPVYNVTSGSINPLQWGHFTK
LCV-KWARENPT--K---
----------MMKLARRFKMAAATGEYFANHEWQFSVSELTALHDEASVASDAGAFSHWP
GHFNWES--YIGSYMLG---IRRFILKD-----SIDSLPQARNKLNRLYWVHRLFQVATG
YYLFKFLAGRLR--

>Ban_EST3

--------------------------------------MECLNGND--LLSPNFNEIGTE
HFNQAY-PEERIADYFHNSIILV----------------TGGTGFVGKALLEKLLRSCPG
INTIY-----ILMRPKRGLSVDQRYKELLKNQ--VFDRIRAKW---PERL-SKLYPITGD

```
VSASNLGVSADQ-QLLLKKCHTIFHSAATVKFTEPLHAATTLNVQGTATLLKLAEDMPL-
LK---------ALVHVSTAYSN--AP--RSYI-------EERVYPPPYDPESIVKCTKML
P-AETVNIIAESLQGDHPNPYTLTKSLAESIVYSHTE---LPVCIIRPSI----------
---------------VTAAF--QEPFPGWID--NVYGVTGIIMEISRGTYRSGYCRERYV
VDLVPVDMVVNSCILAAWRQGS----K-KPGH-------CPVYNVTSGSINPLQWGHFTK
LCV-KWARENPT--KYVMWYPNFA-FTESRFVNTFWEVSCT-------------------
----------FYRLSFMIYCSEHRVEKL--------------------------------
------------------------------------------------------------
------------------------------------------------------------
----------------
```

>Bmo_swdb20

```
------------------------------------------------------------
-------------------MDDITLLTTTDKMEAFQKQMEFLNGNDPNMVNRNQECLPNY
QLLSSC-PAEAVADFYDGSVILV----------------TGGTGFVGKALLEKLLRSCPG
IETVY-----ILMRPKRGLTVEQRYKELLKNQ--VFDHIRTRW---PERL-SKLHPITGD
VSVQNLGVSVDQ-RELLRRVTTVFHSAATVRFTEPLHVATTLNVQGTASLLKLAEDMPK-
LK---------ALVHVSTAYSN--AP--RSYI-------EERVYPPPHDPDNIVRCTRML
P-PETVEVIADSLQGDHPNPYTLTKALAESIVYSHTE---LPVCIVRPSI----------
---------------VTAAS--QEPYPGWID--NIYGVTGIIMEISRGTYRSGYCRERYV
VDLVPVDMVVNSCILAAWRQGV----K---------------------------------
--------------QYVMWYPNFS-FTESRLINTFWEITCHVLPAFLYDLLLRAQGRKAI
----------MMKIARRFKMAAATGEYFANNEWQFGVAELQALQGEASSTRDAGQFPFWP
AGFDWDT--YIGAYMSG---IRKFILKD-----TVDSLPYARTKLKRLFWIHKLLQAATG
YYVFRLLAGRLR------------------------------------------------
----------------
```

>Dpl_FAR6

```
------------------------------------------------------------
------------------------------------------------------------
-MGPPD-LFRLSARRHSEYGTFI----------------N-----LFKVLIERLLSTCPD
VGKLH-----LLLRPKKGVAPEKRLQQLKSSQ--VFDLIRQNN---PRQL-DKLCIIPGD
VSQPGLAIDAEHLKNL-QDVSIVFHSAATLKFDEALPNAVDQNVLSVTRLMDICDTIPN-
LQ---------ALVHVSTAYSN--SE--LTCV-------EEKVYTPPADLGRLLALVEAV
P-KPLLANITPEYIKPKPNTYTFTKAMAEEAVRCRTQR--YPVAIFRPTI----------
---------------VISSL--RHPFPGWIE--NLNGPSGVIAAAGKGLLHVFVRRPDAR
ADLLPVDIAIDTLLAVAWETAV----D-RLPT-------VRVYNC-STNSNPTTWRQFET
ALK-KHLLNYPL--DACLWYPCGS-GVQNRYAHKALEFLLQTIPLHIAEYIIRGLGIKMK
--------LSLITAEQKMRAMNEVLAFFALREWKFNTDNVDKLRARLS-PADAAIYNLDP
KSISWDE--QYYNFIRG---TRKYLLKE-----KDENLDEARKHMNRMYYLHKGVIFFIM
VLVFRLVLQNKNVRQVVYGTLRLLLSLSFAAYKNVIESI---------------------
----------------
```

>Hmel-ctg19

```
------------------------------------------------------------
------------------------------------------------------------
------------------------------------------------VLIERLLSTCPD
VGRLH-----LLMRHKKGEAPEKRLLVLKQSQ--VFDILRQSN---PSQL-DKLCIIPGD
VSQPGLGITGSYLKEL-QEVSIVFHSAATLKFDEALPTAVEQNVLSVMRLMDICDTLPN-
LQ---------ALVHVSTAYSN--PE--RNVI-------EEKVYSAPAPLPRLLALVDAV
```

P-PPLMASITSQYIKPKPNTYTFTKAMAEEAINTRPNK-TYPIAIFRPTI----------
---------------VVCSL--RHPYPGWIE--NLNGPSGVVAAAGKGLLHVFRRKPDAR
ADLLPVDIAIDTMLAVAWETAV----D-KIPE-------VRVYNC-STFENPTTWLAFEN
SLQ-KFLPVNPM--DGCMWYPSGF-GVETKYAYKLLEMFLQTIPLHVAEYIIRTLGFKMK
--------LSLITADKKIRAMSGVLAFFALREWHFKTDNVAKLRARLT-PSDSAIYNLDP
KSIE--

>Ban_EST7

---M
VARPRSPCGALLPQFYAGRDIFI----------------TGATGFMGKVLIERLLATXPD
IGRLH-----LLMRQKKGVTPEKRLQQLIQSQ--VFDVIRQTN---PSQL-DKLCMIPGD
VSQPELGIPLQCLRQL-QEVSIVFHSAATLKFDELLSTAVEQNVLSIIRVMDLCDTLPN-
IQ---------ALVHVSTAYSN--PE--LNVV-------EEKVYDAPASLPRLLALVEAV
P-TPLLANITPDI---

>OnuZ6

---M
VSCKDD-VTAQIPQFYAGRSIFI----------------TGATGFMGKVLVERLLTTCPD
VDQLF-----LLMRTKRDVGPEKRLQQLKESQ--VFDNVRQCS---PAQL-DKLCIVPGD
ITKPQLGMTGESIAQL-RNVSVVFHSAATLKFDEALGLAVDQNVRSVVRLMELCDMLPN-
MQ---------AFVYVSTGYSN--AE--LSVV-------EERVYPPPAPLEQVLALTEAM
P-EDLMASVTRKYIHPKPNTYTFTKAIAEVAVQQHNSS--YPRAIFRPTI----------
---------------VVSAL--RTPFPGWVE--NLNGPTGVIAGVGKGLLHFFSCRRDAR
ADMVPVDIVIDALLAVGWETAI----D-RSSE-------VRVYNC-SSYNNPTTWRTFEA
ALS-RYIREHPM--DQVFWYPYGS-IVENGVAQKCLELLLQTMPLHLADYCSRIFGIKTK
--------LSFIMVSNRLQAMNTVLQFFSQREWKFITNNMERLRQRLT-PADAAIFNLDV
KTINWEE--LCTNFIKG---TKKYIFKE-----KAENTPEAKKHLQRMKLLPPWCNFLHF
NPASTTHL--

>Bmo_swdb18

---------------------------------------MTIREGSETIYTRRLLNMVPR
PVSPYA-REPLIPQFYAGKSIFI----------------TGATGFMGKVLVERLLWTCPE
IERIF-----LLMREKKEVSPEKRLAQLKQSQ--VFDVIREYC---PGQL-EKLYLIPGD
TTAPNLGLSVESIGLL-KEVSVVFHSAATLKFDEALNKAVDQNVRSVLSLMDICDGMPN-
IE---------ALVHVSTAYSN--AE--LSTV-------EERVYAAPVPLQQLLALADAL
P-DQLAADLTPRLISPKPNTYTFTKALAENAVAERTNR-KYPVAIFRPTI----------
---------------VISAL--RHPFPGWIE--NLNGPSGVLVAAGKGLLHFFCCKRSAK

ADMIPVDIAIDTLIAAAWETAV----D-KLDE-------IRVYNC-SSGRNSTTWAQFEM
AIK-KLLIEHPL--DKTYWYPSGA-AVEN------------------------------E
--------LSLITISERLQGMCKVLTFFSMREWDFKSDNVRKLQARLS-PQDALIYNLDP
NTIDWDA--LYVDFVKG---TRKYLLKE-----KDQDIDEAKRHLRKMFYVHNGFLVFVT
ALLLRLSLHAPLVRSLVLGTLRLLFTIVSSTYSRVSS-----------------------

>Yev_FARIII

--MVAR
RLSGEF-PAPTIPAFYAGKSVFI----------------TGATGFMGKVLIERILATCPN
VGCLY-----LLMREKKDQSPEKRLLQLKQSQ--VFDVLRRTQ---PAQL-DKLRSLSGD
TSREQLGMDSNSLQQL-REVSIVFHVAATLKFDEELRKAVEENLRSIMRLLNICDSLPH-
IE---------ALVHVSTAYCM--AE--LAEV-------EERVYTPIVPLPTLEALVDHA
D-EAMRQQLVQLLLGDKPNTYTFTKAMAEHAVVEHKAQ--YPVAIFRPTV----------
---------------VISSL--RSPVPGWIE--YLNGPSGVVVGAGKGLLHTFRCTKSAR
ADLLPVDIAIGTMITCAWDVAT----N-RQAT-------PRVYNC-STYENPTTWSDFER
CIR-KEIIEQPL--DAPLWYPCAF-IAENKYVHKLYELVLQTGPLHLAEWSARAVGMKPK
--------INLITVSRKLRAMSDVLMFFSLREWRFRTENVQRLRDRLS-PQDAAIYNLDV
NTIDWRQ--HMKNFMMG---VRKYLLKE-----KDQDIEAAKKHLRKMYWVHTGVKLLLL
GLTLRLLLRGPAAQSLA-GALRLLLSLCTAVYSRLLPAA---------------------

>Bmo_swdb19

---------------------------------------MFVTDTSTLHEGCVLQERGGT
MADMSE-EAPILPRFYAGRSVFI----------------TGGTGFMGKYTRVICVVSKPS
LKTCNETARSVPPRPLPGPLCSGRSVGLCVQ---IFEKVRERC---PWQL-DKLRVLPGN
LERPLLGLEPVTIARL-HQVSVVFHNAATLKFDEALRKAVDQNVRGLLSLVDVCDALPN-
LE---------ALVHVSTAYSN--AE--LSRV-------EERVYAAPVPLQQLLALADAL
P-DQLAADLTPRYIHPKPNTYTFTKAMAECVLQERTSR-RYPVAIFRPTI----------
---------------VISAD--RHPVPGWIE--NLNGPSGVVVGVGKGLMHVFRAKLALR
ADFIPVDVVVDNMIAVAWETGS----E-PCGS-------LRVYNCASG-EHATRLSDFRA
TAV-RVLRSLPL--DSGLSYPFLI-VVQNRFVYRLLEFVLQTAPLHVAECLRQTDGQKAR
YHNVPCVQLKVLVVQQRNRGRLRLVHYTTIVFLVLLSYKLIKF-----------------
---------FLNLFMNN---

>Dpl-ctg10

-----M-EESQIKAFYSGKNLFI----------------TGGTGFVGLCLIEKILRTIPD
VGKIY-----LLMRPKKGKEIADRLQEFPANP--VFEHLLQNT--SKDIF-NKLIPISGD
VGVENLGINDNDRQILIDEVNIVIHSAATLDFEENLRPTVKINVLGTRYVMDLCQQIKN-
LK---------VMIHVSSAYVN--SY--LKEV-------DEKVYDRPADPENIINMVNTL
T-NDALNDVERLILKNHPNTYTFTKHLAEHEVKKCEAM--FPISIVRPTM----------
---------------IVAAL--KEPVPGWTC--SKVGPQGFLMGAAKGVVRRLPLAKENI
ADYIPVDIVVNQLLAAGWNAAR------QNSG-------LQVYHCSTSTQNPFRWSILES
VVN-KILTNYPL--KSAVWYPHLA-FVRSLWLFRLSAIFIHFFPAVLLDMLLRITGGKPI

----------CM--
--LQWEE--YFVKLHLG---VRRYLNKE-----TEKTLAAAKTKNNM-------------

>Dpl-ctg12

-----M-EESQIKAFYNGKNLFI----------------TGGTGFVGLCLIEKILRTIPD
VGKIY-----LLMRPKKGKEIADRLQEFPGNP--VFEHLLQNT--SKDIF-NKLVPISGD
VGVENLGINDNDRQILIDEVNIVIHSAATLDFEENLRPTVKINVLGTRYVMDLCQQIKN-
LK---------VMIHVSSAYVN--SY--LKEV-------DEKVYDRPADPENIINMVNTL
T-DDALNDVERLILKNHPNTYTFTKHLAEHEVKKCEAM--FPISIVRPTM----------
---------------IVAAL--KEPVPGWTC--SKVGPQGFLMGAAKGVVRRLPIAKENI
ADYIPVDIVVNQLLAAGWNAAR------QNSG-------LQVYHCSTSTQNPFRWSILES
VVN-KILTNYPL--KSAVWYPHLA-FVRSLWLFRLSAIFIHFFPAVLLDMLLRITGGKPI
----------LFRLHKNVWNSLSRLEVFIFTEWKFNNPRTRELSAMMN-KTDSELFDIDV
SKIYWEE--YFVKLHLG---VRRYLNKE-----TEKTLAAAKTKNNILYYVHIIWQVMIL
GLLWVIISWVTGLGLTQTSWVIPPIYLLYSML----------------------------

>Hmel-ctg21

----MS-EESQVRSFYSGKNFFI----------------TGGTGFVGLCLIEKILRTIPD
VGKLY-----LLIRPKKGKDITSRIQEFAKHP--VFEKLLETN--TNDIF-NKLIPISGD
VGTKDLGISEEDRQILIENVNVVVHSAATLDFEESLKPTVQINVLGTRQIMELCDQMKN-
LK---------VMIHVSSAYVN--SY--LKEA-------HEKVYDTPADVESVIDLSRKL
S-EQALVDMEKTILKEHPNTYTFTKHLAEHEVKKYSEK--FPCTIVRPTM----------
---------------IVAAW--KEPVVGWTC--SKVGPQGFLMGAARGVVRRLPLSKDNV
ADYIPVDVVVNQLLAAGWNAGV------SGPG-------LTVYHCSSSTQKPFKWSMLEN
VVN-KMLQNYPL--KSAVWYPYLD-FVSSLWLFRISAIFIHFLPAILLDMLLRITGKKPI
----------LFRLHKNVWNSLSRLETFIFSEWKFHNTNTRELNQKLN-KVDKEIYFIDI
QTIVWEQ--YFVNLHLG---VRKYLNHE-----SEKTLPAAKNKHSM-------------

>Bmo_swdb11

----MT-EESLVRAFYSGKNFFI----------------TGGTGFVGLCLIEKILRCIPD
VGKVY-----LLMRPKKGKEISQRLEEFPKNL--VFEKLLETN--TTDIF-KKLVPISGD
VGEANLGLSPQDRQLLIENVNVVIHSAATLDFQENLRPTVRINLLGTRQVMGLCKEIKN-
LK---------VMIHVSSAYVN--SY--LTEV-------HEKVYPEPEEVEKVISLVGTL
S-DEALDEIEPKILKDHPNTYTFTKHLAEHEVAKCAEL--FPCTIVRPTM----------
---------------IVASW--KEPVPGWTC--SKVGPQGFLMGASKGVVRRLPLAKENI
ADYIPVDVVVNQLLVAGWHAAS------ARSG-------LTVYHCSSSTQKPFKWSMIES
SIN-GMLHKYPL--KSAVWYPHLK-FVPSLWLFRLSAIFVHFFPALLLDMMLRVTGGRPI
----------LIRLHKNVWNSLNRLEKFIFSEWKFHNTNTQQLAKELN-KTDAQTFFIDI
SKLYWEE--YFMNLHLG---VRRYLNNE-----KEGNLKAARNKDTM-------------

>Hmel-ctg8

-----------------------------------MSHVPSGNSLNNCLYALLYERPLFS
FSENKL-KYRSIKESYSGKSIFL----------------TGGTGFLGKVLIEKLLRST-D
VEKIF-----VLIREKKGIDSDKRLKQMLNIP--IFEDLKENK---PNAL-NKLVAIGGD
VTKKDLGISPEDEQKIIDNVSLVFHSAATVKFTDPFSNIMNINYEGTKKVLKLSKKIKN-
LE---------TFVYISTAFCQ--GD--RTHI-------NESMYPRFYEEGEVYKFIDAY
G-DNPTEVV--KFLDGKPNTYTLSKSLCESYIKEEHKP--LSTIVLRPSI----------
---------------VTPVY--KGPVPGWVD--SWVAATALFSDVARGLTKVMYGDKNVI
CDMIPVDYVINLAIVAGARG------N-KTDD-------ILVFNSSSGTANPITWKEGAD
LYL-EESLKYGK--SELK--PRKVMVLSSPLLMDFLSFTLQTTPAFLADLMLRIKGEQPK
----------YLNMQKRAILLRDILRTFSSISFQIKSQKTEGLYTTLS-EEDKVQFPFDP
KTIVWRK--YMRIFYKG---VQKYMLND-----K--------------------------

>Bmo_swdb2

--GLIEKLLYSCVG
IENIY-----VLIREKEEESADQRLKKIIDS---VFDRVKKQD---INVL-NKIVTVPGN
ISEKNLGLTTEHLKIIQEKVSVVFHVAATVKFNEELSKAMAVNVNGTLEIISLCRRINK-
LESDEHTAYLMALIQVSTAFSN--SD--RKHI-------EEIVYCNNVDFEGLKSLTDFW
S---GDEDDVKRLLGKKPNTYTYTKALAEEYVMKYGKD--LPIAIIRPSI----------
---------------IISAI--KEPSQGWID--SWNGSTGTFGGMSSGVMKVMFGSGSNV
ADLVPLDIVVNLIIVAATKC--------KRSS-------LKVYNCCTGTSNPITYSRAEV
IIR-RATYKYSL---NQLPFPFMV-FTNSRLVYTLLRFILQIVPAFLIDLWSLFNGKKAI
----------QMKIQHRITRTLDLMKFFMINEWHFEDSNTRELFENMS-EEDKNVFNFDL
KSIDWDS--CIENYVVG---RRAYGPPDGEWLPSPMDFSNARGRAKPLPTAQAAAYLLIY
RLGYISFVLTCR--

>Ola_pgFAR

---MAANTIE
TDEQFT-YNSPIVNFYSGKSVFV----------------TGATGFLGTVLVEKLLFSCKG
INNIY-----VLIKQTKDLTIEGRVLNYLNSK--AFHRVRNTN---PELM-KKIIPICGN
LEHKNLGISDSDIKTLQEEVSIVFHVAAKLLFKMSLATAVNINTKPTEQLIVICKKMRR-
NP---------IFIYVSSAYSN--VN--KQII-------DEKVYSNGVPLETIYDTLEAE
N-----TRLMDMFLDKRPNTYIYSKALAEEVVGKEFDE--SAAAIVRPSI----------
---------------ILSSI--REPIPGWLT--GSHGFPQLVGAACRGFLLRWHGDGTVV
FDVIPVDHVANLIIAAAWETNERRL-V-GNKG-------VKVYNCCSSLRNPIDVITVMN
TCL-KYRKYFGTERTMSIFTPRYI-MKKNYFIYKLLYFTYHTIPAAIIDAFFWITGRTPM
----------MLKTLNKLGKLSSVLEYFTHHQFLFLDSNVRGLLRRMD-DTDRQTFNFDV
TEIEWEM--YLQNFVRG---IANNYDSC--------------------------------

>Opa_pgFAR

---MSANTIE
SDDQLINYNSPIVNFYSGKSVFV----------------TGATGFLGTVLVEKLLFSCKG
INNIY-----VLIKQSKDLTIEGRILNYLNSK--AFHRVRNAN---PEFM-KKIIPICGN
LEENNLGISDSDMKTLQAEVSIVFHVAAKLLFKMSLATAVNINTKPTEQLIAICKKMRR-
NP---------IFIYVSSAYSN--VN--KQII-------DEKLYTTGVPLKTIYDTLETE
N-----TRLIDIFLDKRPNTYIYSKALAEEVVGKEFDE--SAAAIVRPSI----------
---------------ILSSI--REPIPGWLT--GSHGFPQLVGAACRGLLLRWHGDGTVV
FDIIPVDHVANLIIAAAWETNERKL-V-GNKG-------VKVYNCCSSLRNPIDVITVMN
TCL-KYRKYFGTERTMSIFTPRYI-MKKNYFIYKLLYFTYHTIPAAIIDAFFWLTGRTPM
----------MLKTLNKLSNLSSVLKYFTHHQFLFLDSNVRGLLGRME-DIDRKTFNFDV
TEIEWEM--YLQNFVRG---IASNYDSC--------------------------------

>Ofu_pgFAR

---MSANTME
TDEQFT-YNSPIVNFYSGKSVFV----------------TGATGFLGTVLVEKLLFSCKG
INNIY-----ILIKRTEDLTIEARILNYLNSK--AFHRVKNTN---PELM-KKIIPICGN
LEDKNLGISDSDMKTLLEEVSIVFHVAAKLIFKMSLTAAVNINTKPTEQLIAICKKMRR-
NP---------IFIYVSSAYSN--VN--EQII-------DEKVYSTGVPLETIYDTLDAK
N-----TRLMDIFLDKRPNTYTYSKALAEVVVEKEFDE---SAAIVRPSI----------
---------------IASSI--REPIPGWLS--GSHGFPRVVEAACKGLLLRWHGDGTVA
FDVIPVDHVANLIIAAAWESNERRL-M-GNKG-------VKVYNCCSGLRNPIDVSTVMN
TCL-KYRKYFGT-RTMSIITPRFI-MKKNYFLYKLLYFTYHTIPAAIIDGFFWLTGQTPM
----------MLNTLHKLRKLSSVLEYFTLHQFLFLDSNVRGLLRRME-STDRQTFNFDV
TEIEWEP--YLQNFVRG---IANNYD----------------------------------

>OscZ_pgFAR

---MSANTME
TDEQFT-DNSPIVNFYSGKSVFV----------------TGATGFLGTVLVEKLLFSCKG
INNIY-----ILIKQTEDLTIEARILNYLNSK--AFHRVKNTN---PELM-KKIIPICGN
LEDKNLGISDSDMKTLLEEVSIVFHLAAKLLFKMSLAAAVNINTKSTEQLIAICKKMRR-
NP---------IFIYVSSAYSN--VN--KQII-------DEKVYSTGVPLETIYDTLDAK
N-----TRLVDIFLDKRPNTYTYSKALAEVLVEKEFDE---SAAIVRPSI----------
---------------IASSI--REPIPGWLS--GSHGFPRVVEAACKGLLLRWHGNGTVA
FDIIPVDHVANLIIAAAWESNERRL-I-GNKG-------VKVYNCCSGLRNPIDVSTVMN
TCL-KYRKYFGT-RTMSIITPRFI-MKKNYFLYKLLYFTYHTIPAAIIDGFFWLTGRTPM
----------MLNTLHKLRKLSSVLEYFTLRQFLFLDSNVRGLLRRME-GTDRQTFNFDV
TEIEWEP--FLQNCVRG---IANNYD----------------------------------

>OnuZ_pgFAR

--MSANTME
TDEQFT-YNSPIVNFYSGKSVFV----------------TGATGFLGTVLVEKLLFSCKG
INNIY-----ILIKQTEDLTIEARILNYLNSK--AFHRVKNTN---PELM-KKIIPICGN
LEDKNLGISDSDMKTLLEEVSIVFHLAAKLLFKMSLAAAVNINTKSTEQLIAICKKMRR-
NP---------IFIYVSSAYSN--VN--KQII-------DEKVYSTGVPLETIYDTLDAK
N-----TRLMDIFLDKRPNTYTYSKALAEVLVENEFDE---SAAIVRPSI----------
---------------IASSI--REPIPGWLS--GSHGFPRVVEAACKGLLLRWHGDGTVA
FGIIPVDHVANLIIAAAWESNERRL-I-GNKG-------VKVYNCCSGLRNPIDVSTVMN
TCL-KYRKYFGT-RTMSIITPRFI-MKKNYFLYKLLYFTYHTIPAAIIDGFFWLTGRTPM
----------MLNTLHKLRKLSSVLEYFTLRQFLFLDSNVRGLLRRME-GTDRQTFNFDV
TEIEWEP--FLQNCVRG---IANNYD----------------------------------

>Oze_pgFAR

--MAANTIE
TDEQFT-DNSPIVNFYSGKSVFV----------------TGATGFVGTVLVEKLLFSCKG
INNIY-----ILIKQTEDLTIEARILNYLNSK--PFHRVKNTN---PELL-KKIIPICGN
LEDKNLGISDSDMKTLLEEVSIVFHVAAKLLFKMNLTAAVNINTKPTEQLIAICKKMRR-
NP---------IFIYVSSAYSN--VN--EQII-------DEKVYSTRVPLETVYDTLDIE
N-----TRITDIFLDKRPNTYTYSKALAEVVVEKEFNE---SAAIVRPSI----------
---------------ILSSI--REPIPGWLS--GSHGFPRVVGAACKGLLLRWHGDGTVV
CDVIPVDHVANLSIAVAWESNERRL-M-GNKG-------VKVYNCCSSLRNPIDVSTVLN
TCL-KYRKYFGT-RTMSIFTPRFI-MKKNYFLYKLLYFTYHTIPAAIIDGFFWLTGRTPI
----------MLKTLDKLRKLSSVLEYFTHHQFLFLDSNVRGLLRRME-GTDRQTFNFDV
TEIEWEP--YLQNFVRG---IANNYDNS---M-----------------------------
----

>OscE_pgFAR

--MSANTME
TDEQFT-YNSPIVNFYSGKSVFV----------------TGATGFLGTVLVEKLLFSCKG
INNIY-----ILIKQTEDLTIEARILNYLNSK--AFHRVKNTN---PELM-KKIIPICGN
LEDKNLGISDSDMKTLLEEVSIVFHVAAKLLFKMSLTAAVNINTKPTEQLIAICKKMRR-
NP---------IFIYVSSAYSN--VN--EQII-------DEKVYNTGVPLETIYDTLDTE
N-----TRITDIFLDKRPNTYTYSKALAEVVVEKEFDE---SAAIVRPSI----------
---------------IVSSI--REPIPGWLS--GSHGFPRVVGAACKGLLLRWHGDGTVV
CDLIPVDHVANLIIAAAWESNERRL-M-GNKG-------VKVYNCCSSLRNPIDVITVVK
TCI-KYRKYFGT-RTMSIFTPRFI-MKKNYFIYKLLYFTCHTIPAAIIDGFFWLTGRTPI
----------MLKTLDKLSKISSVLEYFTHHQFIFLDSNVRGLLRRME-GTDRQTFNFDV
TEIEWEP--YLQNFVRG---IANNYDYS---M-----------------------------
----

>OnuE_pgFAR

--MSANTME
TDEQFT-YNSPIVNFYSGKSVFV----------------TGATGFLGTVLVEKLLFSCKG

INNIY-----ILIKQTEDLTIEARILNYLNSK--AFHRVKNTN---PELM-KKIIPICGN
LEDKNLGISDSDMKTLLEEVSIVFHVAAKLLFKMSLTAAVNINTKPTEQLIAICKKMRR-
NP---------IFIYVSSAYSN--VN--EQII-------DEKVYNTGVPLETIYDTLDTE
N-----TRITDIFLDKRPNTYTYSKALAEVVVEKEFDE---SAAIVRPSI----------
---------------IVSSI--REPIPGWLS--GSHGFPRVVGAACKGLLLRWHGDGTVV
CDLIPVDHVANLIIAAAWESNERRL-M-GNKG-------VKVYNCCSSLRNPIDVITVVK
TCI-KYRKYFGT-RTMSIFTPRFI-MKKNYFIYKLLYFTYHTIPAAIIDGFFWLTGRTPI
----------MLKTLDKLGKISSVLEYFTHHQFIFLDSNVRGLLRRME-GTDRQTFNFDV
TEIEWEP--YLQNFVRG---IANNYDYS---M----------------------------

>Oza_pgFAR

---MSANTVE
TDEQFTDNNSPIVNFYSGKSVFV----------------TGATGFLGTVLVEKLLFSCKG
INNIY-----ILIKQTEDLTIEARILNYLNSK--AFHRVKNTN---PELM-KKIIPICGN
LEDKNLGISDSDMKTLLEEVSIVFHLAAKLLFKMSLTAAVNINTKPTEQLIAICKKMRR-
NP---------IFIYVSSAYSN--VN--KQII-------DEKVYSTGVPLETIYDTLNTE
N-----TRITDILLDKRPNTYTYSKALAEVVVEKEFDE---SAAIVRPSI----------
---------------IVSSI--REPIPGWMS--GSHGFPRVVGAACEGLLLRWHGDSTVV
FDVIPVDHVANLIIAAAWESNERRL-M-GNKG-------VKVYNCCSGLRNPIDVSTIMN
TCL-KYRKYFGT-RTLSIFTPRFI-VKKNYFIYKLLYFTYHTIPAAIVDGFFWLTRRTPM
----------MLNTLHKLSKLSSVLEYFTLHQFLFLDSNVRGLLRRME-STDRQTFNFDV
TEIEWEP--YLQNCVRG---IANNCDYS---M----------------------------

>Onr.za_pgFAR

---MSANTVE
TDEQFTDNNSPIVNFYSGKSVFV----------------TGATGFLGTVLVEKLLFSCKG
INNIY-----ILIKQTEDLTIETRILNYLNSK--AFHRVKNTN---PELM-KKIIPICGN
LEDKNLGISDSDMKTLLEEVSIVFHLAAKLLFKMSLTAAVNINTKPTEQLIAICKKMRR-
NP---------IFIYVSSAYSN--VN--KQII-------DEKVYSTGVPLETIYDTLDTE
N-----TRITDILLDKRPNTYTYSKALAEVVVEKEFDE---SAAIVRPSI----------
---------------IVSSI--REPIPGWMS--GSHGFPRVVGAACKGLLLRWHGDSTVV
FDVIPVDHVANLIIAAAWESNERRL-M-GNKG-------VKVYNCCSGLRNPIDVSTIMN
TCL-KYRKYFGT-RTLSIFPPRFI-VKKNYFIYKLLYFTYHTIPAAIVDGFFWLTRRTPM
----------MLNTLHKLSKLSSVLEYFTLHQFLFLDSNVRGLLRRME-STDRQTFNFDV
TEIEWEP--YLQNCVRG---IANNCDYS---M----------------------------

>Dpl-ctg8

-----------------------------MVKCNRRLLFNRNKSFYNAYSNMRSPSHCVT
EDDLNK-KTSSIVDFYNGKSIFI----------------TGGTGFLGKMYIRNILHRCPG
ICKIY-----MLIRPTSKNSVESRLEDMIKNSMNLFEGLN------ENHF-MKLVPIEGD
VTAPNLGIHPDKKRLLQEQVSIIVHSAATLDINATLREAYHVNVIGTENLLAVAKDMKK-

LH---------AFLHLSTFYCCMTNE--KNAI-------EEKVYSPPLYVKDVPKTLEKY
G---HMRFPPQSLLKNKQNTYIFTKAISEYLVSSTHGD--IPTAIIRPSL----------
---------------IGCSN--KDPYKGWID--VFNIHSFILTAISNGFLRILNINKNTV
WDFIPVDYVANLMILAATKC------K-KSTQ-------VDIYNCASSGINPVTILSACS
IFISDFVT-----------------NKKSSKKIRLLNPFVHLIPAFINDVRYILTGRKAN
--------------IKKLLKIKDQIKFFGGHSWKAKSDHVTSLINSLS-DEDRKKFPCDP
SDIDWVE--YFREYSKG---IRNYLSKN--------------------------------

