# Supplementary material for: Mosaic Evolution of Molecular Pathways for Sex Pheromone Communication in a Butterfly
Source: Genes (Basel). 2022 Jul 31;13(8):1372. doi: 10.3390/genes13081372 (PMC9407440; doi:10.3390/genes13081372)
Supplement: Supplementary file 1 [file genes-13-01372-s001.zip › Supplementary File S5.pdf]

Supplementary File S5: Functional expression of *B. anynana* odorant receptor candidate genes in transgenic flies

We set out to functionally investigate if some of our candidate OR genes were specifically responsible for the detection of male pheromone components by using heterologous expression in *D. melanogaster* olfactory sensory neurons (OSNs) coupled to electrophysiological recordings. We focused on the 5 candidate *B. anynana* ORs with the highest expression level in antennae, for which we obtained the full-length sequence by RACE-PCR, including Bany_OR1 and Bany_OR2 previously cited (Supplementary File S5 Table 1 and 2). Each of the five candidate OR genes was expressed in *D. melanogaster* pheromone-sensitive neurons (at1 OSNs) in place of the endogenous fly OR, using the mutant knock-in allele *Or67d*^{GAL4} [95]. Even though *B. anynana* OR transgenes were indeed expressed and yielded a normal spontaneous electrophysiological activity, the three MSPs used as odorant stimuli did not induce any electrophysiological response from the transformed OSNs, in any of the five fly lines generated (Supplementary File Figure S1A). A similar result was obtained when *B. anynana* ORs were overexpressed in the vast majority of fly OSNs, using the Orco-GAL4 driver line (Supplementary File Figure S1B). Thus, none of the five candidate ORs tested seem to be involved in pheromone detection, although this lack of response in a heterologous expression system may also result from a much lower sensitivity of *B. anynana* pheromone receptors compared to moth pheromone receptors. In view of the large amount of pheromone emitted by *B. anynama* (several µg) compared to moths, it is possible that the responding PRs are in fact expressed at low level in antennae. Abundance in antennae may thus not be a good criterium to search for PRs in butterfly, although it is in moths. If PR abundance is low, it is possible that we missed candidates in the classical moth PR clade. It has to be noticed that the genome analyses of both *Danaus* and *Heliconius* identified ORs in this clade (although none have been yet confirmed to be functional PRs).

We used the *D. melanogaster* heterologous expression system to express the entire sequence length of candidate *B. anynana* odorant receptor genes (i.e. “transgene”) within the odorant receptor neurons (ORN) of *D. melanogaster* flies. To the best of our knowledge, this is the first attempt of heterologous expression of butterfly genes in the *D. melanogaster* model system. Unfortunately, none of the transgenic Orco-Gal4 fly strains in which the *B. anynana* candidate OR were inserted were able to sense any of the three *B. anynana* MSPs components (Supplementary Figure 6). It may be that the Orco-Gal4 *D. melanogaster* driver was not sufficiently sensitive to functionally validate the activity of butterfly ORs, which are likely much less sensitive than moth ORs, on which this experiment was technically developed originally (e.g., [34]).

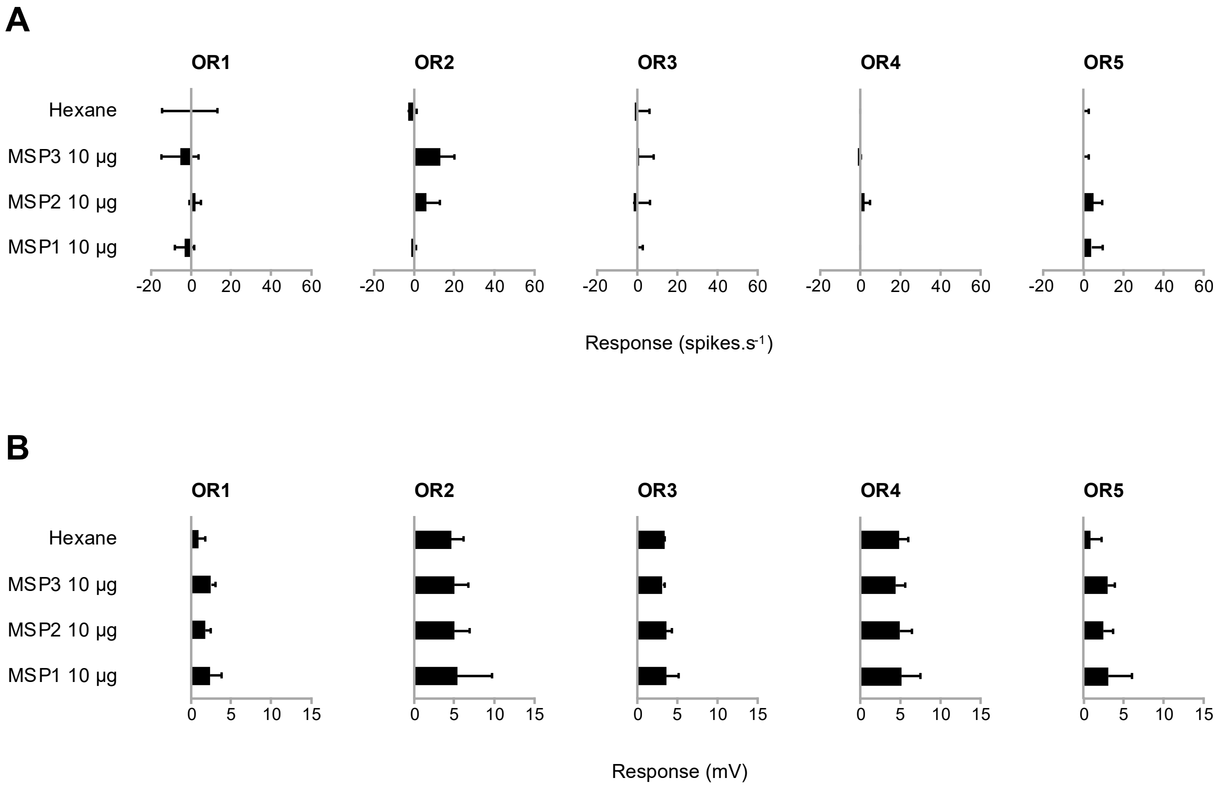

Supplementary File Figure S1. Response spectra of *B. anynana* candidate ORs expressed in *Drosophila* (A) Action potential frequency of at1 OSNs expressing candidate ORs (*w*;*UAS-BanOR*;*Or67d*^{GAL4}) during a 500 ms stimulation, measured using single-sensillum recordings (*n* = 2-3). (**B**) Depolarization amplitude of antennal OSNs expressing candidate ORs (*w*;*UAS-BanOR*/+;*Orco-GAL4*/+) during a 500 ms stimulation, measured using electro-antennogram recordings (*n* = 2-3).

Materials and methods:

We expressed the full-length coding sequence (cds) of 5 candidate *B. anynana* OR genes (BanORs) in olfactory sensory neurons (OSNs) of *D. melanogaster* flies. After *in silico* assembly of both 3' and 5' RACE fragments, the cds was confirmed by RT-PCR using antennal mRNA as a template. The cDNA fragments encoding the 5 BanORs were subcloned into the expression vector pUAST.attB using the Gateway system and plasmids quality was checked by forward and reverse sequencing. Transformant UAS-BanOR fly lines were generated by BestGene Inc. (Chino Hills, California, USA) using phiC31 transgenesis, by injecting the pUAST.attB-BanOR plasmids into fly embryos with the genotype *y1 M{vas-int.Dm}ZH-2A w*; M{3xP3-RFP.attP}ZH-51C* [96]. This resulted in the insertion of transgenes in the region 51C of the chromosome 2. For EAG experiments, UAS-BanOR lines were crossed to an Orco-GAL4 line (#23292 from the Bloomington Drosophila Stock Center). For SSR experiments, UAS-BanOR lines were crossed to a line harboring the *Or67d*^{GAL4} knock-in allele [95] to generate double homozygous flies with the genotype *w; UAS-BanOR; Or67d*^{GAL4}. The presence of transgenes was confirmed by PCR on

genomic DNA extracted from 2 flies, and correct expression of the BanOr transgenes was confirmed by RT-PCR on total RNA extracted from 100 pairs of antennae for each *Drosophila* transformant line. Fly lines were reared on standard cornmeal-yeast-agar medium and kept in a climate and light-controlled environment (25°C, 12h light:12h dark cycle).

Electroantennogram (EAG) recordings were performed on UAS-BanOR and Orco-GAL4 parental lines (controls, data not shown) and on Orco-GAL4/UAS-BanOR lines. Five- to 7-day-old flies were mounted in 200-µL pipette tips and EAGs were recorded using glass electrodes, as previously described [97]. Single-sensillum extracellular recordings of transformant *Drosophila* at1 OSNs were performed using tungsten electrodes as previously described [34]. Stimulus cartridges consisted of Pasteur pipettes containing a filter paper of 1 cm^2 loaded with 100 µg of pheromone diluted in hexane (>98% purity, Carlo-Erba). For EAG experiments, the positive control stimulus consisted of 10 µL of 2-heptanone (>98% purity, Aldrich) diluted in paraffin oil (10^{-3} dilution). All stimuli lasted for 500 ms.

95. Kurtovic, A.; Widmer, A.; Dickson, B.J. A Single Class of Olfactory Neurons Mediates Behavioural Responses to a *Drosophila* Sex Pheromone. *Nature* **2007**, *446*, 542–546, doi:10.1038/nature05672.
96. Bischof, J.; Maeda, R.K.; Hediger, M.; Karch, F.; Basler, K. An Optimized Transgenesis System for *Drosophila* Using Germ-Line-Specific ΦC31 Integrases. *Proc. Natl. Acad. Sci. U. S. A.* **2007**, *104*, 3312–3317, doi:10.1073/pnas.0611511104.
97. Montagné, N.; Chertemps, T.; Brigaud, I.; François, A.; François, M.C.; de Fouchier, A.; Lucas, P.; Larsson, M.C.; Jacquin-Joly, E. Functional Characterization of a Sex Pheromone Receptor in the Pest Moth *Spodoptera Littoralis* by Heterologous Expression in *Drosophila*. *Eur. J. Neurosci.* **2012**, *36*, 2588–2596, doi:10.1111/j.1460-9568.2012.08183.x.
