# Supplementary material for: Mosaic Evolution of Molecular Pathways for Sex Pheromone Communication in a Butterfly
Source: Genes (Basel). 2022 Jul 31;13(8):1372. doi: 10.3390/genes13081372 (PMC9407440; doi:10.3390/genes13081372)
Supplement: Supplementary file 1 [file genes-13-01372-s001.zip › Supplementary File S6.pdf]

Supplementary file S6: Manipulation of male sex pheromone production with Ban_PBAN synthetic peptide

We aimed to demonstrate the functional role of PBAN expression in regulating male sex pheromone synthesis in *B. anynana*. Previously, sex pheromone biosynthesis was successfully modified by application of the 3'-end of the PBAN peptide diluted in DMSO in some moth species (e.g., [98]). If PBAN regulates sex pheromone biosynthesis in male butterflies, as it does in female and male moths, we expected to see an increase in MSP amounts in *B. anynana* males receiving increasing amounts of the Ban_PBAN peptide, as a first evidence that the pathway regulating moth sex pheromone biosynthesis is indeed conserved in butterflies. For this, a synthetic Ban_PBAN peptide was applied either by topic application on the wings, or injected in the abdomen of live adult males. Two experiments were performed in an attempt to increase MSP production by topical application of synthetic PBAN, one experiment in which Ban_PBAN peptide was diluted in acetone, and another was using DMSO (dimethylsulfoxide) as a solvent. Each solution was applied topically to male wings. Overall, Ban_PBAN peptide concentration did not affect the amount of any of the MSP components, irrespective of the solvent (Supplementary File Figure S1A for DMSO, S1C for acetone; Supplementary File Table S1). MSP1 and MSP3 amounts were, however, significantly lower two hours after topical application of Ban_PBAN peptide in DMSO, compared to only one hour after application (Supplementary File Figure S1B; Supplementary File Table S1). In another experiment, we aimed to manipulate the level of Ban_PBAN using injections. The amount of none of the MSP components changed with increasing Ban_PBAN concentration through injections (MSP1: $t = -0.162$; $p = 0.872$; MSP2: $t = -1.079$; $p = 0.285$; MSP3: $t = 0.824$; $p = 0.413$; Figure S1D).

Although we did not convincingly demonstrate that sex pheromone biosynthesis is regulated by the neuropeptide PBAN in *B. anynana*, we at least demonstrated that PBAN expression, pheromone production and mating behavior were correlated. Further functional validation of PBAN regulatory activity on sex pheromone biosynthesis in this and other butterfly species is still needed but we provide indirect evidences that both butterflies and moths may use a similar neuroendocrine control for pheromone production regulation. There is a notable difference, however, in PBAN regulation between moths and butterflies: upregulation of sex pheromone production takes place during the photophase in butterflies (light) and not the scotophase (dark).

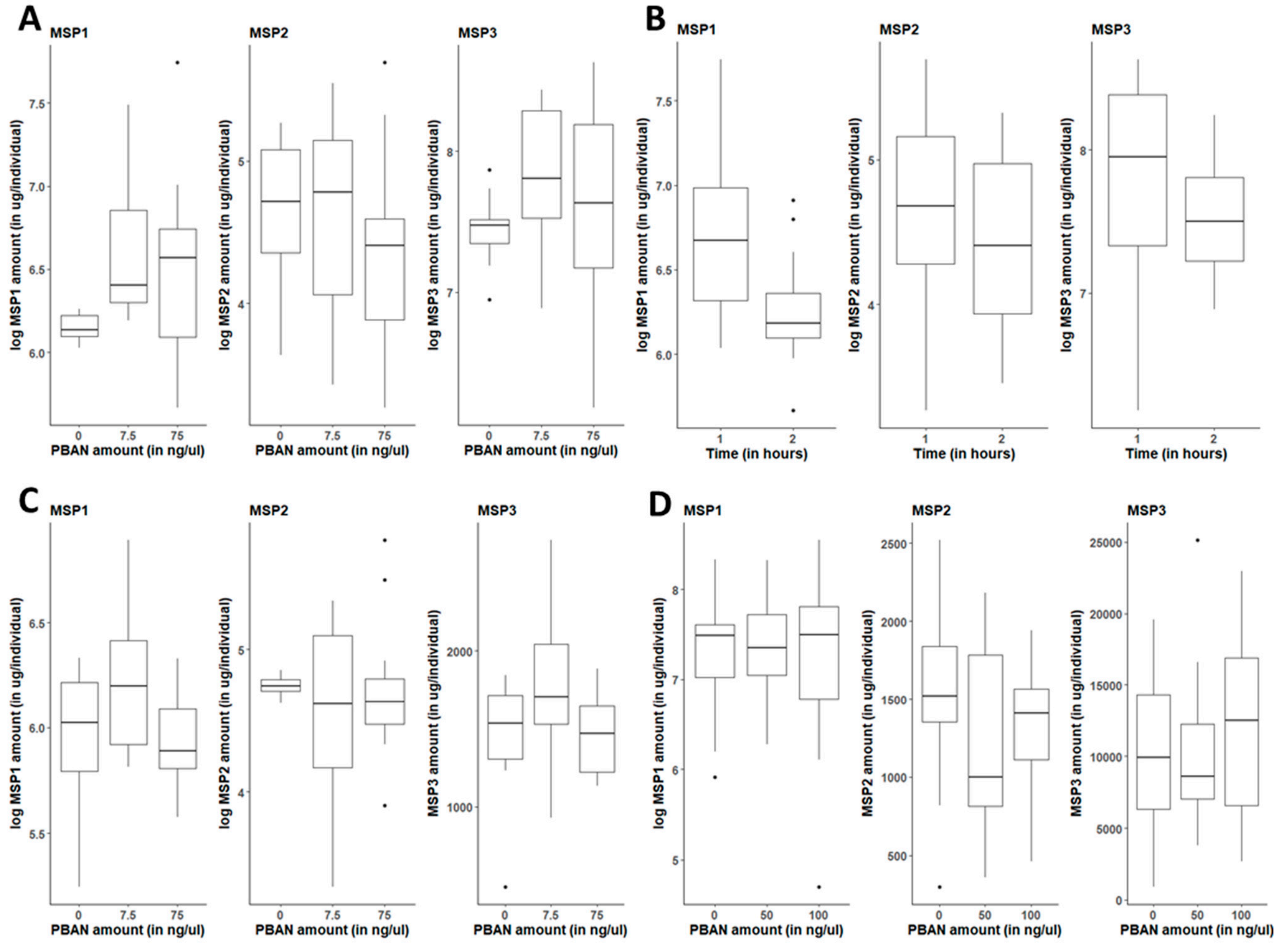

Supplementary File Figure S1: Effect of experimental addition of Ban_PBAN peptide on MSP amounts in adult *B. anynana* males. (A) Amount of MSP1, 2, and 3 components (y-axis) in response to topically applied PBAN in DMSO at different concentrations (x-axis), N = 32. (B) Amount of MSP1, 2, and 3 (y-axis) in response to topically applied PBAN in DMSO one and two hours after application (x-axis), N = 32. (C) Amount of MSP1, 2, and 3 components (y-axis) in response to topically applied PBAN in acetone at different concentrations (x-axis), N = 32. (D) Amount of MSP1, 2, and 3 components (y-axis) in response to Ban_PBAN peptide injections at different concentrations (x-axis), N = 57.

Supplementary File Table S1: Statistics on the effect of topically applied PBAN in DMSO (A) and acetone (B).

**A. DMSO**	MSP1		MSP2		MSP3	
	t-value	*p*-value	*t*-value	*p*-value	*t*-value	*p*-value
PBAN amount	0.454	0.622	-1,899	0.0593	-0.499	0.618
Time after application	**-3,813**	**<0.001**	-1,739	0.0831	**-2,018**	**<0.046**

**B. Acetone**	MSP1		MSP2		MSP3	
	t-value	*p*-value	*t*-value	*p*-value	*t*-value	*p*-value
PBAN amount	-1,346	0.164	0.229	0.797	-0.986	0.334
Time after application	-0.29	0.761	-0.206	0.85	-0.538	0.613

Materials and methods:

Two separate experiments were performed in an attempt to increase MSP production through topical application of synthetic PBAN peptide (Genscript USA; "Ban_PBAN" hereafter). In the first experiment, Ban_PBAN was diluted to a concentration of 0 (control), 7.5, and 75 ng/µl in acetone or DMSO (dimethylsulfoxide). Male butterflies were incapacitated using CO_2, after which the solution was applied topically to males aged 3, 8, and 11 days old (n = 32 for both solvents). Males were sampled 1 or 2 hours after topic application and amounts of the three MSP components were quantified with GC-FID as described below. We used a linear mixed effects model to determine if MSP amounts were affected by topic application of Ban_PBAN using the following model: *log MSP amount ~ Ban_PBAN concentration (fixed) + time since application (fixed) + male age (random)*, as male age is known to affect MSP amounts [41,43].

In the second experiment, we aimed to increase the concentration of Ban_PBAN using injections. Ban_PBAN peptide was dissolved in buffer [150 mM NaCI/3mM CaCl2/3 mM KCl/10 mM N-tris(hydroxywmethyl)methyl2-aminoethanesulfonic acid buffer, adjusted to pH 6.9] [99] and diluted to a concentration of 0 (control), 50 or 100 ng/µl. Prior to injections, 7-day old male butterflies were incapacitated using nitrogen gas. One µl of solution was then injected using a 33 gauge 10 µl Hamilton syringe (Hamilton Company, USA) and males sampled two hours after injection, based on the observation that changes in MSP amounts were largest 2 hours after Ban_PBAN application in experiment 1 (n=57). We used a general linear model to determine if MSP amounts were affected by the injection of Ban_PBAN using the following model: *MSP amount ~ Ban_PBAN concentration* (MSP1 data were log transformed to improve normality).

98. Abernathy, R.L.; Teal, P.E.A.; Meredith, J.A.; Nachman, R.J. Induction of Pheromone Production in a Moth by Topical Application of a Pseudopeptide Mimic of a Pheromonotropic Neuropeptide. *Proc. Natl. Acad. Sci. U. S. A.* **1996**, *93*, 12621–12625.

99. Christensen, T.; Itagaki, H.; Teal, P.; Jasensky, R.; Tumlinson, J.; Hildebrand, J. Innervation and Neural Regulation of the Sex Pheromone Gland in Female Heliothis Moths. *Proc. Natl. Acad. Sci. U. S. A.* **1991**, *88*, 4971–4975.
